# Vinyl Thianthrenium Tetrafluoroborate: A Practical and Versatile Vinylating Reagent Made from Ethylene

**DOI:** 10.1021/jacs.1c06632

**Published:** 2021-08-10

**Authors:** Fabio Juliá, Jiyao Yan, Fritz Paulus, Tobias Ritter

**Affiliations:** Max-Planck-Institut für Kohlenforschung, Kaiser-Wilhelm Platz 1, D-45470 Mülheim an der Ruhr, Germany

## Abstract

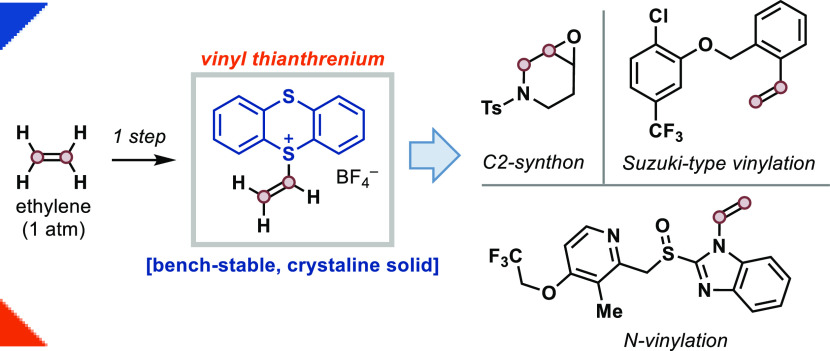

The use of vinyl electrophiles in synthesis has been hampered by the lack of access to a suitable reagent that is practical and of appropriate reactivity. In this work we introduce a vinyl thianthrenium salt as an effective vinylating reagent. The bench-stable, crystalline reagent can be readily prepared from ethylene gas at atmospheric pressure in one step and is broadly useful in the annulation chemistry of (hetero)cycles, N-vinylation of heterocyclic compounds, and palladium-catalyzed cross-coupling reactions. The structural features of the thianthrene core enable a distinct synthesis and reactivity profile, unprecedented for other vinyl sulfonium derivatives.

Owing to the rich chemistry of alkenes, the presence of a terminal alkenyl (vinyl, C_2_H_3_) substituent enables a myriad of opportunities for diversification and elaboration via dihydroxylation, carbofunctionalization, Heck-type arylation, hydroamination, and metathesis, among others.^1−6^ However, the introduction of a vinyl group as a C-2 building block is currently difficult, in contrast to the extended use of other substituted alkenyl electrophiles, in view of the lack of suitable reagents with the desired properties and reactivity profile. Here we report the reagent vinyl thianthrenium tetrafluoroborate (vinyl-TT^+^, **1**) that functions as a versatile reagent for different synthetic transformations. Reagent **1** is accessible directly from ethylene (1 atm) in a single step from commercially available material on multigram scale and is a bench-stable, nonhygroscopic solid that can be stored at room temperature in air without signs of decomposition for at least one year. Despite its high stability, **1** displays a rich reactivity profile and has been implemented in several polar and palladium-catalyzed cross-coupling reactions, which differentiates it from all other vinylating reagents reported to date. The unusual direct conversion of ethylene into a versatile building block for organic synthesis sets the approach apart from previous syntheses of alkenylsulfonium salts; in addition, **1** can participate in useful reactions such as a Suzuki cross-coupling that have not been realized with other alkenylthianthrenium salts.

Ethylene is an inexpensive gas (annual production >100 million tons),^7^ but its use in organic synthesis is rare and typically limited to simple substrates without high levels of complexity.^8^ One of the main drawbacks of the use of ethylene is the high temperature and pressures that are generally required for its conversion. In fact, reactions engaging ethylene at atmospheric pressure (1 atm) are uncommon and almost exclusive to metal-mediated reactions, owing to the ability of metal centers to activate ethylene via coordination.^9−14^ Metal-free reactions utilizing ethylene at 1 atm are manily restricted to photochemical cycloadditions with high-energy UV light.^15−17^ Overall, the general requirement for specialized equipment (high-pressure reactors or UV-photoreactors) has traditionally restricted the use of ethylene as a reagent in organic synthesis involving complex small molecules.

The development of palladium-catalyzed cross-coupling reactions has allowed researchers to reliably construct C–Csp^2^ bonds (Figure 1A).^18,19^ However, in contrast to the widespread use of alkenyl (substituted vinyl) derivatives, the use of vinyl halides as electrophiles (e.g., vinyl bromide) is challenging owing to the difficulty of handling the gaseous compounds that are acutely toxic and carcinogenic, which has historically thwarted their utilization in synthesis.^20^ Alternatively, numerous nucleophilic vinyl-[M] reagents ([M] = SnBu_3_, SiMe_3_, B(OR)_2_, etc.) have been developed over the years,^21^ but most of them are prepared in several steps from vinyl bromide itself, display high toxicity and low stability, or are poorly reactive (Figure 1B). Moreover, while significant advances have been accomplished with vinyl nucleophiles, the development of electrophilic derivatives that can effectively display the reactivity profile of vinyl halides is significantly less accomplished, and none of them are suitable as Michael acceptors for the direct polar addition of nucleophiles. Jimenez,^22^ Mukaiyama,^23^ and Aggarwal^24^ have developed the use of vinyl diphenylsulfonium salts^25^ as a 1,2-ethane dication synthon. This hygroscopic oil, prepared in three steps from bromoethanol, displays some practicality issues^26^ and is often generated in situ from its precursor bromoethyl diphenylsulfonium triflate.^27^ Over the past two decades, Aggarwal and others have reported a series of elegant transformations applying this reagent to the synthesis of (hetero)cycles.^24,28−34^ However, neither the reagent nor its precursors have ever been reported as suitable electrophiles in cross-coupling reactions owing to their fundamental reactivity profile (vide infra). In fact, only a few substituted alkenyl sulfonium salts have been successfully engaged in cross-couplings,^35−37^ but no examples of vinylations have been reported. Our group recently reported the synthesis of alkenyl thianthrenium salts,^38^ but a general reactivity profile in polar and cross-couling reactions has not been explored yet. Moreover, we were unsuccessful in engaging these salts in efficient couplings with aryl boronic acids via Suzuki-type reactions.

**Figure 1:**
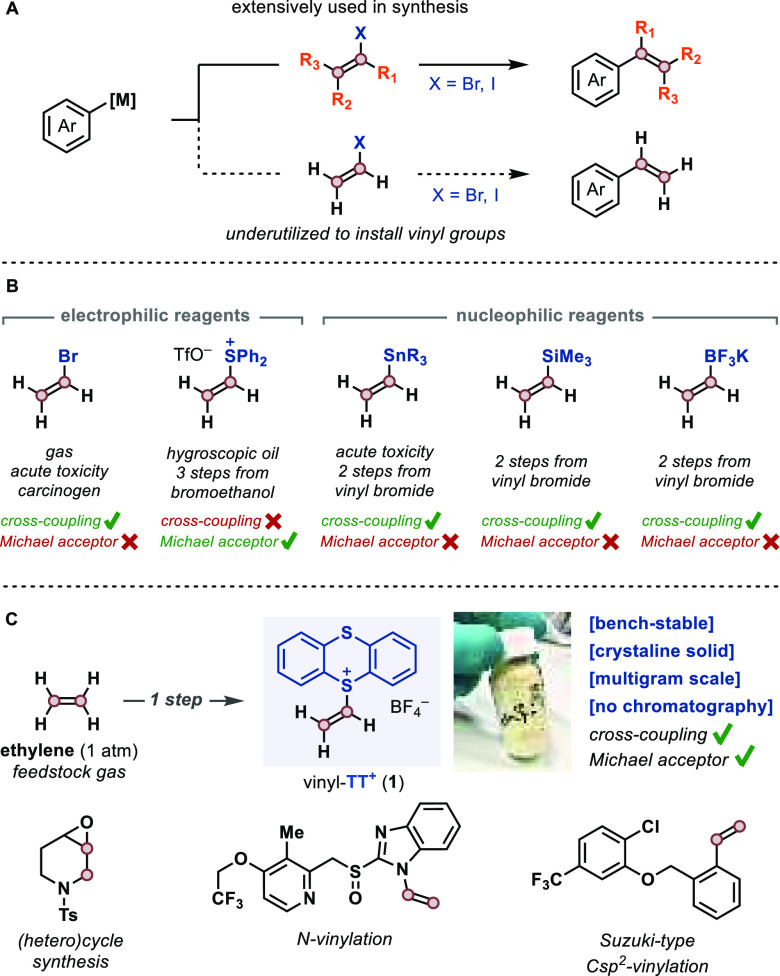
(A) Use of alkenyl electrophiles in cross-coupling reactions. (B) Commonly used vinylating reagents. (C) Vinyl thianthrenium salt **1** can be accessed directly from ethylene and is a versatile C-2 building block.

We recently aimed to design a strategy to trap ethylene efficiently and convert it into a practical and crystalline reagent (Figure 1C). Ideally, this new species should be stable and easy to handle, while simultaneously exhibiting a rich reactivity profile. We questioned whether vinyl thianthrenium salts (vinyl-TT^+^) could provide a valuable solution to this task. The perchlorate salt of vinyl-TT^+^ was published three decades ago while exploring the reactivity of thianthene radical cation (TT^•+^) with (vinyl)_4_Sn,^39^ but only milligram quantities were accessed owing to the involvement of potentially explosive perchlorate salts,^40^ and its synthetic use has never been reported. While, in comparison with other olefins, ethylene gas typically requires high pressure and an autoclave for cycloaddition reactions,^8,41^ we sought to capitalize on the high reactivity of the highly electrophilic thianthrenium dication species generated by treatment of thianthrene-*S*-oxide (**2**) with activating reagents such as Tf_2_O (Figure 2A). Following our new protocol, vinyl thianthrenium tetrafluoroborate (**1**) can now be prepared on multigram scale (50 mmol) with a simple balloon of ethylene (1 atm) in 86% yield. The isolation of **1** as a crystalline solid can be carried out by simple precipitation, to afford an analytically pure compound without the need for further purification. An alternative lab-scale synthetic route from vinyl-SiMe_3_ (2 equiv) was similarly effective (96% yield, see Supporting Information). The salt **1** is a nonhygroscopic solid that can be stored in the presence of air and moisture without signs of decomposition for at least one year, which makes it practical and easy-to-handle. DSC-TGA reveals that **1** does not decompose at temperatures lower than 280 °C, which underscores a desirable safety profile (Figure S6). In contrast, attempts of implementing this protocol using other sulfoxides such as dibenzothiophene-*S*-oxide or diphenylsulfoxide were unsuccessful (Figures S7 and S8). The structural features of thianthrene that allow the formation of a [4 + 2] adduct with ethylene seem crucial for a productive reaction, and indeed has enabled the first report on the formation of sulfonium salts directly from ethylene gas. To further confirm the key role of the [4 + 2] cycloadduct under the reaction conditions, we isolated and characterized intermediate **3**, the crystal structure of which shows the “snapshot” of ethylene activation by the formal thianthrenium dication (Figure 2B). No other known vinylating reagent can currently be accessed directly from ethylene, and the practical and conceptual advantages of **1** allow a rich and divergent chemistry (see below) while avoiding certain limitations associated with other reagents.

**Figure 2:**
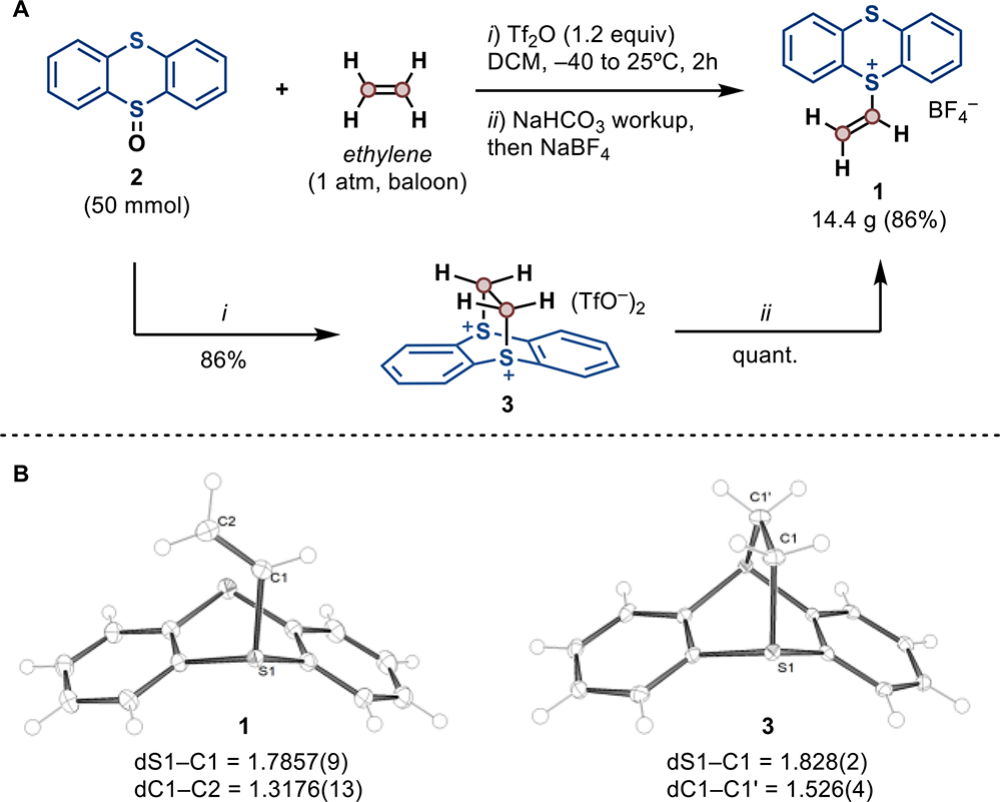
(A) Synthesis of **1** from ethylene, proceeding through a formal [4 + 2] cycloadduct (**3**) as intermediate. (B) Crystal structures of **1** and **3** obtained by X-ray diffraction (counterions omitted for simplicity).

To evaluate the reactivity profile of **1** we started benchmarking the reagent in annulation reactions reported for vinyl-SPh_2_(OTf) or its precursor, which proceed via sulfonium ylide intermediates.^25^ As depicted in Figure 3, we can access (hetero)cyclic motifs that are prevalent in bioactive compounds and pharmaceuticals. Selected examples include a cyclopropanation reaction (**4** → **5**),^28^ the assembly of morpholine (**6** → **7**)^24^ and azetidine (**8** → **9**)^31^ scaffolds, and a tandem N-nucleophilic addition/Corey–Chaykovsky epoxidation (**10** → **11**).^32^ In all cases, the isolated yields were comparable or superior to those obtained with vinyl-SPh_2_(OTf) under the same conditions.

**Figure 3:**
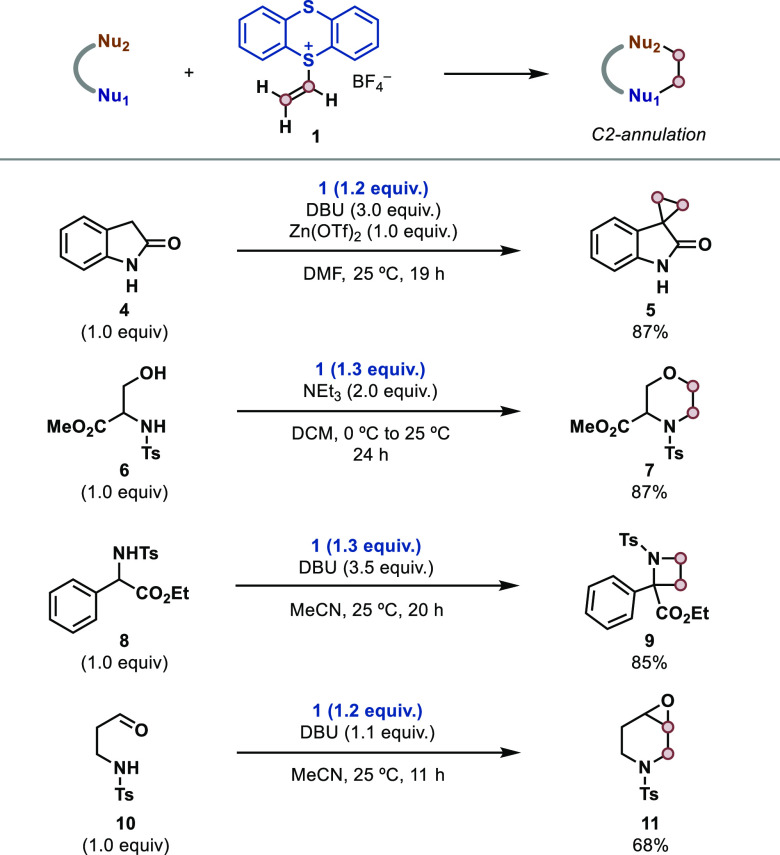
Application of **1** in the annulation of hetero- and carbocycles.

Next, we aimed to implement **1** in new reactions to effectively transfer the vinyl moiety to nucleophilic nitrogen. During his studies on annulation reactions, Aggarwal reported an annulation–vinylation sequence on 1,2-aminoalcohols^42^ only when Cbz is the N-protecting group, but, beyond these examples, a general platform for N-vinylation of heterocycles using sulfonium salts is not yet established^43^ and current methods require harsh conditions^44−47^ or metal-mediated reactions (85–100 °C).^48−51^ We developed a simple protocol that uses **1** in the presence of a base at room temperature (Table 1). A diverse set of N-vinylated nitrogen heterocycles can now be accessed under mild conditions including azacarbazole (**12**), indole (**13** and **14**), imidazole (**19**), pyrazole (**15** and **16**), triazole (**17**), and pyridone (**18**). A broad tolerance to an array of polar groups was displayed as demonstrated by the compatibility of nitro (**13**) and aldehyde groups (**14**), which are not tolerated using calcium carbide,^47^ or aryl halides (**16**, **20**) that are reactive in S_N_Ar and cross-coupling reactions. Other scaffolds of relevance such as deazapurine (**20**) or theophylline (**21**) were also vinylated, as well as the amino acids tryptophan and histidine (**22** and **23**). Finally, we explored the use of **1** for late-stage N-vinylation. The mild conditions and fast reaction times enabled modification of the drugs metaxalone (**24**), carvedilol (**25**), lansoprazole (**27**), and the laser dye coumarin 7 (**26**), further showcasing the compatibility with groups such as alcohols, alkylamines, and sulfoxides.

**Table 1:**
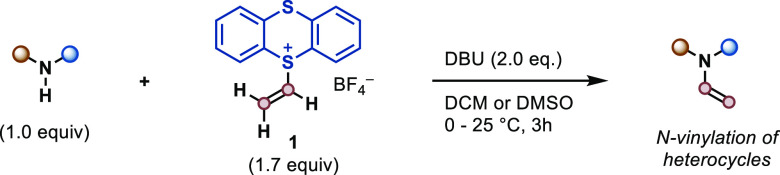

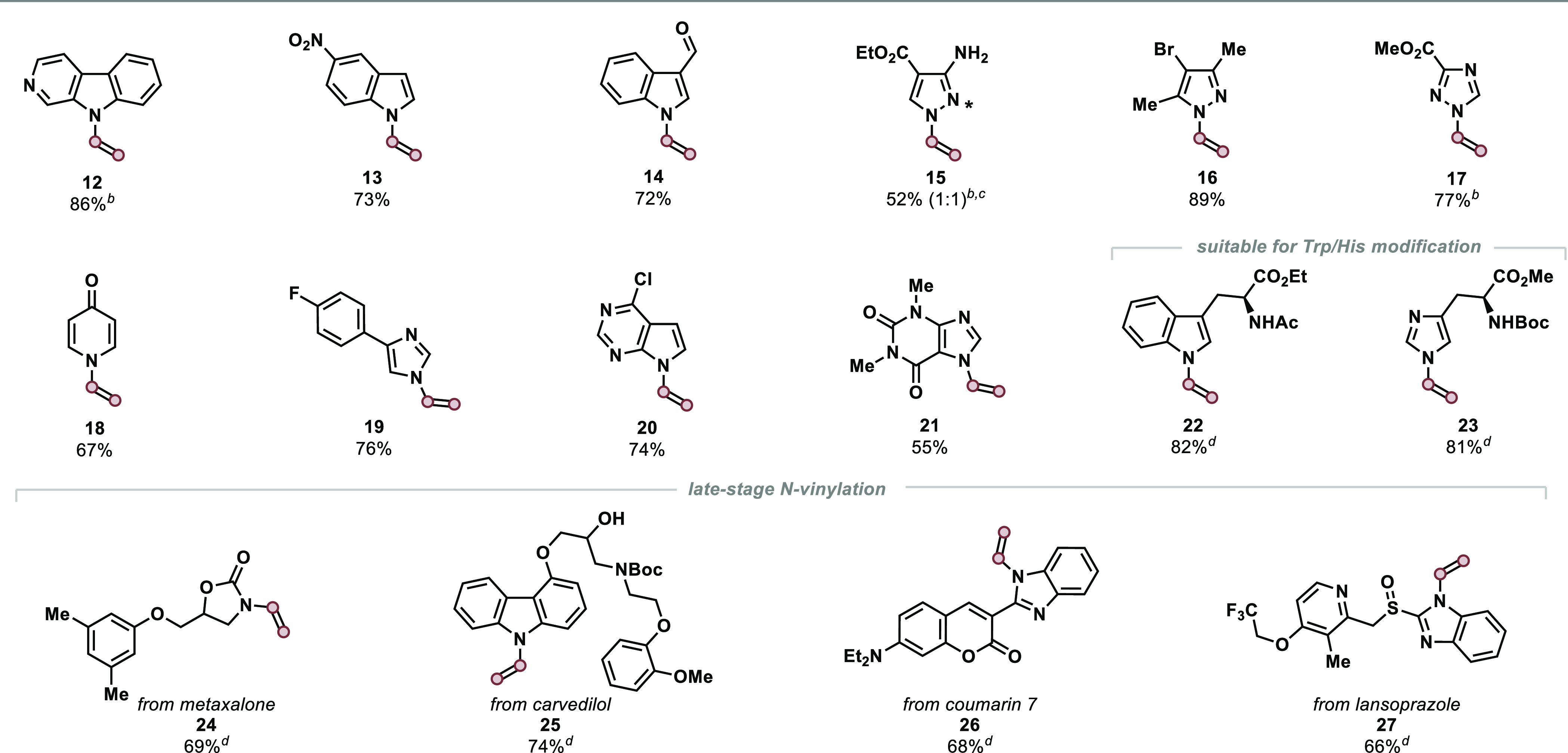
Vinylation of N-Heterocycles Using **1**^a^

a Reaction conditions: 0.300 mmol of N-heterocycle, 1.7 equiv of **1**, 2.0 equiv of DBU in CH_2_Cl_2_ (3.0 mL, *c* = 0.10 M), 25 °C, 3 h.

b DMSO was used as solvent.

c 1.2 equiv of **1** were added to a solution of N-heterocycle and DBU.

d 30 min at 0 °C, then 2.5 h at 25 °C. * denotes the site of vinylation on the constitutional isomer not shown.

Vinylated arenes (styrenes) are activated alkenes with widespread use in transition-metal catalysis,^52,53^ radical chemistry,^54,55^ and electrophilic reactions.^56,57^ In contrast to alkenylation, the assembly of styrenes using vinylating reagents in metal-catalyzed cross-couplings often face several additional challenges,^21^ such as undesired Heck-type reactivity on the vinyl–[M] reagent or polymerization styrene-type products. Vinyl sulfonium salts are ideally positioned to undergo metal-catalyzed vinylations, but no examples have been reported. One of the main reasons is the unselective cleavage of the different C–S bonds in sulfonium salts,^37^ which can result in mixtures of products. We conceived **1** as a suitable coupling partner that could overcome the above-mentioned challenges and enable fast oxidative addition in view of its electropositive character (*E*_red_ = −1.13 V vs SCE). Moreover, in line with what has been observed in palladium-catalyzed reactions of aryl thianthrenium salts,^58−63^ cleavage of the C_vinyl_–S bond selectively over the two C_aryl_–S bonds may be explained by irreversible oxidative addition into the vinyl bond but reversible oxidative addition into the aryl bond^64,65^ of the annulated structure of the thianthrene core (see Supporting Information for a discussion). To demonstrate the performance of **1** in cross-coupling reactions, we developed a Suzuki-type vinylation of aryl boronic acids (Table 2A). The scope of aryl boronic acids encompasses a wide range of (hetero)arenes with different functional groups and substitution patterns (**28**–**36**), including electrophilic groups that are not tolerated by Wittig olefination-based synthesis^66^ (**34**, **39**), with proto-deborylation observed as the main side reaction in those examples with lower yields. The fast rate of oxidative addition of the C–S bond allowed the vinylation of substrates containing C–Br bonds (**33**) that are otherwise reactive in Suzuki reactions.^19^ Likewise, a competition experiment between vinyl thianthrenium **1**-*d*_3_ and vinyl bromide established that the thianthrenium compound reacts substantially faster than vinyl bromide; less than 1% of reaction product based on vinyl bromide could be detected by either NMR spectroscopy or mass spectrometry analysis (Table 2B). Extension of the methodology to other organoboron compounds, such as boronic esters (**38**) and trifluoroborates (**39**), is possible. Alkenyl boronic acids were also suitable substrates, yielding valuable dienes (**40**) that can be employed for further elaboration (e.g., Diels–Alder reactions).

**Table 2:**
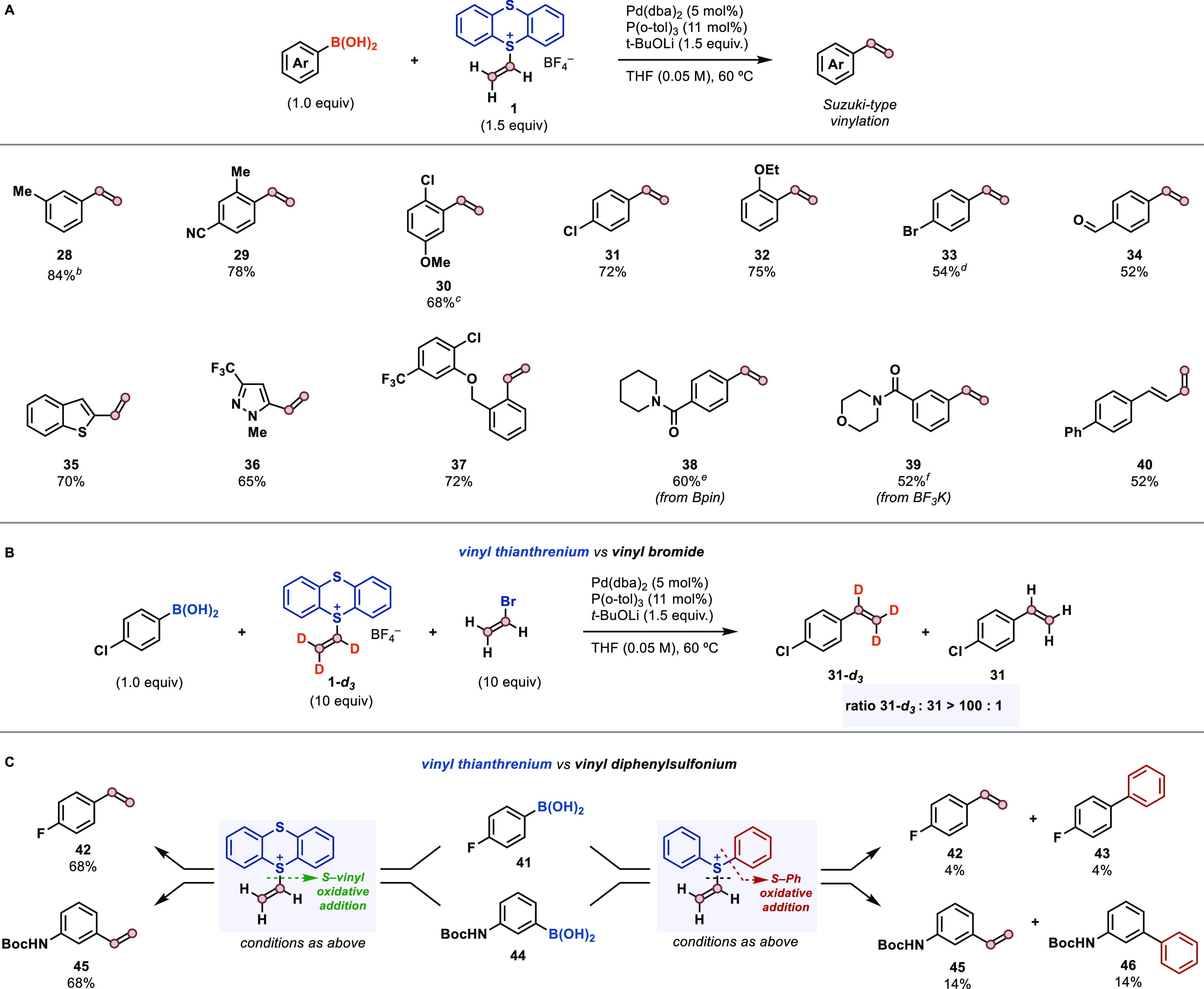
Suzuki-Type Vinylation of Organoboron Compounds^a^

(A) Scope of the transformation. (B) Competition experiment between 1-*d*_3_ and vinyl bromide; analysis by NMR spectroscopy and mass spectrometry. (C) Comparison of the reactivity of **1** and vinyl-SPh_2_(OTf).

a Reaction conditions: 0.300 mmol of ArB(OH)_2_, 1.5 equiv of **1**, 0.050 equiv of Pd(dba)_2_, 0.11 equiv of P(*o*-tol)_3_, 1.5 equiv of *t*-BuOLi in THF (6.0 mL, *c* = 0.05 M), 60 °C, 16 h.

b NMR yield.

c K_2_CO_3_ was used as base.

d 1.7 equiv of **1**, 50 °C, 24 h.

e From ArBpin.

f From ArBF_3_K.

In contrast, the use of vinyl-SPh_2_(OTf) under the same reaction conditions did not afford the desired products or resulted in <15% yield in all the cases studied (Table 2C). For example, while styrene **42** was isolated in 68% yield when using reagent **1**, only a 4% yield could be detected by NMR when using vinyl-SPh_2_(OTf), which may be the result of a faster reagent decomposition or catalyst poisoning. Moreover, analysis of the reaction mixture revealed the presence of equimolar amounts of side-product **43**, arising from aryl–Ph instead of aryl–vinyl bond formation, while no related product resulting from aryl–aryl coupling could be detected in the reaction with **1**. A similar outcome was observed with substrate **44**. These results underline the key benefits of the structural design of thianthrene electrophiles, effectively channelling the oxidative addition process toward the desired C–S bond and allowing, for the first time, a vinylation reaction based on cross-coupling with vinyl sulfonium salts.

In summary, we have developed a convenient vinyl electrophile reagent that is prepared directly from ethylene and can be stored in the presence of air and moisture. The salt has proven to be an effective vinylating reagent and C-2 synthon for the synthesis of carbo- and heterocycles, N-vinylated products, styrenes, and dienes. The distinct structural features of thianthrenium salts in comparison with other sulfonium salts enable both its unique synthesis from ethylene and its superior performance in cross-coupling reactions. Its one-step synthesis, easy-to-handle features, and robust reactivity make it a valuable and versatile reagent that will find synthetic utility in further organic and transition-metal catalyzed transformations.

## Supporting Information Available

Experimental procedures and spectral data (PDF)

## Accession Codes

CCDC 2075820–2075821 contain the supplementary crystallographic data for this paper. These data can be obtained free of charge via www.ccdc.cam.ac.uk/data_request/cif, or by emailing data_request@ccdc.cam.ac.uk, or by contacting The Cambridge Crystallographic Data Centre, 12 Union Road, Cambridge CB2 1EZ, UK; fax: +44 1223 336033.
