# Supplementary material for: Vinyl Thianthrenium Tetrafluoroborate: A Practical and Versatile Vinylating Reagent Made from Ethylene
Source: J Am Chem Soc. 2021 Aug 10;143(33):12992–8. doi: 10.1021/jacs.1c06632 (PMC8391941; doi:10.1021/jacs.1c06632)
Supplement: Supplementary file 1 — ja1c06632_si_001.pdf [file ja1c06632_si_001.pdf]

SUPPORTING INFORMATION

Vinyl thianthrenium tetrafluoroborate: A practical and versatile vinylating reagent made from ethylene

Fabio Juliá, Jiyao Yan, Fritz Paulus and Tobias Ritter*

Max-Planck-Institut für Kohlenforschung

Kaiser-Wilhelm Platz 1, 45470 Mülheim an der Ruhr, Germany

*E-mail: ritter@mpi-muelheim.mpg.de

TABLE OF CONTENTS

TABLE OF CONTENTS 1

MATERIALS AND METHODS 7

EXPERIMENTAL DATA 8

Preparation of vinyl TT+ **1** 8

Preparation of **1** from ethylene 8

Preparation of **1** from vinyltrimethylsilane 9

Preparation of **1-*d*$_3$** from ethylene-*d*$_4$ 13

Preparation of [4+2] cycloadduct intermediate **3** 14

Reaction of other sulfoxides with ethylene 14

Reaction of diphenylsulfoxide with ethylene 14

Reaction of dibenzothiophene-S-oxide with ethylene 15

Preparation of vinyl-SPh$_2$(OTf) (**S3**) 16

Annulation reactions employing vinyl-TT+ **1** 17

Spiro[cyclopropane-1,3'-indolin]-2'-one (**5**) 17

Methyl (±)-4-tosylmorpholine-3-carboxylate (**7**) 18

Ethyl (±)-2-phenyl-1-tosylazetidine-2-carboxylate (**9**) 18

(±)-3-Tosyl-7-oxa-3-azabicyclo[4.1.0]heptane (**11**) 19

Vinylation of *N*-heterocycles using vinyl-TT+ 20

General procedure A 20

9-Vinyl-9*H*-pyrido[3,4-*b*]indole (**12**) 20

5-Nitro-1-vinyl-1*H*-indole (**13**) 21

1-Vinyl-1*H*-indole-3-carbaldehyde (**14**) 21

Ethyl 3-amino-1-vinyl-1H-pyrazole-4-carboxylate (**15-I**) and ethyl 5-amino-1-vinyl-1H-pyrazole-4-carboxylate (**15-II**) 22

4-Bromo-3,5-dimethyl-1-vinyl-1*H*-pyrazole (**16**) 23

Methyl 1-vinyl-1*H*-1,2,4-triazole-3-carboxylate (**17**) 23

1-Vinylpyridin-4(1*H*)-one (**18**) 24

4-(4-Fluorophenyl)-1-vinyl-1*H*-imidazole (**19**) 24

4-Chloro-7-vinyl-7*H*-pyrrolo[2,3-*d*]pyrimidine (**20**) 25

N-Vinyl-theophylline (**21**) 26

N-Vinyl,*N'*-Acetyl-L-tryptophan ethyl ester (**22**) 26

N-Vinyl,*N'*-Boc-L-histidine methyl ester (**23**) 27

N-Vinyl-metaxalone (**24**) 27

N-Vinyl,*N'*-Boc-carvedilol (**25**) 28
N-Vinyl-coumarin 7 (**26**) 29
N-Vinyl-lansoprazole (**27**) 29

Reaction optimization of the vinylation of organoboron compounds 30

General procedure for optimization of reaction conditions 30
Table S1: Optimization of yield as a function of catalyst 30
Table S2: Optimization of yield as a function of base 31
Table S3: Optimization of yield as a function of concentration 32

Suzuki-type vinylation of aryl organoboron compounds using vinyl-TT^+ **1** 32

General procedure B 32
General procedure for the vinylation of organoboron compounds using a Schlenk line 33
1-Methyl-3-vinylbenzene (**28**) 33
3-Methyl-4-vinylbenzonitrile (**29**) 34
1-Chloro-4-methoxy-2-vinylbenzene (**30**) 35
1-Chloro-4-vinylbenzene (**31**) 36
1-Chloro-4-(vinyl-d_3)benzene (**31-d_3**) 36
1-Ethoxy-2-vinylbenzene (**32**) 37
1-Bromo-4-vinylbenzene (**33**) 38
4-Vinylbenzaldehyde (**34**) 39
2-Vinylbenzo[b]thiophene (**35**) 39
1-Methyl-3-(trifluoromethyl)-5-vinyl-1*H*-pyrazole (**36**) 40
1-Chloro-4-(trifluoromethyl)-2-((2-vinylbenzyl)oxy)benzene (**37**) 41
Piperidin-1-yl(4-vinylphenyl)methanone (**38**) 42
Morpholino(3-vinylphenyl)methanone (**39**) 43
(*E*)-4-(Buta-1,3-dien-1-yl)-1,1'-biphenyl (**40**) 43

Competition experiment between **1-d_3** and vinyl bromide 44

Preparation of vinyl bromide solution in THF 44
Competition experiment 44

Comparison on the performance of 1 and vinyl-SPh_2(OTf) in Suzuki-type reactions. 46

General procedure C 46
tert-Butyl (3-vinylphenyl)carbamate (**45**) 47
1-Fluoro-4-vinylbenzene (**42**) 48
Discussion 50

X-RAY CRYSTALLOGRAPHIC ANALYSIS 52

S-vinyl-thianthrenium tetrafluoroborate (vinyl-TT^+, **1**) (CCDC 2075820) 52

Experimental 52

[4+2] cycloadduct intermediate **3** (CCDC 2075821) 54

Experimental 54

SPECTROSCOPIC DATA 57

^{1}H NMR of vinyl TT^{+} (**1**) 57

^{13}C NMR of vinyl TT^{+} (**1**) 58

^{19}F NMR of vinyl TT^{+} (**1**) 59

^{1}H NMR of vinyl TT^{+}-d_3 (**1-*d*$_3$**) 60

^{2}H NMR of vinyl TT^{+}-d_3 (**1-*d*$_3$**) 61

^{13}C NMR of vinyl TT^{+}-d_3 (**1-*d*$_3$**) 61

^{19}F NMR of vinyl TT^{+}-d_3 (**1-*d*$_3$**) 63

^{1}H NMR of the [4+2] cycloadduct intermediate **3** 64

^{13}C NMR of the [4+2] cycloadduct intermediate **3** 65

^{19}F NMR of the [4+2] cycloadduct intermediate **3** 66

^{1}H NMR of spiro[cyclopropane-1,3’-indolin]-2’-one (**5**) 67

^{13}C NMR of spiro[cyclopropane-1,3’-indolin]-2’-one (**5**) 68

^{1}H NMR of methyl (±)-4-tosylmorpholine-3-carboxylate (**7**) 69

^{13}C NMR of methyl (±)-4-tosylmorpholine-3-carboxylate (**7**) 70

^{1}H NMR of ethyl (±)-2-phenyl-1-tosylazetidine-2-carboxylate (**9**) 71

^{13}C NMR of ethyl (±)-2-phenyl-1-tosylazetidine-2-carboxylate (**9**) 72

^{1}H NMR of (±)-3-tosyl-7-oxa-3-azabicyclo[4.1.0]heptane (**11**) 73

^{13}C NMR of (±)-3-tosyl-7-oxa-3-azabicyclo[4.1.0]heptane (**11**) 74

^{1}H NMR of 9-vinyl-9*H*-pyrido[3,4-*b*]indole (**12**) 75

^{13}C NMR of 9-vinyl-9*H*-pyrido[3,4-*b*]indole (**12**) 76

^{1}H NMR of 5-nitro-1-vinyl-1*H*-indole (**13**) 77

^{13}C NMR of 5-nitro-1-vinyl-1*H*-indole (**13**) 78

^{1}H NMR of 1-vinyl-1*H*-indole-3-carbaldehyde (**14**) 79

^{13}C NMR of 1-vinyl-1*H*-indole-3-carbaldehyde (**14**) 80

^{1}H NMR of ethyl 3-amino-1-vinyl-1H-pyrazole-4-carboxylate (15-I) 81

^{13}C NMR of ethyl 3-amino-1-vinyl-1H-pyrazole-4-carboxylate (**15-I**) 82

^{1}H NMR of ethyl 5-amino-1-vinyl-1H-pyrazole-4-carboxylate (**15-II**) 83

^{13}C NMR of ethyl 5-amino-1-vinyl-1H-pyrazole-4-carboxylate (**15-II**) 84

^{1}H NMR of 4-bromo-3,5-dimethyl-1-vinyl-1*H*-pyrazole (**16**) 85

^{13}C NMR of 4-bromo-3,5-dimethyl-1-vinyl-1*H*-pyrazole (**16**) 86

^{1}H NMR of methyl 1-vinyl-1*H*-1,2,4-triazole-3-carboxylate (**17**) 87

^{13}C NMR of methyl 1-vinyl-1*H*-1,2,4-triazole-3-carboxylate (**17**) 88

^{1}H NMR of 1-vinylpyridin-4(1*H*)-one (**18**) 89

^{13}C NMR of 1-vinylpyridin-4(1*H*)-one (**18**) 90

^{1}H NMR of 4-(4-fluorophenyl)-1-vinyl-1*H*-imidazole (**19**) 91

^{13}C NMR of 4-(4-fluorophenyl)-1-vinyl-1*H*-imidazole (**19**) 92

^{19}F NMR of 4-(4-fluorophenyl)-1-vinyl-1*H*-imidazole (**19**) 93

^{1}H NMR of 4-chloro-7-vinyl-7*H*-pyrrolo[2,3-*d*]pyrimidine (**20**) 94

^{13}C NMR of 4-chloro-7-vinyl-7*H*-pyrrolo[2,3-*d*]pyrimidine (**20**) 95

^{1}H NMR of *N*-vinyl-theophylline (**21**) 96

^{13}C NMR of *N*-vinyl-theophylline (**21**) 97

^{1}H NMR of *N*-vinyl-*N'*-acetyl-L-tryptophan ethyl ester (**22**) 98

^{13}C NMR of *N*-vinyl-*N'*-acetyl-L-tryptophan ethyl ester (**22**) 99

^{1}H NMR of *N*-vinyl-*N'*-Boc-L-histidine methyl ester (**23**) 100

^{13}C NMR of *N*-vinyl-*N'*-Boc-L-histidine methyl ester (**23**) 101

^{1}H NMR of *N*-vinyl-metaxalone (**24**) 102

^{13}C NMR of *N*-vinyl-metaxalone (**24**) 103

^{1}H NMR of *N*-vinyl-*N'*-Boc-carvedilol (**25**) 104

^{13}C NMR of *N*-vinyl-*N'*-Boc-carvedilol (**25**) 105

^{1}H NMR of *N*-vinyl-coumarin 7 (**26**) 106

^{13}C NMR of *N*-vinyl-coumarin 7 (**26**) 107

^{1}H NMR of *N*-vinyl-lansoprazole (**27**) 108

^{13}C NMR of *N*-vinyl-lansoprazole (**27**) 109

^{19}F NMR of *N*-vinyl-lansoprazole (**27**) 110

^{1}H NMR of 3-methyl-4-vinylbenzonitrile (**29**) ... 111

^{13}C NMR of 3-methyl-4-vinylbenzonitrile (**29**) ... 111

^{1}H NMR of 1-chloro-4-methoxy-2-vinylbenzene (**30**) ... 112

^{13}C NMR of 1-chloro-4-methoxy-2-vinylbenzene (**30**) ... 114

^{1}H NMR of 1-chloro-4-vinylbenzene (**31**) ... 114

^{13}C NMR of 1-chloro-4-vinylbenzene (**31**) ... 116

^{1}H NMR of 1-chloro-4-(vinyl-d_3)benzene (**31-d_3**) ... 117

^{2}H NMR of 1-chloro-4-(vinyl-d_3)benzene (**31-d_3**) ... 118

^{13}C NMR of 1-chloro-4-(vinyl-d_3)benzene (**31-d_3**) ... 119

^{1}H NMR of 1-ethoxy-2-vinylbenzene (**32**) ... 120

^{13}C NMR of 1-ethoxy-2-vinylbenzene (**32**) ... 121

^{1}H NMR of 1-bromo-4-vinylbenzene (**33**) ... 122

^{13}C NMR of 1-bromo-4-vinylbenzene (**33**) ... 123

^{1}H NMR of 4-vinylbenzaldehyde (**34**) ... 124

^{13}C NMR of 4-vinylbenzaldehyde (**34**) ... 125

^{1}H NMR of 2-vinylbenzo[b]thiophene (**35**) ... 126

^{13}C NMR of 2-vinylbenzo[b]thiophene (**35**) ... 127

^{1}H NMR of 1-methyl-3-(trifluoromethyl)-5-vinyl-1*H*-pyrazole (**36**) ... 128

^{13}C NMR of 1-methyl-3-(trifluoromethyl)-5-vinyl-1*H*-pyrazole (**36**) ... 129

^{19}F NMR of 1-methyl-3-(trifluoromethyl)-5-vinyl-1*H*-pyrazole (**36**) ... 130

^{1}H NMR of 1-chloro-4-(trifluoromethyl)-2-((2-vinylbenzyl)oxy)benzene (**37**) ... 131

^{13}C NMR of 1-chloro-4-(trifluoromethyl)-2-((2-vinylbenzyl)oxy)benzene (**37**) ... 131

^{19}F NMR of 1-chloro-4-(trifluoromethyl)-2-((2-vinylbenzyl)oxy)benzene (**37**) ... 133

^{1}H NMR of piperidin-1-yl(4-vinylphenyl)methanone (**38**) ... 134

^{13}C NMR of piperidin-1-yl(4-vinylphenyl)methanone (**38**) ... 134

^{1}H NMR of morpholino(3-vinylphenyl)methanone (**39**) ... 136

^{13}C NMR of morpholino(3-vinylphenyl)methanone (**39**) ... 137

^{1}H NMR of (*E*)-4-(buta-1,3-dien-1-yl)-1,1'-biphenyl (**40**) ... 138

^{13}C NMR of (*E*)-4-(buta-1,3-dien-1-yl)-1,1'-biphenyl (**40**) ... 139

^{1}H NMR of *tert*-butyl (3-vinylphenyl)carbamate (**45**) 140

^{13}C NMR of *tert*-butyl (3-vinylphenyl)carbamate (**45**) 140

^{1}H NMR of *tert*-butyl [1,1'-biphenyl]-3-ylcarbamate (**46**) 141

^{13}C NMR of *tert*-butyl [1,1'-biphenyl]-3-ylcarbamate (**46**) 142

References 144

MATERIALS AND METHODS

All reactions were carried out under an ambient atmosphere unless otherwise stated and monitored by thin-layer chromatography (TLC). Air- and moisture-sensitive manipulations were performed using standard Schlenk- and glove-box techniques under an atmosphere of argon or dinitrogen. High-resolution mass spectra were obtained using Q Exactive Plus from Thermo. Concentration under reduced pressure was performed by rotary evaporation at 25–40 °C at an appropriate pressure. When it was not removed by means of aqueous workup, DMSO was removed in a Biotage V10 evaporator. Purified compounds were further dried under vacuum (10^{-6} – 10^{-3} bar). Yields refer to purified and spectroscopically pure compounds, unless otherwise stated.

Solvents

Dichloromethane, dimethylsulfoxide, acetonitrile and diethyl ether were purchased from Fisher Scientific GmbH and used as received. Anhydrous solvents were obtained from Phoenix Solvent Drying Systems. All deuterated solvents were purchased from Euriso-Top.

Chromatography

Thin layer chromatography (TLC) was performed using EMD TLC plates pre-coated with 250 µm thickness silica gel 60 F_{254} plates and visualized by fluorescence quenching under UV light and $KMnO_4$ stain. Flash column chromatography was performed using silica gel (40–63 µm particle size) purchased from Geduran®.

Spectroscopy and Instruments

NMR spectra were recorded on a Bruker Ascend™ 500 spectrometer operating at 500 MHz, 471 MHz and 126 MHz, for ^{1}H, ^{19}F and ^{13}C acquisitions, respectively; or on a Varian Unity/Inova 600 spectrometer operating at 600 MHz and 151 MHz for ^{1}H and ^{13}C acquisitions, respectively; or on a Bruker Ultrashield™ 300 spectrometer operating at 300 MHz, 282 MHz and 75 MHz for ^{1}H, ^{19}F and ^{13}C acquisitions, respectively. Chemical shifts are reported in ppm with the solvent residual peak as the internal standard. For ^{1}H NMR: $CDCl_3$, δ 7.26; CD_3CN, δ 1.96; CD_2Cl_2, δ 5.32; For ^{13}C NMR: $CDCl_3$, δ 77.16; CD_3CN, δ 1.32; CD_2Cl_2, δ 53.84. ^{19}F NMR spectra were referenced using a unified chemical shift scale based on the ^{1}H resonance of tetramethylsilane (1% v/v solution in the respective solvent). Data is reported as follows: s = singlet, d = doublet, t = triplet, q = quartet, quin = quintet, sext = sextet, sept = septet, m = multiplet, bs = broad singlet; coupling constants in Hz; integration.

Starting materials

Chemicals were used as received from commercial suppliers, unless otherwise stated. Thianthrene *S*-oxide,[1] *N*-tosyl DL-serine methyl ester,[2] 4-methyl-*N*-(3-oxopropyl)benzenesulfonamide (tosylation of 3-amino-1-propanol,[3] then oxidation[4]), *N*-tosyl DL-phenylglycine ethyl ester,[5] dibenzothiophene-*S*-oxide,[6] and N-Boc-carvedilol[7] were synthesized according to literature reports. Zinc triflate was dried in a desiccator over P_2O_5.

EXPERIMENTAL DATA

Preparation of vinyl TT+ 1

Preparation of 1 from ethylene

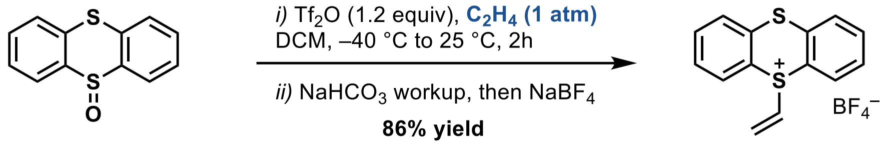

2 **vinyl-TT+** (**1**)

To a round-bottom flask equipped with a stirring bar were added thianthrene-*S*-oxide **2** (11.7 g, 50.3 mmol, 1.00 equiv.) and 400 mL of DCM (c= 0.125 M). The flask was capped with a rubber septum and cooled down to –40°C. Through the solution was then bubbled ethylene gas for 15 minutes, after which a balloon filled with ethylene was connected to the flask to maintain the ethylene atmosphere throughout the reaction. Triflic anhydride (10.2 mL, 17.0 g, 60.4 mmol, 1.20 equiv.) was added dropwise to the reaction, and a dark purple suspension was progressively formed *[Note: in large scale experiments the amount of precipitate formed can complicate an appropriate stirring. Additional portions of DCM may be added to aid stirring]*. After 20 minutes, the cooling bath was removed and the mixture was stirred for 1.5 hour at 25 °C. The ethylene balloon and the rubber septum were removed, and sat. aqueous $NaHCO_3$ (400 mL) was added carefully. The mixture was vigorously shaken in a separation funnel, phases were separated and the aqueous layer was extracted with DCM (2 × 200 mL). All organic phases were combined, partially concentrated (~300 mL), washed with aqueous solutions of 5% $NaBF_4$ (3 × 100 mL), dried over $MgSO_4$, filtered, and the solvent evaporated under reduced pressure. The crude material was dissolved in DCM (60 mL), and Et_2O (300 mL) was subsequently added while stirring, causing the precipitation of a solid that was collected by filtration, washed with Et_2O (3 × 20 mL), and dried under vacuum to afford **1** as an off-white solid (14.37 g, 43.52 mmol, 86%). *[Note: Colored trace impurities coming from old bottles of triflic anhydride might result in **1** as a pale yellow solid (even at >99% purity), but have no observable effect on the reactivity reported in this work].*

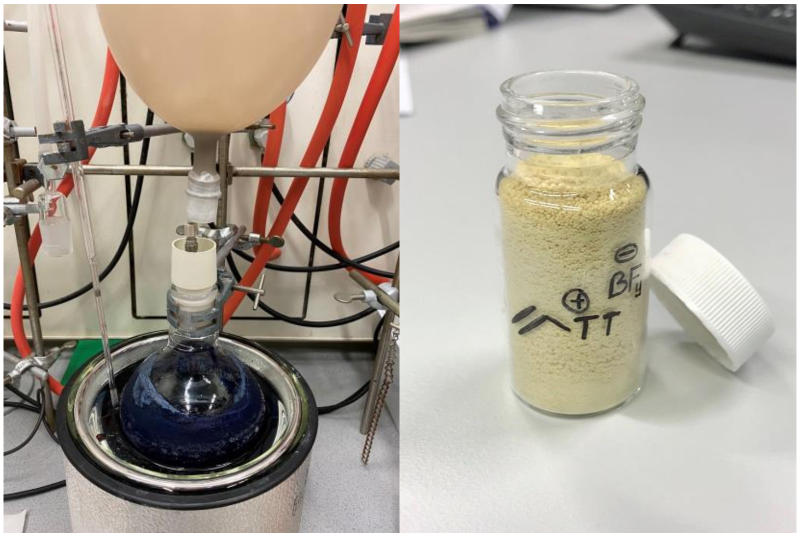

Figure S1. Pictures of the reaction setup (left) and the isolated product (right) on the preparation of **1**.

Preparation of 1 from vinyltrimethylsilane

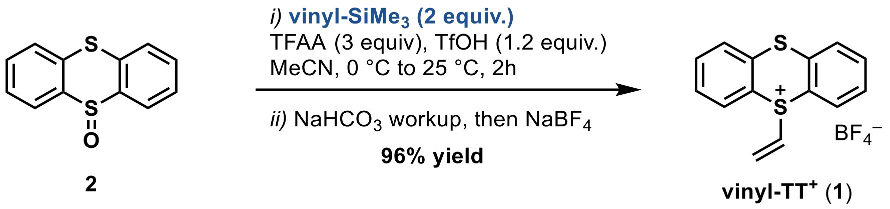

To a round-bottom flask equipped with a stirring bar were added thianthrene-*S*-oxide **2** (3.14 g, 13.5 mmol, 1.00 equiv.), 65 mL of MeCN (c= 0.20 M) and vinyl-$SiMe_3$ (4.0 mL, 2.7 g, 27 mmol, 2.0 equiv.). The solution was cooled down to 0 °C and trifluoroacetic anhydride (5.7 mL, 8.6 g, 41 mmol, 3.0 equiv.), and trifluoromethane sulfonic acid (1.4 mL, 2.4 g, 16 mmol, 1.2 equiv.) were subsequently added. The resultant mixture was stirred at 0 °C for 30 minutes, warmed up to 25 °C and stirred for additional 60 minutes. The mixture was concentrated under reduced pressure, diluted with DCM (200 mL), and washed with sat. aqueous $NaHCO_3$ (200 mL). The aqueous layer was extracted with DCM (2 × 50 mL). All organic phases were combined, washed with aqueous solutions of 5% $NaBF_4$ (2 × 100 mL), dried over $MgSO_4$, filtered, and the solvent evaporated under reduced pressure. The crude material was dissolved in DCM (15 mL), and Et_2O (100 mL) was subsequently added while stirring, causing the precipitation of a solid that was collected by filtration, washed with Et_2O (3 × 10 mL), and dried under vacuum to afford **1** as an off-white solid (4.30 g, 13.0 mmol, 96%).

NMR Spectroscopy:

^{1}**H NMR** (500 MHz, CD_3CN, 298 K, δ): 8.21 (dd, *J* = 7.9, 1.3 Hz, 2H), 8.00 (dd, *J* = 7.9, 1.3 Hz, 2H), 7.88 (td, *J* = 7.8, 1.4 Hz, 2H), 7.77 (td, *J* = 7.8, 1.4 Hz, 2H), 6.65 (dd, *J* = 15.9, 8.9 Hz, 1H), 6.32 (dd, *J* = 8.9, 3.1 Hz, 1H), 5.97 (dd, *J* = 16.0, 3.1 Hz, 1H).

^{13}**C NMR** (75 MHz, CD_3CN, 298 K, δ): 137.1, 135.9, 135.1, 134.3, 131.4, 130.9, 120.9, 119.1.

^{19}**F NMR** (282 MHz, CD_3CN, 298 K, δ): –151.38 (s), –151.43 (s).

HRMS-ESI (m/z) calculated for $C_{14}H_{11}S_2^+$ $[M–BF_4]^+$, 243.0297; found, 243.0298; deviation: –0.0 ppm.

IR (neat, thin film): $ν_{max}(cm^{-1})$ = 3086, 2360, 2341, 1568, 1450, 1435, 1385, 1288, 1267, 1033, 1032, 959, 762, 521, 459.

Melting point: 108–110 °C (recryst. solvents: DCM/Et_2O)

Elemental analysis calcd (%) for $C_{14}H_{11}BF_4S_2$: C 50.93, H 3.36, S 19.42; found: C 50.83, H 3.37, S 19.39.

No decomposition was observed after storing vinyl-TT^+ (**1**) between 20–28 °C under air. In Figure S2 is shown the ^{1}H NMR spectrum recorded for one sample kept in a vial with no further precautions for one year:

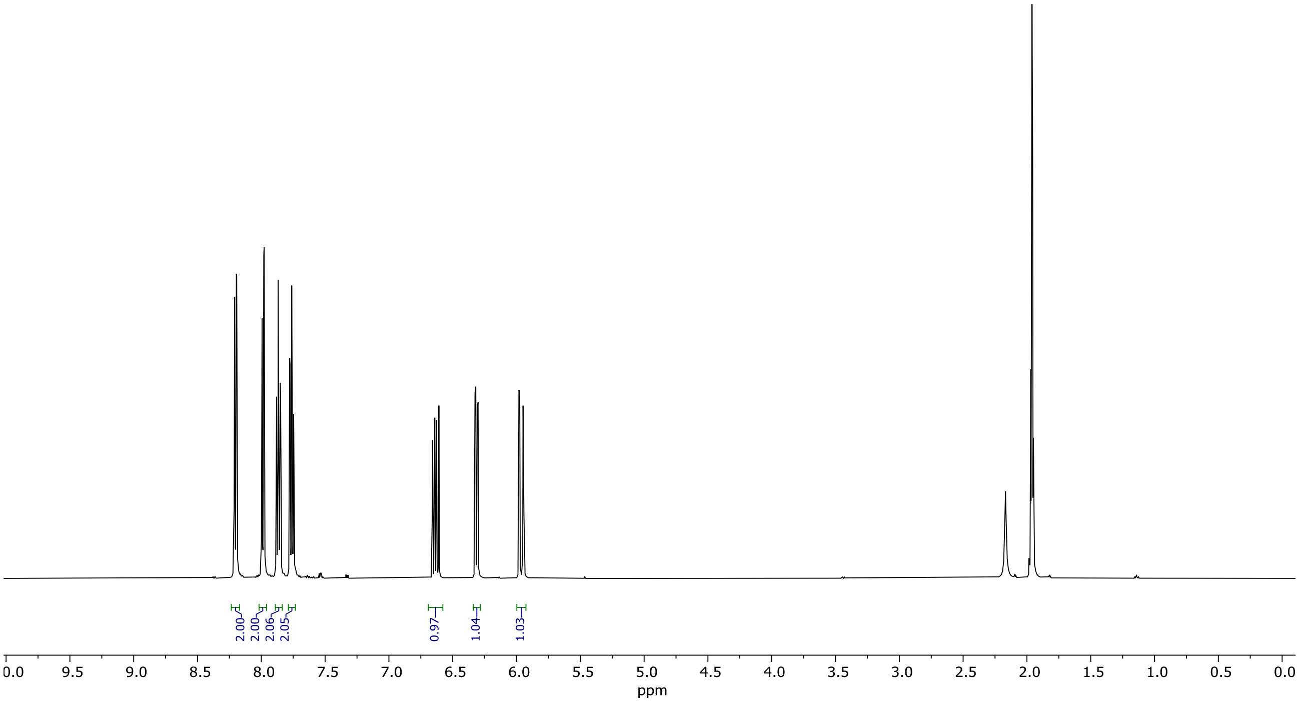

Figure S2. ^{1}H NMR spectrum of a sample of vinyl-TT^{+} (**1**) stored for one year, CD_3CN, 500 MHz, 298 K.

In addition, no decomposition was observed after heating a solid sample of **1** up to 200 °C for 5 minutes. In Figure S3 is shown the ^{1}H NMR spectrum for one sample after heating.

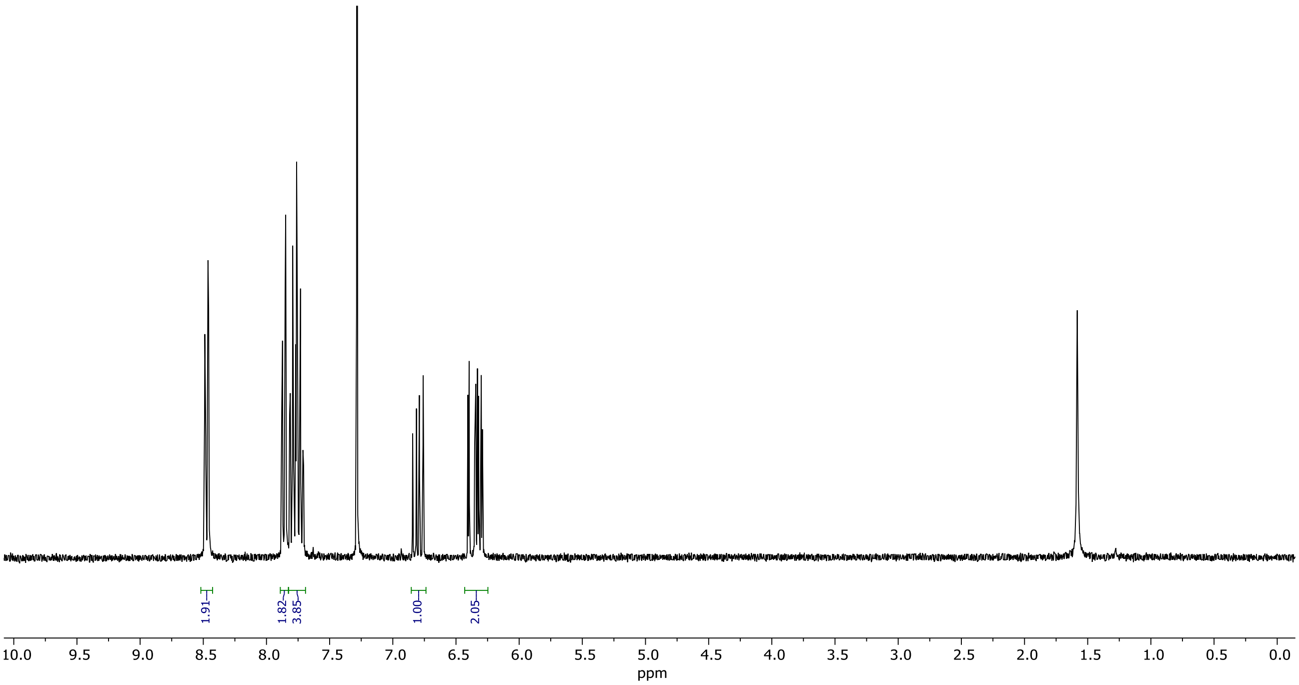

Figure S3. ^{1}H NMR spectrum of vinyl-TT^{+} (**1**) after heating up to 200 °C, $CDCl_3$, 300 MHz, 298 K.

UV-vis absorption

UV–vis absorption spectra were recorded on a UV-2600 Shimadzu spectrophotometer using a 5 × 10^{-5} M solution of vinyl-TT^+ (**1**) in MeCN.

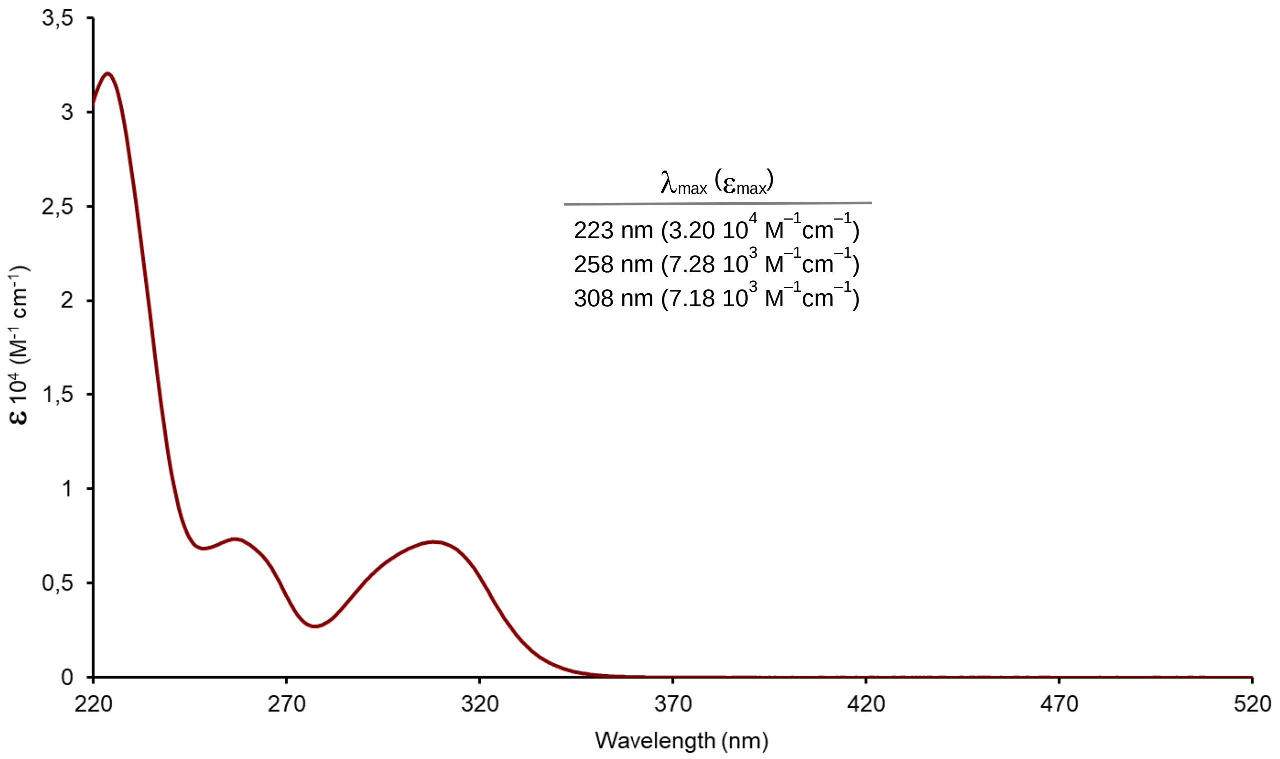

Figure S4. UV-vis absorption spectrum of **1** in solution (5 × 10^{-5} M in CH_3CN, 298 K).

Cyclic voltammetry

Cyclic voltammetry was conducted using an Autolab PGSTAT204 potentiostat equipped with a glassy carbon working electrode, a Ag/AgCl reference electrode and a Pt counter electrode. Samples containing 10mM of vinylTT and 0.1 M of tetrabutylammonium hexafluorophosphate were prepared in dry acetonitrile and degassed before measurement. Voltammograms were measured at 0.1 V/s scan rate. Potentials values were converted to SCE substracting 0.047 V according to tabulated conversions. Potentials for irreversible waves were estimated at half the maximum current ($E_{p/2}$), as previously described by Nicewicz.[8]

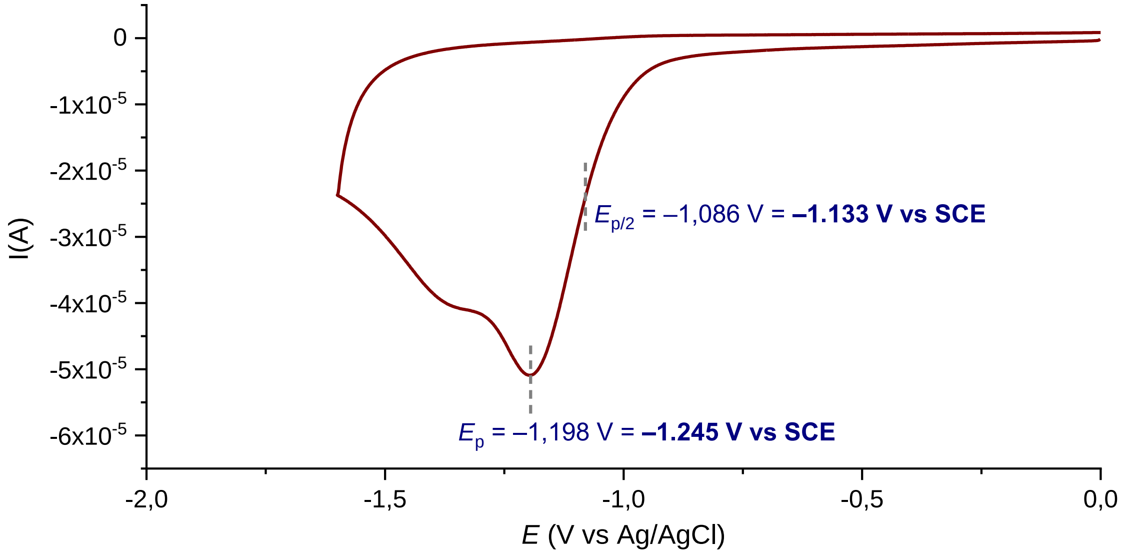

Figure S5. Cyclic voltammogram of **1**.

DSC-TGA analysis

A blank group and an experimental group were tested under Ar purge. The whole process included heating and cooling regions. The blank group and measurement groups used aluminum crucibles with pierced lids (open crucibles) as a container.

Ar gas flow velocity: 50 mL/min

Heating region: from 25 ºC to 300 ºC

Heating rate: 3 K/min

Cooling region: from 300 ºC to 25 ºC

Vinyl-$TT^+BF_4^-$(**1**, powder, 10.4566 mg, 31.6707 µmol) was placed in an Al-crucible for the DSC-TGA measurement. At 108 °C, melting of the reagent begins, which corresponds to the first endothermic peak in the DSC curve (Figure S6, bottom). Decomposition of **1** begins at around 280 °C, which is an exothermic process.

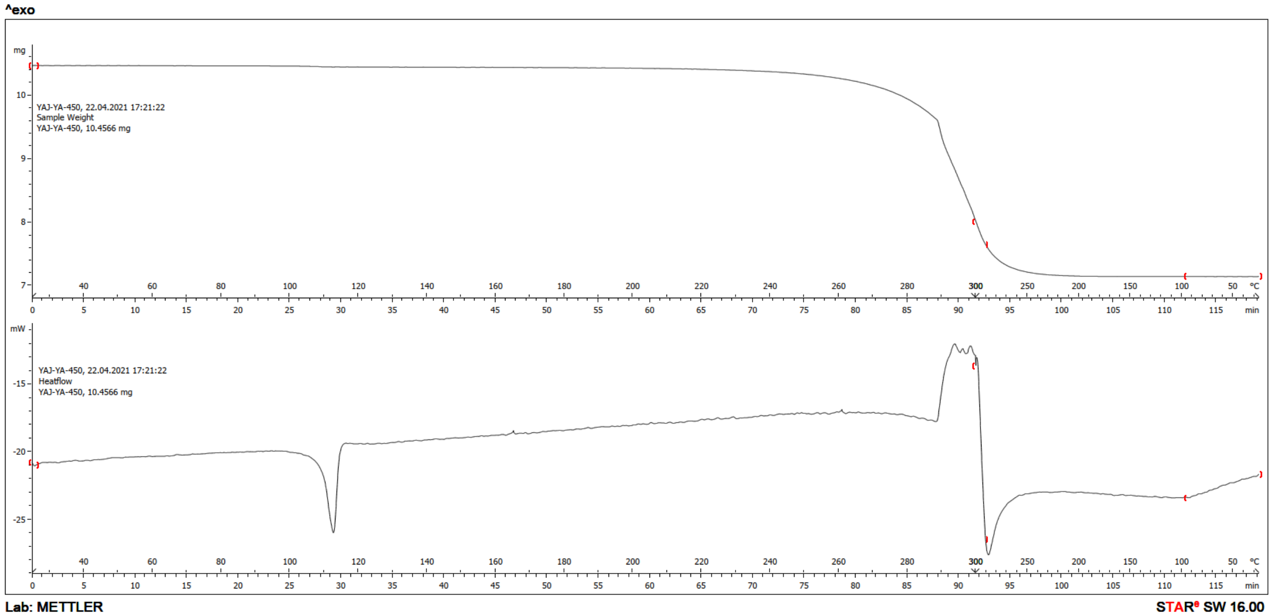

Figure S6. DSC-TGA analysis of **1**.

Preparation of 1-d_3 from ethylene-d_4

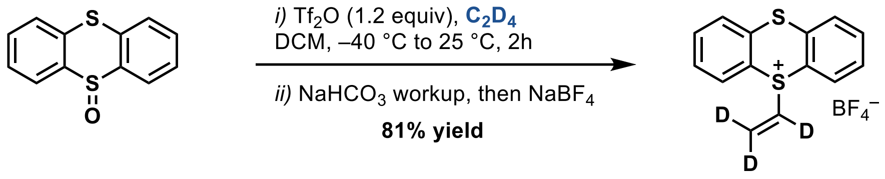

2 **vinyl-TT$^+$-d_3** (**1-d_3**)

To a 100 mL round-bottom flask equipped with a stirring bar was added thianthrene-*S*-oxide **2** (1.16 g, 5.00 mmol, 1.00 equiv.). The flask was capped with a rubber septum and was evacuated. The flask was then cooled down to –40 °C and was backfilled with ethylene-d_4. DCM (50 mL, c = 0.10 M) was added into the flask via a syringe and the solution was stirred at –40°C for 10 min. A small balloon filled with argon was then attached to the flask through a needle to balance pressure. Triflic anhydride (1.01 mL, 1.69 g, 6.00 mmol, 1.20 equiv.) was added dropwise to the reaction at –40°C, and a dark purple suspension was formed progressively. After 30 minutes, the cooling bath was removed and the mixture was stirred for 1.5 hour at 25 °C. The balloon and the rubber septum were removed, and sat. aqueous $NaHCO_3$ (30 mL) was added carefully. The mixture was poured into a separation funnel and was vigorously shaken. The phases were separated and the aqueous layer was extracted with DCM (2 × 20 mL). The combined organic phase was washed with aqueous of 5% $NaBF_4$ (4 × 60 mL), dried over $MgSO_4$, filtered and evaporated under reduced pressure. The crude material was dissolved in DCM (5 mL), and Et_2O (50 mL) was subsequently added while stirring at –40 °C, causing the precipitation of a solid that was collected by filtration, washed with Et_2O (3 × 10 mL), and dried under vacuum to afford **1-d_3** as an off-white solid (1.35 g, 4.06 mmol, 81% yield).

Deuterium incorporation: >2.99 ^{2}H/molecule (^{1}H NMR)

NMR Spectroscopy:

^{1}H NMR (500 MHz, CD_2Cl_2, 298 K, δ): 8.34 – 8.21 (m, 2H), 7.97 – 7.87 (m, 2H), 7.85 – 7.78 (m, 2H), 7.73 (ddd, *J* = 7.9, 7.4, 1.4 Hz, 2H).

^{2}H NMR (92 MHz, CD_2Cl_2, 298 K, δ): 6.74 (s, 1H), 6.35 (s, 1H), 6.22 (s, 1H).

^{13}C NMR (151 MHz, CD_2Cl_2, 298 K, δ): 136.7, 135.3, 134.5, 134.2 (m), 130.7, 130.6, 119.3 (t, *J* = 30.5 Hz), 118.6.

^{19}F NMR (470 MHz, CD_2Cl_2, 298 K, δ): –151.81 (s), –151.87 (s).

HRMS-ESI (m/z) calculated for $C_{14}H_8D_3S_2^+$ $[M–BF_4]^+$, 246.0486; found, 246.0485; deviation: –0.5 ppm.

Preparation of [4+2] cycloadduct intermediate 3

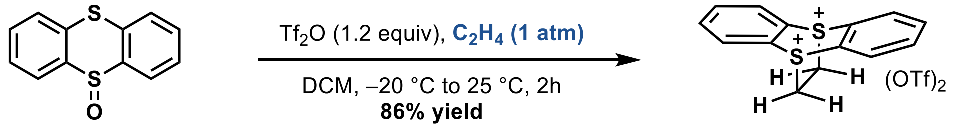

2 **3**

To a round-bottom flask equipped with a stirring bar were added thianthrene-*S*-oxide **2** (232 mg, 1.00 mmol, 1.00 equiv.) and 8.0 mL of DCM (c= 0.13 M). The flask was capped with a rubber septum and cooled down to –40°C. Through the solution was then bubbled ethylene gas for 5 minutes, after which a balloon filled with ethylene was connected to the flask to maintain the ethylene atmosphere throughout the reaction. Triflic anhydride (202 µL, 338 mg, 1.20 mmol, 1.20 equiv.) was added dropwise to the reaction and a dark purple suspension was progressively formed. After 20 minutes the cooling bath was removed, and the mixture was stirred for 1.5 hour at 25 °C. The ethylene balloon and the rubber septum were removed, and Et_2O (20 mL) was added. The resultant precipitate was collected by filtration, washed with Et_2O (3 × 5 mL), and dried under vacuum to give **3** as a colourless powder (467 mg, 0.861 mmol, 86%).

NMR Spectroscopy:

^{1}H NMR (500 MHz, CD_3CN, 298 K, δ): δ 8.56 – 8.50 (m, 4H), 8.14 – 8.09 (m, 4H), 4.18 (s, 4H).

^{13}C NMR (75 MHz, CD_3CN, 298 K, δ): 136.9, 136.4, 126.3, 37.6.

HRMS-ESI (m/z) calculated for $C_{14}H_{12}S_2^{2+}$ $[M–2OTf]^{2+}$, 122.0185; found, 122.0185; deviation: –0.4 ppm.

Reaction of other sulfoxides with ethylene

Reaction of diphenylsulfoxide with ethylene

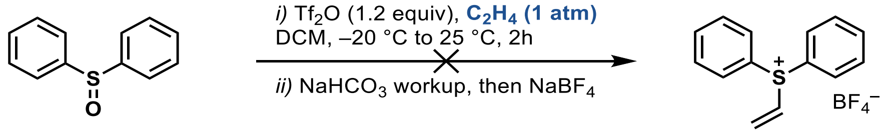

To a round-bottom flask equipped with a stirring bar were added diphenylsulfoxide (101 mg, 0.500 mmol,

1.00 equiv.) and 4 mL of DCM (c= 0.125 M). The flask was capped with a rubber septum and cooled down to –40°C. Through the solution was then bubbled ethylene gas for 5 minutes, after which a balloon filled with ethylene was connected to the flask to maintain the ethylene atmosphere throughout the reaction. Triflic anhydride (101 µL, 169 mg, 0.600 mmol, 1.20 equiv.) was added dropwise to the reaction. After 20 minutes the cooling bath was removed and the mixture was stirred for 1.5 hour at 25 °C. The ethylene balloon and the rubber septum were removed, and solution was concentrated under reduced pressure, diluted with DCM (20 mL) and washed with sat. aqueous $NaHCO_3$ (20 mL). The aqueous layer was extracted with DCM (2 × 10 mL). All organic phases were combined, washed with aqueous solutions of 5% $NaBF_4$ (2 × 10 mL), dried over $MgSO_4$, filtered, and the solvent evaporated to dryness under reduced pressure. $CDCl_3$ (1 mL) was then added and the crude was analyzed by ^1H NMR, but no vinyl-SPh_2^+ could be detected.

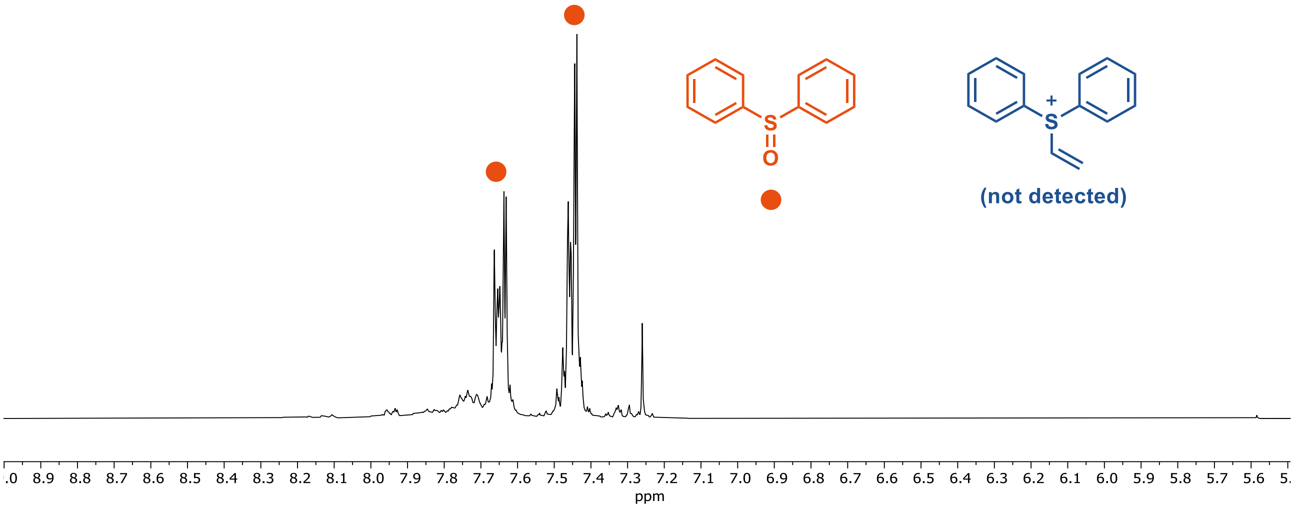

Figure S7. ^1H NMR spectrum of the crude of the reaction of diphenylsulfoxide and Tf_2O under ethylene atmosphere, $CDCl_3$, 300 MHz, 298 K.

Reaction of dibenzothiophene-S-oxide with ethylene

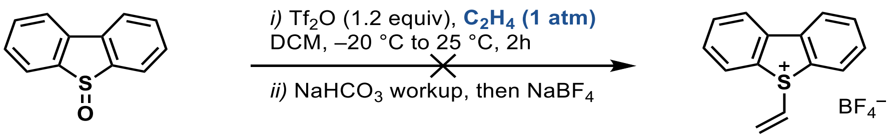

To a round-bottom flask equipped with a stirring bar were added dibenzothiophene-*S*-oxide (100 mg, 0.500 mmol, 1.00 equiv.) and 4 mL of DCM (c= 0.125 M). The flask was capped with a rubber septum and cooled down to –40°C. Through the solution was then bubbled ethylene gas for 5 minutes, after which a balloon filled with ethylene was connected to the flask to maintain the ethylene atmosphere throughout the reaction. Triflic anhydride (101 µL, 169 mg, 0.600 mmol, 1.20 equiv.) was added dropwise to the reaction. After 20 minutes the cooling bath was removed and the mixture was stirred for 1.5 hour at 25 °C. The ethylene balloon and the rubber septum were removed, and solution was concentrated under reduced pressure, diluted with DCM (20 mL) and washed with aqueous $NaHCO_3$ (20 mL). The aqueous layer was extracted with DCM (2 × 10 mL). All organic phases were combined, washed with aqueous solutions of 5%

$NaBF_4$ (2 × 10 mL), dried over $MgSO_4$, filtered, and the solvent evaporated to dryness under reduced pressure. $CDCl_3$ (1 mL) was then added and the crude was analyzed by ^{1}H NMR, but no *S*-vinyl-sulfonium salt could be detected.

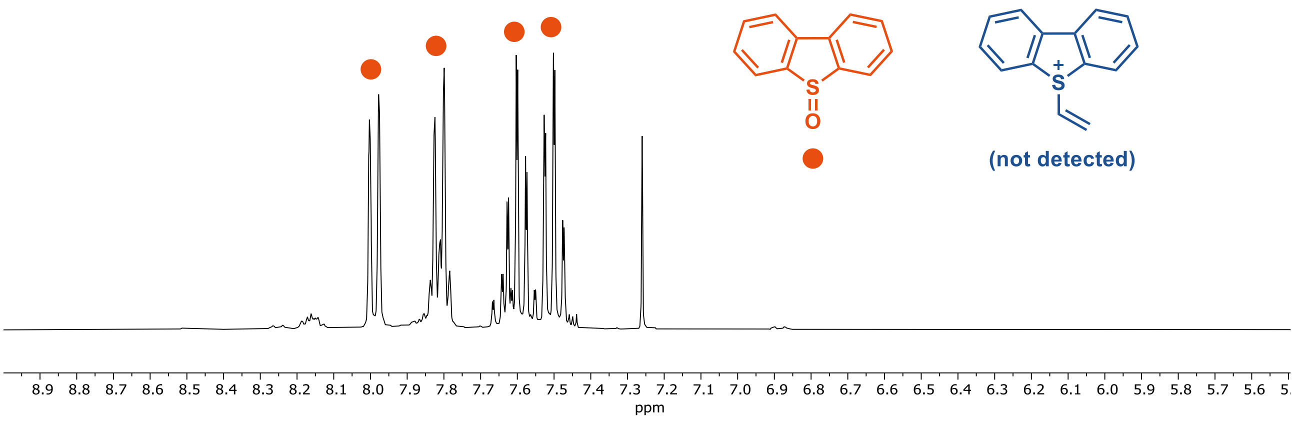

Figure S8. ^{1}H NMR spectrum of the crude of the reaction of dibenzothiophene-*S*-oxide and Tf_2O under ethylene atmosphere, $CDCl_3$, 300 MHz, 298 K.

Preparation of vinyl-SPh_2(OTf) (S3)

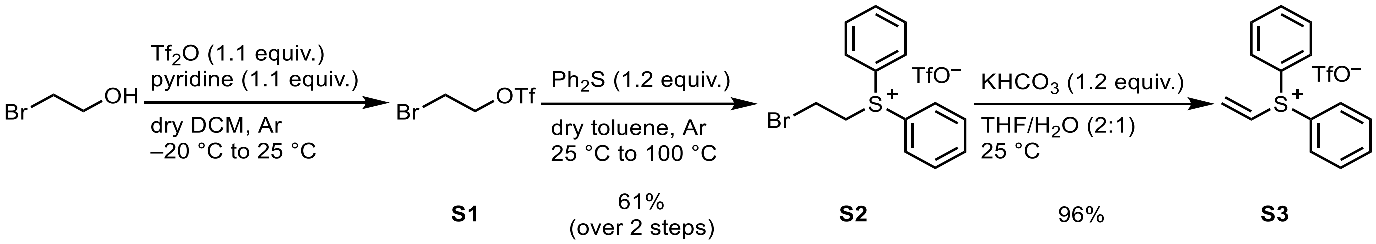

This compound was prepared following the 3-step procedure reported by Aggarwal:[2]

To an oven-dried 100 mL round-bottom flask under argon atmosphere containing a teflon-coated magnetic stirring bar were added pyridine (1.7 g, 1.7 mL, 22 mmol, 1.1 equiv.) and anhydrous DCM (35 mL) and the mixture was cooled down to –20 °C. Trifluoromethanesulfonic anhydride (5.9 g, 3.5 mL, 21 mmol, 1.1 equiv.) was added dropwise and the reaction mixture was allowed to stir for 10 minutes at the same temperature. 2-Bromoethanol (2.5 g, 1.4 mL, 20 mmol, 1.0 equiv.) was then added dropwise to the reaction mixture under –20 °C. The cooling bath was removed and the reaction stirred for a further 10 minutes (do not allow more than this time) while warming. The resulting suspension was filtered, concentrated (using a rotary evaporator, keeping the water bath temp below 20 °C) and pentane (30 mL) was added. The mixture was filtered and the filtrate was concentrated again under reduced pressure and dried under vacuum to give the title product **S1** as a clear colorless oil (4.6 g, 89%). It was used immediately in the next step without further purification.

To a round-bottom flask under argon atmosphere containing a teflon-coated magnetic stirring bar were added **S1** (4.58 g, 17.8 mmol, 1.00 equiv.), anhydrous toluene (20 mL) and diphenyl sulfide (4.0 g, 3.6 mL, 15 mmol,

1.2 equiv.) at 25 °C. The reaction mixture was then heated at 100 °C under argon for 5 h. The solution was allowed to cool to 25 °C and diethyl ether (20 mL) was added. The resulting mixture was filtered and the residue was washed with diethyl ether (10 mL) to afford 5.4 g of the title compound **S2** (69% yield) as a white power.

Under ambient atmosphere, a 20 mL vail equipped with a teflon-coated magnetic stirring bar was charged with **S2** (443 mg, 1.00 mmol, 1.00 equiv.) and THF/H_2O (2:1) (3 mL). $KHCO_3$ (120 mg, 1.20 mmol, 1.20 equiv.) was added in one portion and the reaction mixture was stirred for 30 min at 25 °C (do not allow more than this time). Water (1 mL) was added and the mixture was extracted with DCM (3 × 5 mL). The organic layers were collected, dried over anhydrous Na_2SO_4, filtered and concentrated under reduced pressure. The residue was purified by column chromatography on silica gel eluting with a solvent mixture of methonal:DCM (1:15 (v:v)) to afford 348 mg of the title compound (S3) as a yellow oil (96% yield). The NMR spectra are in accordance with the literature.[2]

Annulation reactions employing vinyl-TT$^+$ 1

Spiro[cyclopropane-1,3'-indolin]-2'-one (5)

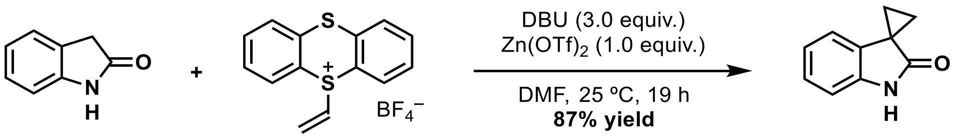

1 (1.2 equiv.) **5**

Following a modified reported procedure,[9] 2-oxindole (26.6 mg, 0.200 mmol, 1.00 equiv.), **1** (79.2 mg, 0.240 mmol, 1.20 equiv.), and zinc triflate (72.7 mg, 0.200 mmol, 1.00 equiv.) were dissolved in DMF (1.0 mL, *c*= 0.20 M) under ambient atmosphere. To this solution was added DBU (90 µL, 92 mg, 0.60 mmol, 3.0 equiv.). After stirring for 19 h at 25 °C, a saturated aqueous solution of NH_4Cl (7 mL) was added, and the phases were separated. The aqueous phase was extracted with EtOAc (3 × 50 mL). All organic phases were combined, washed with water (2 × 10 mL), dried over Na_2SO_4, filtered, and concentrated under reduced pressure. Purification of the residue by column chromatography on silica gel eluting with hexanes/EtOAc (3:1, v/v) afforded the title compound as a pale-yellow solid (27.6 mg, 0.173 mmol, 87%). The NMR spectra are in good accordance with the literature.[9]

R_f = 0.40 (hexanes/EtOAc, 1:1).

NMR Spectroscopy:

^{1}H NMR (500 MHz, $CDCl_3$, 298 K, δ): 9.43 (s, 1H), 7.22–7.16 (m, 1H), 7.04–6.97 (m, 2H), 6.83 (m, 1H), 1.81–1.74 (m, 2H), 1.58–1.51 (m, 2H).

^{13}C NMR (126 MHz, $CDCl_3$, 298 K, δ): 180.0, 141.0, 131.4, 126.9, 122.1, 118.7, 110.1, 27.7, 19.6.

HRMS-EI (m/z) calculated for $C_{10}H_9NO^+$ [M]$^+$, 159.0679; found, 159.0680; deviation: –1.0 ppm.

Methyl (±)-4-tosylmorpholine-3-carboxylate (7)

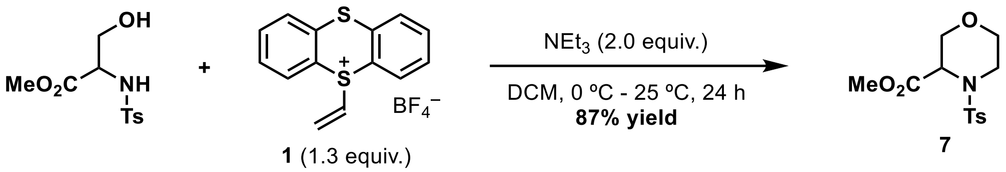

Following a modified reported procedure,[2] *N*-tosyl DL-serine methyl ester (54.7 mg, 0.200 mmol, 1.00 equiv.) was dissolved in dry DCM (1.0 mL, *c*= 0.20 M) under an argon atmosphere, and the resulting solution was cooled to 0 °C. To the solution was added dry NEt_3 (56 μL, 41 mg, 0.40 mmol, 2.0 equiv.), and after 10 min a solution of **1** (87.4 mg, 0.265 mmol, 1.32 equiv.) in dry DCM (0.5 mL) was added dropwise. After stirring for 3 h at 0 °C, and then for 21 h at 25 °C, a saturated aqueous solution of NH_4Cl (3 mL) was added. The phases were separated, and the aqueous phase was extracted with DCM (3 × 20 mL). All organic phases were combined, washed with brine (20 mL), dried over $MgSO_4$, filtered, and concentrated under reduced pressure. Purification of the residue by column chromatography on silica gel eluting with hexanes/EtOAc (3:1 to 1:1, v/v) afforded the title compound as a colorless solid (52.2 mg, 0.174 mmol, 87%). The NMR spectra are in good accordance with the literature.[2]

R_f = 0.54 (hexanes/EtOAc).

NMR Spectroscopy:

^{1}H NMR (500 MHz, $CDCl_3$, 298 K, δ): 7.65 (d, *J* = 8.3 Hz, 2H), 7.29 (d, *J* = 8.3 Hz, 2H), 4.50 (d, *J* = 3.3 Hz, 1H), 4.26 (d, *J* = 11.6 Hz, 1H), 3.87–3.82 (m, 1H), 3.69 (dd, *J* = 11.6, 3.7 Hz, 1H), 3.57–3.44 (m, 6H), 2.42 (s, 3H).

^{13}C NMR (126 MHz, $CDCl_3$, 298 K, δ): 169.6, 143.7, 136.5, 129.7, 127.5, 68.9, 66.8, 55.5, 52.4, 42.0, 21.7.

HRMS-CI (m/z) calculated for $C_{13}H_{18}NO_5S^+$ [M+H]$^+$, 300.0900; found, 300.0901; deviation: –0.2 ppm.

Ethyl (±)-2-phenyl-1-tosylazetidine-2-carboxylate (9)

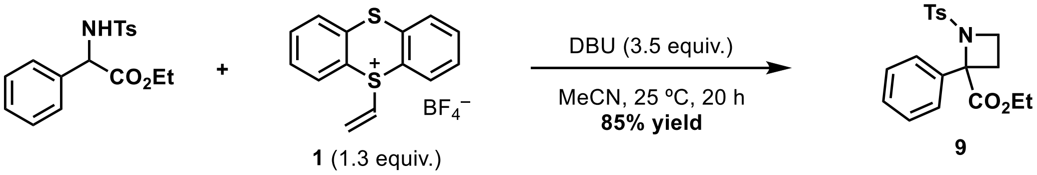

Following a modified reported procedure,[10] a vial was charged with *N*-tosyl DL-phenylglycine ethyl ester (55.7 mg, 0.167 mmol, 1.00 equiv.), **1** (69.3 mg, 0.210 mmol, 1.26 equiv.), and MeCN (2.3 mL, *c*= 0.073 M) at 25 °C under air. Then, DBU (87 μL, 89 mg, 0.58 mmol, 3.5 equiv.) was added. The reaction was stirred for 20 h at 25 °C and was then concentrated under reduced pressure. The residue was purified by column chromatography on silica gel eluting with pentane/EtOAc (100% pentane to 5:1, v/v). The title compound (51.2 mg, 0.142 mmol, 85%) was obtained as a colorless oil. The NMR spectra are in good accordance with

the literature.[10]

R*f* = 0.21 (pentane/EtOAc, 5:1).

NMR Spectroscopy:

^{1}H NMR (500 MHz, $CDCl_3$, 298 K, δ): 7.58 (d, *J* = 8.3 Hz, 2H), 7.45–7.40 (m, 2H), 7.36–7.28 (m, 3H), 7.23 (d, *J* = 8.3 Hz, 2H), 4.27–4.16 (m, 3H), 3.82 (ddd, *J* = 9.1, 6.7, 4.9 Hz, 1H), 2.90 (ddd, *J* = 11.3, 9.2, 4.9 Hz, 1H), 2.57 (ddd, *J* = 11.2, 9.1, 7.0 Hz, 1H), 2.41 (s, 3H), 1.27 (t, *J* = 7.1 Hz, 3H).

^{13}C NMR (126 MHz, $CDCl_3$, 298 K, δ): 170.8, 143.4, 139.3, 136.9, 129.5, 128.4, 128.1, 127.5, 126.4, 77.4, 62.2, 47.5, 29.3, 21.6, 14.0.

HRMS-ESI (m/z) calculated for $C_{19}H_{21}NO_4SNa^+$ $[M+Na]^+$, 382.1084; found, 382.1086; deviation: –0.5 ppm.

(±)-3-Tosyl-7-oxa-3-azabicyclo[4.1.0]heptane (11)

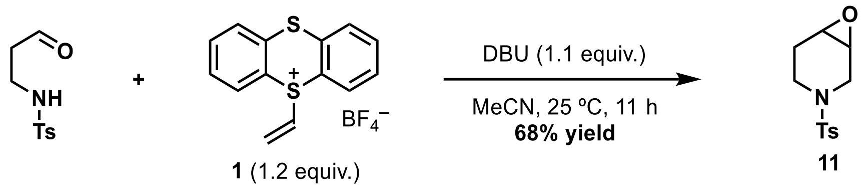

Epoxide **11** was prepared following a modified reported procedure.[11] Under air, DBU (14 μL, 14 mg, 0.094 mmol, 1.1 equiv.) was added to a mixture of 4-methyl-*N*-(3-oxopropyl)benzenesulfonamide[4] (19.0 mg, 0.0836 mmol, 1.00 equiv.) (used immediately after preparation) and **1** (33.1 mg, 0.100 mmol, 1.20 equiv.) in MeCN (1.0 mL, *c*= 0.084 M). The reaction mixture was stirred at 25 °C for 11 h and was then concentrated under reduced pressure. Purification of the residue by column chromatography on silica gel eluting with hexanes/EtOAc (3:1, v/v) afforded the title compound as a colorless solid (14.3 mg, 0.0565 mmol, 68%). The NMR spectra are in good accordance with the literature.[11]

R*f* = 0.48 (hexanes/EtOAc, 1:1).

NMR Spectroscopy:

^{1}H NMR (500 MHz, $CDCl_3$, 298 K, δ): 7.62 (d, *J* = 8.2 Hz, 2H), 7.32 (d, *J* = 8.2 Hz, 2H), 3.85 (ddd, *J* = 13.7, 4.3, 1.5 Hz, 1H), 3.41–3.33 (m, 1H), 3.30–3.24 (m, 2H), 3.09 (d, *J* = 13.7 Hz, 1H), 2.56 (ddd, *J* = 12.1, 8.6, 6.0 Hz, 1H), 2.43 (s, 3H), 2.13–2.08 (m, 2H).

^{13}C NMR (126 MHz, $CDCl_3$, 298 K, δ): 143.9, 133.4, 129.9, 127.7, 50.5, 49.9, 44.3, 39.3, 25.4, 21.7.

HRMS-CI (m/z) calculated for $C_{12}H_{16}N_1O_3S_1^+$ $[M+H]^+$, 254.0845; found, 254.0844; deviation: 0.4 ppm.

Vinylation of *N*-heterocycles using vinyl-TT$^+$

General procedure A

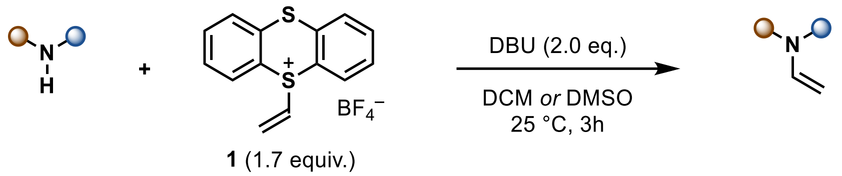

Under air, a vial was charged with the substrate to be vinylated (0.300 mmol, 1.00 equiv.) and **1** (168 mg, 0.510 mmol, 1.70 equiv.). DCM or DMSO (3.0 mL, *c*= 0.10 M) was added, followed by DBU (90 µL, 92 mg, 0.60 mmol, 2.0 equiv.), and the mixture was stirred at 25 °C for 3 h. Then, the solvent was removed under reduced pressure, and the residue was purified as indicated to give the corresponding product**. *Important note:*** *some N-vinylated product were observed to decompose by polymerization during chromatography. To prevent this 1% NEt_3 was included in the eluent solvent system to deactivate silica.*

9-Vinyl-9*H*-pyrido[3,4-*b*]indole (12)

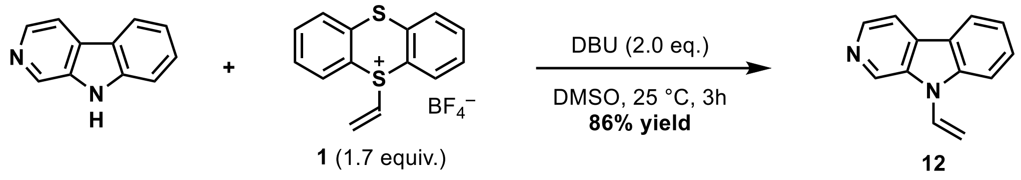

The title compound was prepared following general procedure A. Under air, a vial was charged with norharmane (50.5 mg, 0.300 mmol, 1.00 equiv.) and **1** (119 mg, 0.360 mmol, 1.20 equiv.). DMSO (3.0 mL, *c*= 0.10 M) was added, followed by DBU (90 µL, 92 mg, 0.60 mmol, 2.0 equiv.), and the mixture was stirred at 25 °C for 3 h. Then, the solvent was removed under reduced pressure. Purification by column chromatography on silica gel eluting with hexanes/EtOAc (100% hexanes to 1:1, containing 1% of NEt_3) yielded 9-vinyl-9*H*-pyrido[3,4-*b*]indole (**12**) as a yellow oil (50.0 mg, 0.257 mmol, 86%).

R_f = 0.22 (hexanes/EtOAc, 1:1).

NMR Spectroscopy:

^{1}H NMR (600 MHz, DMSO-d_6, 298 K, δ): 9.27 (d, *J* = 0.8 Hz, 1H), 8.50 (d, *J* = 5.1 Hz, 1H), 8.35–8.30 (m, 1H), 8.21 (dd, *J* = 5.1, 1.0 Hz, 1H), 7.95–7.92 (m, 1H), 7.71–7.65 (m, 2H), 7.39 (ddd, *J* = 7.9, 7.2, 0.9 Hz, 1H), 5.73 (dd, *J* = 15.9, 1.5 Hz, 1H), 5.22 (dd, *J* = 9.3, 1.5 Hz, 1H).

^{13}C NMR (151 MHz, DMSO-d_6, 298 K, δ): 140.5, 139.4, 134.9, 133.9, 129.4, 129.2, 129.0, 122.0, 121.4, 121.4, 114.7, 111.6, 102.5.

HRMS-EI (m/z) calculated for $C_{13}H_{10}N_2^+$ [M]$^+$, 194.0838; found, 194.0839; deviation: –0.5 ppm.

5-Nitro-1-vinyl-1*H*-indole (13)

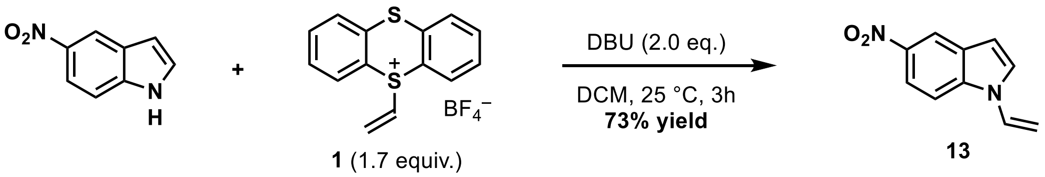

The title compound was prepared following general procedure A. Under air, a vial was charged with 5-nitroindole (48.6 mg, 0.300 mmol, 1.00 equiv.) and **1** (168 mg, 0.510 mmol, 1.70 equiv.). DCM (3.0 mL, *c*= 0.10 M) was added, followed by DBU (90 µL, 92 mg, 0.60 mmol, 2.0 equiv.), and the mixture was stirred at 25 °C for 3 h. Then, the solvent was removed under reduced pressure. Purification by column chromatography on silica gel eluting with hexanes/EtOAc (100% hexanes to 10:1, containing 1% of NEt_3) yielded 5-nitro-1-vinyl-1*H*-indole (**13**) as a yellow solid (41.3 mg, 0.219 mmol, 73%).

R_f = 0.37 (hexanes/EtOAc, 4:1).

NMR Spectroscopy:

^{1}H NMR (500 MHz, $CDCl_3$, 298 K, δ): 8.57 (d, *J* = 2.1 Hz, 1H), 8.16 (dd, *J* = 9.1, 2.2 Hz, 1H), 7.58 (d, *J* = 3.5 Hz, 1H), 7.48 (d, *J* = 9.1 Hz, 1H), 7.23 (dd, *J* = 15.7, 8.9 Hz, 1H), 6.80 (d, *J* = 3.5 Hz, 1H), 5.34 (dd, *J* = 15.7, 1.8 Hz, 1H), 4.98 (dd, *J* = 8.9, 1.8 Hz, 1H).

^{13}C NMR (75 MHz, $CDCl_3$, 298 K, δ): 142.6, 138.1, 129.1, 128.5, 126.7, 118.3, 118.2, 109.6, 106.8, 100.2.

HRMS-ESI (m/z) calculated for $C_{10}H_8N_2O_2Na^+$ $[M+Na]^+$, 211.0478; found, 211.0478; deviation: 0.1 ppm.

1-Vinyl-1*H*-indole-3-carbaldehyde (14)

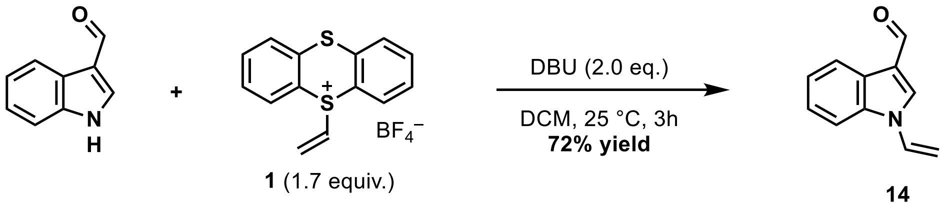

The title compound was prepared following general procedure A. Under air, a vial was charged with 1*H*-indole-3-carbaldehyde (43.5 mg, 0.300 mmol, 1.00 equiv.) and **1** (168 mg, 0.510 mmol, 1.70 equiv.). DCM (3.0 mL, *c*= 0.10 M) was added, followed by DBU (90 µL, 92 mg, 0.60 mmol, 2.0 equiv.), and the mixture was stirred at 25 °C for 3 h. Then, the solvent was removed under reduced pressure. Purification by column chromatography on silica gel eluting with DCM/EtOAc (100% DCM to 4:1, containing 1% of NEt_3) yielded 1-vinyl-1*H*-indole-3-carbaldehyde (**14**) as a pale-yellow oil (37.2 mg, 0.217 mmol, 72%).

R_f = 0.20 (DCM).

NMR Spectroscopy:

^{1}**H NMR** (500 MHz, $CDCl_3$, 298 K, δ): 10.09 (s, 1H), 8.34–8.29 (m, 1H), 8.00 (s, 1H), 7.51–7.48 (m, 1H), 7.41–7.33 (m, 2H), 7.22 (dd, *J* = 15.7, 8.8 Hz, 1H), 5.47 (dd, *J* = 15.7, 1.9 Hz, 1H), 5.09 (dd, *J* = 8.8, 1.8 Hz, 1H).

^{13}**C NMR** (75 MHz, $CDCl_3$, 298 K, δ): 185.1, 136.7, 133.7, 129.2, 125.5, 124.9, 123.8, 122.4, 120.2, 110.1, 102.3.

HRMS-EI (m/z) calculated for $C_{11}H_9NO^+$ $[M]^+$, 171.0679; found, 171.0679; deviation: –0.1 ppm.

Ethyl 3-amino-1-vinyl-1H-pyrazole-4-carboxylate (15-I) and ethyl 5-amino-1-vinyl-1H-pyrazole-4-carboxylate (15-II)

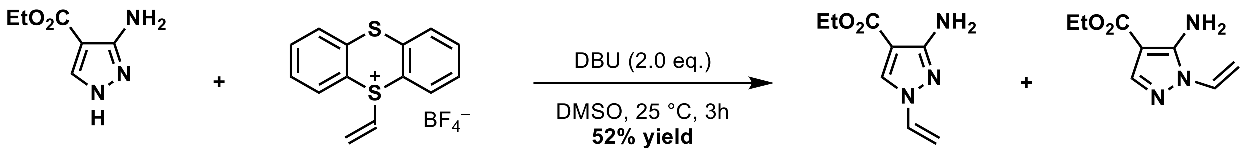

1 (1.2 equiv.) **15-I** (26%) **15-II** (26%)

Under air, a vial was charged with ethyl 3-amino-1H-pyrazole-4-carboxylate (46.5 mg, 0.300 mmol, 1.00 equiv.) and DMSO (2.0 mL). DBU (90 μL, 92 mg, 0.60 mmol, 2.0 equiv.) was added, and the mixture was stirred for 5 min at 25 °C. Then, a solution of **1** (119 mg, 0.360 mmol, 1.20 equiv.) in DMSO (1.0 mL) was added, and the resulting solution was stirred at at 25 °C for 3 hours. The mixture was then diluted with DCM (20 mL) and washed with H_2O (20 mL). The aqueous phase was extracted with DCM (2 × 10 mL) and the combined organic phases were washed with brine (20 mL), dried over MgSO4 and the solvent removed under reduced pressure. Purification by column chromatography silica gel eluting with hexanes/EtOAc (8:1 to 3:1, containing 1% of NEt_3) yielded ethyl 3-amino-1-vinyl-1H-pyrazole-4-carboxylate (**15-I**) (14.0 mg, 0.077 mmol, 26%) and ethyl 5-amino-1-vinyl-1H-pyrazole-4-carboxylate (**15-II**) (14.0 mg, 0.077 mmol, 26%), both as a colorless solids.

Data for 15-I:

R_f = 0.36 (hexanes/EtOAc, 3:1).

NMR Spectroscopy:

^{1}**H NMR** (500 MHz, CD_2Cl_2, 298 K, δ): δ 7.71 (s, 1H), 6.78 (dd, *J* = 15.4, 8.7 Hz, 1H), 5.48 (dd, *J* = 15.4, 1.0 Hz, 1H), 4.81 (br s, 2H), 4.79 (dd, *J* = 8.7, 1.0 Hz, 3H), 4.26 (q, *J* = 7.1 Hz, 2H), 1.32 (t, *J* = 7.2 Hz, 3H).

^{13}**C NMR** (75 MHz, CD_2Cl_2, 298 K, δ): 164.2, 157.1, 132.3, 131.9, 101.5, 100.4, 60.4, 14.6.

HRMS-ESI (m/z) calculated for $C_8H_{12}N_3O_2^+$ $[M+H]^+$, 182.0924; found, 182.0927; deviation: –1.5 ppm.

Data for 15-II:

R_f = 0.29 (hexanes/EtOAc, 3:1).

NMR Spectroscopy:

^{1}**H NMR** (500 MHz, CD_2Cl_2, 298 K, δ): 7.66 (s, 1H), 6.80 (ddd, *J* = 15.2, 8.9, 0.8 Hz, 1H), 5.60 (dd, *J* = 15.4, 0.7 Hz, 1H), 5.26 (br s, 2H), 4.94 (dd, *J* = 8.9, 0.7 Hz, 1H), 4.25 (q, *J* = 7.1 Hz, 2H), 1.32 (t, *J* = 7.1 Hz, 3H).

^{13}**C NMR** (151 MHz, CD_2Cl_2, 298 K, δ): 164.6, 149.0, 141.1, 128.0, 102.2, 96.8, 60.1, 14.7.

HRMS-EI (m/z) calculated for $C_8H_{11}N_3O_2^+$ $[M]^+$, 181.0846; found, 181.0848; deviation: –1.2 ppm.

4-Bromo-3,5-dimethyl-1-vinyl-1*H*-pyrazole (16)

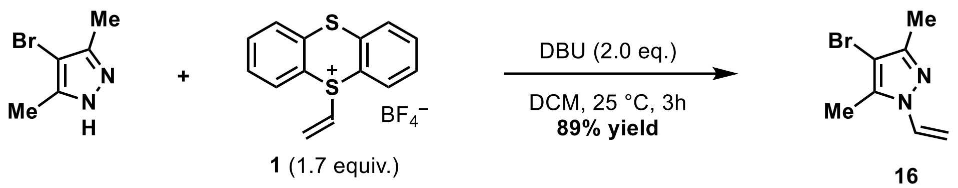

16

The title compound was prepared following general procedure A. Under air, a vial was charged with 4-bromo-3,5-dimethyl-1*H*-pyrazole (52.5 mg, 0.300 mmol, 1.00 equiv.) and **1** (168 mg, 0.510 mmol, 1.70 equiv.). DCM (3.0 mL, *c*= 0.10 M) was added, followed by DBU (90 µL, 92 mg, 0.60 mmol, 2.0 equiv.), and the mixture was stirred at 25 °C for 3 h. Then, the solvent was removed under reduced pressure. Purification by column chromatography on silica gel eluting with DCM (containing 1% of NEt_3) yielded 4-bromo-3,5-dimethyl-1-vinyl-1*H*-pyrazole (**16**) as a colorless oil (53.5 mg, 0.266 mmol, 89%).

$\mathbf{R}_f$ = 0.30 (DCM).

NMR Spectroscopy:

^{1}**H NMR** (500 MHz, $CDCl_3$, 298 K, δ): 6.87 (dd, *J* = 15.3, 8.9 Hz, 1H), 5.62 (d, *J* = 15.3 Hz, 1H), 4.81 (d, *J* = 8.9 Hz, 1H), 2.28 (s, 3H), 2.25 (s, 3H).

^{13}**C NMR** (75 MHz, $CDCl_3$, 298 K, δ): 148.4, 137.1, 129.6, 100.7, 96.4, 12.6, 10.4.

HRMS-EI (m/z) calculated for $C_7H_9N_2Br_1^+$ $[M]^+$, 199.9944; found, 199.9945; deviation: –0.8 ppm.

Methyl 1-vinyl-1*H*-1,2,4-triazole-3-carboxylate (17)

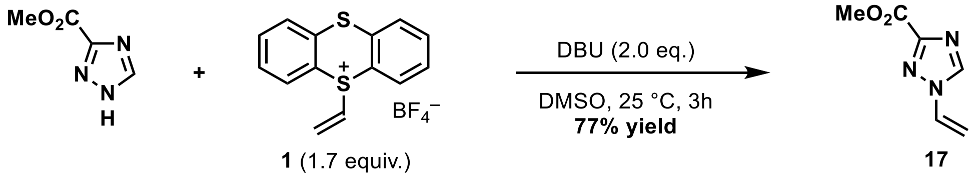

17

The title compound was prepared following general procedure A. Under air, a vial was charged with methyl 4*H*-1,2,4-triazole-3-carboxylate (38.1 mg, 0.300 mmol, 1.00 equiv.) and **1** (168 mg, 0.510 mmol, 1.70 equiv.). DMSO (3.0 mL, *c*= 0.10 M) was added, followed by DBU (90 µL, 92 mg, 0.60 mmol, 2.0 equiv.), and the mixture was stirred at 25 °C for 3 h. Then, the solvent was removed under reduced pressure. Purification by column chromatography on silica gel eluting with DCM/EtOAc (100% DCM to 2:1, containing 1% of NEt_3)

yielded methyl 1-vinyl-1*H*-1,2,4-triazole-3-carboxylate (**17**) as an off-white solid (35.3 mg, 0.231 mmol, 77%).

R*f* = 0.27 (DCM/EtOAc, 2:1).

NMR Spectroscopy:

^{1}H NMR (500 MHz, $CDCl_3$, 298 K, δ): 8.36 (s, 1H), 7.11 (dd, *J* = 15.6, 8.8 Hz, 1H), 5.92 (dd, *J* = 15.6, 1.9 Hz, 1H), 5.22 (dd, *J* = 8.8, 1.8 Hz, 1H), 3.99 (s, 3H).

^{13}C NMR (126 MHz, $CDCl_3$, 298 K, δ): 160.0, 155.1, 143.1, 129.1, 107.1, 53.0.

HRMS-ESI (m/z) calculated for $C_6H_7N_3O_2Na^+$ [M+Na]$^+$, 176.0431; found, 176.0431; deviation: –0.2 ppm.

1-Vinylpyridin-4(1*H*)-one (18)

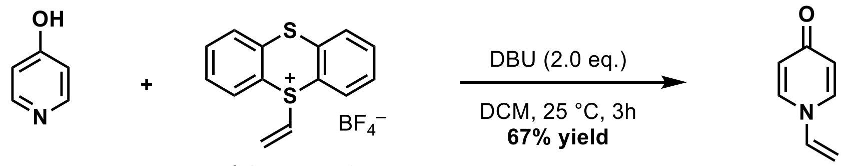

1 (1.7 equiv.)

18

The title compound was prepared following general procedure A. Under air, a vial was charged with 4-hydroxypyridine (28.5 mg, 0.300 mmol, 1.00 equiv.) and **1** (168 mg, 0.510 mmol, 1.70 equiv.). DCM (3.0 mL, *c*= 0.10 M) was added, followed by DBU (90 µL, 92 mg, 0.60 mmol, 2.0 equiv.), and the mixture was stirred at 25 °C for 3 h. Then, the solvent was removed under reduced pressure. Purification by column chromatography on silica gel eluting with DCM/MeOH (50:1 to 20:1, containing 1% of NEt_3) yielded 1-vinylpyridin-4(1*H*)-one (**18**) as a colorless solid (24.3 mg, 0.201 mmol, 67%).

R*f* = 0.08 (DCM/MeOH, 20:1).

NMR Spectroscopy:

^{1}H NMR (600 MHz, $CDCl_3$, 298 K, δ): 7.52–7.49 (m, 2H), 6.63 (dd, *J* = 15.5, 8.8 Hz, 1H), 6.43–6.39 (m, 2H), 5.20 (dd, *J* = 15.6, 2.9 Hz, 1H), 4.96 (dd, *J* = 8.8, 2.9 Hz, 1H).

^{13}C NMR (151 MHz, $CDCl_3$, 298 K, δ): 179.7, 136.3, 135.6, 119.0, 101.7.

HRMS-EI (m/z) calculated for $C_7H_7N_1O_1^+$ [M]$^+$, 121.0522; found, 121.0523; deviation: –0.6 ppm.

4-(4-Fluorophenyl)-1-vinyl-1*H*-imidazole (19)

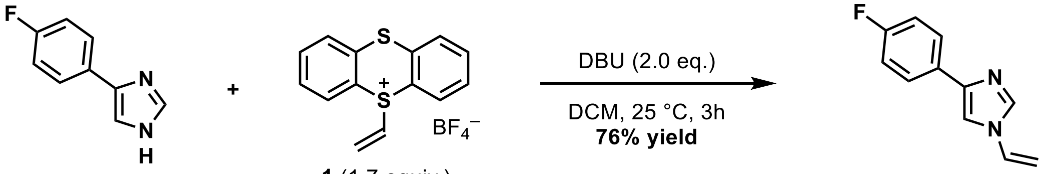

1 (1.7 equiv.)

19

The title compound was prepared following general procedure A. Under air, a vial was charged with 4-(4-

fluorophenyl)-1*H*-imidazole (48.6 mg, 0.300 mmol, 1.00 equiv.) and **1** (168 mg, 0.510 mmol, 1.70 equiv.). DCM (3.0 mL, *c*= 0.10 M) was added, followed by DBU (90 µL, 92 mg, 0.60 mmol, 2.0 equiv.), and the mixture was stirred at 25 °C for 3 h. Then, the solvent was removed under reduced pressure. Purification by column chromatography on silica gel eluting with hexanes/EtOAc (100% hexanes to 1:1, containing 1% of NEt_3) yielded 4-(4-fluorophenyl)-1-vinyl-1*H*-imidazole (**19**) as a colorless solid (43.0 mg, 0.228 mmol, 76%).

R*f* = 0.27 (hexanes/EtOAc, 1:1).

NMR Spectroscopy:

^{1}**H NMR** (600 MHz, CD_2Cl_2, 298 K, δ): 7.79–7.75 (m, 2H), 7.65 (d, *J* = 1.3 Hz, 1H), 7.47 (d, *J* = 1.3 Hz, 1H), 7.10–7.06 (m, 2H), 6.94 (dd, *J* = 15.7, 8.8 Hz, 1H), 5.34 (dd, *J* = 15.7, 1.8 Hz, 1H), 4.93 (dd, *J* = 8.8, 1.8 Hz, 1H).

^{13}**C NMR** (151 MHz, CD_2Cl_2, 298 K, δ): 162.5 (d, *J* = 245.0 Hz), 142.3, 137.0, 130.5 (d, *J* = 3.1 Hz), 129.7, 127.0 (d, *J* = 7.9 Hz), 115.8 (d, *J* = 21.6 Hz), 111.1, 101.6.

^{19}**F NMR** (282 MHz, CD_2Cl_2, 298 K, δ): –116.2 (s).

HRMS-EI (m/z) calculated for $C_{11}H_9N_2F^+$ $[M]^+$, 188.0744; found, 188.0746; deviation: –0.9 ppm.

4-Chloro-7-vinyl-7*H*-pyrrolo[2,3-*d*]pyrimidine (20)

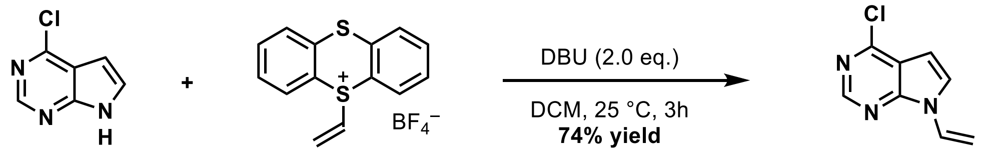

1 (1.7 equiv.) **20**

The title compound was prepared following general procedure A. Under air, a vial was charged with 4-chloro-7*H*-pyrrolo[2,3-*d*]pyrimidine (46.1 mg, 0.300 mmol, 1.00 equiv.) and **1** (168 mg, 0.510 mmol, 1.70 equiv.). DCM (3.0 mL, *c*= 0.10 M) was added, followed by DBU (90 µL, 92 mg, 0.60 mmol, 2.0 equiv.), and the mixture was stirred at 25 °C for 3 h. Then, the solvent was removed under reduced pressure. Purification by column chromatography on silica gel eluting with hexanes/EtOAc (100% hexanes to 7:1, containing 1% of NEt_3) yielded 4-chloro-7-vinyl-7*H*-pyrrolo[2,3-*d*]pyrimidine (**20**) as a colorless solid (40.1 mg, 0.223 mmol, 74%).

R*f* = 0.40 (hexanes/EtOAc, 4:1).

NMR Spectroscopy:

^{1}**H NMR** (500 MHz, $CDCl_3$, 298 K, δ): 8.67 (s, 1H), 7.59–7.49 (m, 2H), 6.70 (d, *J* = 3.7 Hz, 1H), 5.48 (dd, *J* = 16.0, 1.7 Hz, 1H), 5.01 (dd, *J* = 9.1, 1.7 Hz, 1H).

^{13}**C NMR** (75 MHz, $CDCl_3$, 298 K, δ): 152.6, 151.6, 150.6, 127.8, 125.0, 118.4, 101.9, 100.5.

HRMS-EI (m/z) calculated for $C_8H_6N_3Cl_1^+$ $[M]^+$, 179.0245; found, 179.0247; deviation: –1.0 ppm.

N-Vinyl-theophylline (21)

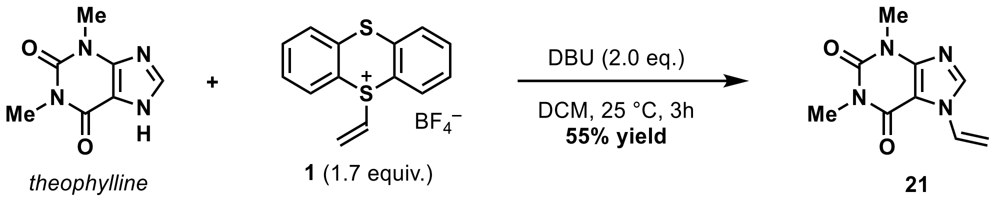

The title compound was prepared following general procedure A. Under air, a vial was charged with theophylline (54.0 mg, 0.300 mmol, 1.00 equiv.) and **1** (168 mg, 0.510 mmol, 1.70 equiv.). DCM (3.0 mL, *c*= 0.10 M) was added, followed by DBU (90 µL, 92 mg, 0.60 mmol, 2.0 equiv.), and the mixture was stirred at 25 °C for 3 h. Then, the solvent was removed under reduced pressure. Purification by column chromatography on silica gel eluting with DCM/EtOAc (100% DCM to 1:1, containing 1% of NEt_3) yielded N-vinyl-theophylline (**21**) as a colorless solid (34.0 mg, 0.165 mmol, 55%).

$\mathbf{R}_f$ = 0.19 (DCM/EtOAc, 2:1).

NMR Spectroscopy:

> **^{1}H NMR** (500 MHz, $CDCl_3$, 298 K, δ): 7.92 (s, 1H), 7.56 (dd, *J* = 16.0, 8.9 Hz, 1H), 5.58 (dd, *J* = 16.0, 2.0 Hz, 1H), 5.14 (dd, *J* = 8.9, 2.0 Hz, 1H), 3.60 (s, 3H), 3.41 (s, 3H).
>
> **^{13}C NMR** (75 MHz, $CDCl_3$, 298 K, δ): 155.2, 151.6, 149.2, 137.5, 128.7, 106.4, 105.2, 30.0, 28.2.

HRMS-ESI (m/z) calculated for $C_9H_{10}N_4O_2Na_1^+$ $[M+Na]^+$, 229.0696; found, 229.0698; deviation: –0.8 ppm.

N-Vinyl,*N'*-Acetyl-L-tryptophan ethyl ester (22)

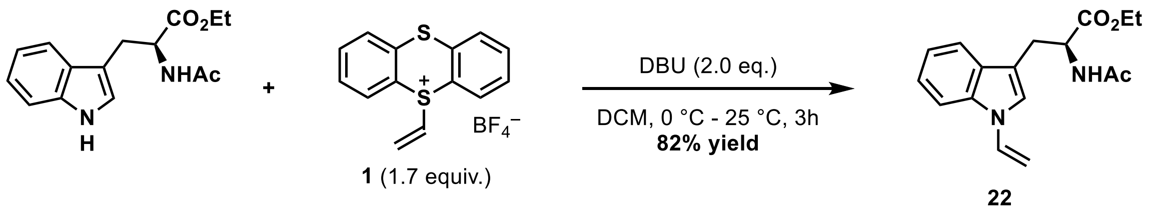

Under air, a vial was charged with *N*-acetyl-L-tryptophan ethyl ester (82.3 mg, 0.300 mmol, 1.00 equiv.) and DCM (3.0 mL, *c*= 0.10 M). DBU (90 µL, 92 mg, 0.60 mmol, 2.0 equiv.) was added, and the mixture was cooled to 0 °C. At 0 °C, a solution of **1** (168 mg, 0.510 mmol, 1.70 equiv.) in DCM (1.0 mL) was added, and the resulting solution was stirred at 0 °C for 30 min, followed by 2.5 h at 25 °C. After that, the solvent was removed under reduced pressure. Purification by column chromatography on silica gel eluting with DCM/EtOAc (5:1, containing 1% of NEt_3) yielded *N*-vinyl,*N'*-acetyl-L-tryptophan ethyl ester (**22**) as a colorless solid (74.0 mg, 0.246 mmol, 82%).

$\mathbf{R}_f$ = 0.16 (DCM/EtOAc, 5:1).

NMR Spectroscopy:

> **^{1}H NMR** (500 MHz, $CDCl_3$, 298 K, δ): 7.52 (dt, *J* = 7.8, 1.0 Hz, 1H), 7.43 (d, *J* = 8.2 Hz, 1H), 7.28–7.24

(m, 1H), 7.22–7.13 (m, 3H), 6.03 (d, *J* = 7.9 Hz, 1H), 5.10 (dd, *J* = 15.7, 1.4 Hz, 1H), 4.94 (dt, *J* = 7.8, 5.4 Hz, 1H), 4.73 (dd, *J* = 8.9, 1.4 Hz, 1H), 4.21–4.10 (m, 2H), 3.37–3.26 (m, 2H), 1.98 (s, 3H), 1.22 (t, *J* = 7.2 Hz, 3H).

^{13}C NMR (151 MHz, $CDCl_3$, 298 K, δ): 172.0, 169.8, 135.8, 129.4, 129.2, 123.2, 121.9, 120.8, 119.2, 113.0, 109.7, 96.3, 61.7, 53.0, 27.8, 23.4, 14.3.

HRMS-ESI (m/z) calculated for $C_{17}H_{19}N_2O_3^-$ [M-H]$^-$, 299.1401; found, 299.1403; deviation: –0.7 ppm.

N-Vinyl,*N'*-Boc-L-histidine methyl ester (23)

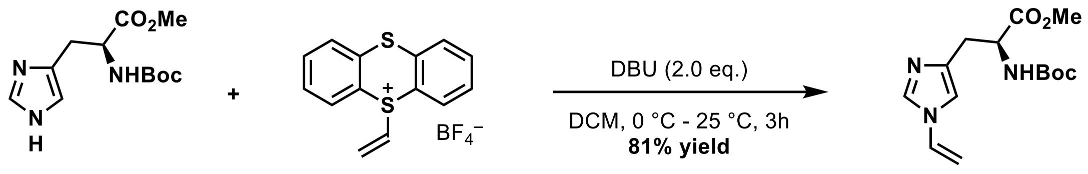

1 (1.7 equiv.) **23**

Under air, a vial was charged with *N*-Boc-L-histidine methyl ester (80.8 mg, 0.300 mmol, 1.00 equiv.), **1** (168 mg, 0.510 mmol, 1.70 equiv.) and DCM (3.0 mL, *c*= 0.10 M), and the mixture was cooled to 0 °C. DBU (90 µL, 92 mg, 0.60 mmol, 2.0 equiv.) was then added and the resulting solution was stirred at 0 °C for 30 min, followed by 2.5 h at 25 °C. After that, the solvent was removed under reduced pressure. Purification by column chromatography on silica gel eluting with DCM/MeOH (100:1 to 20:1, containing 1% of NEt_3) yielded *N*-vinyl,*N'*-Boc-L-histidine methyl ester (**23**) as an off-white solid (72.0 mg, 0.244 mmol, 81%).

R*f* = 0.21 (DCM/MeOH, 50:1).

NMR Spectroscopy:

S1H NMR (600 MHz, $CDCl_3$, 298 K, δ): 7.57 (s, 1H), 6.95 (s, 1H), 6.81 (dd, *J* = 15.7, 8.8 Hz, 1H), 5.80 (bs, 1H), 5.21 (dd, *J* = 15.8, 1.8 Hz, 1H), 4.85 (dd, *J* = 8.8, 1.8 Hz, 1H), 4.61 – 4.36 (m, 1H), 3.69 (s, 3H), 3.14 – 2.96 (m, 2H), 1.41 (s, 9H).

^{13}C NMR (75 MHz, $CDCl_3$, 298 K, δ): 172.4, 155.5, 138.5, 135.8, 129.1, 113.3, 101.5, 79.6, 53.3, 52.2, 30.2, 28.2.

HRMS-ESI (m/z) calculated for $C_{14}H_{22}N_3O_4^+$ [M+H]$^+$, 296.1605; found, 296.1603; deviation: 0.5 ppm.

N-Vinyl-metaxalone (24)

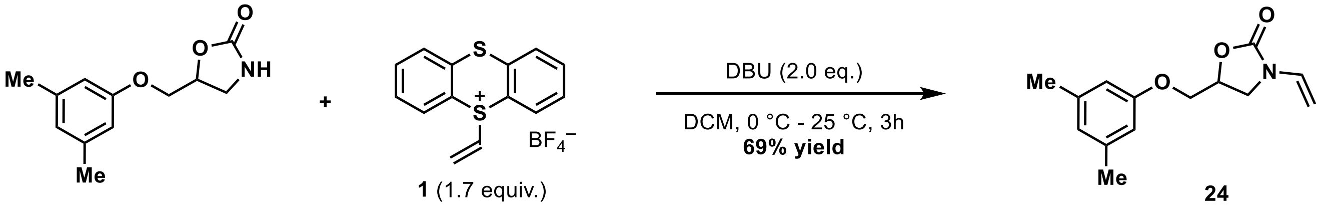

metaxalone **1** (1.7 equiv.) **24**

Under air, a vial was charged with metaxalone (66.4 mg, 0.300 mmol, 1.00 equiv.), **1** (168 mg, 0.510 mmol,

1.70 equiv.) and DCM (3.0 mL, *c*= 0.10 M), and the mixture was cooled to 0 °C. DBU (90 µL, 92 mg, 0.60 mmol, 2.0 equiv.) was then added, and the resulting solution was stirred at 0 °C for 30 min, followed by 2.5 h at 25 °C. After that, the solvent was removed under reduced pressure. Purification by column chromatography on silica gel eluting with hexanes/EtOAc (9:1 to 4:1, containing 1% of NEt_3) yielded *N*-vinyl-metaxalone (**24**) as a colorless solid (51 mg, 0.206 mmol, 69%).

Reaction run at 1.00 mmol scale: 181 mg, 0.732 mmol, 73%.

R*f* = 0.25 (hexanes/EtOAc, 4:1).

NMR Spectroscopy:

¹H NMR (500 MHz, $CDCl_3$, 298 K, δ): 6.90 (dd, *J* = 15.8, 8.9 Hz, 1H), 6.64 (s, 1H), 6.53 (s, 2H), 4.93 (ddt, *J* = 9.1, 5.8, 4.7 Hz, 1H), 4.45 (dd, *J* = 9.0, 1.3 Hz, 1H), 4.32 (dd, *J* = 15.9, 1.3 Hz, 1H), 4.16 – 4.10 (m, 2H), 3.82 (t, *J* = 9.2 Hz, 1H), 3.68 (dd, *J* = 9.3, 5.8 Hz, 1H), 2.29 (s, 6H).

¹³C NMR (75 MHz, $CDCl_3$, 298 K, δ): 158.0, 154.5, 139.3, 129.6, 123.4, 112.2, 93.5, 71.9, 67.8, 44.1, 21.3.

HRMS-ESI (m/z) calculated for $C_{14}H_{18}NO_3^+$ [M]$^+$, 248.1281; found, 248.1281; deviation: –0.1 ppm.

N-Vinyl,*N'*-Boc-carvedilol (25)

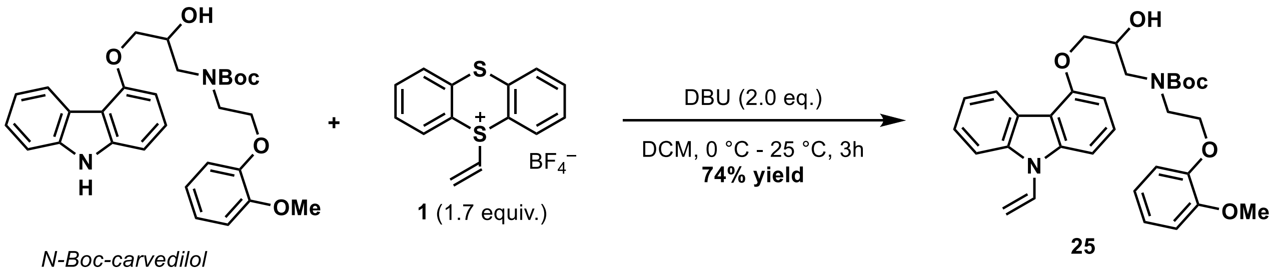

Under air, a vial was charged with N-Boc-carvedilol (151 mg, 0.300 mmol, 1.00 equiv.), **1** (168 mg, 0.510 mmol, 1.70 equiv.) and DCM (3.0 mL, *c*= 0.10 M), and the mixture was cooled to 0 °C. DBU (90 µL, 92 mg, 0.60 mmol, 2.0 equiv.) was then added, and the resulting solution was stirred at 0 °C for 30 min, followed by 2.5 h at 25 °C. After that, the solvent was removed under reduced pressure. Purification by column chromatography on silica gel eluting with DCM/EtOAc (100:0 to 20:1, containing 1% of NEt_3) yielded *N*-vinyl-*N'*-Boc-carvedilol (**25**) as a colorless solid (118 mg, 0.226 mmol, 74%).

R*f* = 0.41 (DCM/EtOAc, 9:1).

NMR Spectroscopy:

¹H NMR (300 MHz, CD_2Cl_2, 298 K, δ): 8.48 – 8.32 (m, 1H), 7.66 (d, *J* = 8.2 Hz, 1H), 7.49 – 7.22 (m, 5H), 6.97 – 6.83 (m, 4H), 6.80 (d, *J* = 7.9 Hz, 1H), 5.57 (dd, *J* = 16.0, 0.9 Hz, 1H), 5.19 (dd, *J* = 9.2, 0.9 Hz, 1H), 4.55 – 4.43 (m, 1H), 4.39 – 4.13 (m, 4H), 3.89 – 3.67 (m, 3H), 3.75 (s, 3H), 1.49 (bs, 9H).

¹³C NMR (75 MHz, CD_2Cl_2, 298 K, δ): 157.7, 155.6, 149.7, 148.5, 141.2, 139.0, 130.0, 127.4, 125.7,

123.6, 121.9, 121.2, 121.1, 121.0, 113.9, 113.3, 112.2, 110.3, 103.9, 103.0, 102.6, 80.9, 70.8, 70.5, 68.1, 55.9, 53.7, 49.5, 28.5.

HRMS-ESI (m/z) calculated for $C_{31}H_{16}N_2O_6Na^+$ $[M+Na]^+$, 555.2466; found, 555.2468; deviation: –0.4 ppm.

N-Vinyl-coumarin 7 (26)

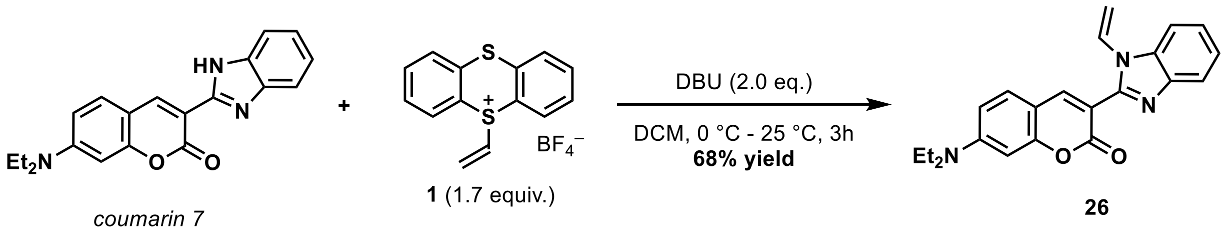

Under air, a vial was charged with coumarin 7 (100 mg, 0.300 mmol, 1.00 equiv.), **1** (168 mg, 0.510 mmol, 1.70 equiv.) and DCM (3.0 mL, *c*= 0.10 M), and the mixture was cooled to 0 °C. DBU (90 µL, 92 mg, 0.60 mmol, 2.0 equiv.) was then added, and the resulting solution was stirred at 0 °C for 30 min, followed by 2.5 h at 25 °C. After that, the solvent was removed under reduced pressure. Purification by column chromatography on silica gel eluting with DCM/EtOAc (100:0 to 20:1, containing 1% of NEt_3) yielded *N*-vinyl-coumarin 7 (**26**) as a bright yellow solid (73 mg, 0.21 mmol, 68%).

R*f* = 0.42 (DCM/EtOAc, 9:1).

NMR Spectroscopy:

^{1}H NMR (500 MHz, $CDCl_3$, 298 K, δ): 7.83 – 7.77 (m, 1H), 7.68 – 7.62 (m, 1H), 7.36 (d, *J* = 8.9 Hz, 1H), 7.35 – 7.29 (m, 2H), 7.13 (dd, *J* = 15.8, 8.9 Hz, 1H), 6.62 (dd, *J* = 8.9, 2.5 Hz, 1H), 6.53 (d, *J* = 2.5 Hz, 1H), 5.54 (dd, *J* = 15.7, 1.2 Hz, 1H), 5.20 (dd, *J* = 8.9, 1.2 Hz, 1H), 3.45 (q, *J* = 7.2 Hz, 4H), 1.24 (t, *J* = 7.1 Hz, 6H).

^{13}C NMR (75 MHz, $CDCl_3$, 298 K, δ): 159.9, 157.6, 151.9, 149.0, 147.3, 143.2, 134.5, 130.3, 130.1, 123.7, 123.0, 119.9, 111.6, 110.4, 109.5, 108.2, 107.0, 97.0, 45.0, 12.4.

HRMS-ESI (m/z) calculated for $C_{22}H_{22}N_3O_2^+$ $[M+H]^+$, 360.1707; found, 360.1710; deviation: –1.0 ppm.

N-Vinyl-lansoprazole (27)

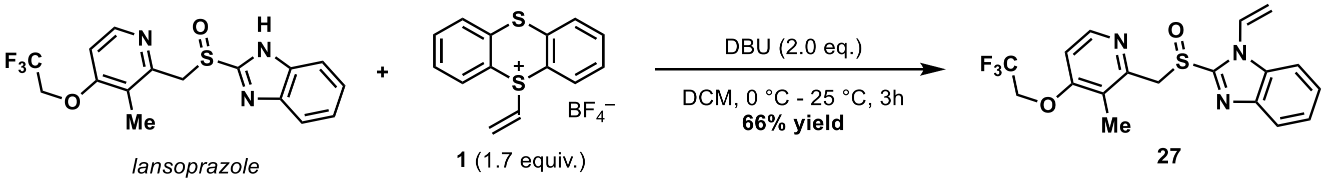

Under air, a vial was charged with lansoprazole (111 mg, 0.300 mmol, 1.00 equiv.) and DCM (3.0 mL). DBU (90 µL, 92 mg, 0.60 mmol, 2.0 equiv.) was added, and the mixture was cooled to 0 °C. At 0 °C, a solution of **1** (168 mg, 0.510 mmol, 1.70 equiv.) in DCM (1.0 mL) was added, and the resulting solution was stirred at 0 °C for 30 min, followed by 2.5 h at 25 °C. After that, the solvent was removed under reduced pressure.

Purification by column chromatography on silica gel eluting with DCM/EtOAc (1:2, containing 1% of NEt_3) yielded *N*-vinyl-lansoprazole (**27**) as a colorless solid (78.1 mg, 0.198 mmol, 66%).

R*f* = 0.15 (DCM/EtOAc, 1:2).

NMR Spectroscopy:

^{1}H NMR (300 MHz, CD_2Cl_2, 298 K, δ): 8.18 (d, *J* = 5.7 Hz, 1H), 7.82–7.77 (m, 1H), 7.71–7.64 (m, 1H), 7.50 (dd, *J* = 15.8, 8.9 Hz, 1H), 7.46–7.33 (m, 2H), 6.63 (d, *J* = 5.7 Hz, 1H), 5.70 (dd, *J* = 15.8, 1.4 Hz, 1H), 5.37 (dd, *J* = 8.9, 1.4 Hz, 1H), 5.06–4.84 (m, 2H), 4.41 (q, *J* = 8.0 Hz, 2H), 2.25 (s, 3H).

^{13}C NMR (75 MHz, CD_2Cl_2, 298 K, δ): 162.1, 153.2, 152.0, 148.5, 142.9, 134.9, 128.6, 125.7, 124.3, 123.5 (q, *J* = 277.7 Hz), 123.1, 121.5, 112.4, 109.2, 106.3, 65.8 (q, *J* = 36.2 Hz), 59.7, 11.0.

^{19}F NMR (282 MHz, CD_2Cl_2, 298 K, δ): –74.3 (s).

HRMS-ESI (m/z) calculated for $C_{18}H_{17}N_3O_2S_1F_3^+$ $[M+H]^+$, 396.0988; found, 396.0990; deviation: –0.5 ppm.

Reaction optimization of the vinylation of organoboron compounds

General procedure for optimization of reaction conditions

Under ambient atmosphere, a 4 mL vial equipped with a teflon-coated magnetic stirring bar was charged with 4-biphenylboronic acid (**S4**, 9.9 mg, 0.050 mmol, 1.0 equiv.), $Pd(dba)_2$ (1.4 mg, 2.5 µmol, 5.0 mol%), P(*o*-tol)$_3$ (1.7 mg, 5.5 µmol, 11 mol%) and *t*-BuOLi (6.0 mg, 0.075 mmol, 1.5 equiv.). The vial was transferred into a N_2-filled glove box. Subsequently, dry THF (0.5 mL, c = 0.1 M) was added into the vial. The reaction mixture was stirred for 2 min at 25 °C before **1** (24.8 mg, 0.0750 mmol, 1.50 equiv.) was added into the vial in one portion. The vial was capped and was then transferred out of the glove box. The vial was placed on a heating block preheated at 60 °C where the reaction mixture was stirred for 16 h. The reaction mixture was cooled to 25 °C and filtered through a pad of celite eluting with DCM (4 mL). The filtrate was collected and concentrated under reduced pressure. To the residue was added dibromomethane (7.0 µL, 17 mg, 0.10 mmol) as an internal standard. The ^{1}H NMR resonances of the vinyl protons of the product between 5.1 and 6.0 ppm were integrated relative to the ^{1}H NMR resonances of the protons of dibromomethane (δ = 4.90 ppm).

Table S1: Optimization of yield as a function of catalyst

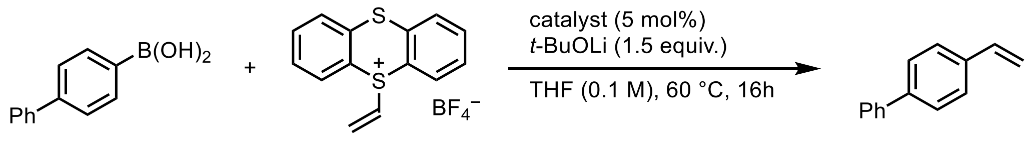

S4 **1** **S5**

Entry	Catalyst	Yield
1	No catalyst	0%

2	$Pd(OAc)_2$ + 11 mol% PPh_3	47%
3	$Pd(OAc)_2$ + 11 mol% P(*o*-tol)$_3$	50%
4	$Pd(OAc)_2$ + 11 mol% P(2-fur)$_3$	39%
5	$Pd(dba)_2$ + 11 mol% PPh_3	45%
**6**	**$Pd(dba)_2$ + 11 mol% P(*o*-tol)$_3$**	**60%**
7	$Pd(dba)_2$ + 11 mol% P(2-fur)$_3$	43%
8	Pd(dppf)Cl_2	5%
9	Pd(PCy_3)$_2$$Cl_2$	0%
10	Pd(P*t*Bu_3)$_2$	42%
11	Pd(PPh_3)$_4$	40%

Table S2: Optimization of yield as a function of base

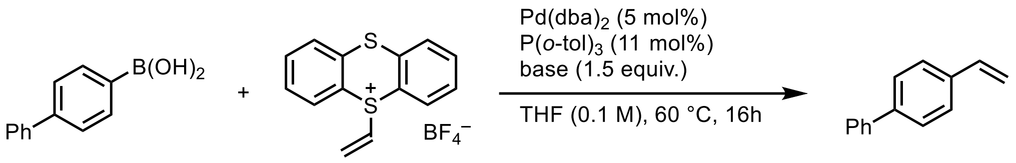

S4 **1** **S5**

Entry	Base	Yield
1	No base	<5%
2	Na_2CO_3	11%
3	Cs_2CO_3	76%
4	K_3PO_4	60%
5	CsF	75%
**6**	***t*-BuOLi**	**81%**
7	*t*-BuONa	<5%
8	*t*-BuOK	10%
9	TMSOK	11%

Table S3: Optimization of yield as a function of concentration

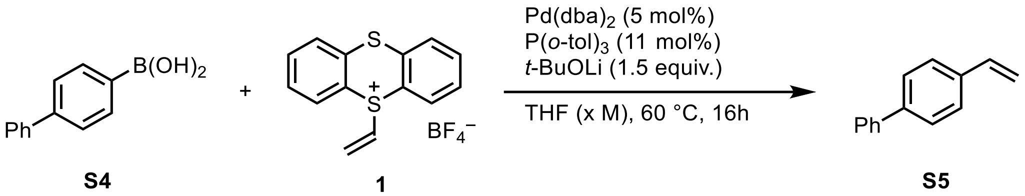

Entry	Concentration	Yield
1	0.025 M	52%
**2**	**0.05 M**	**90%**
3	0.1 M	81%
4	0.2 M	68%

Suzuki-type vinylation of aryl organoboron compounds using vinyl-TT$^+$ 1

General procedure B

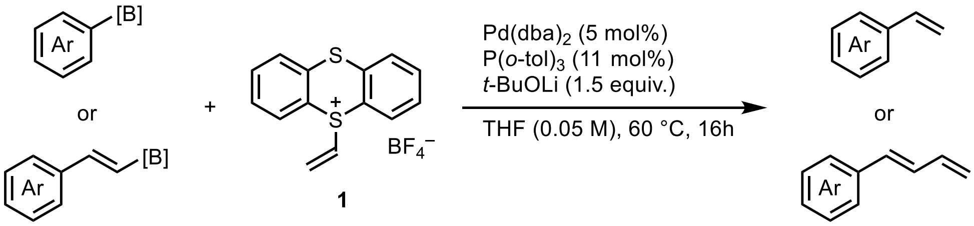

Under ambient atmosphere, a 20 mL vial equipped with a teflon-coated magnetic stirring bar was charged with organoboron species (0.300 mmol, 1.00 equiv.), Pd(dba)$_2$ (8.6 mg, 15 µmol, 5.0 mol%), P(*o*-tol)$_3$ (10.0 mg, 33.0 µmol, 11.0 mol%) and *t*-BuOLi (36.0 mg, 0.450 mmol, 1.50 equiv.). The vial was transferred into a N$_2$-filled glove box. Subsequently, dry THF (6 mL, c = 0.05 M) was added into the vial. The reaction mixture was stirred for 2 min at 25 °C before **1** (149 mg, 0.450 mmol, 1.50 equiv.) was added into the vial in one portion. The vial was capped and was then transferred out of the glove box. The vial was placed on a heating block preheated at 60 °C where the reaction mixture was stirred for 16 h. The reaction mixture was cooled to 25 °C and filtered through a pad of celite eluting with DCM (20 mL). The filtrate was collected and concentrated under vacuum to roughly 5 mL. Silica gel (approximately 300 mg) was added, and the mixture was evaporated to dryness under reduced pressure. The residue was purified by column chromatography on silica gel to give corresponding product. *[Note: unless otherwise mentioned, t-BuOLi stored at ambient atmosphere was used. When extra-dry t-BuOLi stored in the glovebox was used, poor yields were obtained]*.

General procedure for the vinylation of organoboron compounds using a Schlenk line

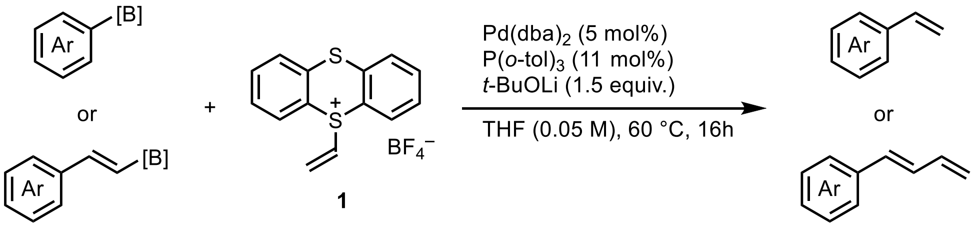

Under ambient atmosphere, a 20 mL Schlenk tube equipped with a teflon-coated magnetic stirring bar was charged with organoboron species (0.300 mmol, 1.00 equiv.), Pd(dba)$_2$ (8.6 mg, 15 µmol, 5.0 mol%), P(*o*-tol)$_3$ (10.0 mg, 33.0 µmol, 11.0 mol%) and *t*-BuOLi (36.0 mg, 0.450 mmol, 1.50 equiv.). The Schlenk tube was evacuated and backfilled with argon. Subsequently, dry THF (6 mL, c = 0.05 M) was added into the Schlenk tube via a syringe. The reaction mixture was stirred for 2 min at 25 °C before **1** (149 mg, 0.450 mmol, 1.50 equiv.) was added into the Schlenk tube in one portion. The Schlenk tube was placed in an oil bath preheated at 60 °C where the reaction mixture was stirred for 16 h. The reaction mixture was cooled to 25 °C and filtered through a pad of celite eluting with DCM (20 mL). The filtrate was collected and concentrated under vacuum to roughly 5 mL. Silica gel (approximately 300 mg) was added, and the mixture was evaporated to dryness under reduced pressure. The residue was purified by column chromatography on silica gel to give corresponding product.

1-Methyl-3-vinylbenzene (28)

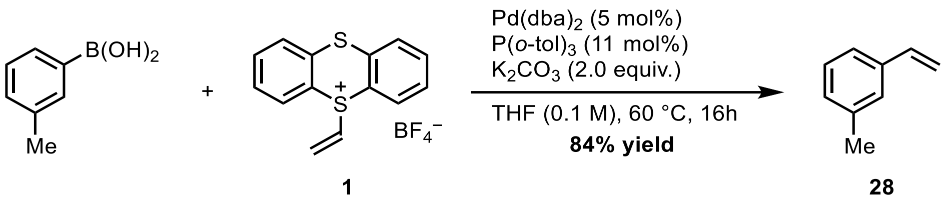

Under ambient atmosphere, a 4 mL vial equipped with a teflon-coated magnetic stirring bar was charged with 3-methylbenzeneboronic acid (6.8 mg, 0.050 mmol, 1.0 equiv.), Pd(dba)$_2$ (1.4 mg, 2.5 µmol, 5.0 mol%), P(*o*-tol)$_3$ (1.7 mg, 5.5 µmol, 11 mol%) and *t*-BuOLi (6.0 mg, 0.075 mmol, 1.5 equiv.). The vial was transferred into a N_2-filled glove box. Subsequently, dry THF (0.5 mL, c = 0.1 M) was added into the vial. The reaction mixture was stirred for 2 min at 25 °C before **1** (24.8 mg, 0.0750 mmol, 1.50 equiv.) was added into the vial in one portion. The vial was capped and was then transferred out of the glove box. The vial was placed on a heating block preheated at 60 °C where the reaction mixture was stirred for 16 h. The reaction mixture was cooled to 25 °C and filtered through a pad of celite eluting with DCM (4 mL). The filtrate was collected and concentrated under reduced pressure. Due to the volatility of the title product, its yield was determined via NMR analysis of the reaction mixture. To the residue was added dibromomethane (7.0 µL, 17 mg, 0.10 mmol, 2.0 equiv.) as an internal standard. The ^{1}H NMR resonances of the vinyl protons of the product between 5.6 and 5.8 ppm were integrated relative to the ^{1}H NMR resonances of the protons of dibromomethane (δ = 4.90 ppm). The yield was determined as 84% (Figure S9).

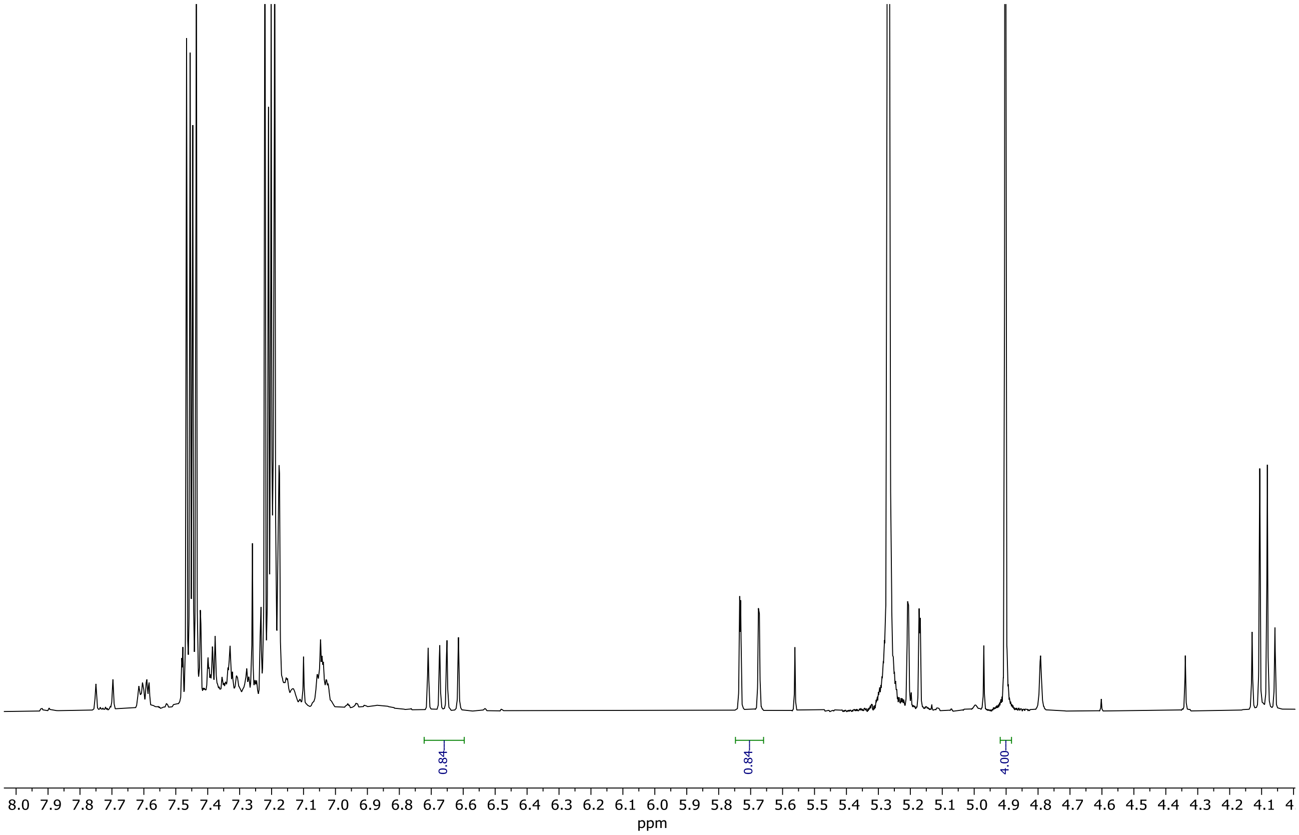

Figure S9. Determination of the yield of **28** via ^{1}H NMR analysis, $CDCl_3$, 300 MHz, 298 K.

3-Methyl-4-vinylbenzonitrile (29)

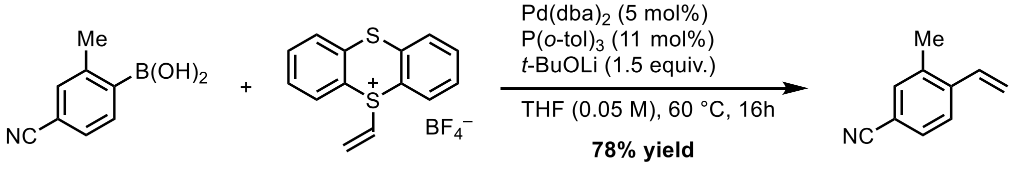

The title compound was prepared following general procedure B. Under ambient atmosphere, a 20 mL vial equipped with a teflon-coated magnetic stirring bar was charged with 2-methyl-4-cyanophenylboronic acid (48.3 mg, 0.300 mmol, 1.00 equiv.), $Pd(dba)_2$ (8.6 mg, 15 µmol, 5.0 mol%), P(*o*-tol)$_3$ (10.0 mg, 33.0 µmol, 11.0 mol%) and *t*-BuOLi (36.0 mg, 0.450 mmol, 1.50 equiv.). The vial was transferred into a N_2-filled glove box. Subsequently, dry THF (6 mL, c = 0.05 M) was added into the vial. The reaction mixture was stirred for 2 min at 25 °C before **1** (149 mg, 0.450 mmol, 1.50 equiv.) was added into the vial in one portion. The vial was capped and was then transferred out of the glove box. The vial was placed on a heating block preheated at 60 °C where the reaction mixture was stirred for 16 h. The reaction mixture was cooled to 25 °C and filtered through a pad of celite eluting with DCM (20 mL). The filtrate was collected and concentrated under vacuum to roughly 5 mL. Silica gel (approximately 300 mg) was added, and the mixture was evaporated to dryness under reduced pressure. The residue was purified by column chromatography on silica gel eluting with a

solvent mixture of EtOAc:pentane (1:50 (v:v)) to afford 33.5 mg of the title compound (**29**) as a colorless oil (78% yield).

R*f* = 0.35 (EtOAc:pentane, 1:19 (v:v)).

NMR Spectroscopy:

^{1}H NMR (500 MHz, $CDCl_3$, 298 K, δ): 7.54 (m, 1H), 7.47 – 7.38 (m, 2H), 6.91 (dd, *J* = 17.4, 11.1 Hz, 1H), 5.75 (dd, *J* = 17.4, 1.0 Hz, 1H), 5.47 (dd, *J* = 11.0, 1.0 Hz, 1H), 2.37 (s, 3H) ppm.

^{13}C NMR (126 MHz, $CDCl_3$, 298 K, δ): 141.6, 136.6, 133.8, 133.6, 129.9, 126.1, 119.2, 118.7, 111.1, 19.6 ppm.

HRMS-EI (m/z) calculated for $C_{10}H_9N_1^+$ [M]$^+$, 143.0731; found, 143.0729; deviation: –1.1 ppm.

1-Chloro-4-methoxy-2-vinylbenzene (30)

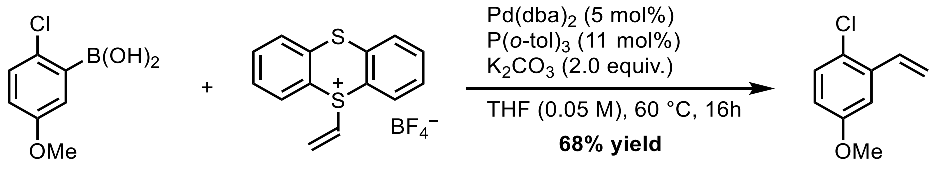

1 **30**

The title compound was prepared following general procedure B. Under ambient atmosphere, a 20 mL vial equipped with a teflon-coated magnetic stirring bar was charged with 2-chloro-5-methoxyphenylboronic acid (55.9 mg, 0.300 mmol, 1.00 equiv.), $Pd(dba)_2$ (8.6 mg, 15 µmol, 5.0 mol%), P(*o*-tol)$_3$ (10.0 mg, 33.0 µmol, 11.0 mol%) and K_2CO_3 (82.9 mg, 0.600 mmol, 2.00 equiv.). The vial was transferred into a N_2-filled glove box. Subsequently, dry THF (6 mL, c = 0.05 M) was added into the vial. The reaction mixture was stirred for 2 min at 25 °C before **1** (149 mg, 0.450 mmol, 1.50 equiv.) was added into the vial in one portion. The vial was capped and was then transferred out of the glove box. The vial was placed on a heating block preheated at 60 °C where the reaction mixture was stirred for 16 h. The reaction mixture was cooled to 25 °C and filtered through a pad of celite eluting with DCM (20 mL). The filtrate was collected and concentrated under vacuum to roughly 5 mL. Silica gel (approximately 300 mg) was added, and the mixture was evaporated to dryness under reduced pressure. The residue was purified by column chromatography on silica gel eluting with pentane to afford 34.3 mg of the title compound (**30**) as a colorless oil (68% yield).

R*f* = 0.40 (EtOAc:pentane, 1:19 (v:v)).

NMR Spectroscopy:

^{1}H NMR (500 MHz, $CDCl_3$, 298 K, δ): 7.25 (d, *J* = 8.8 Hz, 1H), 7.15 – 6.98 (m, 2H), 6.77 (dd, *J* = 8.8, 3.0 Hz, 1H), 5.73 (dd, *J* = 17.5, 1.1 Hz, 1H), 5.38 (dd, *J* = 11.0, 1.1 Hz, 1H), 3.81 (s, 3H) ppm.

^{13}C NMR (126 MHz, $CDCl_3$, 298 K, δ): 158.5, 136.6, 133.5, 130.4, 125.0, 116.7, 115.0, 111.7, 55.7 ppm.

HRMS-EI (m/z) calculated for $C_9H_9O_1Cl_1^+$ [M]$^+$, 168.0337; found, 168.0336; deviation: –0.6 ppm.

1-Chloro-4-vinylbenzene (31)

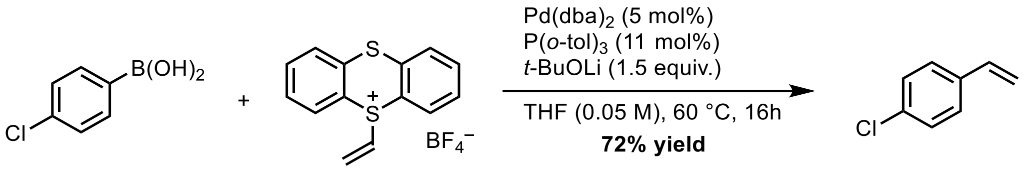

1 **31**

The title compound was prepared following general procedure B. Under ambient atmosphere, a 20 mL vial equipped with a teflon-coated magnetic stirring bar was charged with 4-chlorophenylboronic acid (46.9 mg, 0.300 mmol, 1.00 equiv.), $Pd(dba)_2$ (8.6 mg, 15 µmol, 5.0 mol%), P(*o*-tol)$_3$ (10.0 mg, 33.0 µmol, 11.0 mol%) and *t*-BuOLi (36.0 mg, 0.450 mmol, 1.50 equiv.). The vial was transferred into a N_2-filled glove box. Subsequently, dry THF (6 mL, c = 0.05 M) was added into the vial. The reaction mixture was stirred for 2 min at 25 °C before **1** (149 mg, 0.450 mmol, 1.50 equiv.) was added into the vial in one portion. The vial was capped and was then transferred out of the glove box. The vial was placed on a heating block preheated at 60 °C where the reaction mixture was stirred for 16 h. The reaction mixture was cooled to 25 °C and filtered through a pad of celite eluting with DCM (20 mL). The filtrate was collected and concentrated under vacuum to roughly 5 mL. Silica gel (approximately 300 mg) was added, and the mixture was evaporated to dryness under reduced pressure. The residue was purified by column chromatography on silica gel eluting with pentane to afford 34.3 mg of the title compound (**31**) as a colorless oil (72% yield).

R*f* = 0.51 (pentane).

NMR Spectroscopy:

^{1}**H NMR** (300 MHz, $CDCl_3$, 298 K, δ): 7.43 – 7.27 (m, 4H), 6.67 (dd, *J* = 17.6, 10.9 Hz, 1H), 5.73 (dd, *J* = 17.6, 0.8 Hz, 1H), 5.27 (dd, *J* = 10.9, 0.8 Hz, 1H) ppm.

^{13}**C NMR** (75 MHz, $CDCl_3$, 298 K, δ): 136.2, 135.8, 133.6, 128.8, 127.6, 114.6 ppm.

HRMS-EI (m/z) calculated for $C_8H_7Cl_1^+$ $[M]^+$, 138.0232; found, 138.0230; deviation: –0.8 ppm.

1-Chloro-4-(vinyl-*d*$_3$)benzene (31-*d*$_3$)

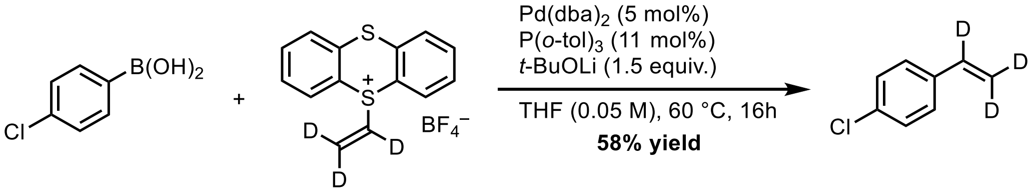

1-*d*$_3$ **31-*d*$_3$**

Under ambient atmosphere, a 4 mL vial equipped with a teflon-coated magnetic stirring bar was charged with 4-chlorophenylboronic acid (7.8 mg, 0.050 mmol, 1.0 equiv.), $Pd(dba)_2$ (1.4 mg, 2.5 µmol, 5.0 mol%), P(*o*-tol)$_3$ (1.7 mg, 5.5 µmol, 11 mol%) and *t*-BuOLi (6.0 mg, 0.075 mmol, 1.5 equiv.). The vial was transferred into a N_2-filled glove box. Subsequently, dry THF (1 mL, c = 0.05 M) was added into the vial. The reaction mixture

was stirred for 2 min at 25 °C before **1-*d*$_3$** (25.0 mg, 0.0750 mmol, 1.50 equiv.) was added into the vial in one portion. The vial was capped and was then transferred out of the glove box. The vial was placed on a heating block preheated at 60 °C where the reaction mixture was stirred for 16 h. The reaction mixture was cooled to 25 °C and filtered through a pad of celite eluting with DCM (5 mL). Silica gel (approximately 50 mg) was added to the filtrate, and the mixture was evaporated to dryness under reduced pressure. The residue was purified by column chromatography on silica gel eluting with pentane to afford 4.1 mg of the title compound (**31-*d*$_3$**) as a colorless oil (58% yield).

R*f* = 0.52 (pentane).

NMR Spectroscopy:

^{1}H NMR (300 MHz, CD_2Cl_2, 298 K, δ): 7.42 – 7.34 (m, 2H), 7.33 – 7.26 (m, 2H) ppm.

^{2}H NMR (92 MHz, CD_2Cl_2, 298 K, δ): 6.71 (s, 1H), 5.76 (s, 1H), 5.30 (s, 1H) ppm.

^{13}C NMR (151 MHz, CD_2Cl_2, 298 K, δ): 136.5, 135.5 (t, *J* = 24.9 Hz), 133.7, 129.0, 127.9, 114.2 (m) ppm.

HRMS-EI (m/z) calculated for $C_8H_4D_3Cl_1^+$ [M]$^+$, 141.0421; found, 141.0419; deviation: –1.6 ppm.

1-Ethoxy-2-vinylbenzene (32)

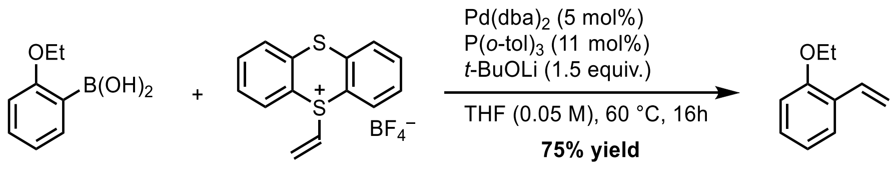

1 32

The title compound was prepared following general procedure B. Under ambient atmosphere, a 20 mL vial equipped with a teflon-coated magnetic stirring bar was charged with 2-ethoxyphenylboronic acid (49.8 mg, 0.300 mmol, 1.00 equiv.), $Pd(dba)_2$ (8.6 mg, 15 µmol, 5.0 mol%), P(*o*-tol)$_3$ (10.0 mg, 33.0 µmol, 11.0 mol%) and *t*-BuOLi (36.0 mg, 0.450 mmol, 1.50 equiv.). The vial was transferred into a N_2-filled glove box. Subsequently, dry THF (6 mL, c = 0.05 M) was added into the vial. The reaction mixture was stirred for 2 min at 25 °C before **1** (149 mg, 0.450 mmol, 1.50 equiv.) was added into the vial in one portion. The vial was capped and was then transferred out of the glove box. The vial was placed on a heating block preheated at 60 °C where the reaction mixture was stirred for 16 h. The reaction mixture was cooled to 25 °C and filtered through a pad of celite eluting with DCM (20 mL). The filtrate was collected and concentrated under vacuum to roughly 5 mL. Silica gel (approximately 300 mg) was added, and the mixture was evaporated to dryness under reduced pressure. The residue was purified by column chromatography on silica gel eluting with pentane to afford 33.3 mg of the title compound (**32**) as a colorless oil (75% yield).

R*$_f$* = 0.51 (EtOAc:pentane, 1:19 (v:v)).

NMR Spectroscopy:

^{1}H NMR (300 MHz, $CDCl_3$, 298 K, δ): 7.86 (dd, *J* = 7.7, 1.8 Hz, 1H), 7.60 (ddd, *J* = 8.2, 7.4, 1.7 Hz, 1H), 7.46 (dd, *J* = 17.9, 11.2 Hz, 1H), 7.31 (td, *J* = 7.5, 0.6 Hz, 1H), 7.24 (dd, *J* = 8.3, 1.1 Hz, 1H), 6.14 (dd, *J* = 17.8, 1.6 Hz, 1H), 5.64 (dd, *J* = 11.2, 1.6 Hz, 1H), 4.45 (q, *J* = 7.0 Hz, 2H), 1.83 (t, *J* = 7.0 Hz, 3H) ppm.

^{13}C NMR (75 MHz, $CDCl_3$, 298 K, δ): 156.3, 132.0, 128.9, 127.0, 126.7, 120.7, 114.4, 112.2, 64.0, 15.0 ppm.

HRMS-EI (m/z) calculated for $C_{10}H_{12}O_1^+$ $[M]^+$, 148.0885; found, 148.0883; deviation: –1.3 ppm.

1-Bromo-4-vinylbenzene (33)

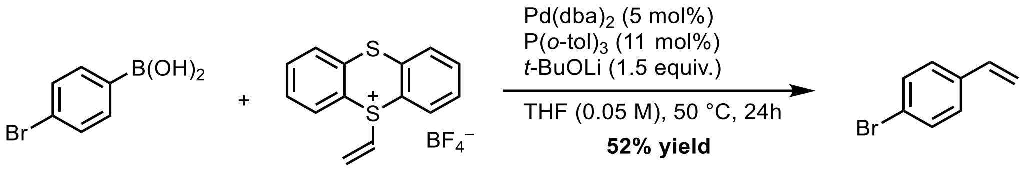

Under ambient atmosphere, a 20 mL vial equipped with a teflon-coated magnetic stirring bar was charged with **1** (168 mg, 0.510 mmol, 1.70 equiv.), $Pd(dba)_2$ (8.6 mg, 15 µmol, 5.0 mol%), P(*o*-tol)$_3$ (10.0 mg, 33.0 µmol, 11.0 mol%) and *t*-BuOLi (36.0 mg, 0.450 mmol, 1.50 equiv.). The vial was transferred into a N_2-filled glove box. Subsequently, dry THF (6 mL, c = 0.05 M) was added into the vial. The reaction mixture was stirred for 2 min at 25 °C before 4-bromophenylboronic acid (60.2 mg, 0.300 mmol, 1.00 equiv.) was added into the vial in one portion. The vial was capped and was then transferred out of the glove box. The vial was placed on a heating block preheated at 50 °C where the reaction mixture was stirred for 24 h. The reaction mixture was cooled to 25 °C and filtered through a pad of celite eluting with DCM (20 mL). The filtrate was collected and concentrated under vacuum to roughly 5 mL. Silica gel (approximately 300 mg) was added, and the mixture was evaporated to dryness under reduced pressure. The residue was purified by column chromatography on silica gel eluting with pentane to afford 28.5 mg of the title compound (**33**) as a colorless oil (52% yield).

R*f* = 0.51 (pentane).

NMR Spectroscopy:

^{1}H NMR (500 MHz, $CDCl_3$, 298 K, δ): 7.49 – 7.40 (m, 2H), 7.32 – 7.24 (m, 2H), 6.66 (dd, *J* = 17.6, 10.8 Hz, 1H), 5.74 (dd, *J* = 17.6, 0.7 Hz, 1H), 5.28 (dd, *J* = 11.0, 0.7 Hz, 1H) ppm.

^{13}C NMR (126 MHz, $CDCl_3$, 298 K, δ): 136.6, 135.9, 131.8, 127.9, 121.7, 114.8 ppm.

HRMS-EI (m/z) calculated for $C_8H_7Br_1^+$ $[M]^+$, 181.9727; found, 181.9726; deviation: –0.6 ppm.

4-Vinylbenzaldehyde (34)

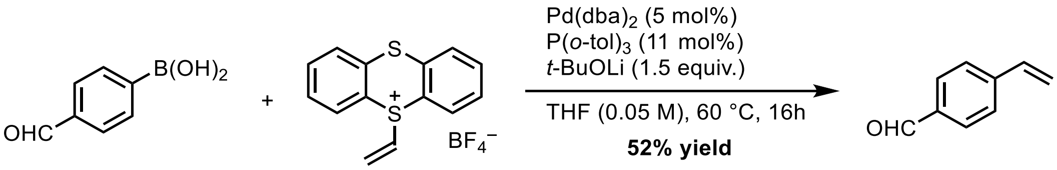

1 **34**

The title compound was prepared following general procedure B. Under ambient atmosphere, a 20 mL vial equipped with a teflon-coated magnetic stirring bar was charged with 4-formylphenylboronic acid (45.0 mg, 0.300 mmol, 1.00 equiv.), Pd(dba)$_2$ (8.6 mg, 15 µmol, 5.0 mol%), P(*o*-tol)$_3$ (10.0 mg, 33.0 µmol, 11.0 mol%) and *t*-BuOLi (36.0 mg, 0.450 mmol, 1.50 equiv.). The vial was transferred into a N$_2$-filled glove box. Subsequently, dry THF (6 mL, c = 0.05 M) was added into the vial. The reaction mixture was stirred for 2 min at 25 °C before **1** (149 mg, 0.450 mmol, 1.50 equiv.) was added into the vial in one portion. The vial was capped and was then transferred out of the glove box. The vial was placed on a heating block preheated at 60 °C where the reaction mixture was stirred for 16 h. The reaction mixture was cooled to 25 °C and filtered through a pad of celite eluting with DCM (20 mL). The filtrate was collected and concentrated under vacuum to roughly 5 mL. Silica gel (approximately 300 mg) was added, and the mixture was evaporated to dryness under reduced pressure. The residue was purified by column chromatography on silica gel eluting with a solvent mixture of EtOAc:pentane (1:50, (v:v)) to afford 21.0 mg of the title compound (**34**) as a colorless oil (52% yield).

R*f* = 0.29 (EtOAc:pentane, 1:19 (v:v)).

NMR Spectroscopy:

^{1}H NMR (500 MHz, CDCl$_3$, 298 K, δ): 9.99 (s, 1H), 8.05 – 7.78 (m, 2H), 7.58 – 7.52 (m, 2H), 6.77 (dd, *J* = 17.6, 10.9 Hz, 1H), 5.91 (dd, *J* = 17.6, 0.6 Hz, 1H), 5.44 (dd, *J* = 10.9, 0.6 Hz, 1H) ppm.

^{13}C NMR (75 MHz, CDCl$_3$, 298 K, δ): 191.86, 143.62, 136.06, 135.85, 130.25, 126.90, 117.61 ppm.

HRMS-EI (m/z) calculated for $C_9H_8O_1^+$ [M]$^+$, 132.0571; found, 132.0570; deviation: –0.9 ppm.

2-Vinylbenzo[b]thiophene (35)

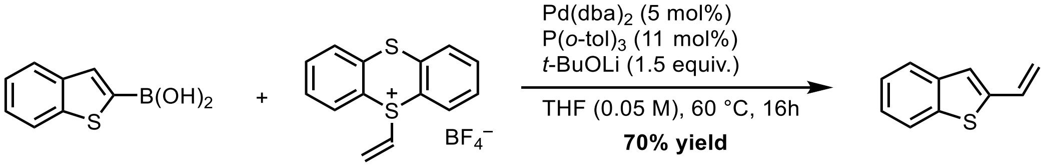

1 **35**

The title compound was prepared following general procedure B. Under ambient atmosphere, a 20 mL vial equipped with a teflon-coated magnetic stirring bar was charged with benzo[b]thien-2-ylboronic acid (53.4 mg, 0.300 mmol, 1.00 equiv.), Pd(dba)$_2$ (8.6 mg, 15 µmol, 5.0 mol%), P(*o*-tol)$_3$ (10.0 mg, 33.0 µmol, 11.0 mol%) and *t*-BuOLi (36.0 mg, 0.450 mmol, 1.50 equiv.). The vial was transferred into a N$_2$-filled glove

box. Subsequently, dry THF (6 mL, c = 0.05 M) was added into the vial. The reaction mixture was stirred for 2 min at 25 °C before **1** (149 mg, 0.450 mmol, 1.50 equiv.) was added into the vial in one portion. The vial was capped and was then transferred out of the glove box. The vial was placed on a heating block preheated at 60 °C where the reaction mixture was stirred for 16 h. The reaction mixture was cooled to 25 °C and filtered through a pad of celite eluting with DCM (20 mL). The filtrate was collected and concentrated under vacuum to roughly 5 mL. Silica gel (approximately 300 mg) was added, and the mixture was evaporated to dryness under reduced pressure. The residue was purified by column chromatography on silica gel eluting with pentane to afford 33.6 mg of the title compound (**35**) as a white solid (70% yield).

R*f* = 0.29 (pentane).

NMR Spectroscopy:

^{1}H NMR (500 MHz, $CDCl_3$, 298 K, δ): 7.86 – 7.74 (m, 1H), 7.74 – 7.59 (m, 1H), 7.41 – 7.27 (m, 2H), 7.18 (s, 1H), 6.93 (ddd, *J* = 17.2, 10.7, 0.6 Hz, 1H), 5.68 (d, *J* = 17.3 Hz, 1H), 5.32 (d, *J* = 10.7 Hz, 1H) ppm.

^{13}C NMR (126 MHz, $CDCl_3$, 298 K, δ): 143.2, 140.1, 139.0, 130.7, 124.9, 124.5, 123.7, 123.2, 122.4, 116.1 ppm.

HRMS-EI (m/z) calculated for $C_{10}H_8S_1^+$ $[M]^+$, 160.0341; found, 160.0341; deviation: +0.1 ppm.

1-Methyl-3-(trifluoromethyl)-5-vinyl-1*H*-pyrazole (36)

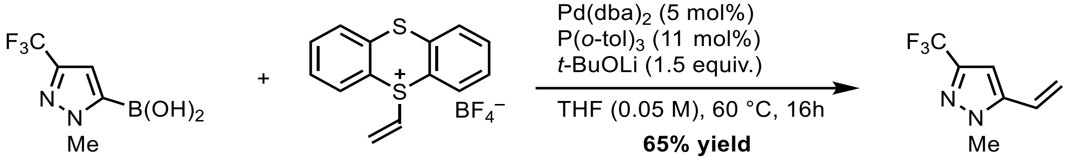

1 **36**

The title compound was prepared following general procedure B. Under ambient atmosphere, a 20 mL vial equipped with a teflon-coated magnetic stirring bar was charged with (1-methyl-3-(trifluoromethyl)-1*H*-pyrazol-5-yl)boronic acid (58.2 mg, 0.300 mmol, 1.00 equiv.), $Pd(dba)_2$ (8.6 mg, 15 µmol, 5.0 mol%), P(*o*-tol)$_3$ (10.0 mg, 33.0 µmol, 11.0 mol%) and *t*-BuOLi (36.0 mg, 0.450 mmol, 1.50 equiv.). The vial was transferred into a N_2-filled glove box. Subsequently, dry THF (6 mL, c = 0.05 M) was added into the vial. The reaction mixture was stirred for 2 min at 25 °C before **1** (149 mg, 0.450 mmol, 1.50 equiv.) was added into the vial in one portion. The vial was capped and was then transferred out of the glove box. The vial was placed on a heating block preheated at 60 °C where the reaction mixture was stirred for 16 h. The reaction mixture was cooled to 25 °C and filtered through a pad of celite eluting with DCM (20 mL). The filtrate was collected and concentrated under vacuum to roughly 5 mL. Silica gel (approximately 300 mg) was added, and the mixture was evaporated to dryness under reduced pressure at temperature < 30 °C. The residue was purified by column chromatography on silica gel eluting with a solvent mixture of DCM:pentane (1:2, (v:v)) to afford 34.3 mg of the title compound (**36**) as a colorless oil (65% yield). *[Note: due to the volatility of the product, drying of the purified product was processed under vacuum in a bath of dry ice]*.

R*f* = 0.30 (DCM:pentane, 1:1 (v:v)).

NMR Spectroscopy:

^{1}H NMR (500 MHz, $CDCl_3$, 298 K, δ): 6.62 (s, 1H), 6.58 (dd, *J* = 17.5, 11.2 Hz, 1H), 5.76 (dd, *J* = 17.4, 1.0 Hz, 1H), 5.57 – 5.35 (m, 1H), 3.91 (s, 3H) ppm.

^{13}C NMR (151 MHz, $CDCl_3$, 298 K, δ): 142.6, 141.6 (q, *J* = 38.0 Hz), 122.7, 121.4 (q, *J* = 268.4 Hz), 119.8, 101.6 (q, *J* = 2.3 Hz), 37.4 ppm.

^{19}F NMR (471 MHz, $CDCl_3$, 298 K, δ): -62.23 ppm.

HRMS-EI (m/z) calculated for $C_7H_7N_2F_3^+$ [M]$^+$, 176.0556; found, 176.0556; deviation: 0.2 ppm.

1-Chloro-4-(trifluoromethyl)-2-((2-vinylbenzyl)oxy)benzene (37)

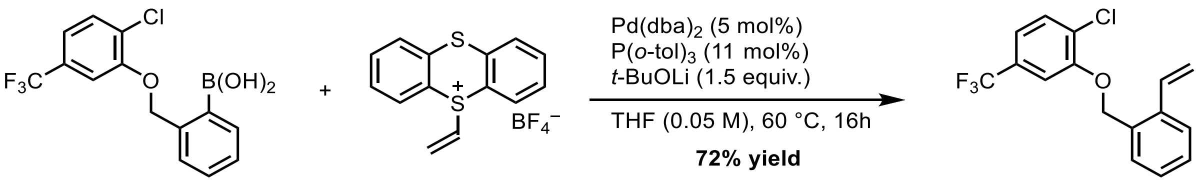

1 **37**

The title compound was prepared following general procedure B. Under ambient atmosphere, a 20 mL vial equipped with a teflon-coated magnetic stirring bar was charged with 2-((2'-Chloro-5'-(trifluoromethyl)phenoxy)methyl)phenylboronic acid (99.1 mg, 0.300 mmol, 1.00 equiv.), $Pd(dba)_2$ (8.6 mg, 15 µmol, 5.0 mol%), P(*o*-tol)$_3$ (10.0 mg, 33.0 µmol, 11.0 mol%) and *t*-BuOLi (36.0 mg, 0.450 mmol, 1.50 equiv.). The vial was transferred into a N_2-filled glove box. Subsequently, dry THF (6 mL, c = 0.05 M) was added into the vial. The reaction mixture was stirred for 2 min at 25 °C before **1** (149 mg, 0.450 mmol, 1.50 equiv.) was added into the vial in one portion. The vial was capped and was then transferred out of the glove box. The vial was placed on a heating block preheated at 60 °C where the reaction mixture was stirred for 16 h. The reaction mixture was cooled to 25 °C and filtered through a pad of celite eluting with DCM (20 mL). The filtrate was collected and concentrated under vacuum to roughly 5 mL. Silica gel (approximately 300 mg) was added, and the mixture was evaporated to dryness under reduced pressure. The residue was purified by column chromatography on silica gel eluting with pentane to afford 67.4 mg of the title compound (**37**) as a white solid (72% yield).

R*f* = 0.51 (EtOAc:pentane, 1:19 (v:v)).

NMR Spectroscopy:

^{1}H NMR (500 MHz, $CDCl_3$, 298 K, δ): 7.57 (dd, *J* = 7.6, 1.5 Hz, 1H), 7.50 (ddd, *J* = 8.0, 6.0, 1.2 Hz, 2H), 7.37 (td, *J* = 7.5, 1.6 Hz, 1H), 7.32 (td, *J* = 7.5, 1.5 Hz, 1H), 7.23 (m, 1H), 7.19 (ddd, *J* = 8.2, 1.9, 0.8 Hz, 1H), 7.03 (dd, *J* = 17.3, 11.0 Hz, 1H), 5.71 (dd, *J* = 17.4, 1.3 Hz, 1H), 5.40 (dd, *J* = 11.0, 1.2 Hz, 1H), 5.21 (s, 2H) ppm.

^{13}C NMR (126 MHz, $CDCl_3$, 298 K, δ): 154.4, 137.3, 133.6, 132.3, 130.7, 129.9 (q, *J* = 32.8 Hz), 128.9,

128.8, 128.0, 127.4, 126.3, 123.6 (q, *J* = 272.4 Hz), 118.5 (q, *J* = 3.9 Hz), 117.3, 110.6 (q, *J* = 3.7 Hz), 69.4 ppm.

^{19}F NMR (471 MHz, $CDCl_3$, 298 K, δ): -62.50 ppm.

HRMS-EI (m/z) calculated for $C_{16}H_{12}O_1Na_1F_3Cl_1^+$ $[M+Na]^+$, 335.0421; found, 335.0421; deviation: –0.1 ppm.

Piperidin-1-yl(4-vinylphenyl)methanone (38)

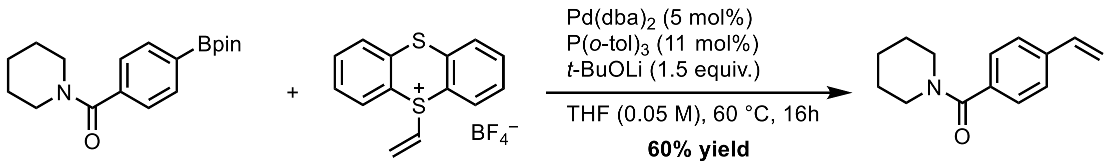

1 **38**

The title compound was prepared following general procedure B. Under ambient atmosphere, a 20 mL vial equipped with a teflon-coated magnetic stirring bar was charged with 4-(piperidine-1-carbonyl)phenylboronic acid pinacol ester (94.6 mg, 0.300 mmol, 1.00 equiv.), $Pd(dba)_2$ (8.6 mg, 15 µmol, 5.0 mol%), P(*o*-tol)$_3$ (10.0 mg, 33.0 µmol, 11.0 mol%) and *t*-BuOLi (36.0 mg, 0.450 mmol, 1.50 equiv.). The vial was transferred into a N_2-filled glove box. Subsequently, dry THF (6 mL, c = 0.05 M) was added into the vial. The reaction mixture was stirred for 2 min at 25 °C before **1** (149 mg, 0.450 mmol, 1.50 equiv.) was added into the vial in one portion. The vial was capped and was then transferred out of the glove box. The vial was placed on a heating block preheated at 60 °C where the reaction mixture was stirred for 16 h. The reaction mixture was cooled to 25 °C and filtered through a pad of celite eluting with DCM (20 mL). The filtrate was collected and concentrated under vacuum to roughly 5 mL. Silica gel (approximately 300 mg) was added, and the mixture was evaporated to dryness under reduced pressure. The residue was purified by column chromatography on silica gel eluting with a solvent mixture of EtOAc:pentane (1:4 (v:v)) to afford 38.8 mg of the title compound (**38**) as a white solid (60% yield).

R_f = 0.40 (EtOAc:pentane, 1:1 (v:v)).

NMR Spectroscopy:

^{1}H NMR (500 MHz, $CDCl_3$, 298 K, δ): 7.47 – 7.39 (m, 2H), 7.37 – 7.30 (m, 2H), 6.72 (dd, *J* = 17.6, 10.9 Hz, 1H), 5.79 (dd, *J* = 17.6, 0.8 Hz, 1H), 5.30 (dd, *J* = 10.8, 0.6 Hz, 1H), 3.75 – 3.60 (m, 2H), 3.44 – 3.27 (m, 2H), 1.74 – 1.62 (m, 4H), 1.55 – 1.42 (m, 2H) ppm.

^{13}C NMR (126 MHz, CD_3CN, 243 K, δ): 169.6, 138.6, 136.6, 136.4, 127.6, 126.5, 115.3, 48.8, 42.9, 26.5, 25.8, 24.7.

HRMS-EI (m/z) calculated for $C_{14}H_{17}N_1O_1^+$ $[M]^+$, 215.1303; found, 215.1305; deviation: –0.9 ppm.

Morpholino(3-vinylphenyl)methanone (39)

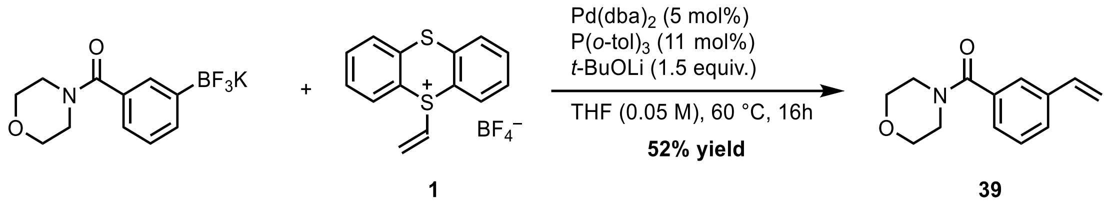

The title compound was prepared following general procedure B. Under ambient atmosphere, a 20 mL vial equipped with a teflon-coated magnetic stirring bar was charged with potassium 3-(4-morpholinylcarbonyl)phenyltrifluoroborate (89.1 mg, 0.300 mmol, 1.00 equiv.), $Pd(dba)_2$ (8.6 mg, 15 µmol, 5.0 mol%), $P(o\text{-tol})_3$ (10.0 mg, 33.0 µmol, 11.0 mol%) and *t*-BuOLi (36.0 mg, 0.450 mmol, 1.50 equiv.). The vial was transferred into a N_2-filled glove box. Subsequently, dry THF (6 mL, c = 0.05 M) was added into the vial. The reaction mixture was stirred for 2 min at 25 °C before **1** (149 mg, 0.450 mmol, 1.50 equiv.) was added into the vial in one portion. The vial was capped and was then transferred out of the glove box. The vial was placed on a heating block preheated at 60 °C where the reaction mixture was stirred for 16 h. The reaction mixture was cooled to 25 °C and filtered through a pad of celite eluting with DCM (20 mL). The filtrate was collected and concentrated under vacuum to roughly 5 mL. Silica gel (approximately 300 mg) was added, and the mixture was evaporated to dryness under reduced pressure. The residue was purified by column chromatography on silica gel eluting with pentane to afford 34.3 mg of the title compound (**39**) as a colorless oil (52% yield).

R_f = 0.23 (EtOAc:pentane, v:v (1:1)).

NMR Spectroscopy:

^{1}H NMR (500 MHz, $CDCl_3$, 298 K, δ): 7.54 – 7.43 (m, 2H), 7.36 (t, *J* = 7.6 Hz, 1H), 7.26 (dt, *J* = 7.6, 1.4 Hz, 1H), 6.71 (dd, *J* = 17.6, 10.9 Hz, 1H), 5.78 (d, *J* = 17.6 Hz, 1H), 5.31 (d, *J* = 10.9 Hz, 1H), 3.79 – 3.43 (m, 8H) ppm.

^{13}C NMR (126 MHz, $CDCl_3$, 298 K, δ): 170.4, 138.2, 136.1, 135.8, 128.8, 127.7, 126.3, 125.0, 115.3, 67.0 ppm.

HRMS-EI (m/z) calculated for $C_{13}H_{15}N_1O_2Na_1^+$ $[M+Na]^+$, 240.0997; found, 240.0995; deviation: –1.0 ppm.

(*E*)-4-(Buta-1,3-dien-1-yl)-1,1'-biphenyl (40)

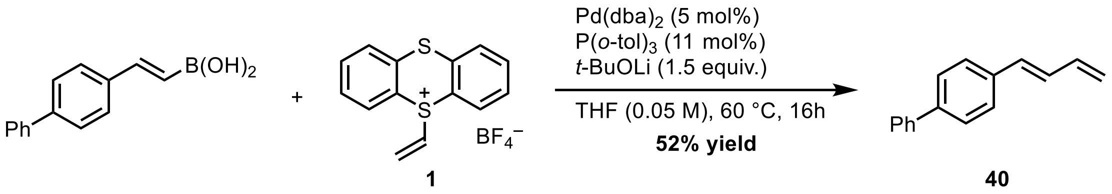

The title compound was prepared following general procedure B. Under ambient atmosphere, a 20 mL vial equipped with a teflon-coated magnetic stirring bar was charged with *trans*-2-(4-Biphenyl)vinylboronic acid

(67.2 mg, 0.300 mmol, 1.00 equiv.), $Pd(dba)_2$ (8.6 mg, 15 µmol, 5.0 mol%), $P(o\text{-tol})_3$ (10.0 mg, 33.0 µmol, 11.0 mol%) and *t*-BuOLi (36.0 mg, 0.450 mmol, 1.50 equiv.). The vial was transferred into a N_2-filled glove box. Subsequently, dry THF (6 mL, c = 0.05 M) was added into the vial. The reaction mixture was stirred for 2 min at 25 °C before **1** (149 mg, 0.450 mmol, 1.50 equiv.) was added into the vial in one portion. The vial was capped and was then transferred out of the glove box. The vial was placed on a heating block preheated at 60 °C where the reaction mixture was stirred for 16 h. The reaction mixture was cooled to 25 °C and filtered through a pad of celite eluting with DCM (20 mL). The filtrate was collected and concentrated under vacuum to roughly 5 mL. Silica gel (approximately 300 mg) was added, and the mixture was evaporated to dryness under reduced pressure. The residue was purified by column chromatography on silica gel eluting with pentane to afford 32.0 mg of the title compound (**40**) as a white solid (52% yield).

R_f = 0.20 (pentane).

NMR Spectroscopy:

^{1}H NMR (500 MHz, $CDCl_3$, 298 K, δ): 7.64 – 7.54 (m, 4H), 7.51 – 7.42 (m, 4H), 7.38 – 7.32 (m, 1H), 6.91 – 6.78 (m, 1H), 6.67 – 6.44 (m, 2H), 5.36 (dd, *J* = 17.0, 1.4 Hz, 1H), 5.20 (dd, *J* = 10.1, 1.5 Hz, 1H) ppm.

^{13}C NMR (126 MHz, $CDCl_3$, 298 K, δ): 140.8, 140.5, 137.4, 136.3, 132.5, 129.8, 128.9, 127.5, 127.4, 127.1, 127.0, 117.9 ppm.

HRMS-EI (m/z) calculated for $C_{16}H_{14}^+$ $[M]^+$, 206.1086; found, 206.1090; deviation: 2.1 ppm.

Competition experiment between 1-*d*3 and vinyl bromide.

Preparation of vinyl bromide solution in THF

A pre-weighed 10 mL argon-filled Schlenk tube with rubber septum was cooled down and kept at –40°C. To the Schlenk tube was filled with vinyl bromide through a needle from a vinyl bromide cylinder and liquid vinyl bromide condensed at the bottom of the tube. The whole tube was weighed again and the weight of vinyl bromide was the difference between two weights (in this case, 628 mg, 5.88 mmol). Then an argon-filled balloon was attached to the tube to balance pressure. THF (2.0 mL) was added into the tube via syringe to obtain a solution of vinyl bromide (2.9 M).

Competition experiment

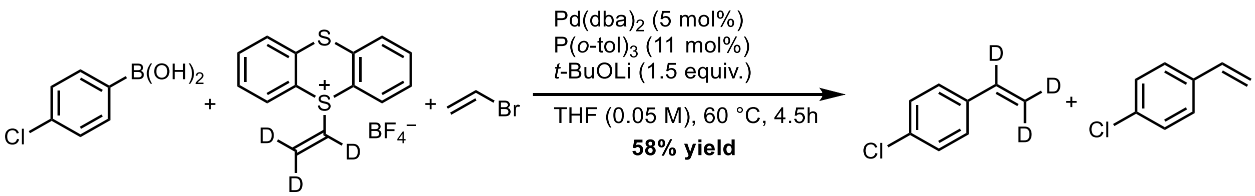

1-*d*3 **31-*d*3** **31**

Under ambient atmosphere, a 4 mL vial equipped with a teflon-coated magnetic stirring bar was charged with 4-chlorophenylboronic acid (7.8 mg, 0.050 mmol, 1.0 equiv.), $Pd(dba)_2$ (1.4 mg, 2.5 µmol, 5.0 mol%), $P(o\text{-tol})_3$

(1.7 mg, 5.5 µmol, 11 mol%) and *t*-BuOLi (6.0 mg, 0.075 mmol, 1.5 equiv.). The vial was transferred into a N_2-filled glove box. Subsequently, dry THF (0.82 mL) was added into the vial. The reaction mixture was stirred for 2 min at 25 °C before **1-*d*$_3$** (167 mg, 0.500 mmol, 10.0 equiv.) was added into the vial in one portion. The vial was capped and was then transferred out of the glove box. A solution of vinyl bromide in THF (0.18 mL, 2.9 M, 0.52 mmol, 10 equiv.) was added into the vial via syringe. The vial was placed on a heating block preheated at 60 °C where the reaction mixture was stirred for 4.5 h. The reaction mixture was cooled to 25 °C and filtered through a pad of celite eluting with DCM (10 mL). Silica gel (approximately 50 mg) was added to the filtrate, and the mixture was evaporated to dryness under reduced pressure. The residue was purified by column chromatography on silica gel eluting with pentane to afford 3.2 mg of a mixture of **31-*d*$_3$** and **31** as a colorless oil. The ratio between **31-*d*$_3$** and **31** was determined by NMR and GC-HRMS analysis.

In the ^1H NMR spectrum of the mixture, the vinyl protons of **31** cannot be observed (Figure S10).

Deuterium incorporation: >2.99 ^2H/molecule

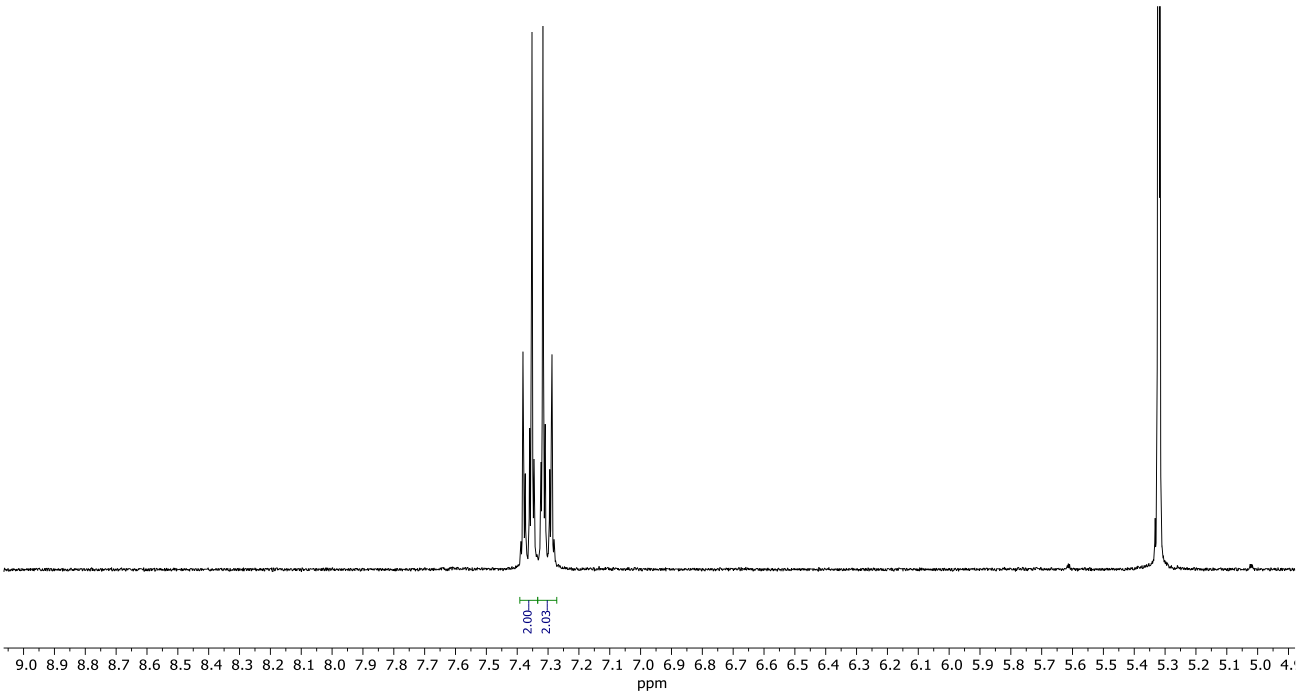

Figure S10. NMR spectrum of a mixture of **31-*d*$_3$** and **31**, CD_2Cl_2, 300 MHz, 298 K

Isotopic labeling analysis by HRMS spectrometry shows the mixture contains 99.38% of **31-*d*$_n$** (n = 1-3) and 0.61% of **31-*d*$_0$** (Figure S11). The ratio is 163:1.

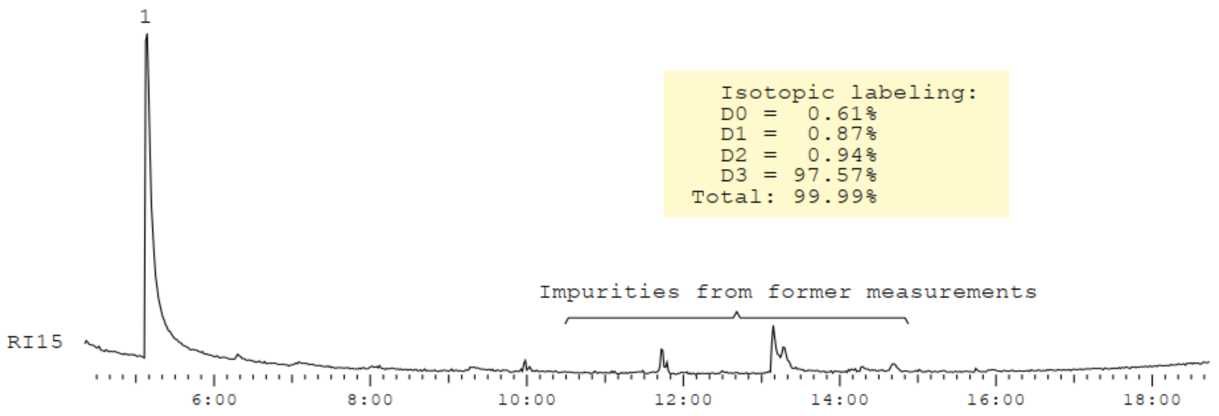

Figure S11. GC-MS isotopic labeling analysis of the mixture.

Comparison on the performance of 1 and vinyl-SPh_2(OTf) in Suzuki-type reactions.

General procedure C

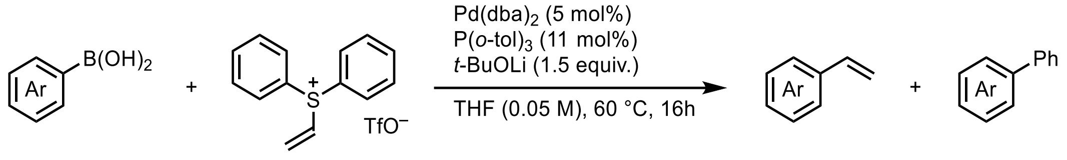

S3

Under ambient atmosphere, a 20 mL vial equipped with a teflon-coated magnetic stirring bar was charged with aryl boronic acid (0.300 mmol, 1.00 equiv.), $Pd(dba)_2$ (8.6 mg, 15 µmol, 5.0 mol%), P(*o*-tol)$_3$ (10.0 mg, 33.0 µmol, 11.0 mol%) and *t*-BuOLi (36.0 mg, 0.450 mmol, 1.50 equiv.). The vial was transferred into a N_2-filled glove box. Subsequently, dry THF (4 mL) was added into the vial. The reaction mixture was stirred for 2 min at 25 °C before a solution of vinylSPh_2(OTf) (**S3**, 163 mg, 0.450 mmol, 1.50 equiv.) in THF (2 mL) was added into the vial via syringe. The vial was capped and was then transferred out of the glove box. The vial was placed on a heating block preheated at 60 °C where the reaction mixture was stirred for 16 h. The reaction mixture was cooled to 25 °C and filtered through a pad of celite eluting with DCM (20 mL). The filtrate was collected and concentrated under vacuum to roughly 5 mL. Silica gel (approximately 300 mg) was added, and the mixture was evaporated to dryness under reduced pressure. The residue was purified by column chromatography on silica gel to give corresponding product.

tert-Butyl (3-vinylphenyl)carbamate (45)

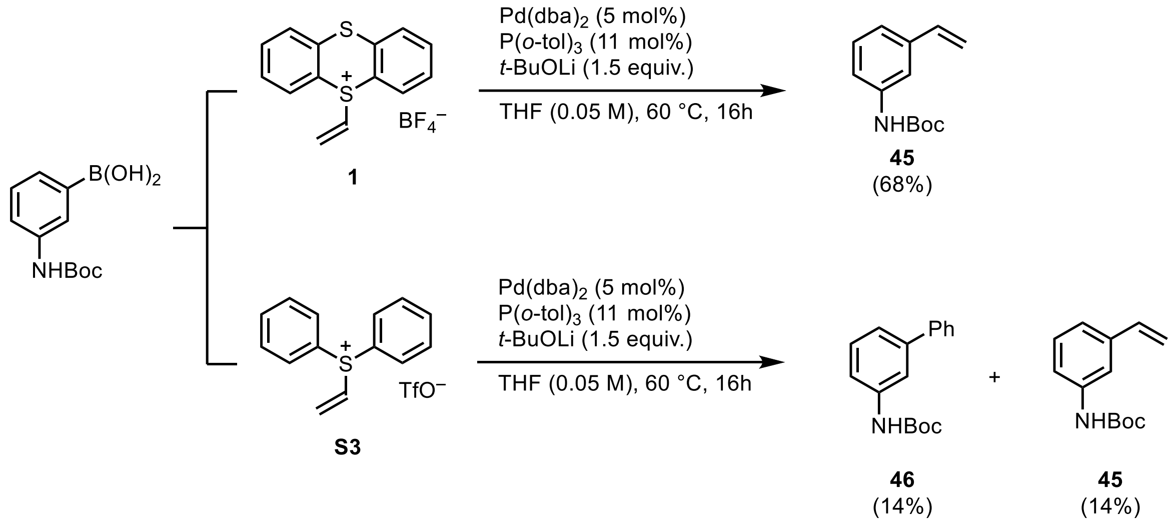

The title compound was prepared following general procedure B (for **1**) or C (for **S3**). Under ambient atmosphere, a 20 mL vial equipped with a teflon-coated magnetic stirring bar was charged with 3-(*N*-Boc-amino)phenylboronic acid (71.1 mg, 0.300 mmol, 1.00 equiv.), $Pd(dba)_2$ (8.6 mg, 15 µmol, 5.0 mol%), P(*o*-tol)$_3$ (10.0 mg, 33.0 µmol, 11.0 mol%) and *t*-BuOLi (36.0 mg, 0.450 mmol, 1.50 equiv.). The vial was transferred into a N_2-filled glove box. Subsequently, dry THF (4 mL) was added into the vial. The reaction mixture was stirred for 2 min at 25 °C before **1** (149 mg, 0.450 mmol, 1.50 equiv.) or **S3** (163 mg, 0.450 mmol, 1.50 equiv; as a solution in 2 mL of THF) were added into the vial in one portion. The vial was capped and was then transferred out of the glove box. The vial was placed on a heating block preheated at 60 °C where the reaction mixture was stirred for 16 h. The reaction mixture was cooled to 25 °C and filtered through a pad of celite eluting with DCM (20 mL). The filtrate was collected and concentrated under vacuum to roughly 5 mL. Silica gel (approximately 300 mg) was added, and the mixture was evaporated to dryness under reduced pressure. The residue was purified by column chromatography on silica gel eluting with a solvent mixture of EtOAc:pentane (1:50 (v:v)) to afford the title compound (**45**) as a colorless oil.

Yield of **45** using **1** as vinylating reagent: 44.7 mg, 68%.

Yield of **45** using **3** as vinylating reagent: 9.3 mg, 14%. In addition, arylation product **46** was obtained as a colorless oil: 11.3 mg, 14% yield.

Data for 45:

R*f* = 0.30 (EtOAc:pentane, 1:19 (v:v)).

NMR Spectroscopy:

^{1}H NMR (300 MHz, $CDCl_3$, 298 K, δ): 7.46 (s, 1H), 7.27 – 7.17 (m, 2H), 7.09 (ddd, *J* = 6.0, 3.1, 1.7 Hz, 1H), 6.68 (dd, *J* = 17.6, 10.9 Hz, 1H), 6.53 (s, 1H), 5.74 (dd, *J* = 17.6, 0.9 Hz, 1H), 5.24 (dd, *J* = 10.9, 0.9 Hz, 1H), 1.53 (s, 9H) ppm.

^{13}**C NMR** (75 MHz, $CDCl_3$, 298 K, δ): 152.9, 138.8, 138.6, 136.8, 129.2, 121.1, 118.1, 116.4, 114.4, 80.7, 28.5 ppm.

HRMS-EI (m/z) calculated for $C_{13}H_{16}N_1O_2^+$ [M]$^+$, 218.1188; found, 218.1187; deviation: -0.8 ppm.

Data for 46:

R*f* = 0.24 (EtOAc:pentane, 1:19 (v:v)).

NMR Spectroscopy:

^{1}**H NMR** (600 MHz, $CDCl_3$, 298 K, δ): 7.63 (s, 1H), 7.60 – 7.57 (m, 2H), 7.45 – 7.40 (m, 2H), 7.38 – 7.30 (m, 3H), 7.28 – 7.25 (m, 2H), 6.54 (s, 1H), 1.54 (s, 9H) ppm.

^{13}**C NMR** (151 MHz, $CDCl_3$, 298 K, δ): 153.0, 142.4, 141.1, 139.0, 129.6, 128.9, 127.6, 127.4, 122.2, 117.6, 117.5, 80.8, 28.6 ppm.

HRMS-EI (m/z) calculated for $C_{17}H_{19}N_1O_2^+$ [M]$^+$, 269.1409; found, 269.1410; deviation: +0.3 ppm.

1-Fluoro-4-vinylbenzene (42)

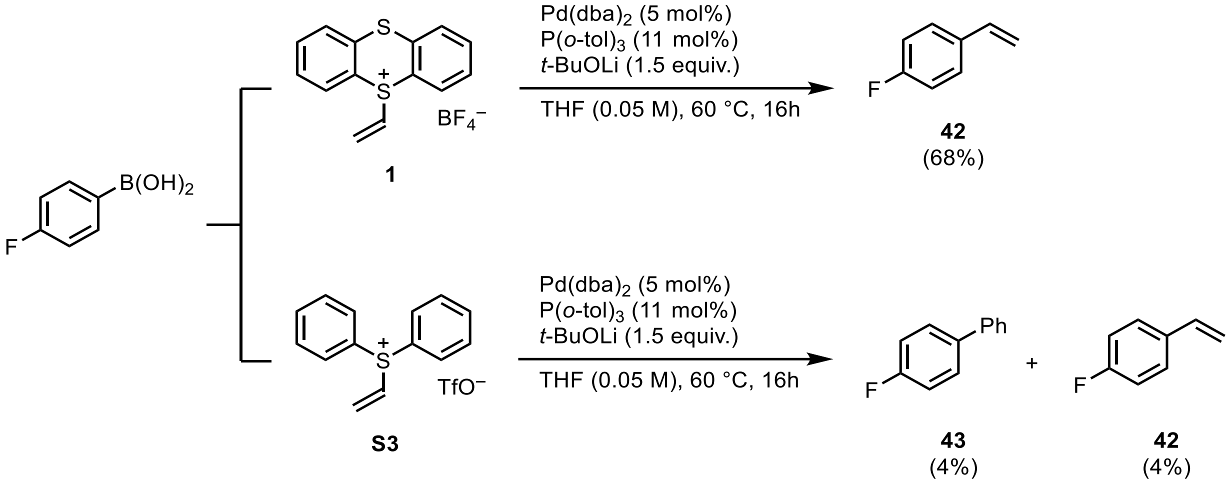

The title compound was prepared following general procedure B (for **1**) or C (for **S3**). Under ambient atmosphere, a 4 mL vial equipped with a teflon-coated magnetic stirring bar was charged with (4-fluorophenyl)boronic acid (7.0 mg, 0.050 mmol, 1.0 equiv.), $Pd(dba)_2$ (1.4 mg, 2.5 µmol, 5.0 mol%), P(*o*-tol)$_3$ (1.7 mg, 5.5 µmol, 11 mol%) and *t*-BuOLi (6.0 mg, 0.075 mmol, 1.5 equiv.). The vial was transferred into a N_2-filled glove box. Subsequently, dry THF (1 mL) was added into the vial. The reaction mixture was stirred for 2 min at 25 °C before **1** (25 mg, 0.075 mmol, 1.5 equiv.) was added into the vial in one portion. The vial was capped and was then transferred out of the glove box. The vial was placed on a heating block preheated at 60 °C where the reaction mixture was stirred for 16 h. The reaction mixture was cooled to 25 °C. To the cooled reaction mixture was added 4-fluorobenzotrifluoride (12.7 µL, 16.4 mg, 0.10 mmol, 2.0 equiv.) as internal standard. The ^{19}F NMR resonance of the product at -114.6 ppm was integrated relative to the ^{19}F NMR resonances of the aromatic fluorine atom of 4-fluorobenzotrifluoride (δ = -107.6 ppm).

The scale of using **S3** as vinylating reagent was doubled to 0.10 mmol. In this case, 0.10 mmol (1.0 equiv.) of 4-fluorobenzotrifluoride was added as internal standard.

Yield of **42** using **1** as vinylating reagent: 68%. Reaction run at 1.00 mmol scale: 161 mg, 74%.

Yield of **42** using **S3** as vinylating reagent: 4%. In addition, arylation product **43** was obtained in 4% yield.

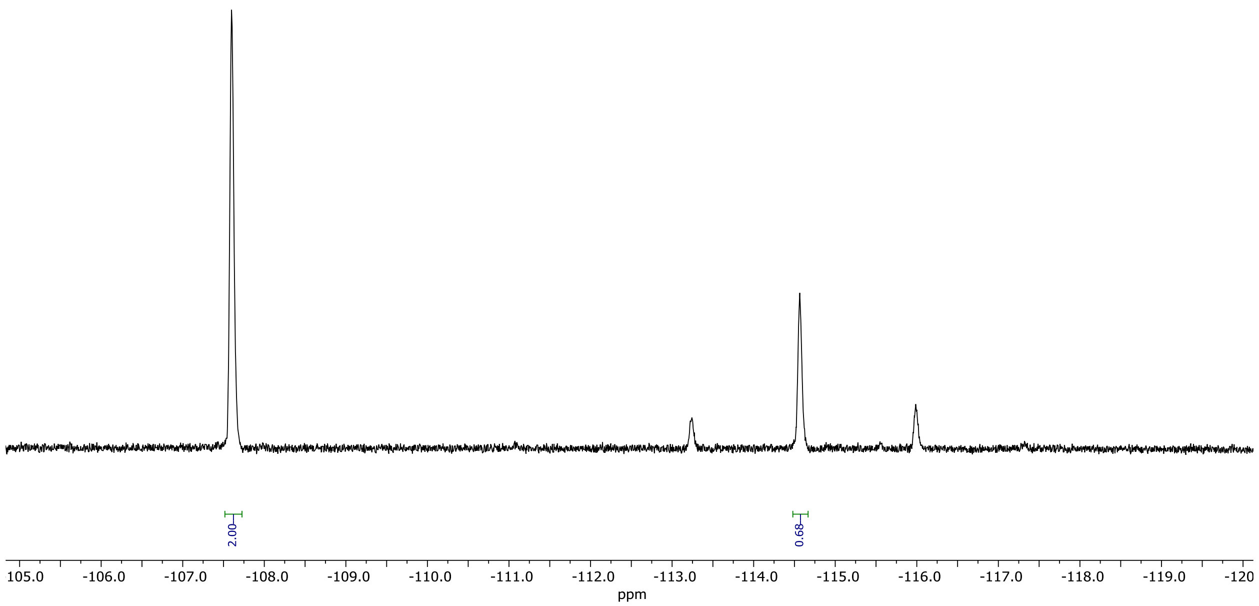

Figure S12. ^{19}F NMR spectrum of the crude mixture of the Suzuki-type vinylation of (4-fluorophenyl)boronic acid with **1**, THF, 471 MHz, 298 K.

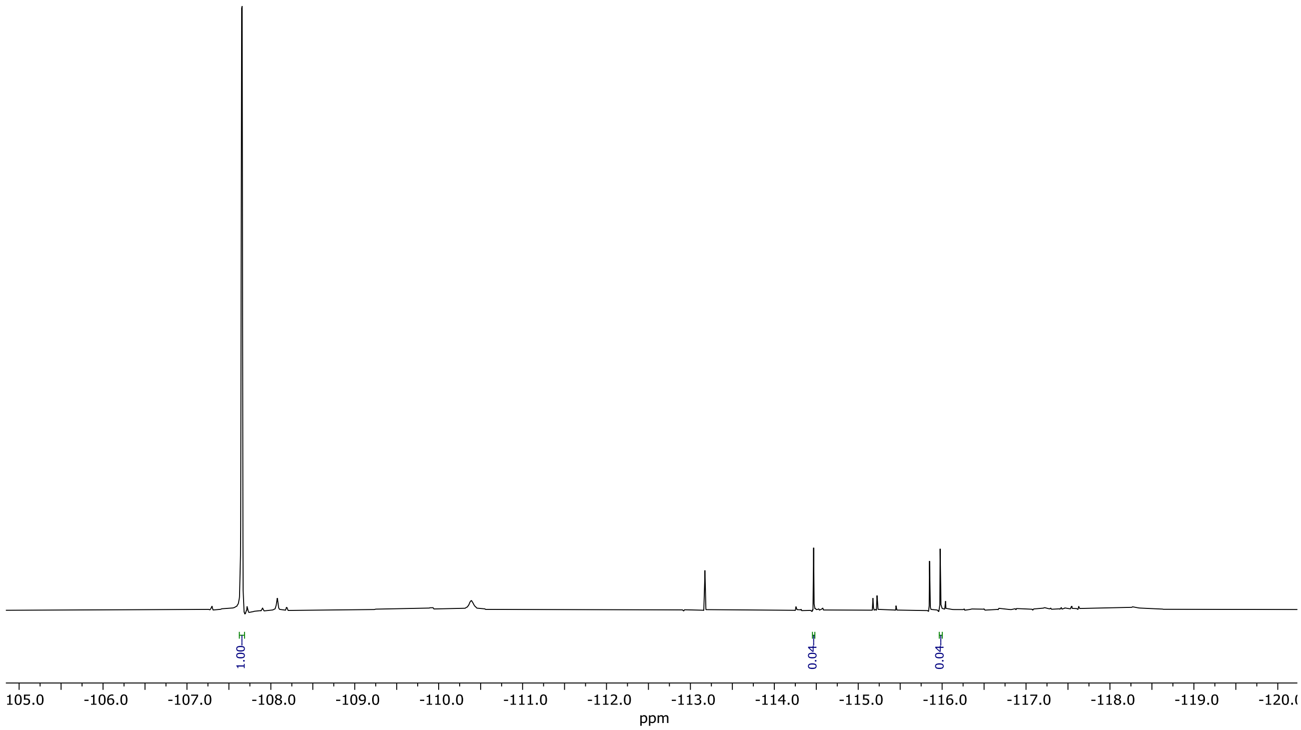

Figure S13. ^{19}F NMR spectrum of the crude mixture of the Suzuki-type vinylation of (4-fluorophenyl)boronic acid with **S3**, THF, 282 MHz, 298 K.

Discussion

In stark difference to the direct nucleophilic substitution on thianthrenium salts, which often result in the endo C–S bond cleavage and ring-opening of the thianthrene core,[6,12] palladium-catalyzed reactions on these substrates generally result in the exo C–S bond cleavage.[1,13-16] This outcome may be rationalized considering that oxidative addition into the endocyclic C–S bond may be reversible.[17-18] This feature efficiently channels the reaction towards potentially irreversible cleavage of the exocyclic C(vinyl)–S bond in reactions with **1**. In contrast, the reaction based on vinyl-SPh_2(OTf) does not benefit from a similar behavior, which may the reason for the unselective C(vinyl)–S vs C(phenyl)–S bond cleavage under the same reaction conditions.

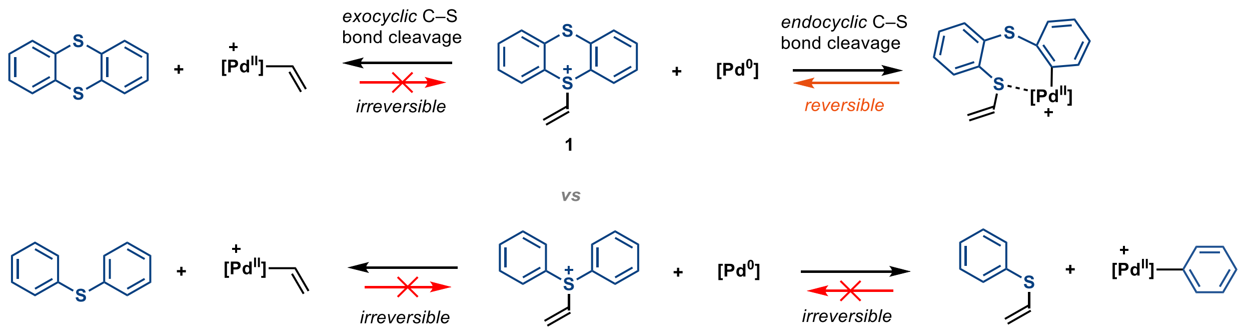

In order to rule out the formation of C-Ph products arising from an alternative pathway involving oxidative addition of Ph_2S into Pd(0) we set up the following experiments, described in the figure below:

(1) 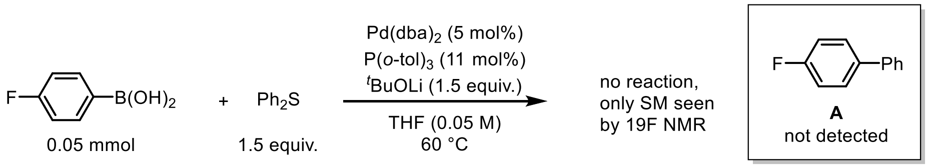

(2) 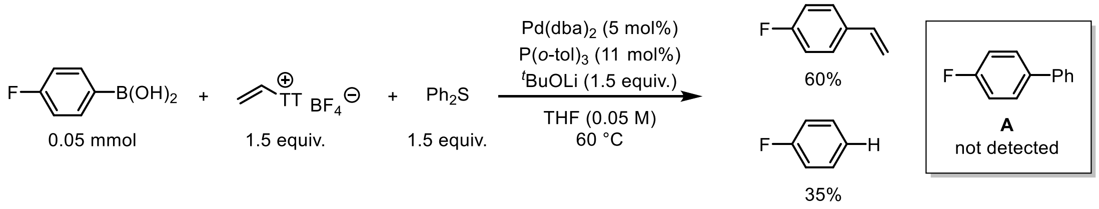

To assess the ability of Ph_2S to undergo oxidative addition to Pd(0) under our reaction conditions, we evaluated this possibility in reaction 1. Under the same conditions, the substitution of sulfonium salts by Ph_2S did not resulted in the formation of product A and only boronic acid starting material was detected. Moreover, we run our reaction using vinyl-TT in presence of equimolar amounts of Ph_2S (reaction 2), but could not detect product A either. All observations are fully consistent with the original claims.

In addition, we also monitored the reaction profile of the reaction with vinyl-SPh_2(OTf), which can be found in the following Figure:

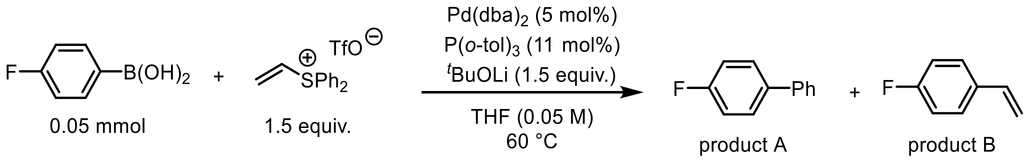

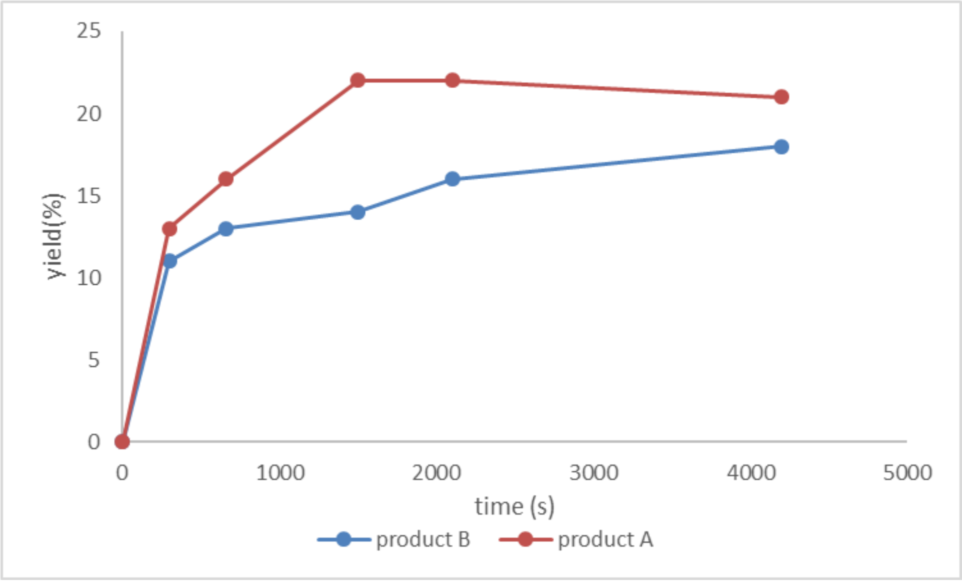

Our data shows that product A is formed simultaneously to product B since the beginning of the reaction, pointing against the formation of Ph2S and subsequent oxidative addition to afford product A.

Collectively, these data suggest that product A arises from Cphenyl–S oxidative addition on vinyl-SPh2(OTf) rather than oxidative addition on Ph2S.

X-RAY CRYSTALLOGRAPHIC ANALYSIS

S-vinyl-thianthrenium tetrafluoroborate (vinyl-TT$^+$, 1) (CCDC 2075820)

Experimental

Vinyl-TT$^+$ (**1**) was crystallized from a dichloromethane/diethyl ether. The atoms are depicted with 50% probability ellipsoids. The crystallographic data are summarized in the following table.

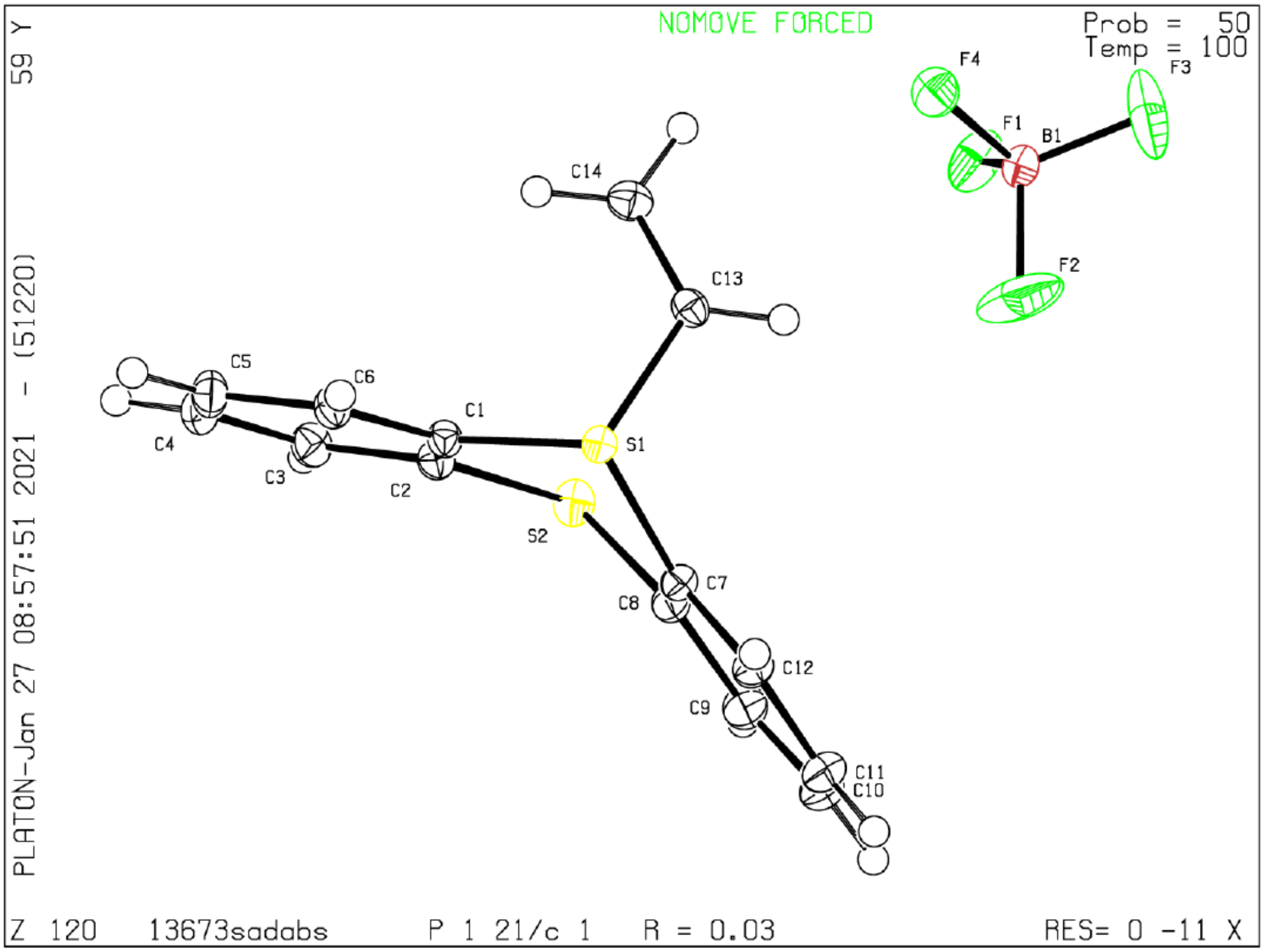

Figure S14. X-ray structure of vinyl-TT$^+$ (**1**).

Table S4. Crystal data and structure refinement.

Empirical formula	$C_{14} H_{11} B F_4 S_2$
Color	colourless
Formula weight	330.16 g · mol^{-1}

Temperature	100(2) K
Wavelength	0.71073 Å
Crystal system	MONOCLINIC
Space group	**$P2_1/c$, (no. 14)**
Unit cell dimensions	a = 5.4988(4) Å α= 90°.
	b = 18.2398(8) Å β= 99.116(7)°.
	c = 14.4633(12) Å γ= 90°.
Volume	1432.30(17) $Å^3$
Z	4
Density (calculated)	1.531 Mg · m^{-3}
Absorption coefficient	0.403 mm^{-1}
F(000)	672 e
Crystal size	0.18 x 0.17 x 0.13 mm^3
θ range for data collection	2.650 to 36.023°.
Index ranges	$-9 \leq h \leq 9$, $-30 \leq k \leq 30$, $-23 \leq l \leq 23$
Reflections collected	50539
Independent reflections	6779 [R_{int} = 0.0438]
Reflections with I>2σ (I)	5582
Completeness to θ = 25.242°	99.8 %
Absorption correction	Gaussian
Max. and min. transmission	0.96 and 0.91
Refinement method	Full-matrix least-squares on F^2
Data / restraints / parameters	6779 / 0 / 202
Goodness-of-fit on F^2	1.040
Final R indices [I>2□(I)]	R_1 = 0.0324 wR^2 = 0.0793
R indices (all data)	R_1 = 0.0441 wR^2 = 0.0845
Largest diff. peak and hole	1.0 and -0.5 e · $Å^{-3}$

Table S5. Bond lengths [Å] and angles [°]

S(1)-C(1)	1.7635(9)	S(1)-C(7)	1.7718(9)
S(1)-C(13)	1.7857(9)	S(2)-C(2)	1.7613(9)
S(2)-C(8)	1.7634(10)	C(1)-C(2)	1.3996(12)
C(1)-C(6)	1.3950(12)	C(2)-C(3)	1.3989(13)
C(3)-C(4)	1.3909(14)	C(4)-C(5)	1.3905(14)
C(5)-C(6)	1.3890(13)	C(7)-C(8)	1.3972(12)
C(7)-C(12)	1.3965(13)	C(8)-C(9)	1.4012(13)
C(9)-C(10)	1.3890(16)	C(10)-C(11)	1.3929(17)
C(11)-C(12)	1.3907(14)	C(13)-H(13)	0.945(16)
C(13)-C(14)	1.3176(13)	C(14)-H(14A)	0.961(16)
C(14)-H(14B)	0.941(15)	F(1)-B(1)	1.3924(12)
F(2)-B(1)	1.3804(13)	F(3)-B(1)	1.3766(13)
F(4)-B(1)	1.3920(12)		
C(1)-S(1)-C(7)	102.27(4)	C(1)-S(1)-C(13)	103.57(4)
C(7)-S(1)-C(13)	101.29(4)	C(2)-S(2)-C(8)	102.62(4)
C(2)-C(1)-S(1)	121.42(7)	C(6)-C(1)-S(1)	116.80(6)
C(6)-C(1)-C(2)	121.73(8)	C(1)-C(2)-S(2)	123.47(7)
C(3)-C(2)-S(2)	118.13(7)	C(3)-C(2)-C(1)	118.39(8)

C(4)-C(3)-C(2)	120.04(8)	C(5)-C(4)-C(3)	120.78(9)
C(6)-C(5)-C(4)	120.12(9)	C(5)-C(6)-C(1)	118.90(8)
C(8)-C(7)-S(1)	120.94(7)	C(12)-C(7)-S(1)	117.21(7)
C(12)-C(7)-C(8)	121.82(8)	C(7)-C(8)-S(2)	123.80(7)
C(7)-C(8)-C(9)	118.27(9)	C(9)-C(8)-S(2)	117.92(7)
C(10)-C(9)-C(8)	120.19(10)	C(9)-C(10)-C(11)	120.74(9)
C(12)-C(11)-C(10)	120.00(10)	C(11)-C(12)-C(7)	118.89(9)
S(1)-C(13)-H(13)	112.2(10)	C(14)-C(13)-S(1)	122.05(7)
C(14)-C(13)-H(13)	125.6(10)	C(13)-C(14)-H(14A)	119.6(9)
C(13)-C(14)-H(14B)	123.4(9)	H(14A)-C(14)-H(14B)	117.0(13)
F(2)-B(1)-F(1)	108.18(9)	F(2)-B(1)-F(4)	109.52(9)
F(3)-B(1)-F(1)	109.33(9)	F(3)-B(1)-F(2)	110.56(10)
F(3)-B(1)-F(4)	109.94(8)	F(4)-B(1)-F(1)	109.28(8)

[4+2] cycloadduct intermediate 3 (CCDC 2075821)

Experimental

[4+2] cycloadduct intermediate **3** was crystallized from acetone/diethyl ether. The atoms are depicted with 50% probability ellipsoids. The crystallographic data are summarized in the following table.

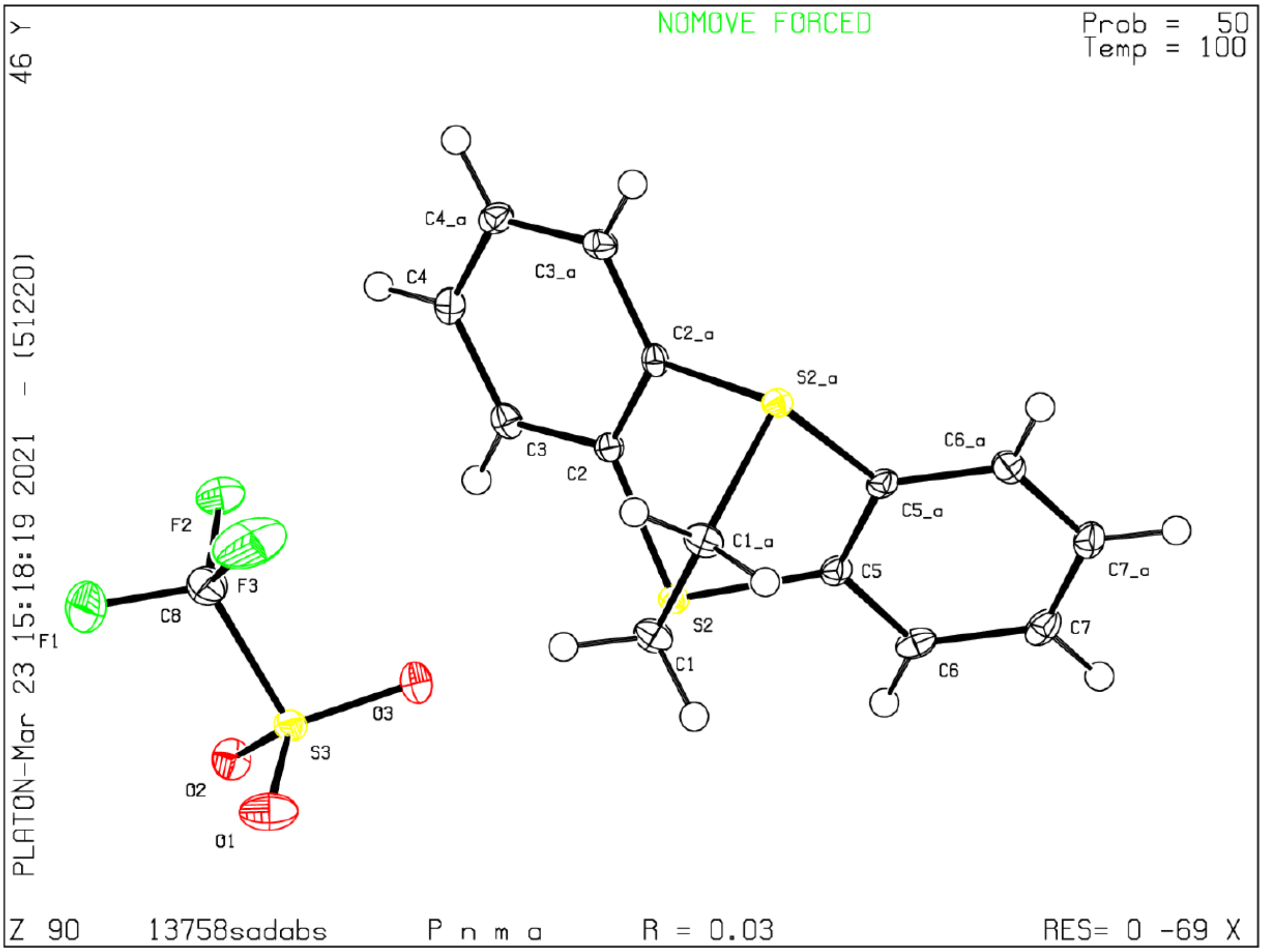

Figure S15. X-ray structure of [4+2] cycloadduct intermediate **3**.

Table S6. Crystal data and structure refinement.

Empirical formula	$C_{16} H_{12} F_6 O_6 S_4$	
Color	colourless	
Formula weight	542.50 g · mol^{-1}	
Temperature	100(2) K	
Wavelength	1.54178 Å	
Crystal system	ORTHORHOMBIC	
Space group	**Pnma, (no. 62)**	
Unit cell dimensions	a = 6.6681(2) Å	α= 90°.
	b = 14.5847(5) Å	β= 90°.
	c = 21.1524(7) Å	γ= 90°.
Volume	2057.12(12) Å^3	
Z	4	
Density (calculated)	1.752 Mg · m^{-3}	
Absorption coefficient	5.083 mm^{-1}	
F(000)	1096 e	
Crystal size	0.261 x 0.090 x 0.040 mm^3	
θ range for data collection	3.681 to 72.123°.	
Index ranges	$-7 \le h \le 8$, $-17\le k \le 17$, $-26 \le l \le 26$	
Reflections collected	61680	
Independent reflections	2096 [R_{int} = 0.0788]	
Reflections with I>2σ(I)	1790	
Completeness to θ = 67.679°	99.9 %	
Absorption correction	Gaussian	
Max. and min. transmission	0.89 and 0.63	
Refinement method	Full-matrix least-squares on F^2	
Data / restraints / parameters	2096 / 0 / 145	
Goodness-of-fit on F^2	1.159	
Final R indices [I>2σ(I)]	R_1 = 0.0331	wR^2 = 0.0915
R indices (all data)	R_1 = 0.0447	wR^2 = 0.0999
Largest diff. peak and hole	0.5 and -0.6 e · Å^{-3}	

Table S7. Bond lengths [Å] and angles [°]

S(1)-C(1)	1.828(2)	S(1)-C(2)	1.7762(19)
S(1)-C(5)	1.7766(19)	C(1)-C(1)*	1.526(4)
C(2)-C(2)*	1.391(4)	C(2)-C(3)	1.387(3)
C(3)-C(4)	1.391(3)	C(4)-C(4)*	1.398(4)
C(5)-C(5)*	1.392(4)	C(5)-C(6)	1.384(3)
C(6)-C(7)	1.391(3)	C(7)-C(7)*	1.390(4)
S(2)-O(1)	1.4367(17)	S(2)-O(2)	1.4478(14)
S(2)-O(3)	1.4385(16)	S(2)-C(8)	1.822(2)
F(1)-C(8)	1.329(3)	F(2)-C(8)	1.334(3)
F(3)-C(8)	1.326(3)		

C(2)-S(1)-C(1)	97.93(9)	C(2)-S(1)-C(5)	103.06(9)
C(5)-S(1)-C(1)	97.03(9)	C(1)*-C(1)-S(1)	116.03(6)
C(2)*-C(2)-S(1)	119.32(6)	C(3)-C(2)-S(1)	119.41(15)
C(3)-C(2)-C(2)*	121.02(12)	C(2)-C(3)-C(4)	118.22(19)
C(3)-C(4)-C(4)*	120.75(12)	C(5)*-C(5)-S(1)	119.29(7)
C(6)-C(5)-S(1)	119.51(16)	C(6)-C(5)-C(5)*	120.93(12)
C(5)-C(6)-C(7)	118.3(2)	C(7)*-C(7)-C(6)	120.82(12)
O(1)-S(2)-O(2)	114.06(9)	O(1)-S(2)-O(3)	115.79(10)
O(1)-S(2)-C(8)	104.17(10)	O(2)-S(2)-C(8)	103.44(9)
O(3)-S(2)-O(2)	114.09(10)	O(3)-S(2)-C(8)	103.19(10)
F(1)-C(8)-S(2)	111.68(14)	F(1)-C(8)-F(2)	107.09(17)
F(2)-C(8)-S(2)	110.82(15)	F(3)-C(8)-S(2)	110.99(15)
F(3)-C(8)-F(1)	108.36(19)	F(3)-C(8)-F(2)	107.73(18)

Symmetry transformations used to generate equivalent atoms: * x,-y+3/2,z

SPECTROSCOPIC DATA

^{1}H NMR of vinyl TT$^+$ (1)

CD_3CN, 298 K

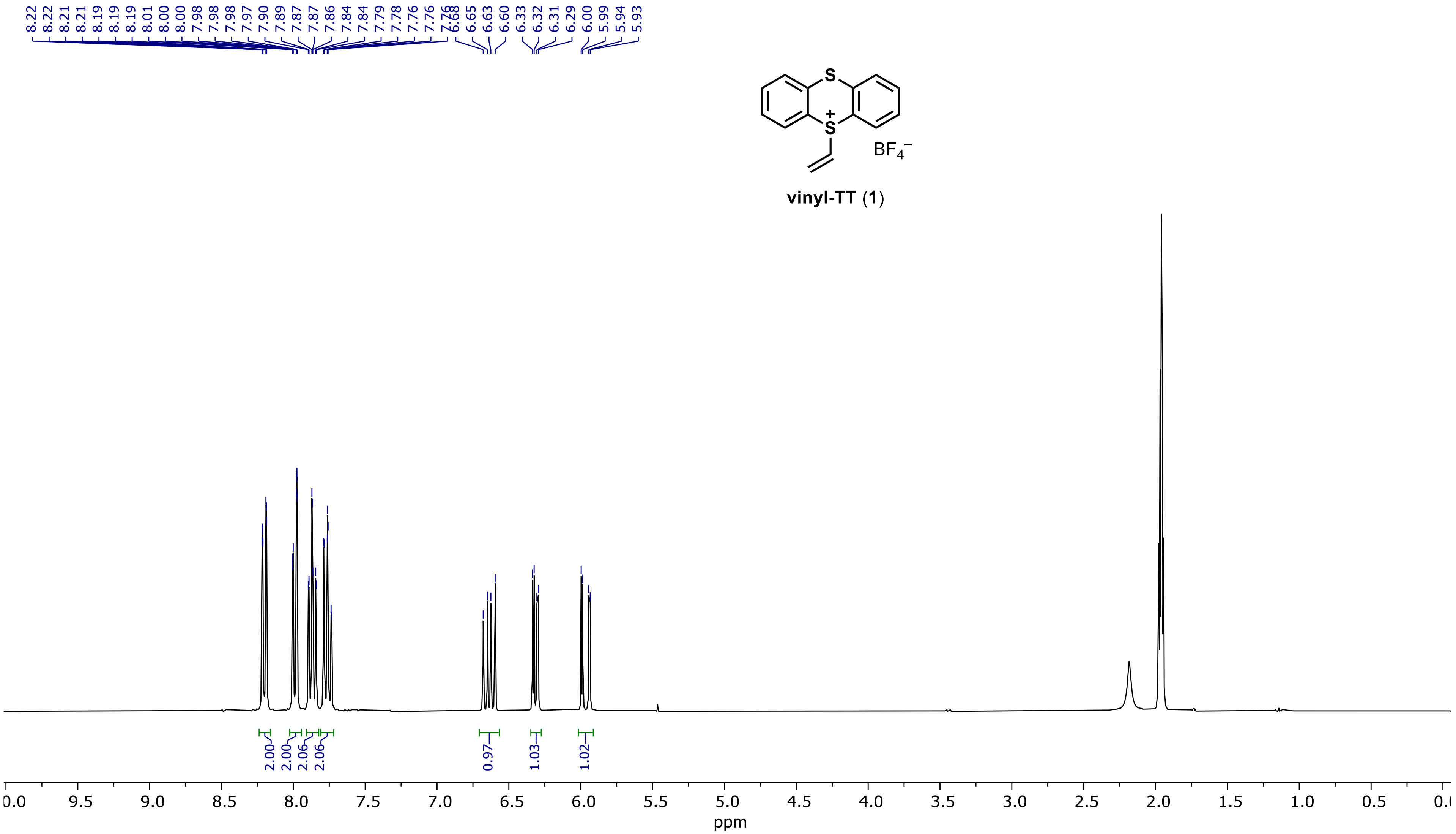

^{13}C NMR of vinyl TT$^+$ (1)

CD_3CN, 298 K

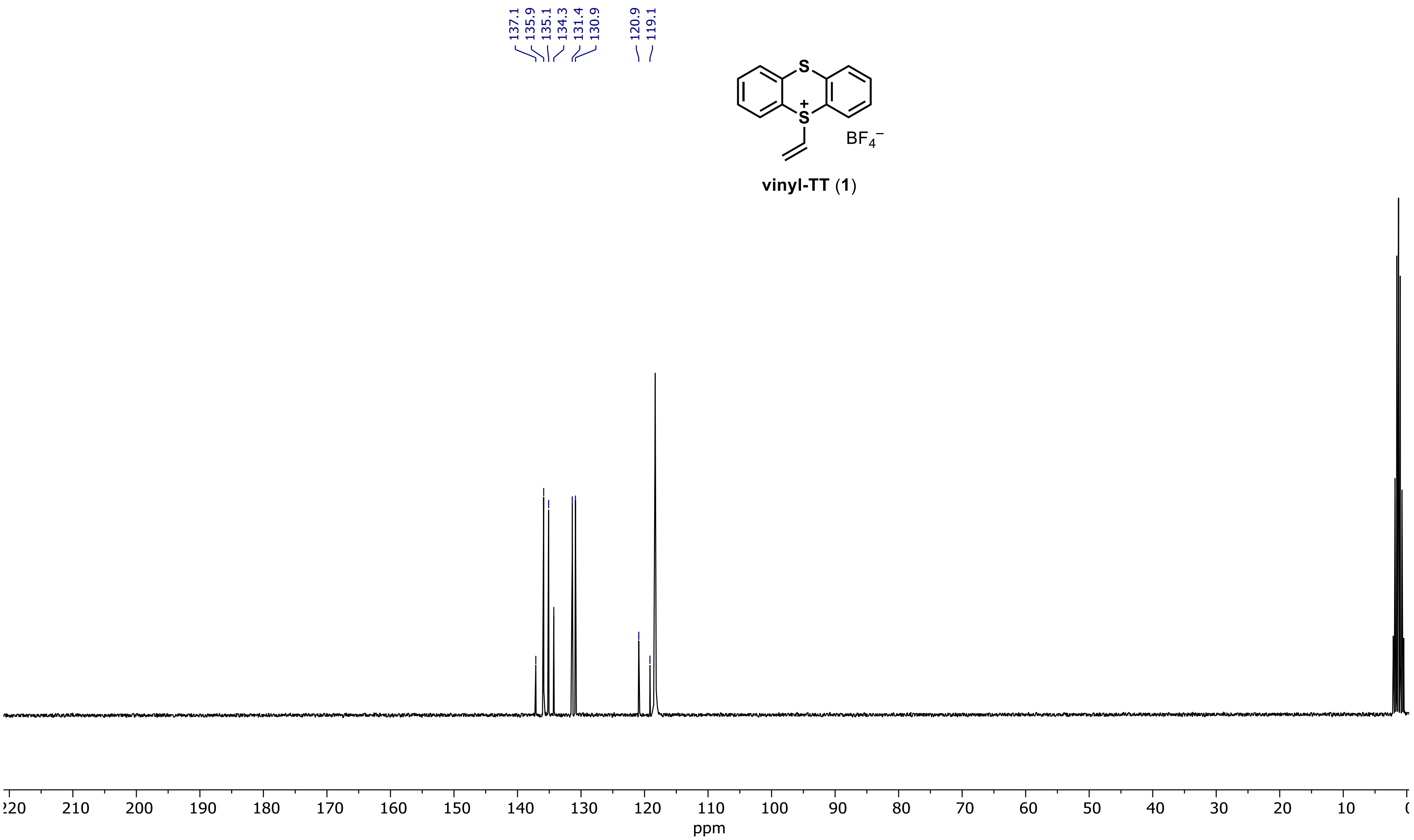

^{19}F NMR of vinyl TT^{+} (1)

CD_3CN, 298 K

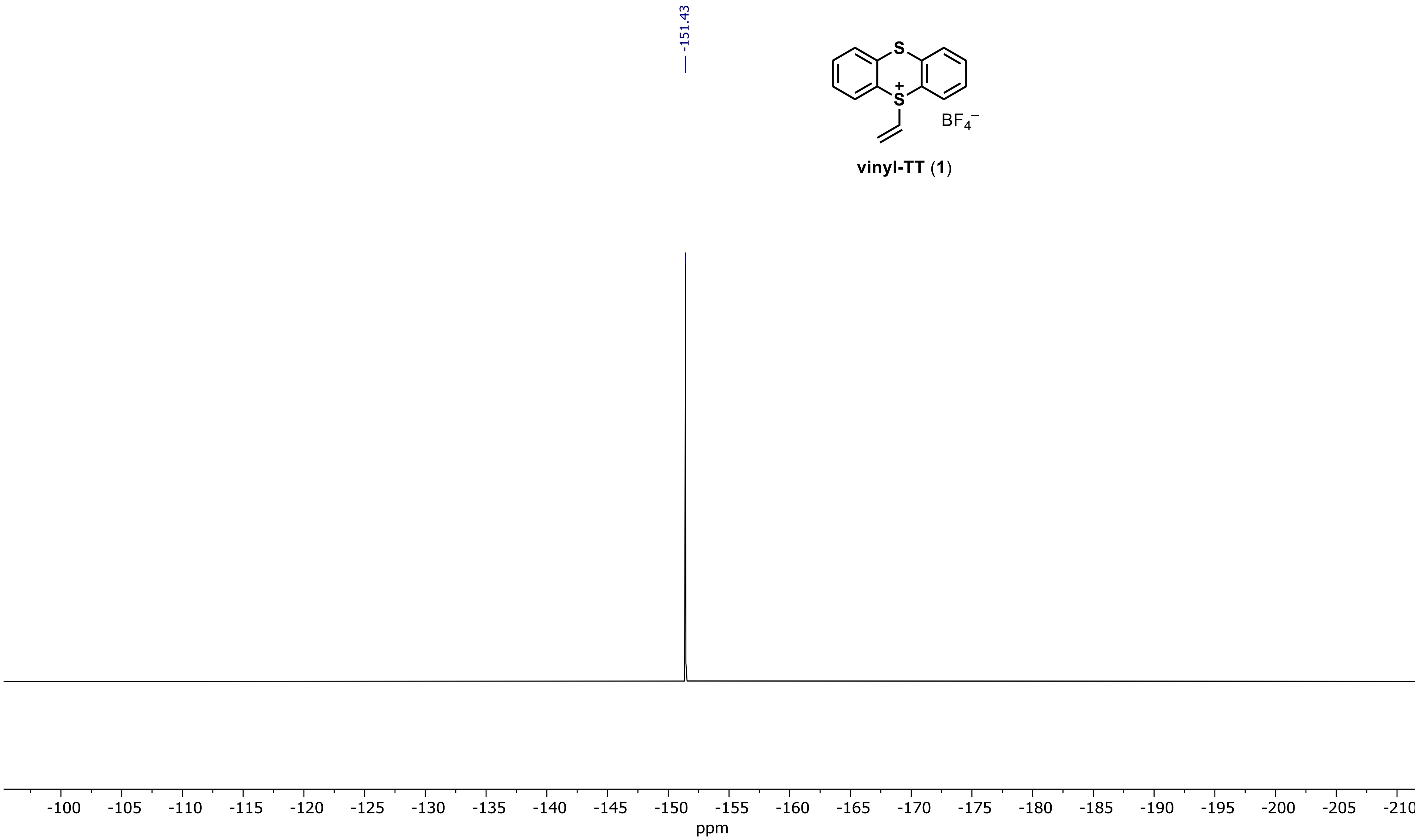

^{1}H NMR of vinyl TT^{+}-d_3 (1-d_3)

CD_2Cl_2, 298 K

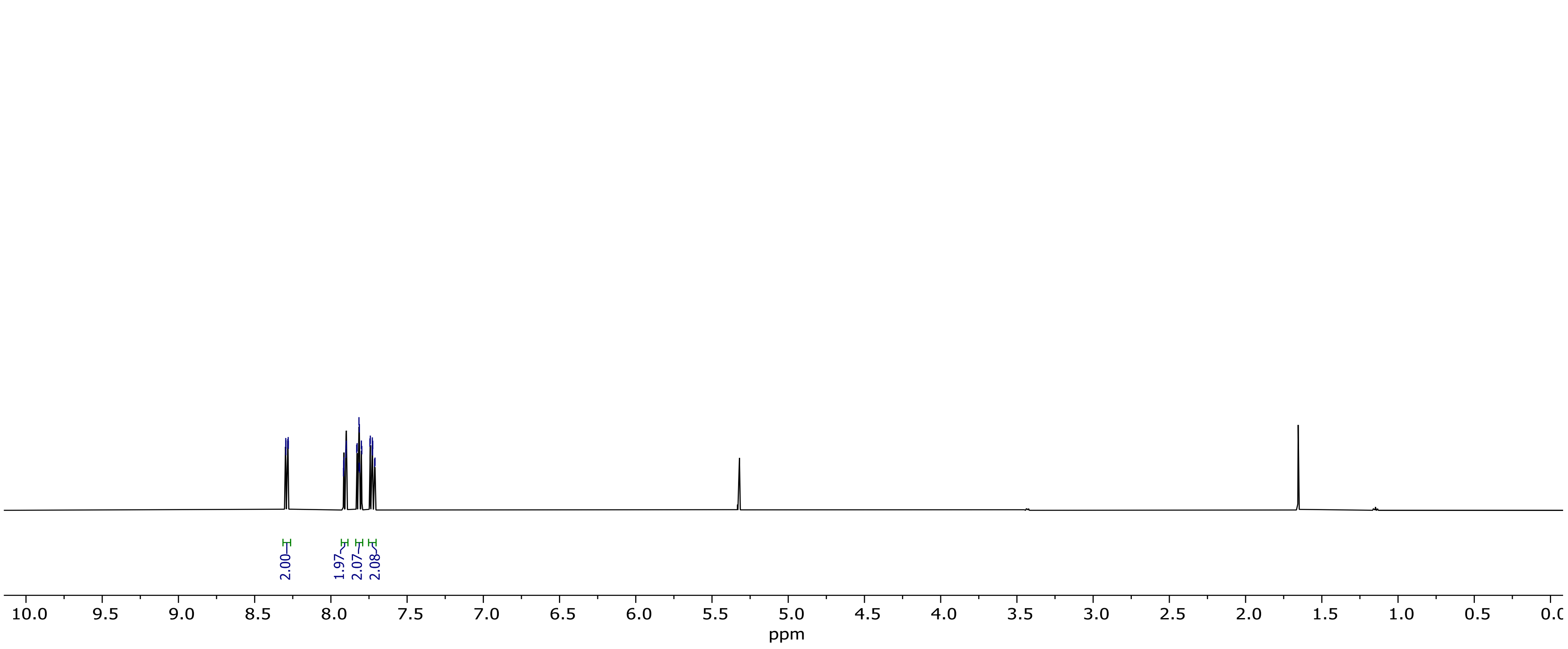

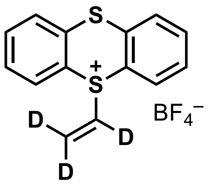

vinyl-TT^{+}-d_3 (1-d_3)

^{2}H NMR of vinyl TT^{+}-d_3 (1-d_3)

CD_2Cl_2, 298 K

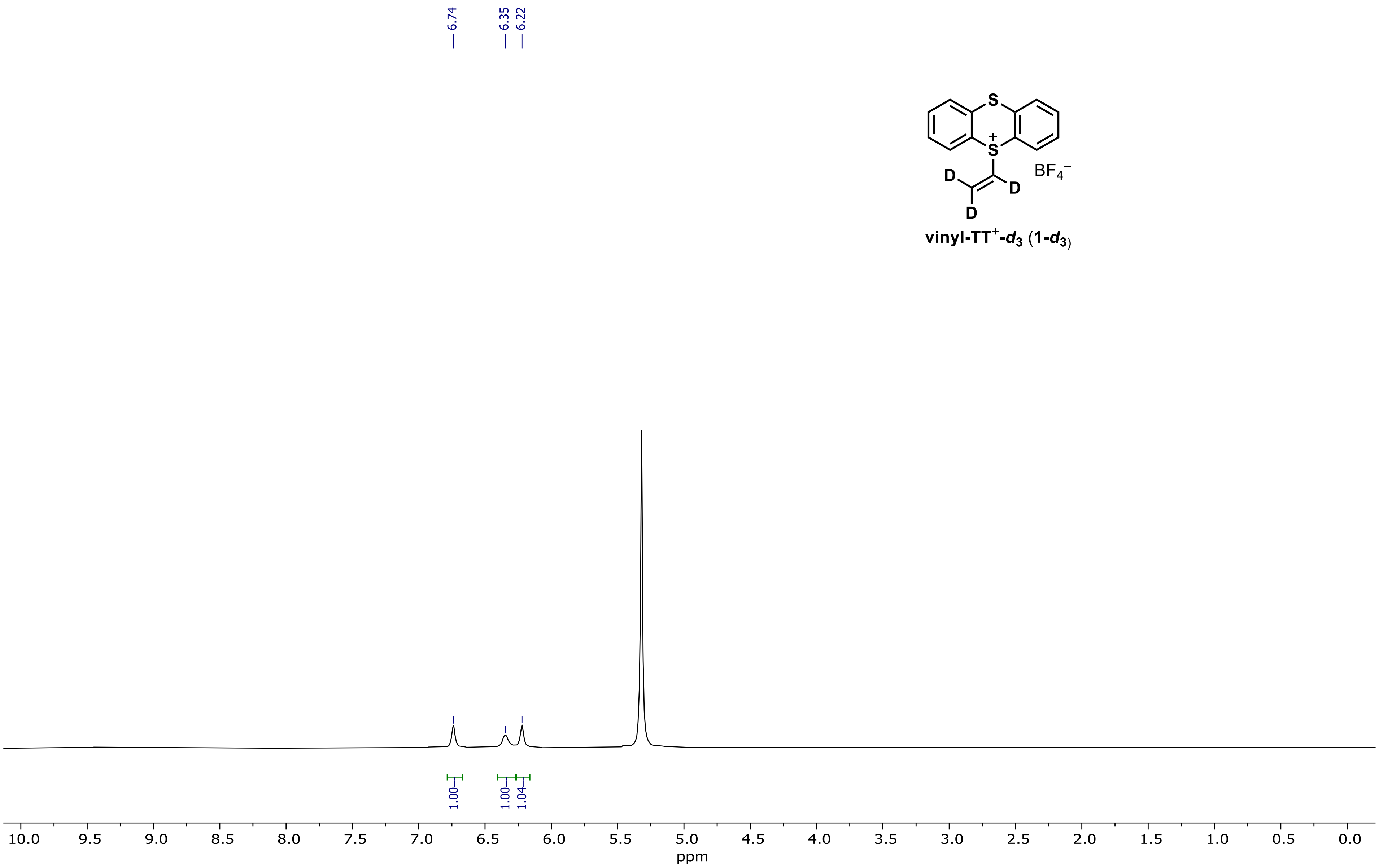

vinyl-TT^{+}-d_3 (1-d_3)

^{13}C NMR of vinyl TT$^+$-d_3 (1-d_3)

CD_2Cl_2, 298 K

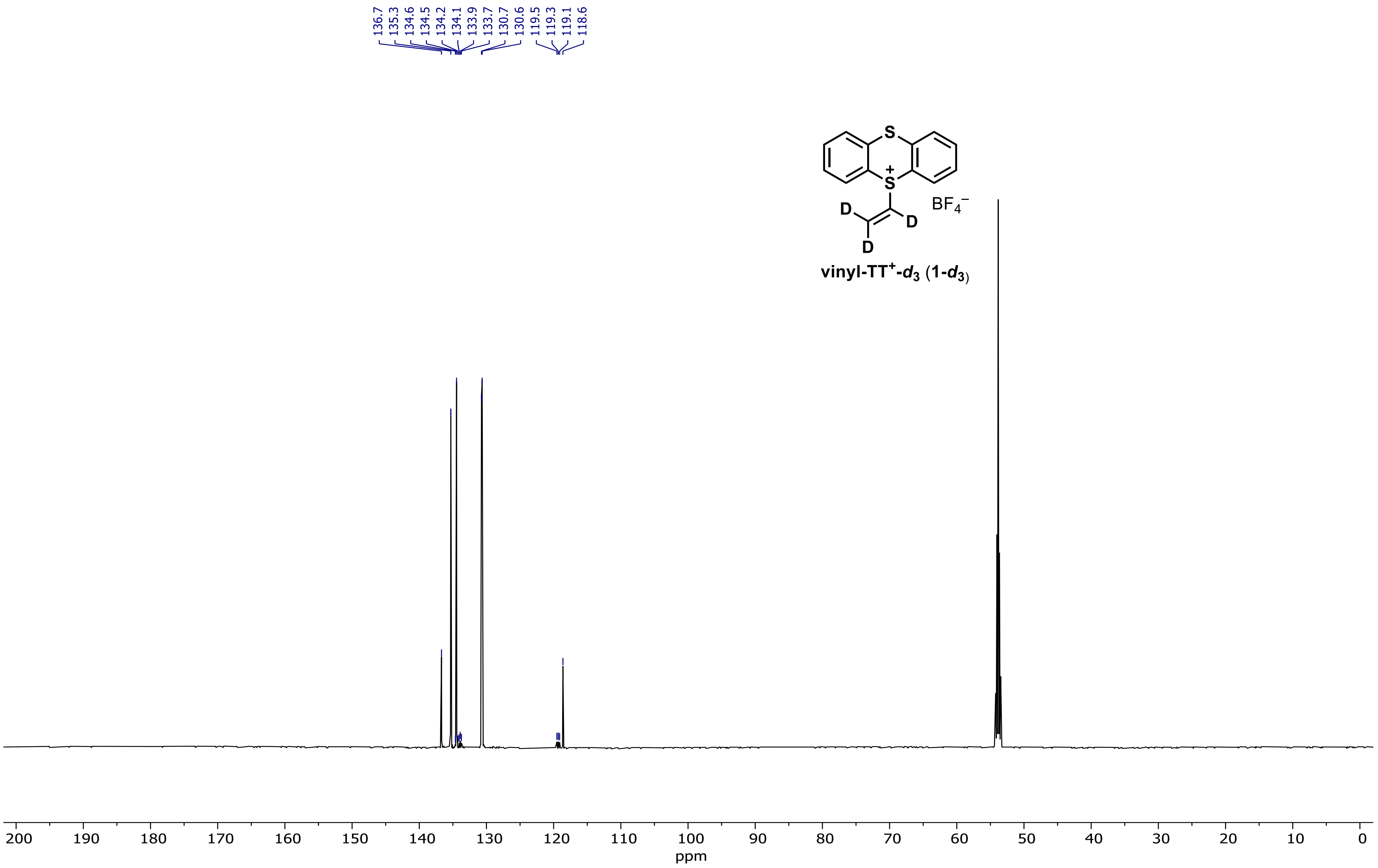

^{19}F NMR of vinyl TT^{+}-d_3 (1-d_3)

CD_2Cl_2, 298 K

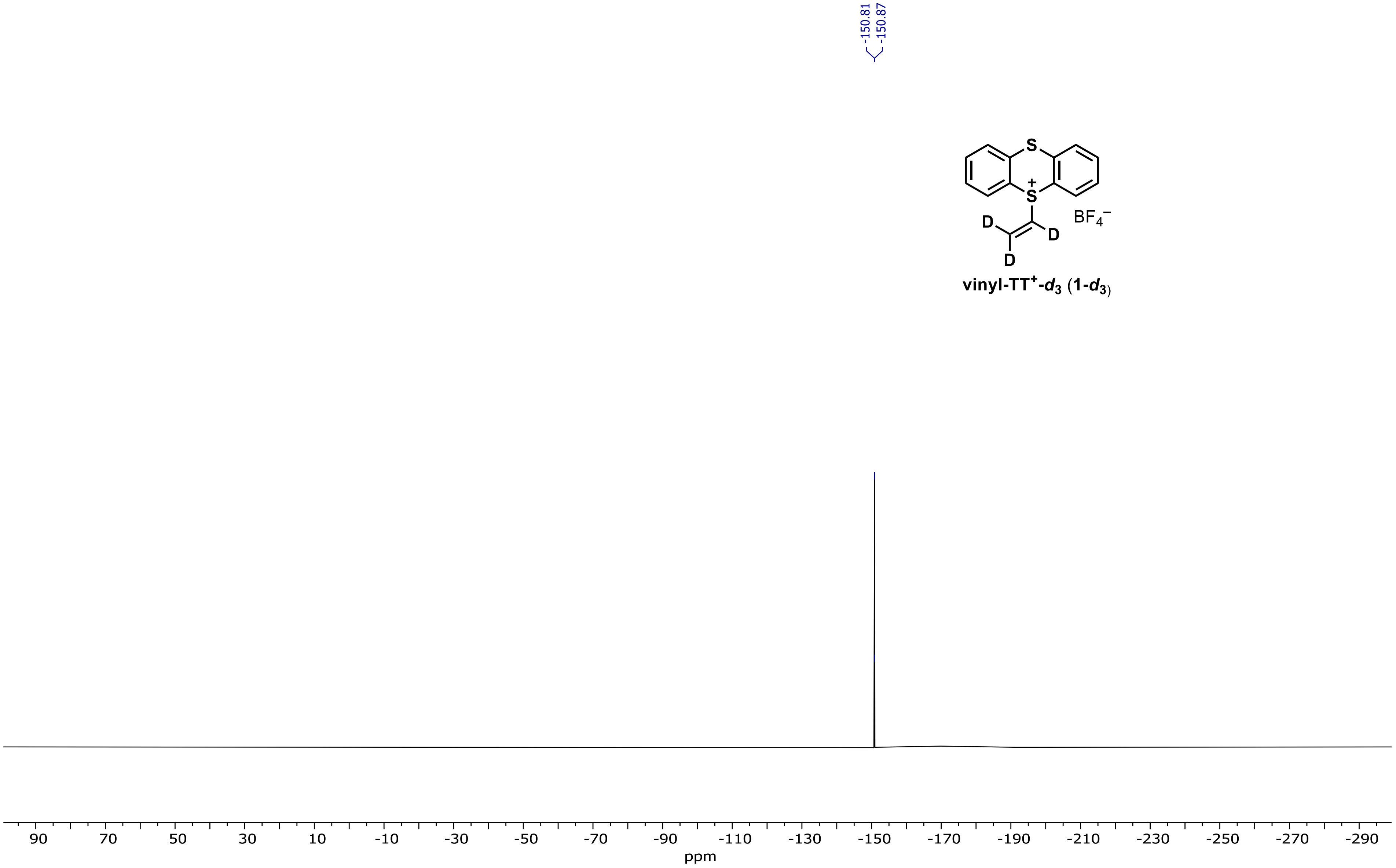

^{1}H NMR of the [4+2] cycloadduct intermediate 3

CD_3CN, 23 ºC

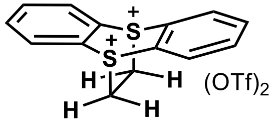

3

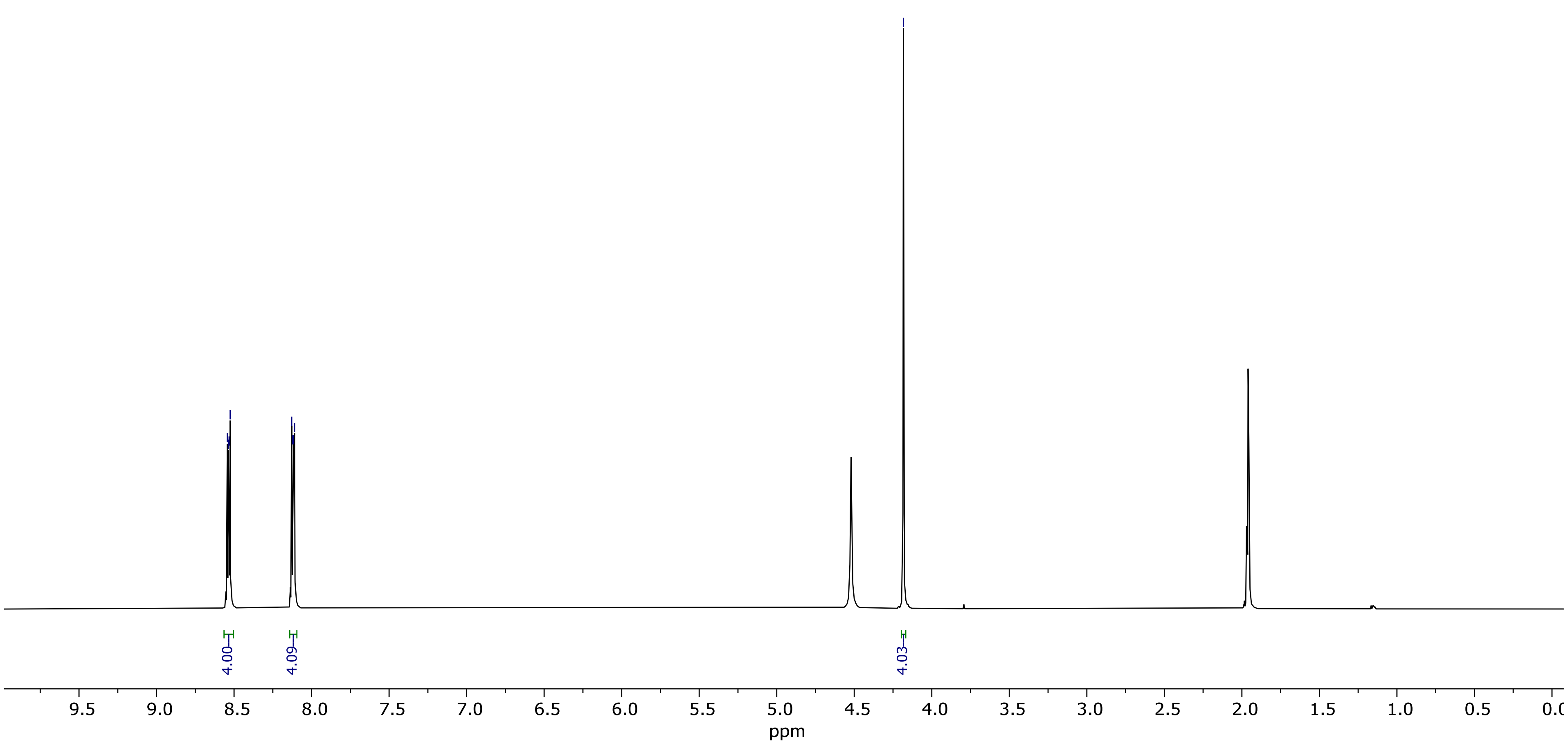

^{13}C NMR of the [4+2] cycloadduct intermediate 3

CD_3CN, 23 ºC

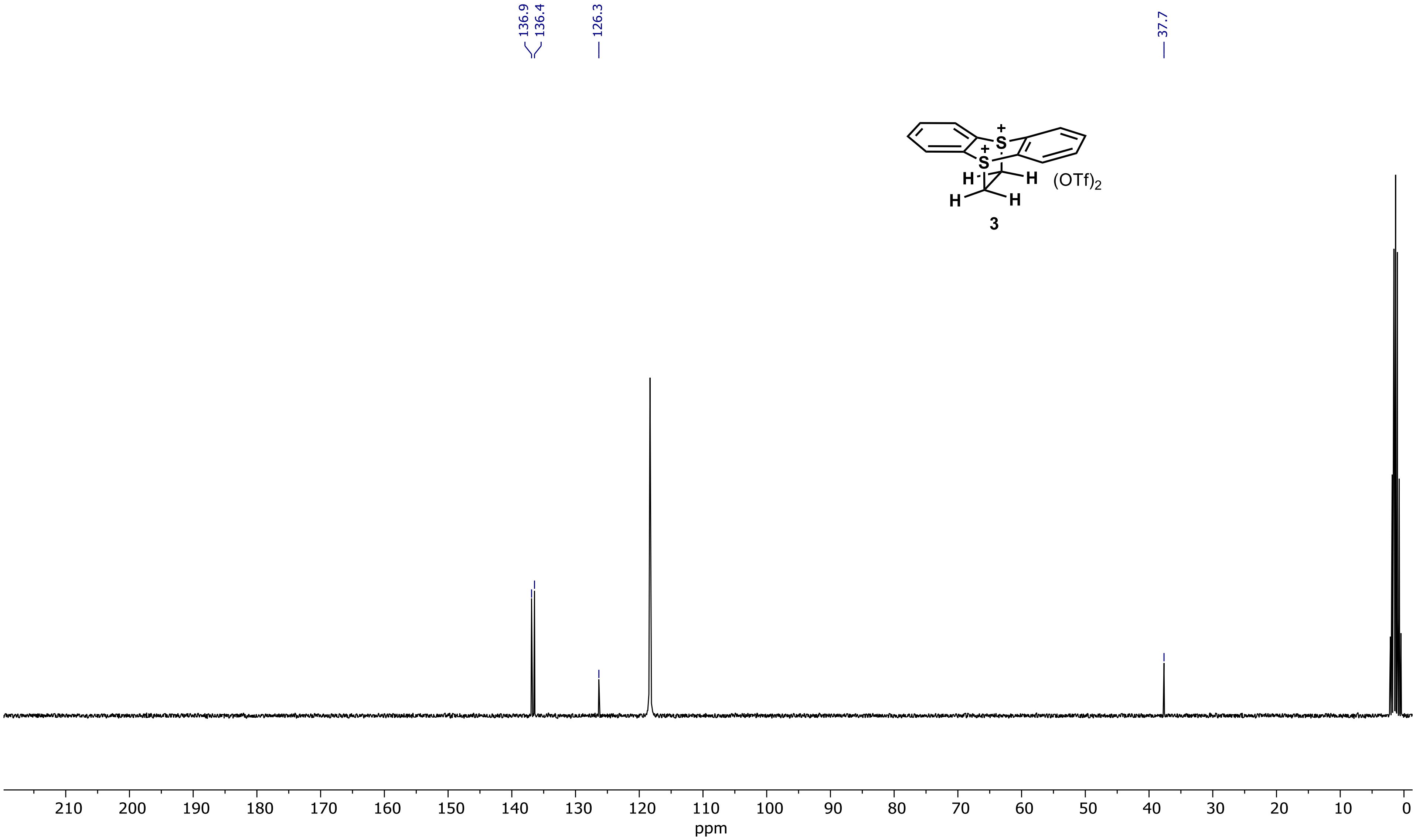

^{19}F NMR of the [4+2] cycloadduct intermediate 3

CD_3CN, 23 ºC

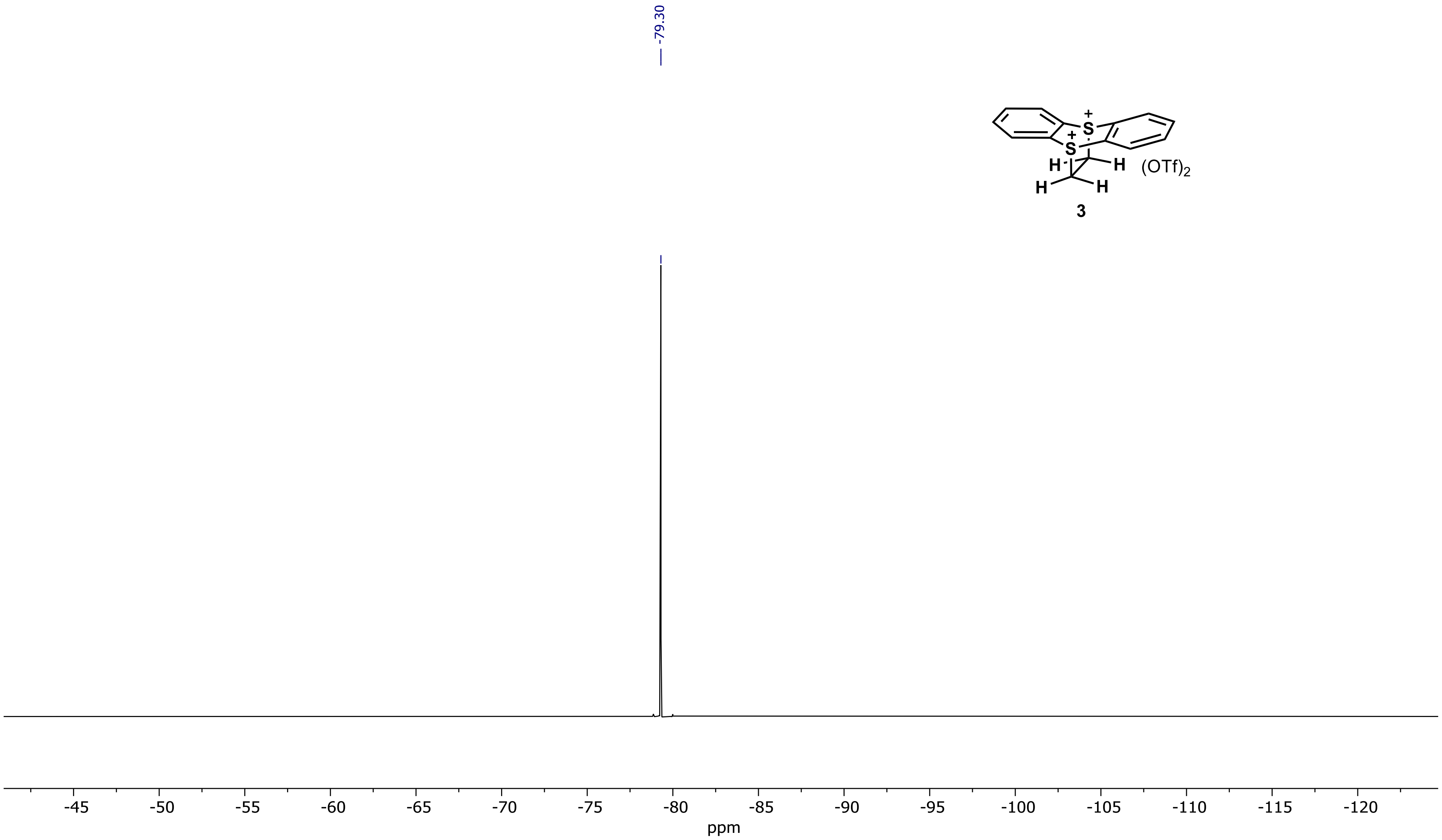

^{1}H NMR of spiro[cyclopropane-1,3'-indolin]-2'-one (5)

$CDCl_3$, 298 K

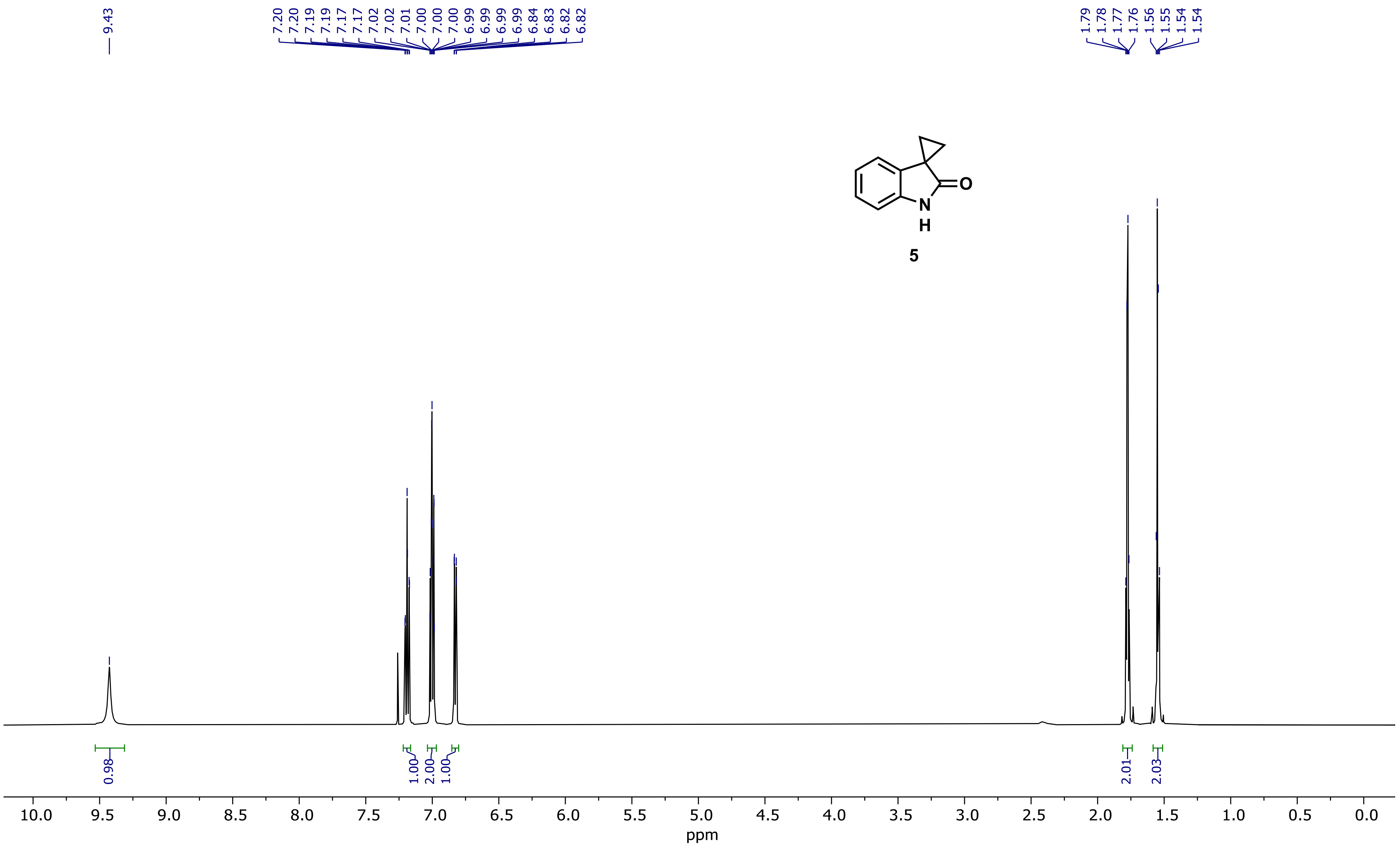

^{13}C NMR of spiro[cyclopropane-1,3'-indolin]-2'-one (5)

CDCl$_3$, 298 K

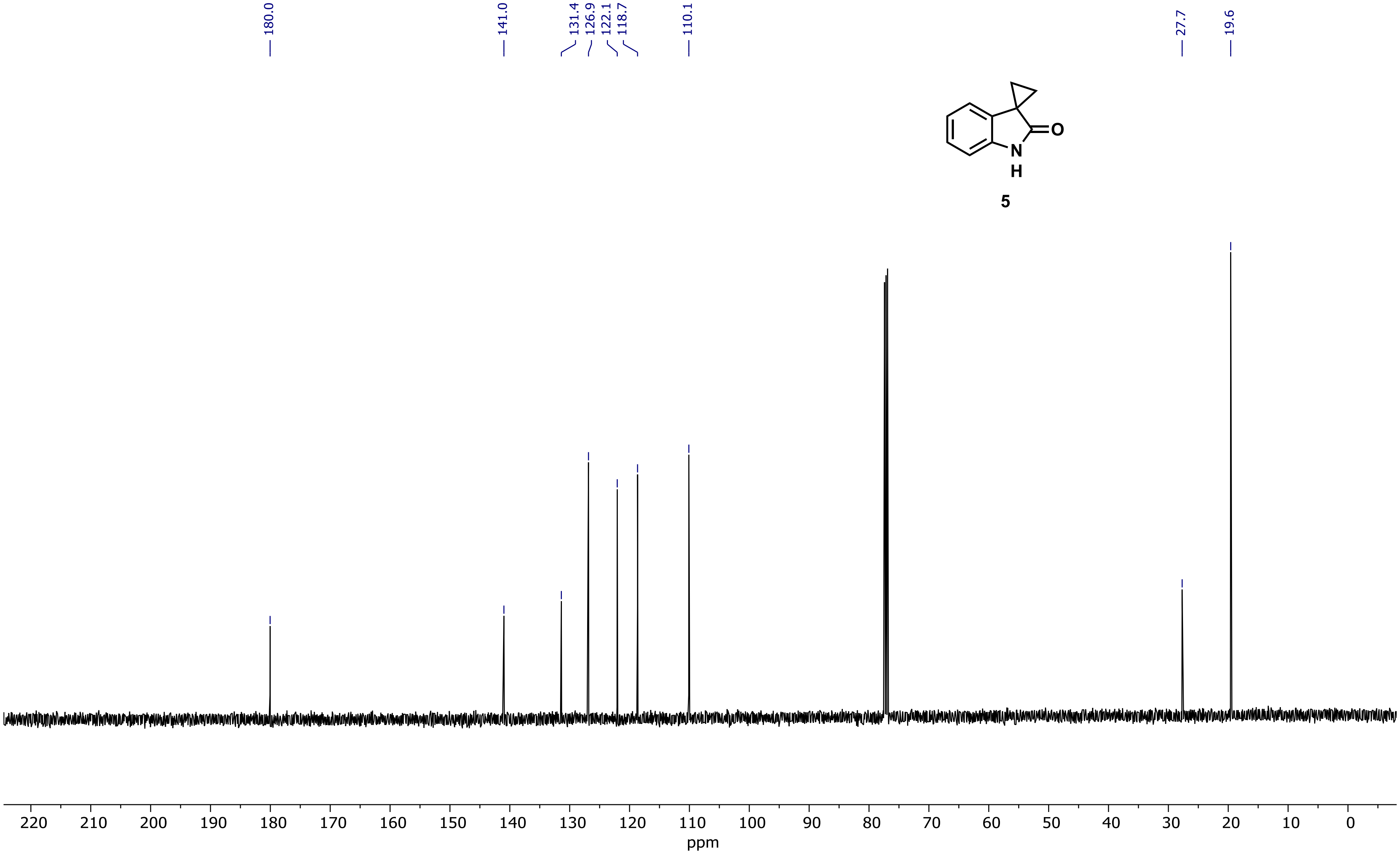

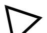

^{1}H NMR of methyl (±)-4-tosylmorpholine-3-carboxylate (7)

$CDCl_3$, 298 K

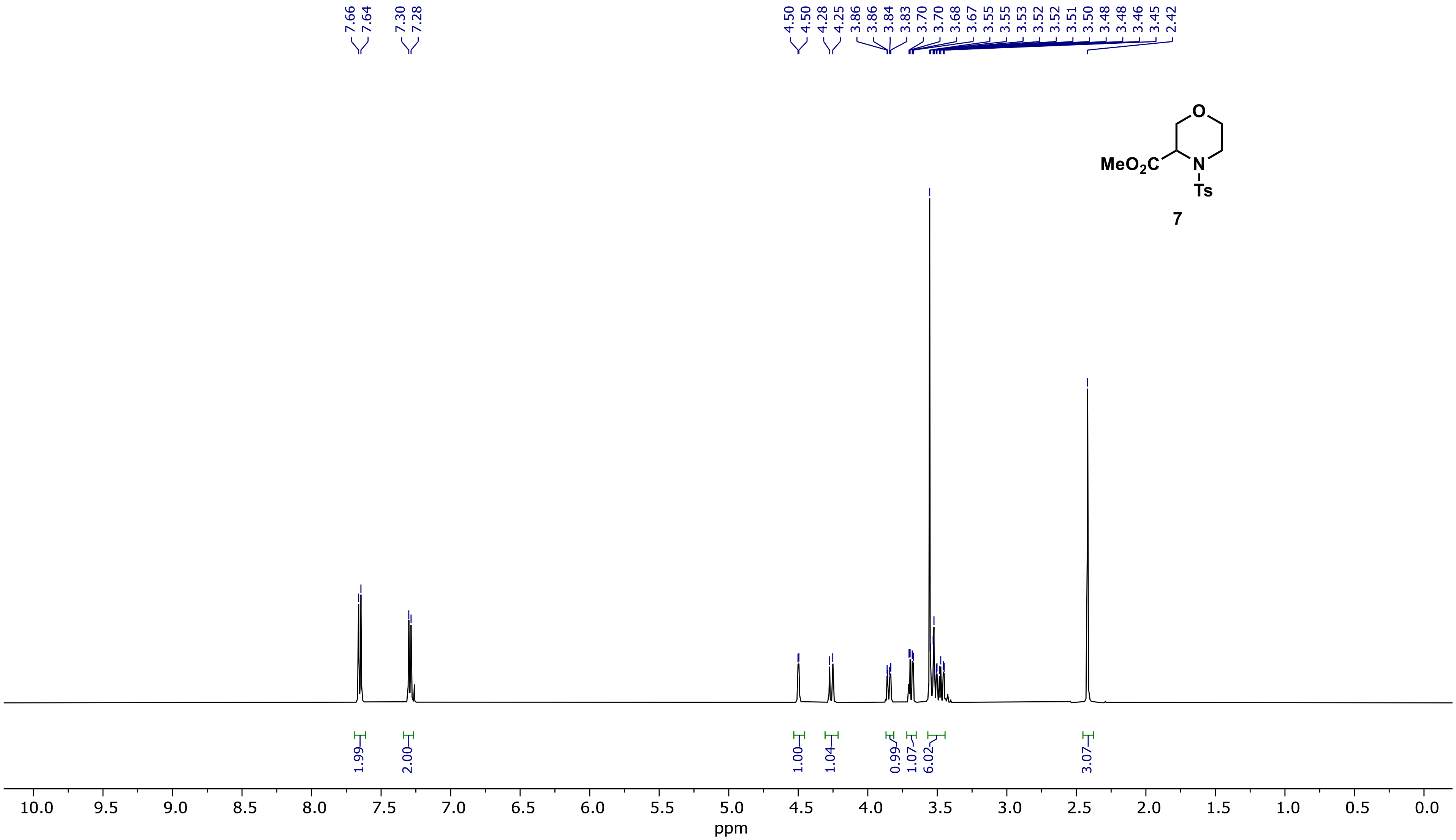

^{13}C NMR of methyl (±)-4-tosylmorpholine-3-carboxylate (7)

$CDCl_3$, 298 K

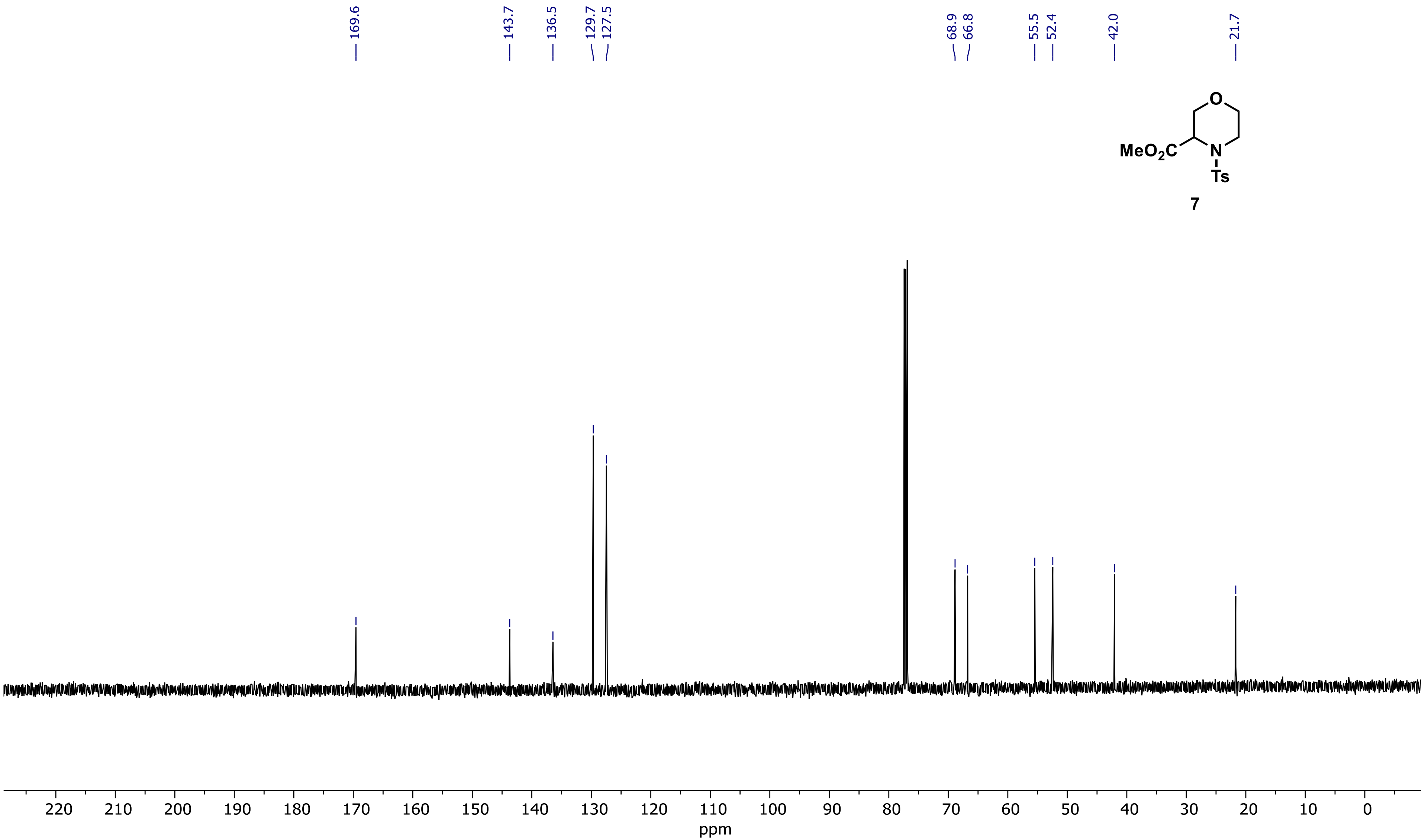

^{1}H NMR of ethyl (±)-2-phenyl-1-tosylazetidine-2-carboxylate (9)

$CDCl_3$, 298 K

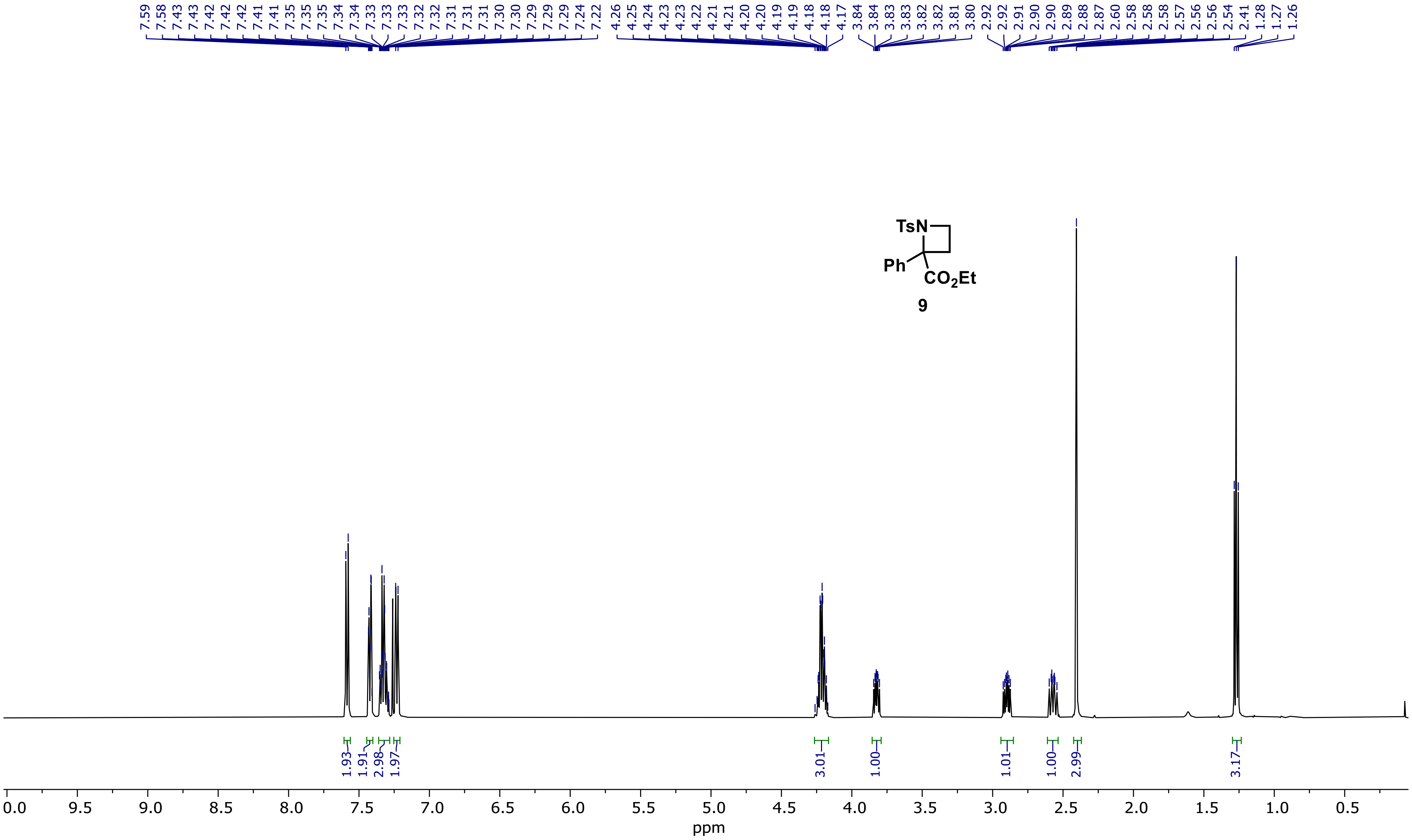

^{13}C NMR of ethyl (±)-2-phenyl-1-tosylazetidine-2-carboxylate (9)

$CDCl_3$, 298 K

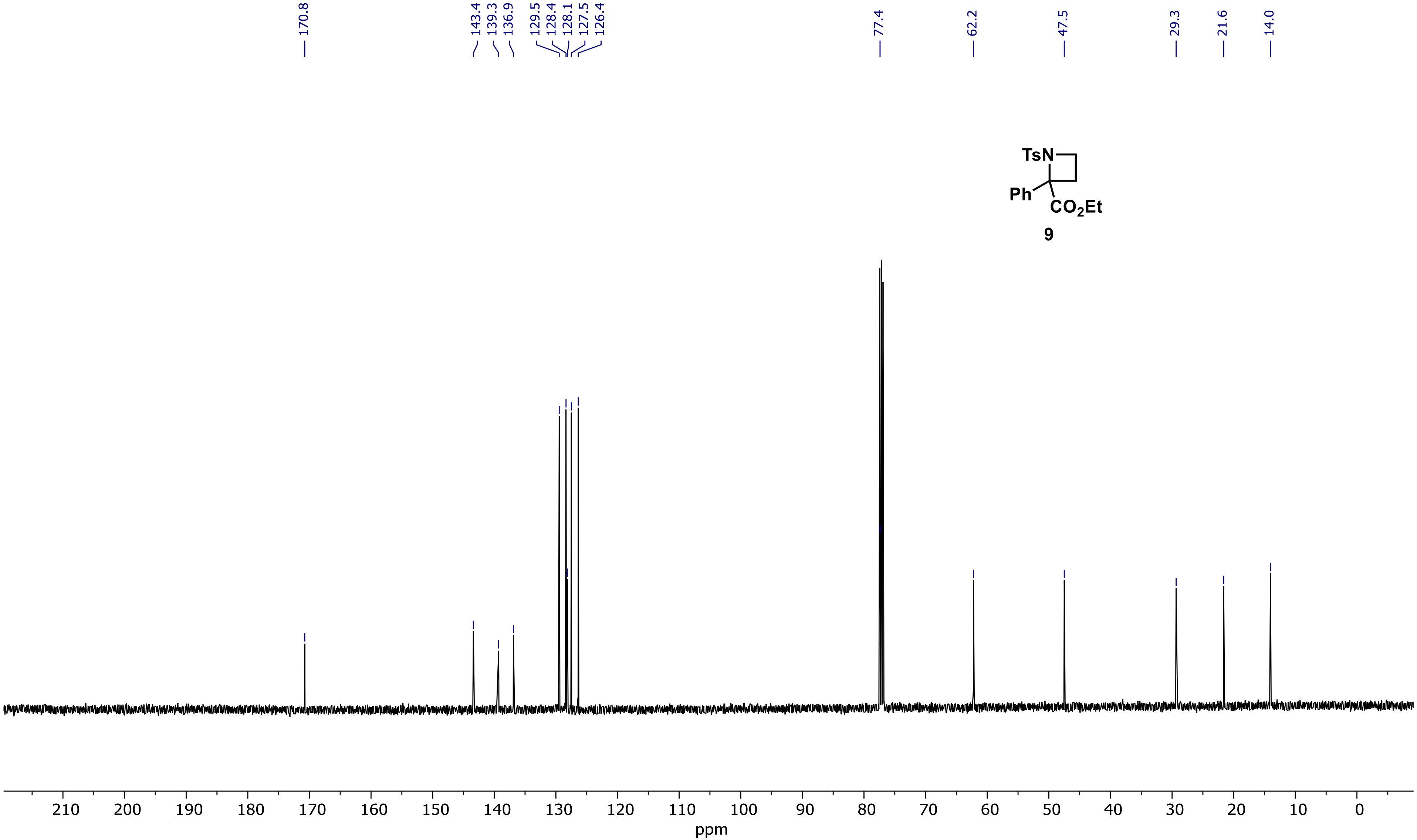

^{1}H NMR of (±)-3-tosyl-7-oxa-3-azabicyclo[4.1.0]heptane (11)

CDCl3, 298 K

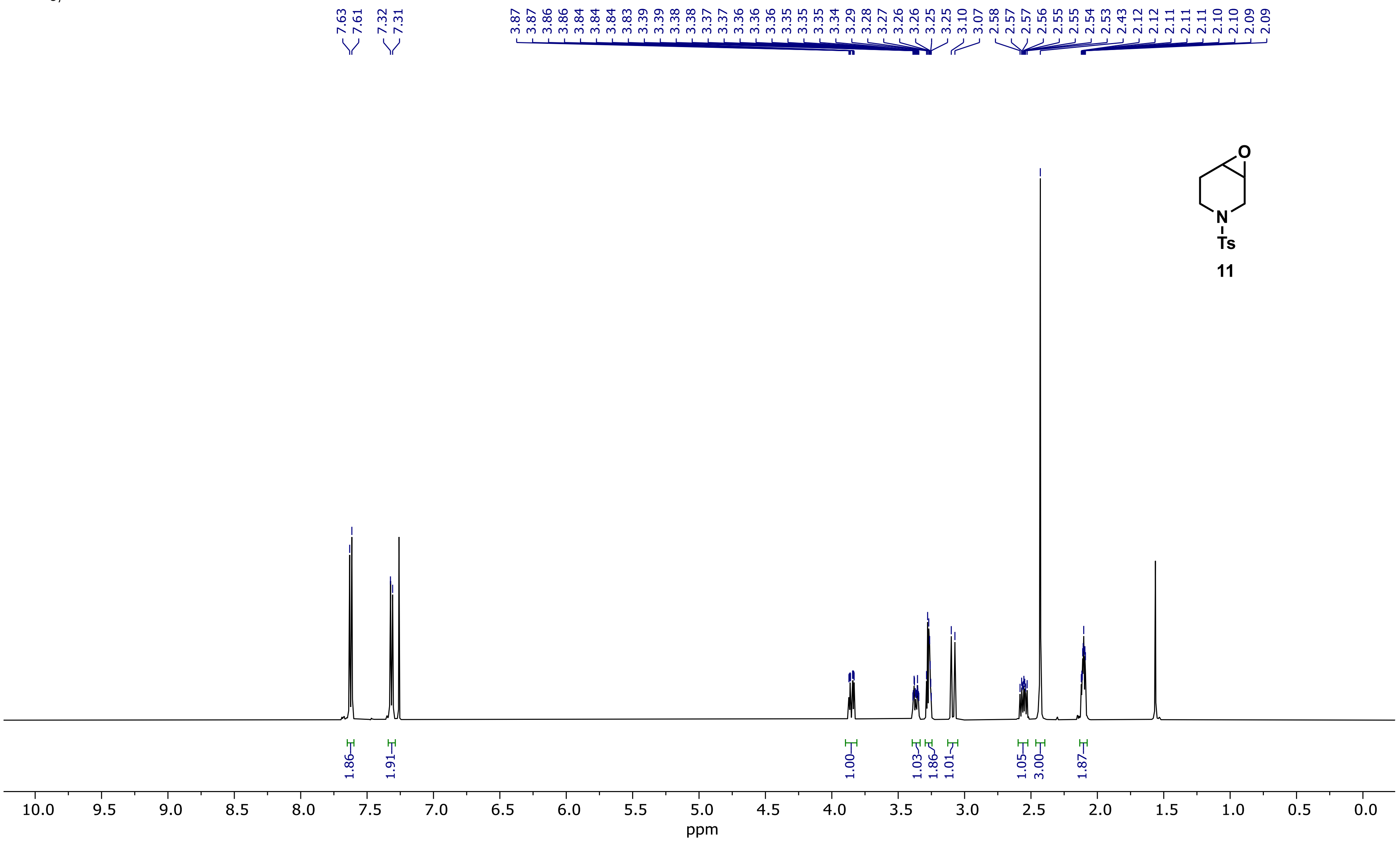

^{13}C NMR of (±)-3-tosyl-7-oxa-3-azabicyclo[4.1.0]heptane (11)

CDCl$_3$, 298 K

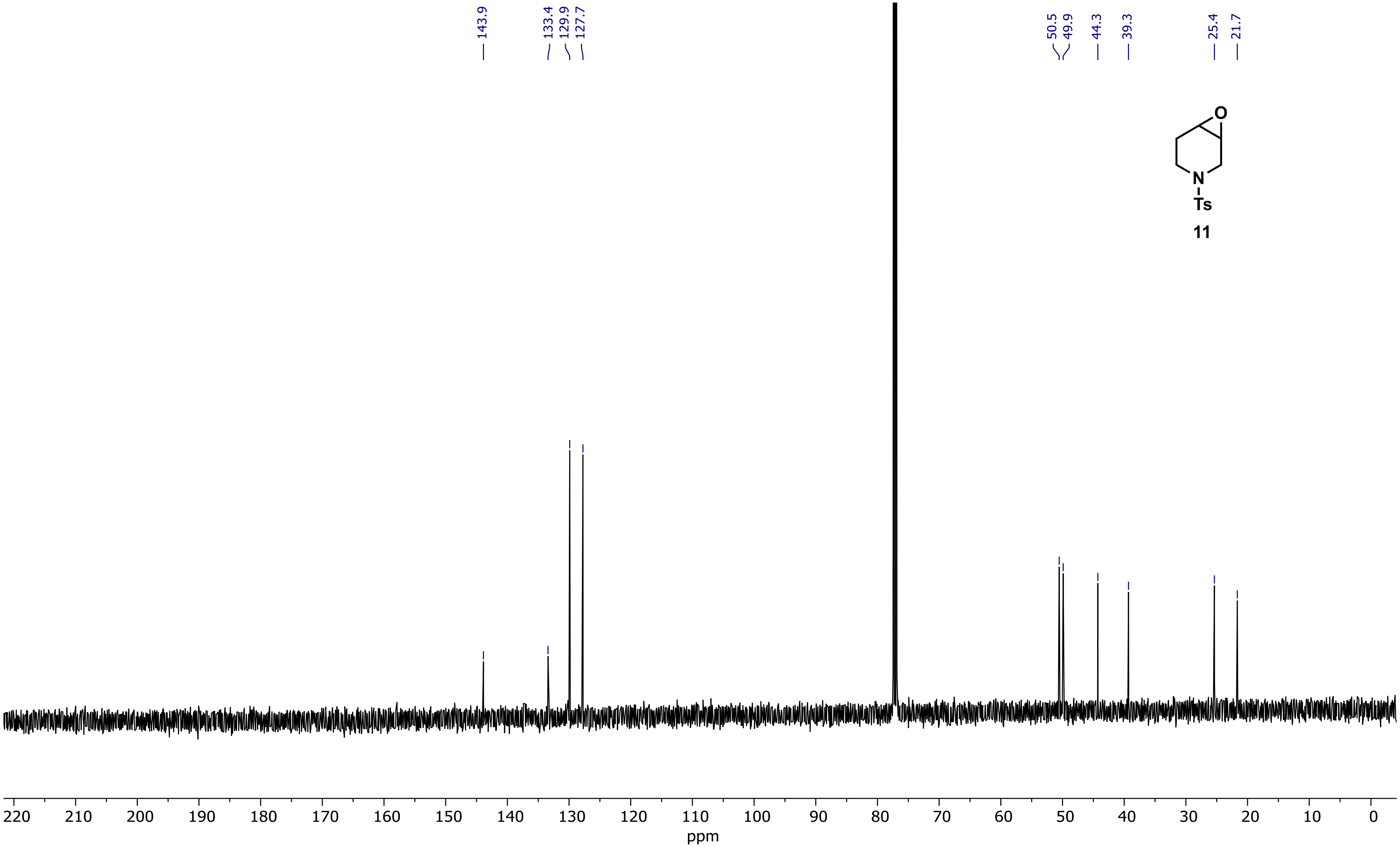

^{1}H NMR of 9-vinyl-9*H*-pyrido[3,4-*b*]indole (12)

DMSO-d$_6$, 298 K

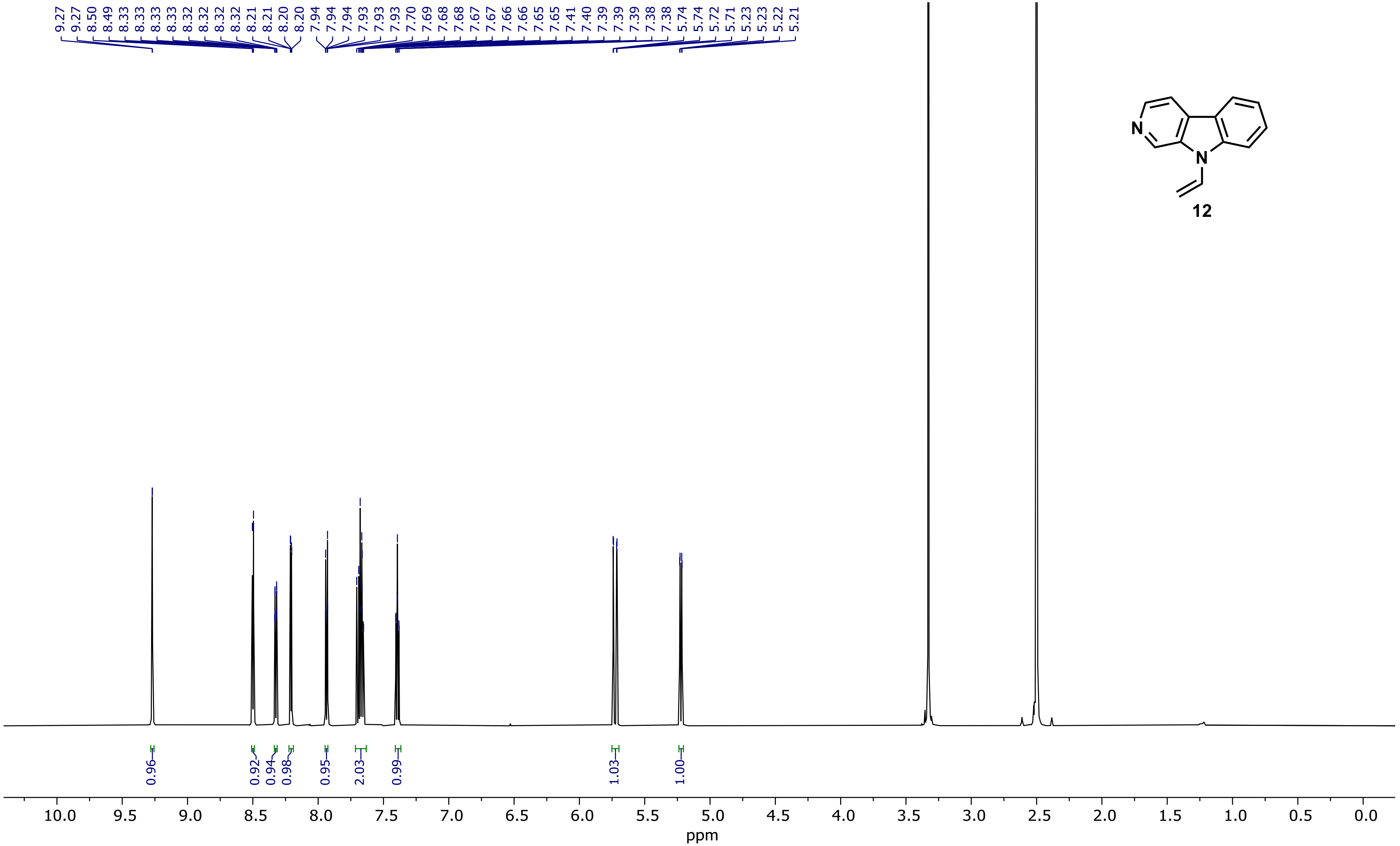

^{13}C NMR of 9-vinyl-9*H*-pyrido[3,4-*b*]indole (12)

DMSO-d$_6$, 298 K

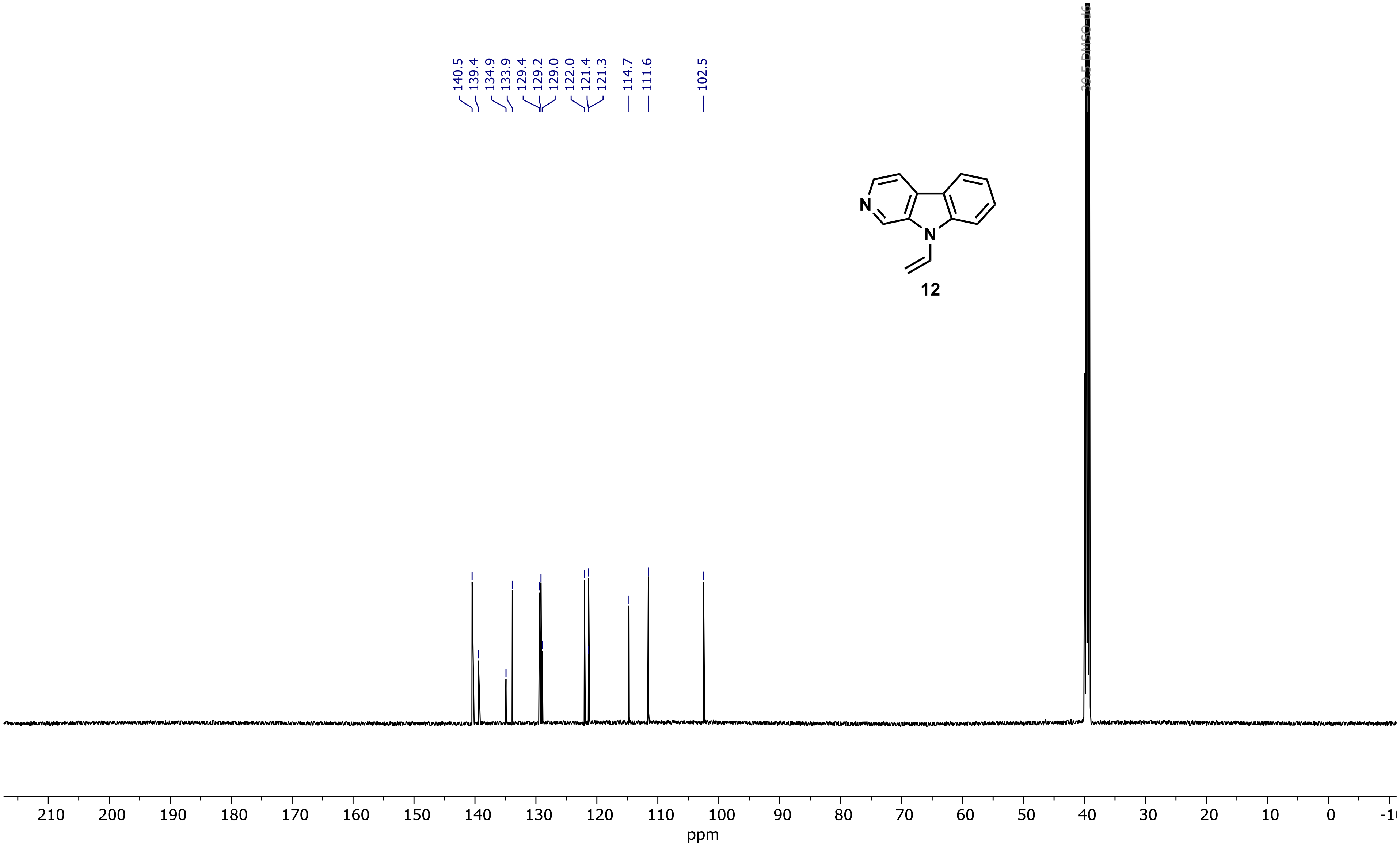

^{1}H NMR of 5-nitro-1-vinyl-1*H*-indole (13)

$CDCl_3$, 298 K

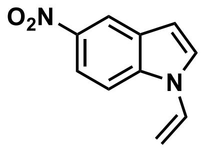

13

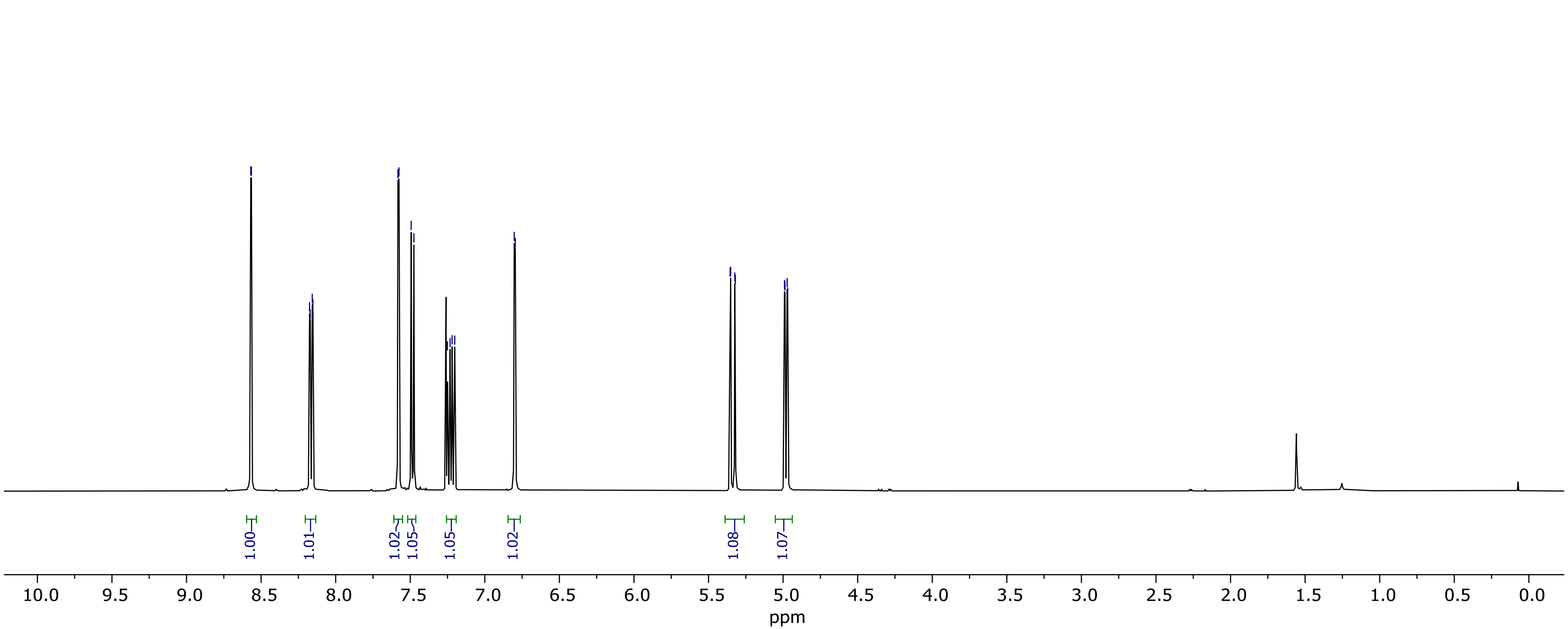

^{13}C NMR of 5-nitro-1-vinyl-1*H*-indole (13)

$CDCl_3$, 298 K

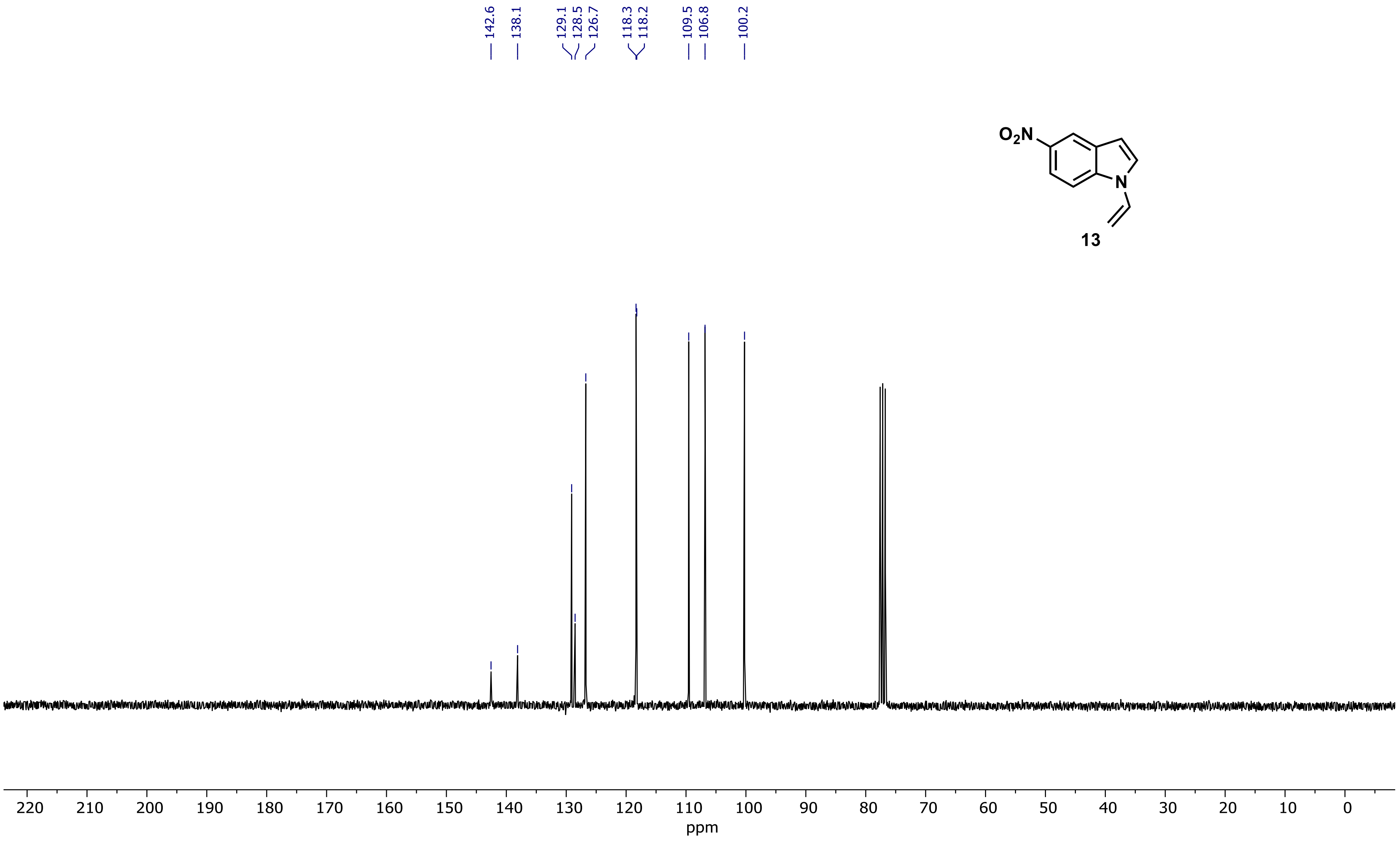

^{1}H NMR of 1-vinyl-1*H*-indole-3-carbaldehyde (14)

$CDCl_3$, 298 K

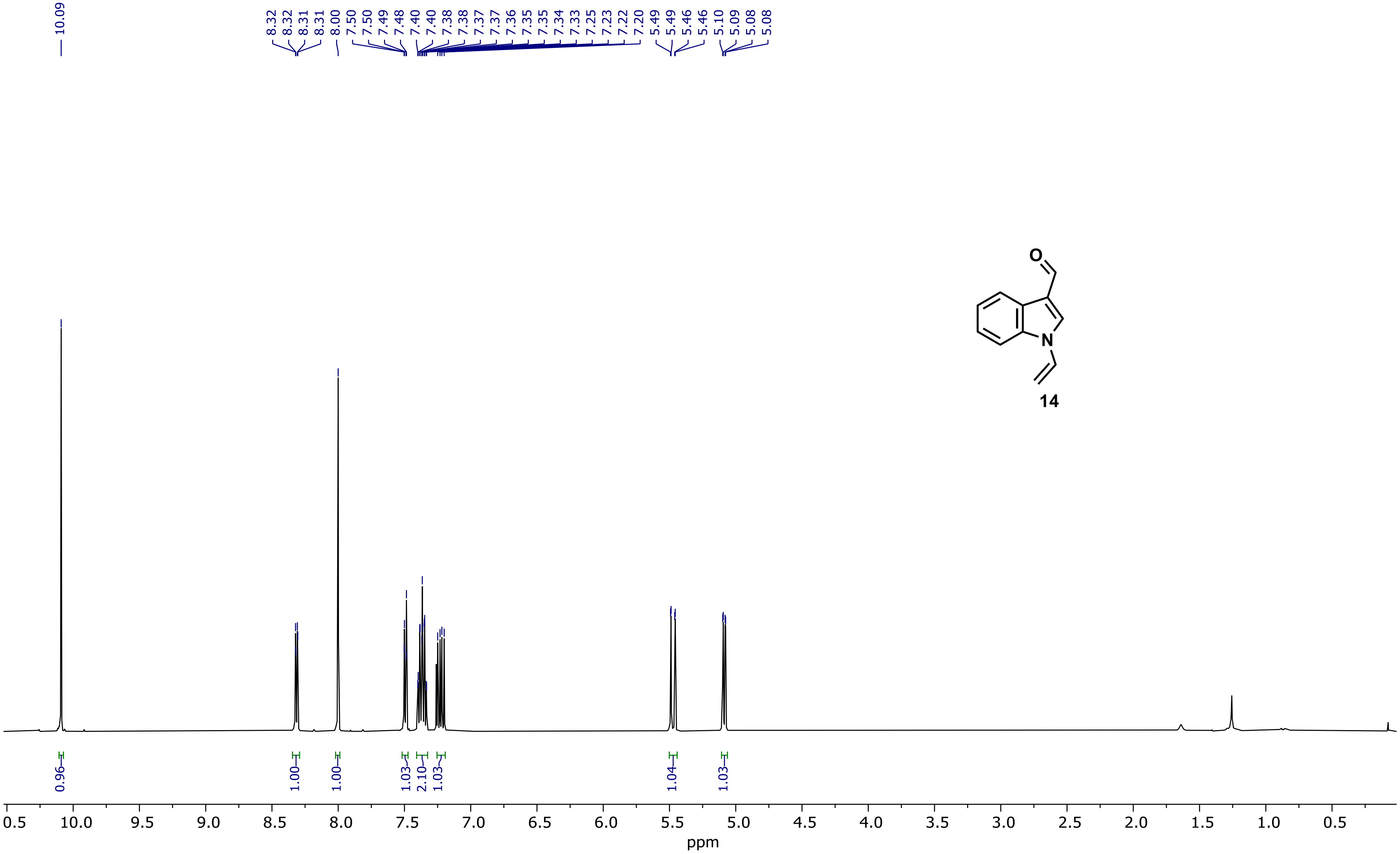

^{13}C NMR of 1-vinyl-1*H*-indole-3-carbaldehyde (14)

$CDCl_3$, 298 K

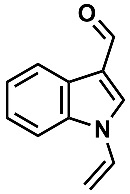

14

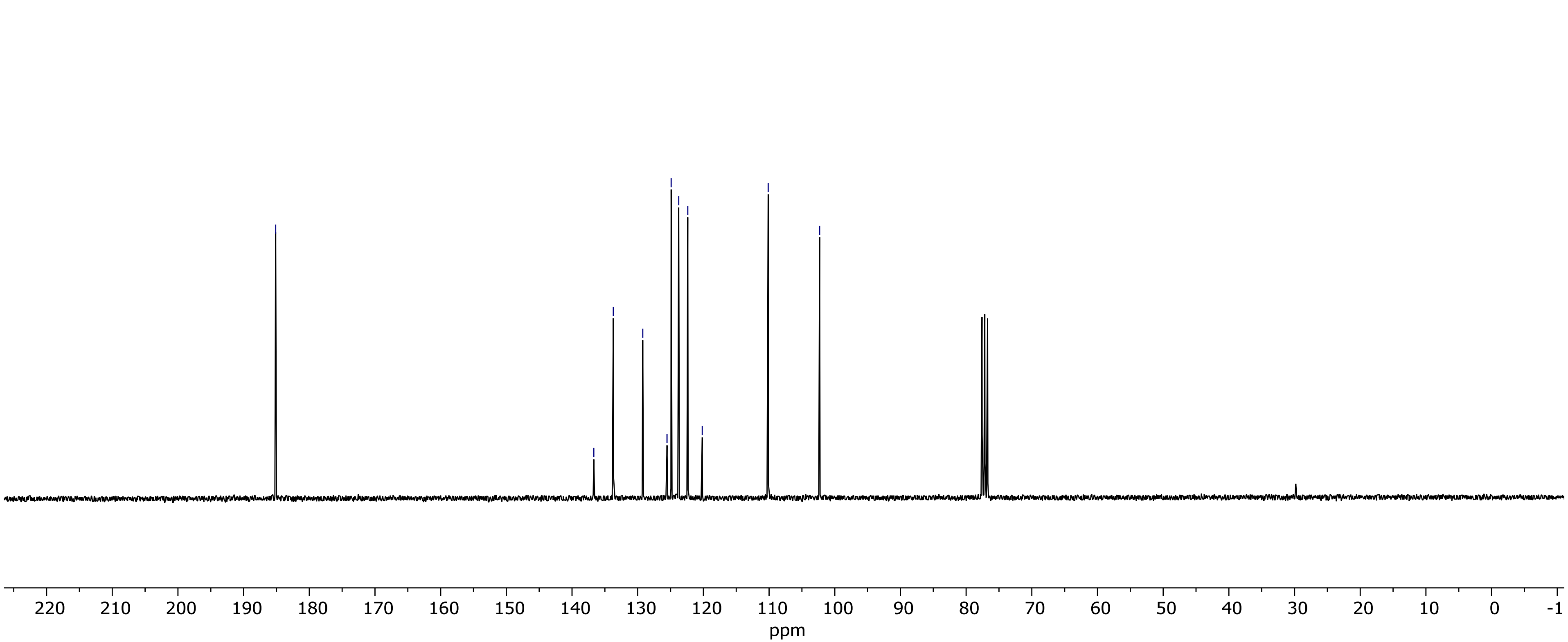

^{1}H NMR of ethyl 3-amino-1-vinyl-1H-pyrazole-4-carboxylate (15-I)

CD_2Cl_2, 298 K

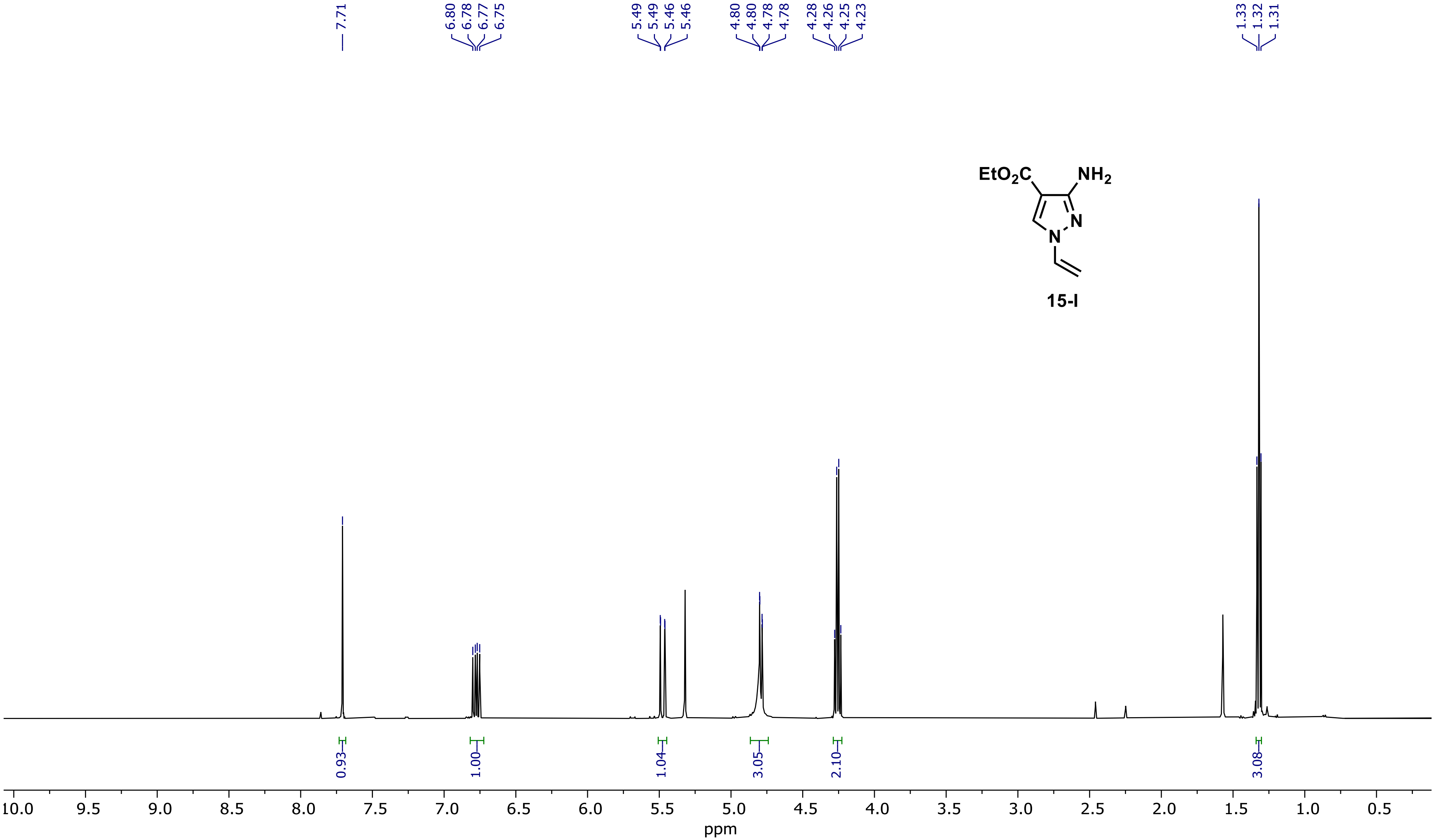

^{13}C NMR of ethyl 3-amino-1-vinyl-1H-pyrazole-4-carboxylate (15-I)

CD_2Cl_2, 298 K

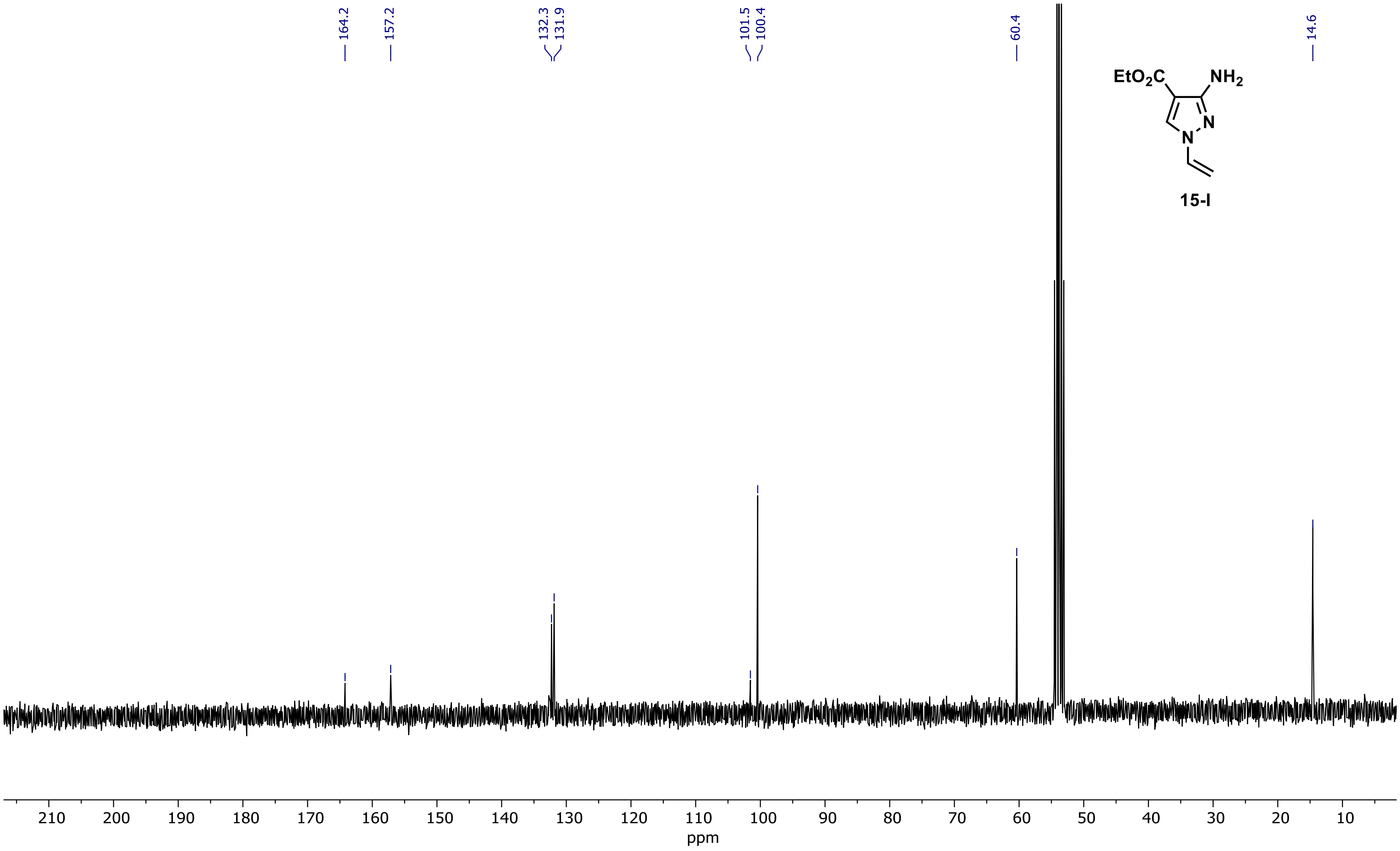

^{1}H NMR of ethyl 5-amino-1-vinyl-1H-pyrazole-4-carboxylate (15-II)

CD_2Cl_2, 298 K

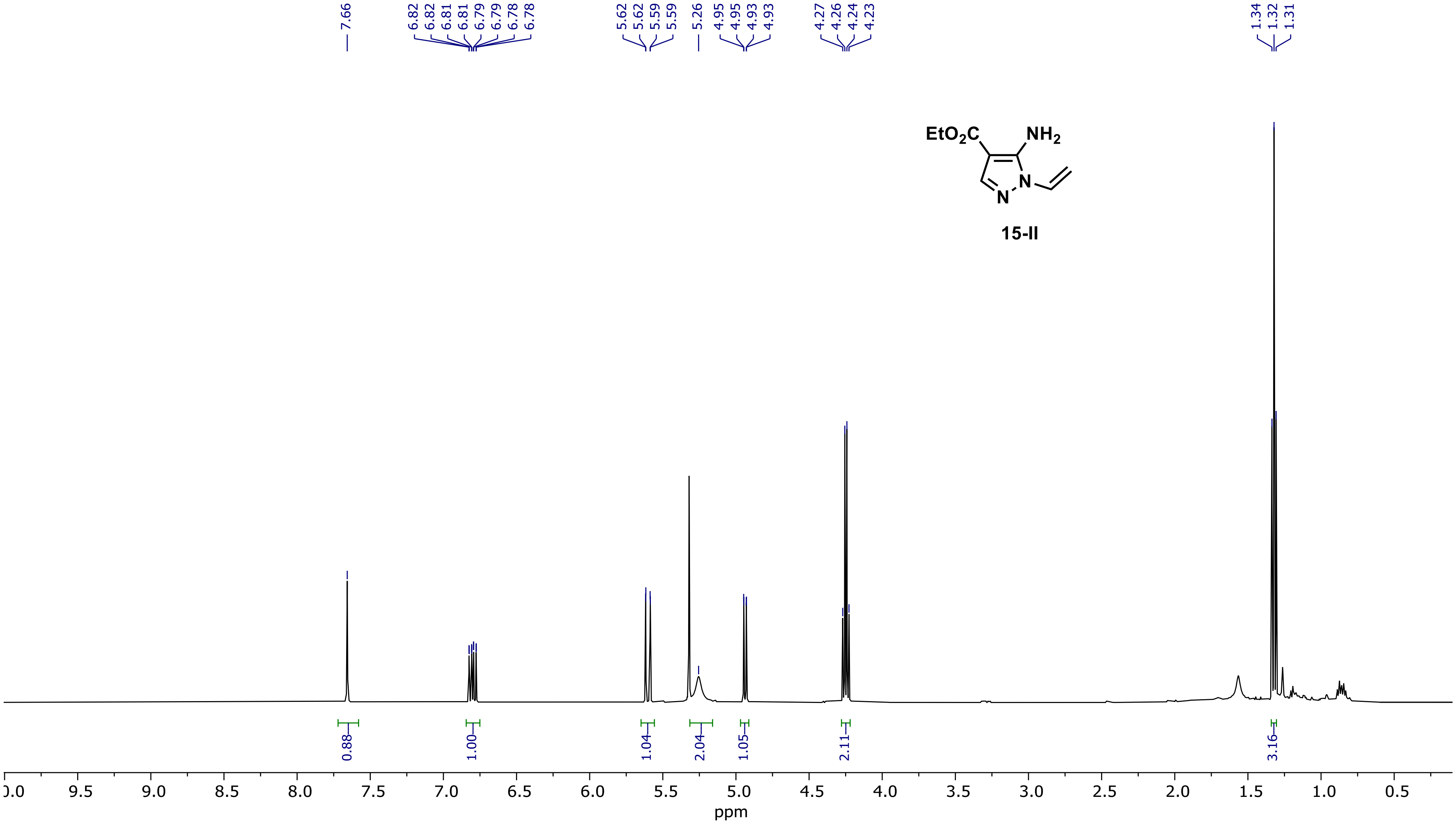

^{13}C NMR of ethyl 5-amino-1-vinyl-1H-pyrazole-4-carboxylate (15-II)

CD_2Cl_2, 298 K

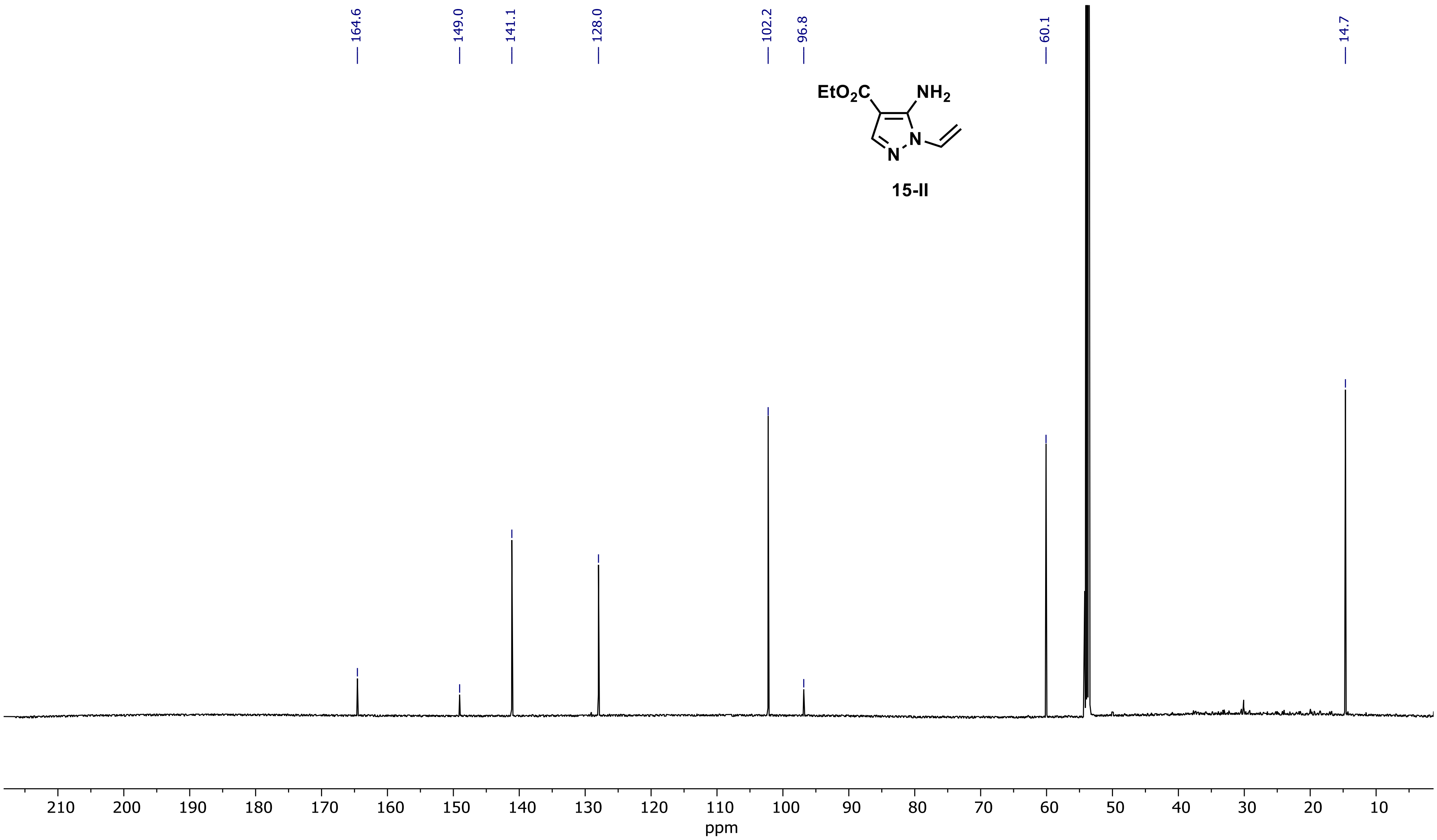

^{1}H NMR of 4-bromo-3,5-dimethyl-1-vinyl-1*H*-pyrazole (16)

$CDCl_3$, 298 K

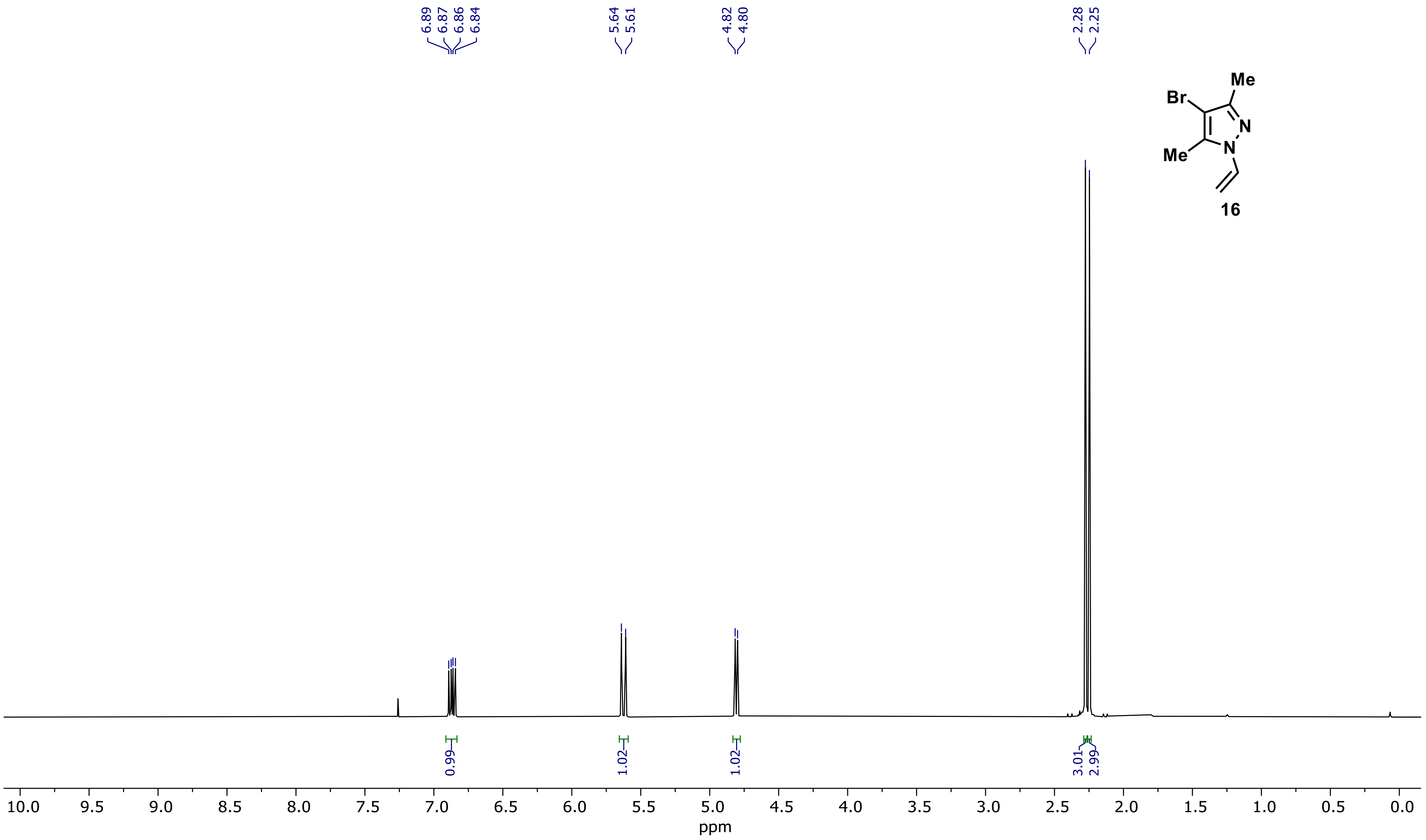

^{13}C NMR of 4-bromo-3,5-dimethyl-1-vinyl-1*H*-pyrazole (16)

$CDCl_3$, 298 K

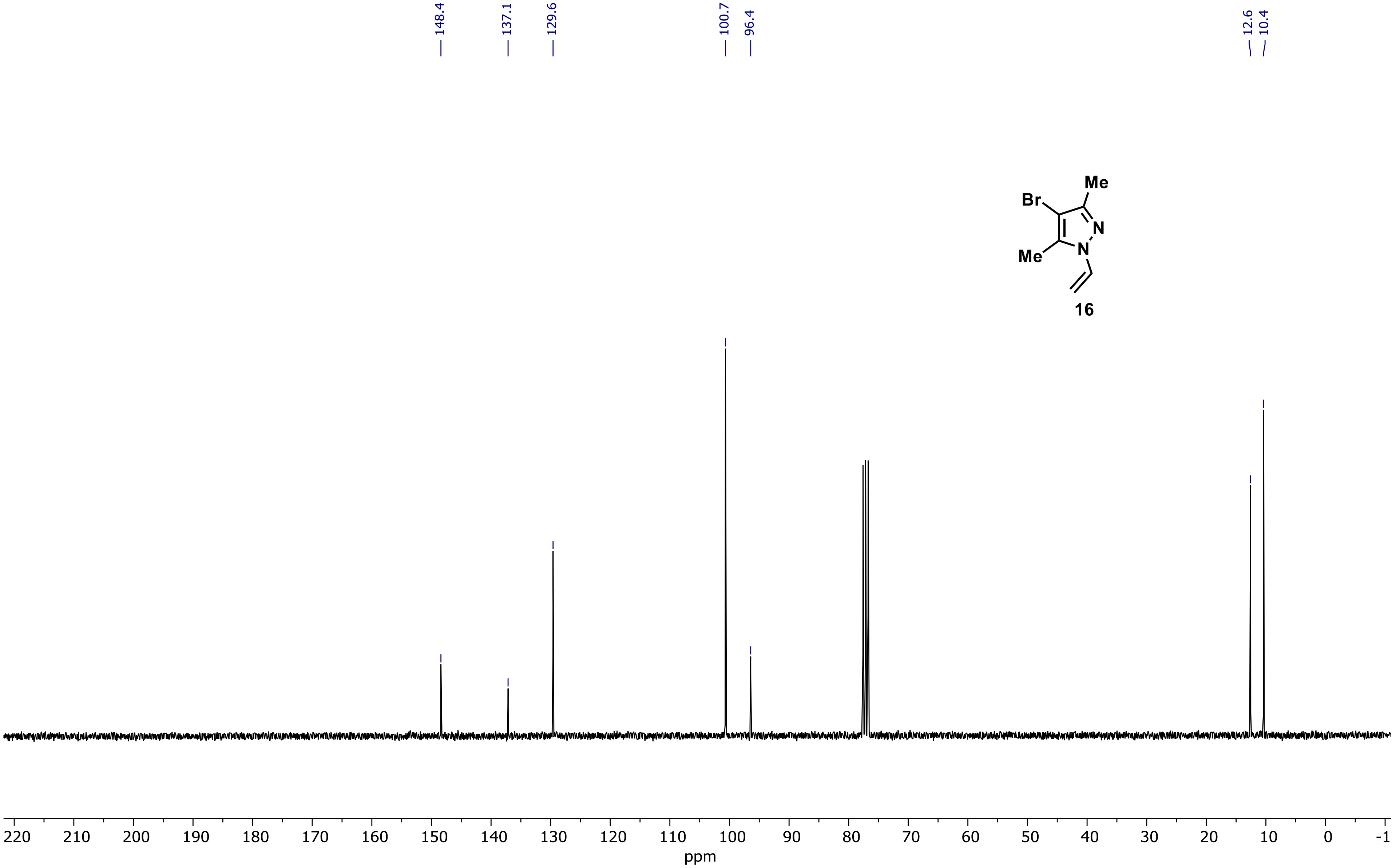

16

^{1}H NMR of methyl 1-vinyl-1*H*-1,2,4-triazole-3-carboxylate (17)

CDCl$_3$, 298 K

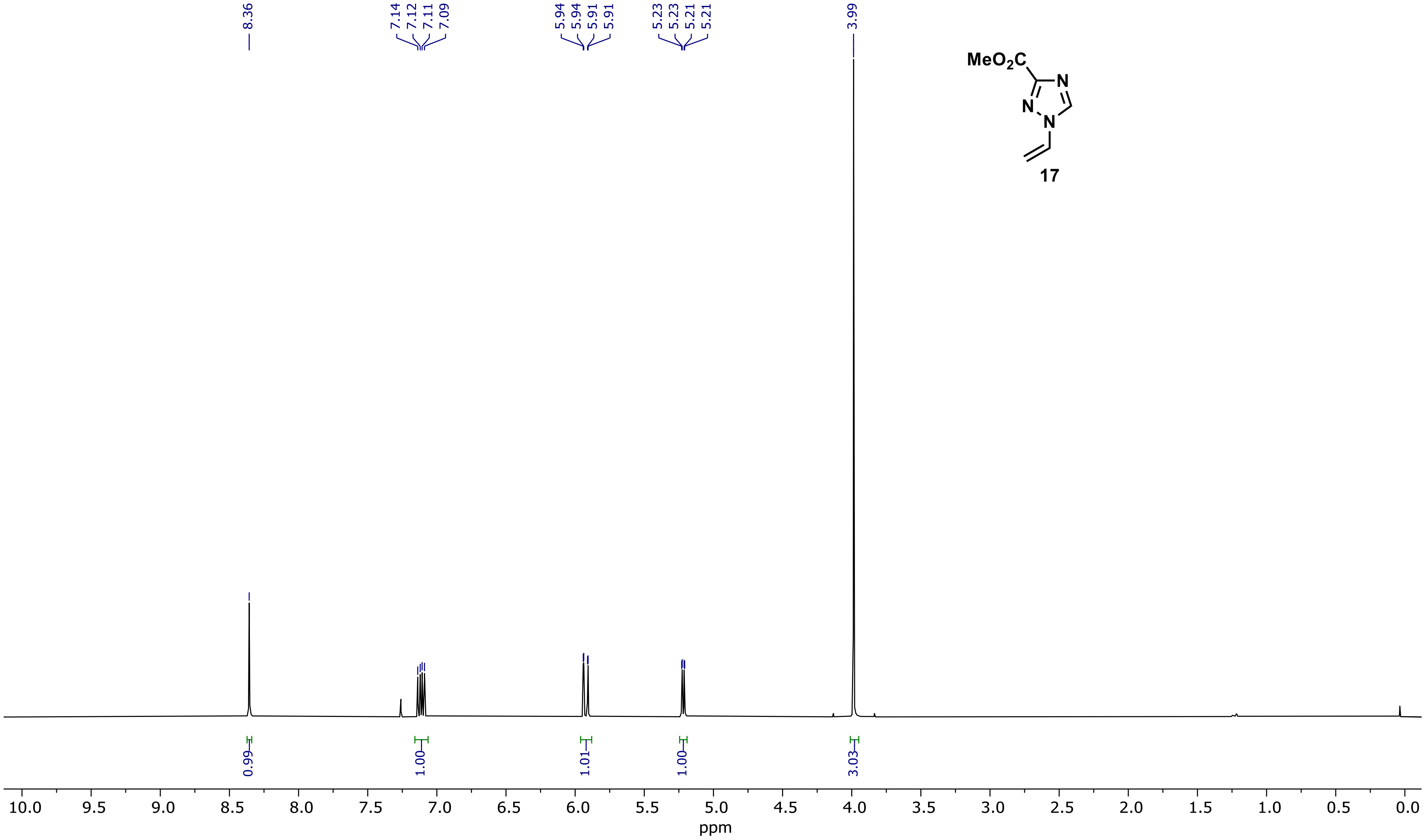

17

^{13}C NMR of methyl 1-vinyl-1*H*-1,2,4-triazole-3-carboxylate (17)

$CDCl_3$, 298 K

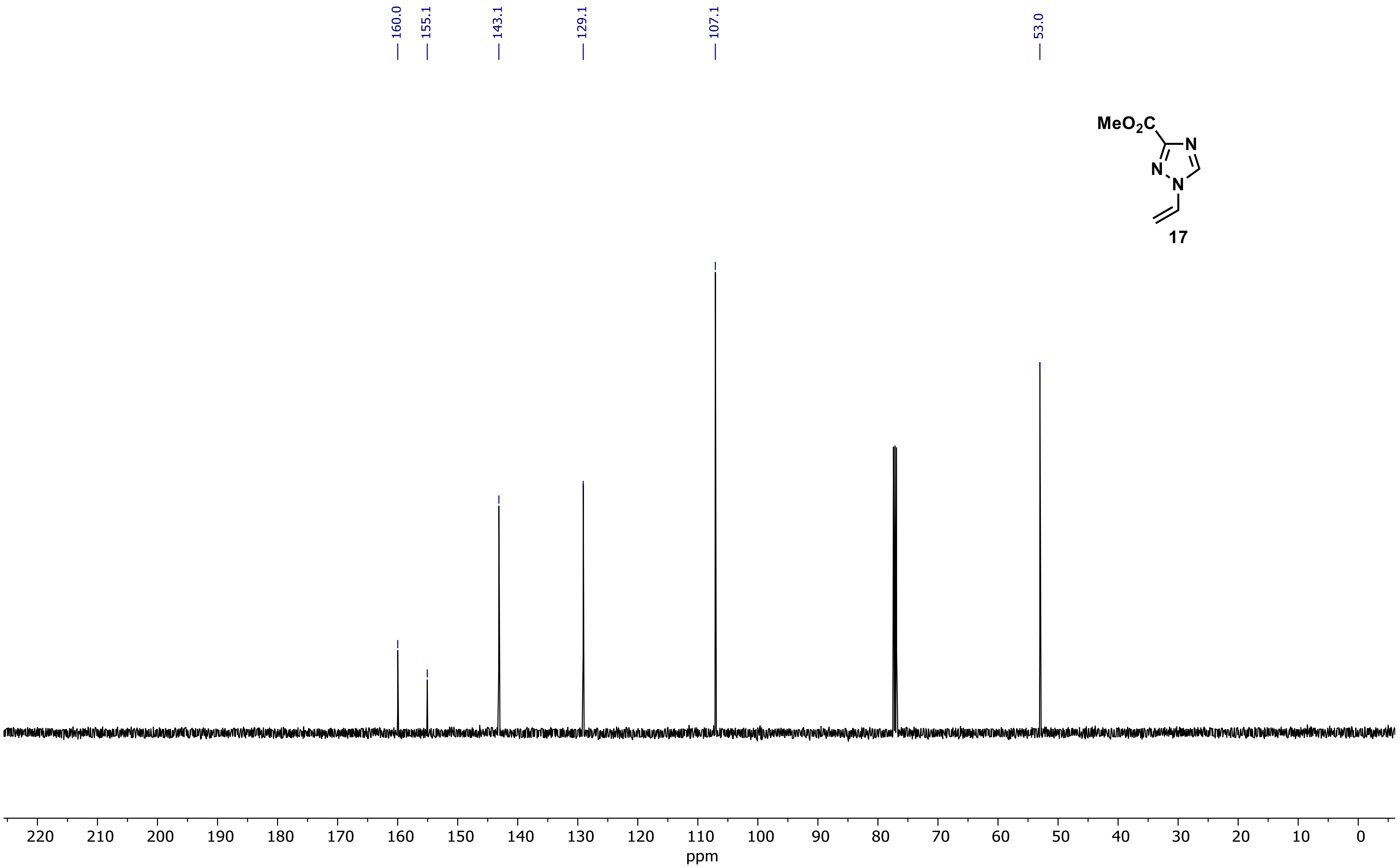

^{1}H NMR of 1-vinylpyridin-4(1*H*)-one (18)

$CDCl_3$, 298 K

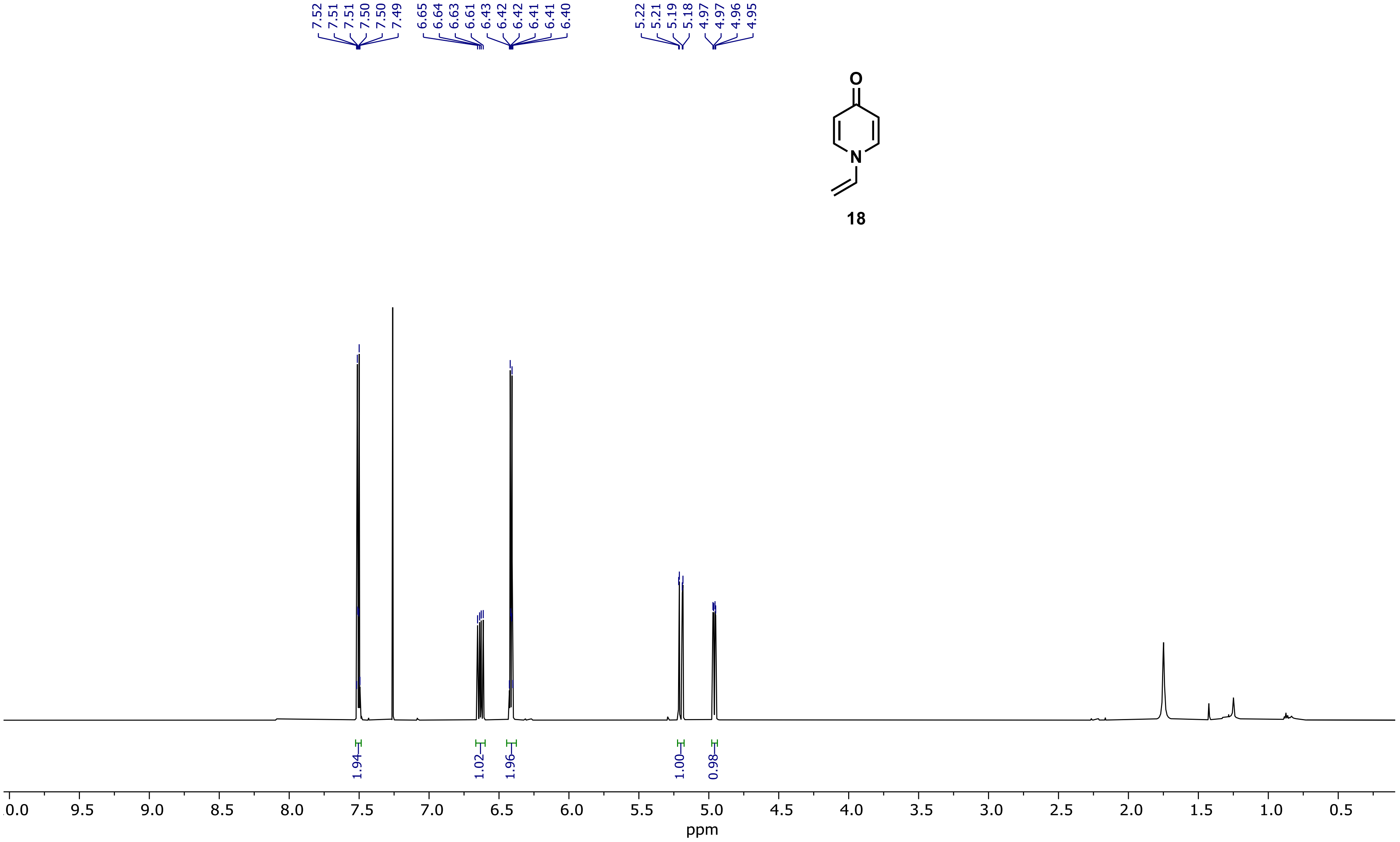

^{13}C NMR of 1-vinylpyridin-4(1*H*)-one (18)

$CDCl_3$, 298 K

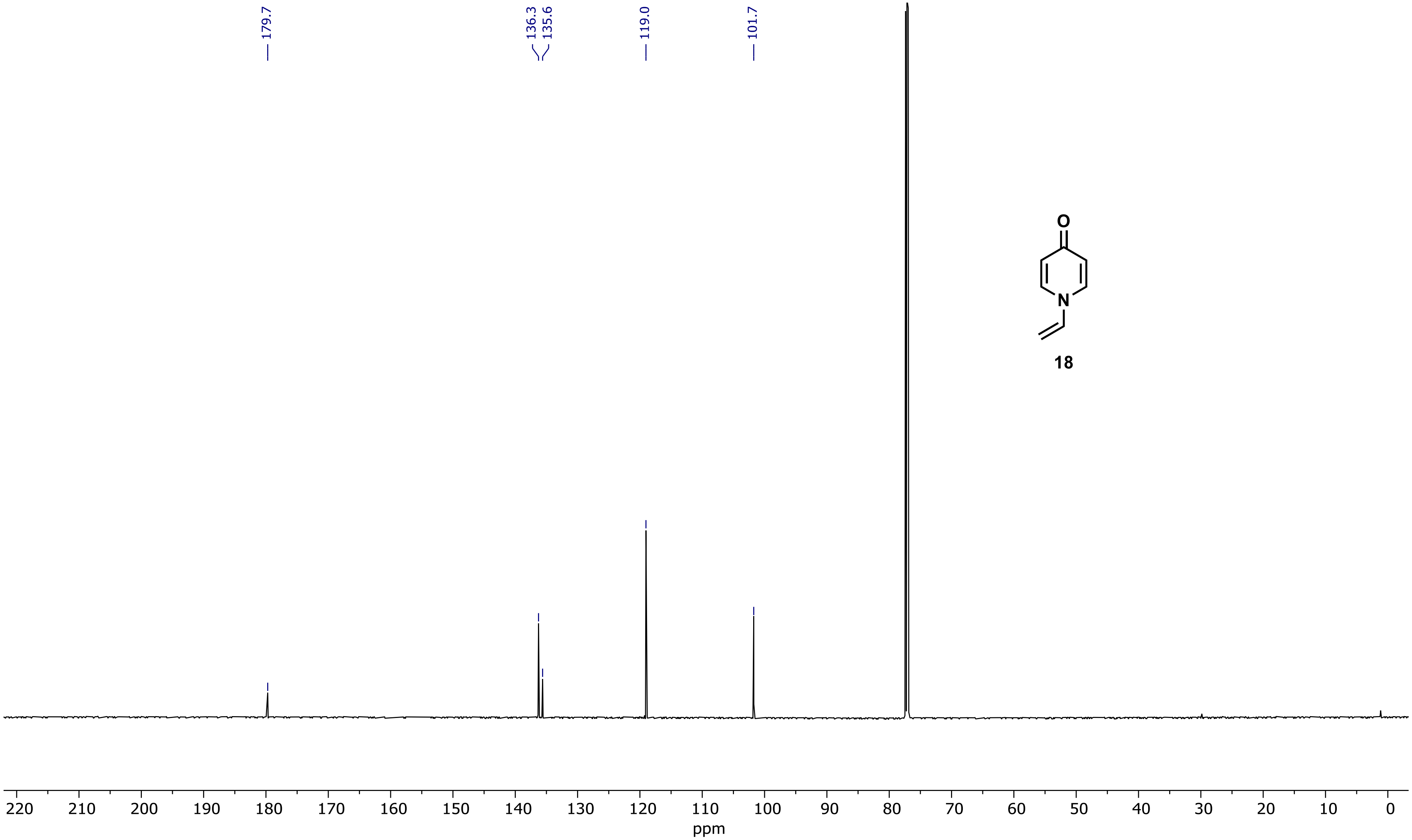

^{1}H NMR of 4-(4-fluorophenyl)-1-vinyl-1*H*-imidazole (19)

CD_2Cl_2, 298 K

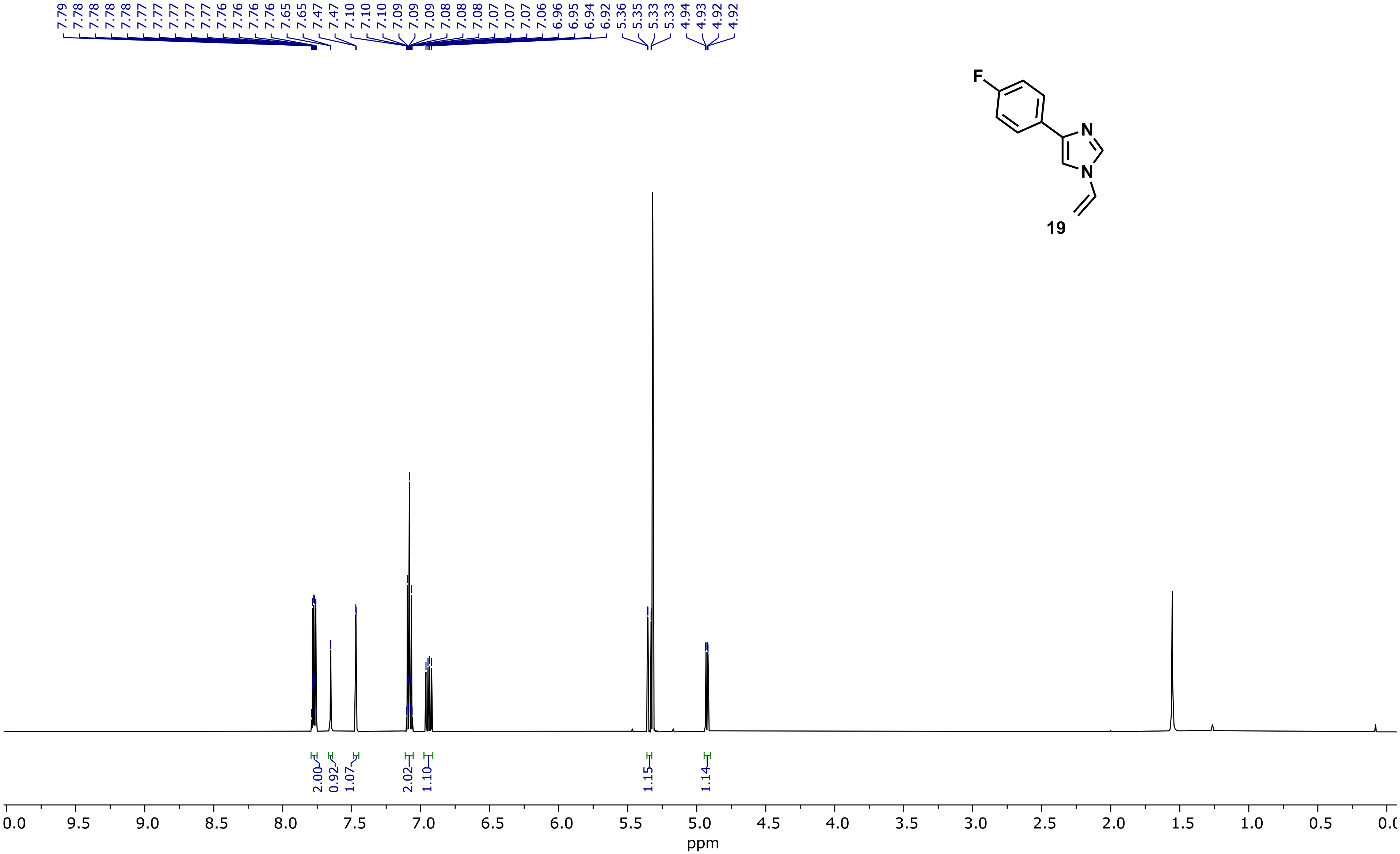

19

^{13}C NMR of 4-(4-fluorophenyl)-1-vinyl-1*H*-imidazole (19)

CD_2Cl_2, 298 K

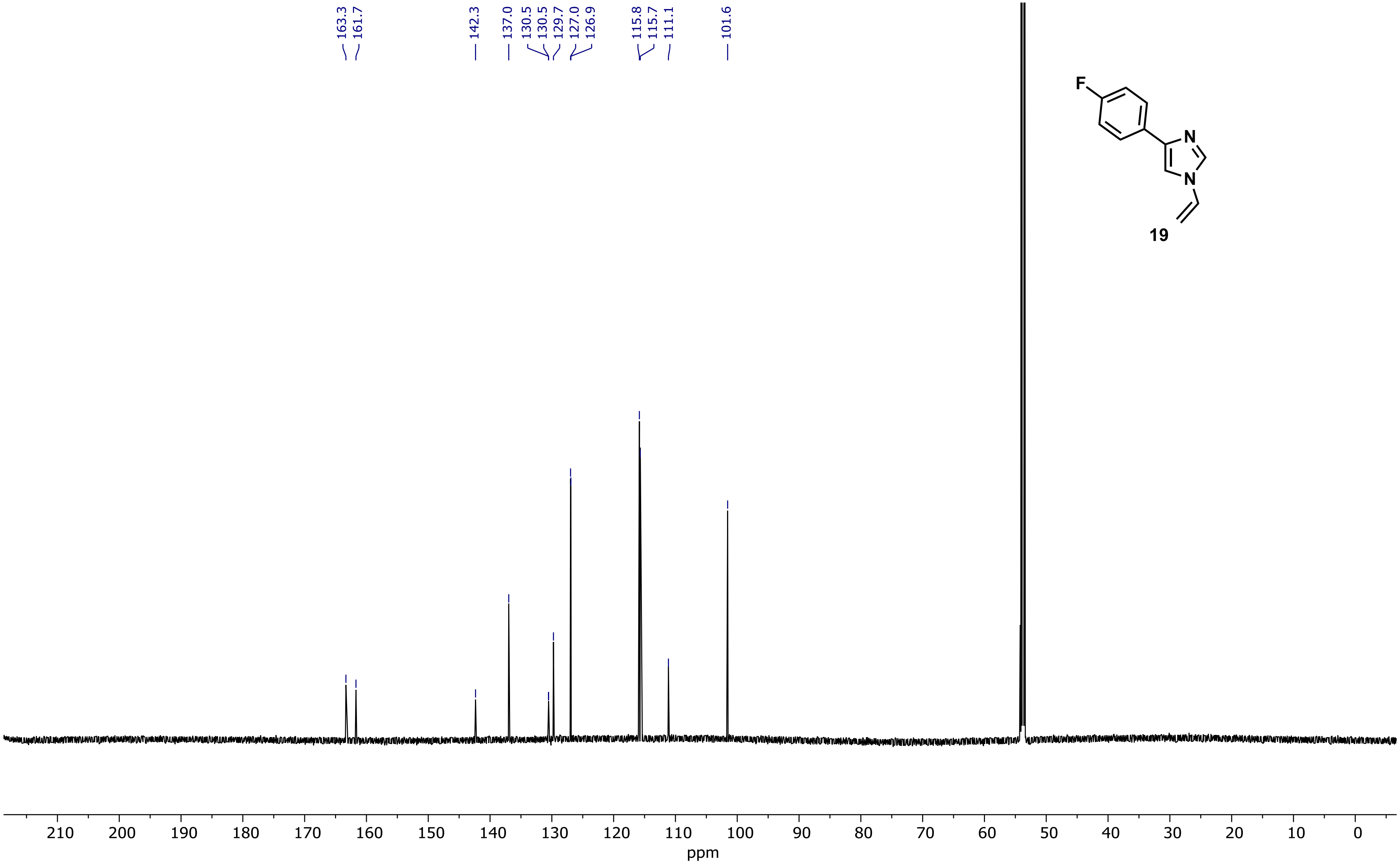

^{19}F NMR of 4-(4-fluorophenyl)-1-vinyl-1*H*-imidazole (19)

CD_2Cl_2, 298 K

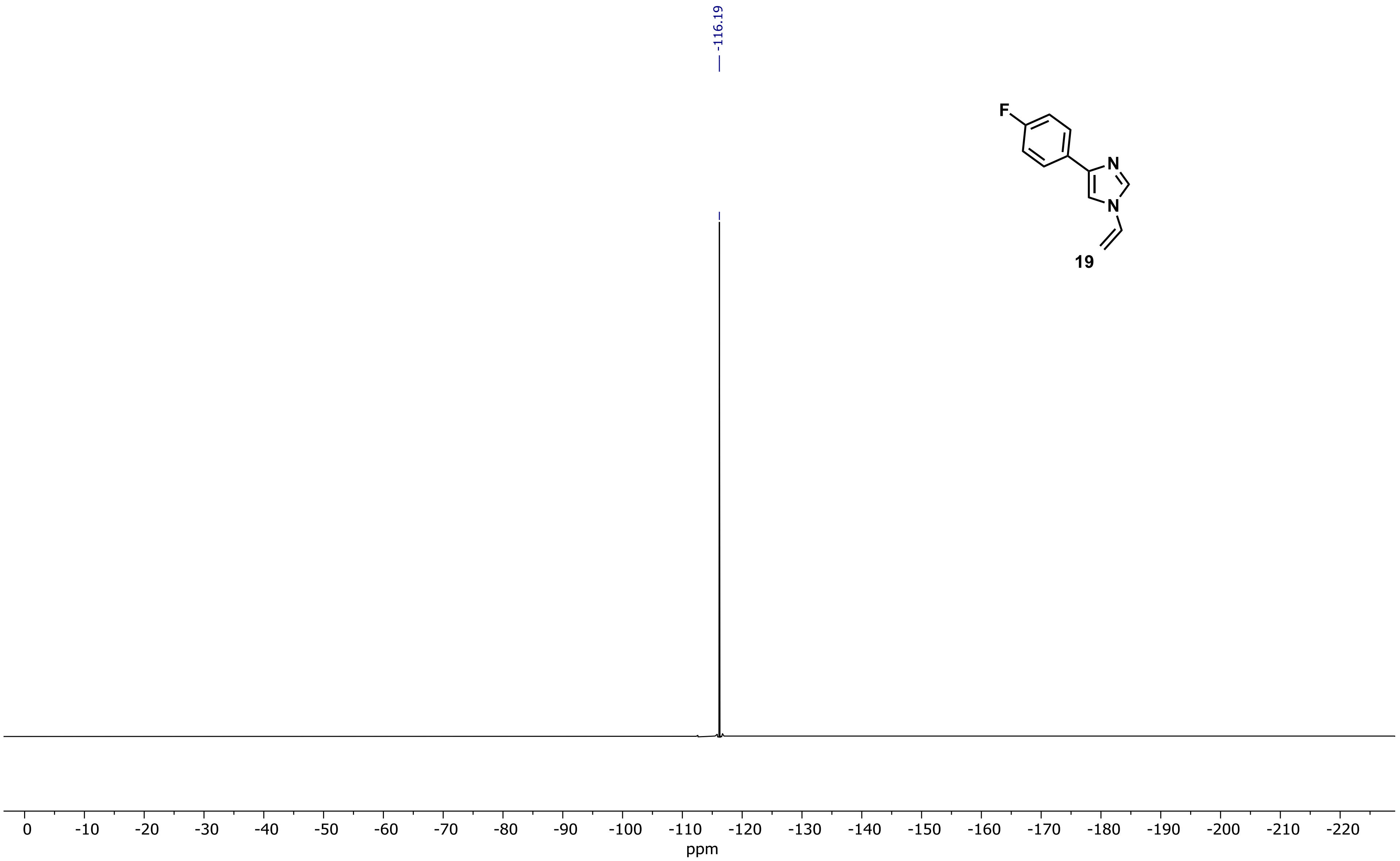

^{1}H NMR of 4-chloro-7-vinyl-7*H*-pyrrolo[2,3-*d*]pyrimidine (20)

CDCl$_3$, 298 K

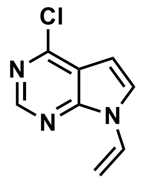

20

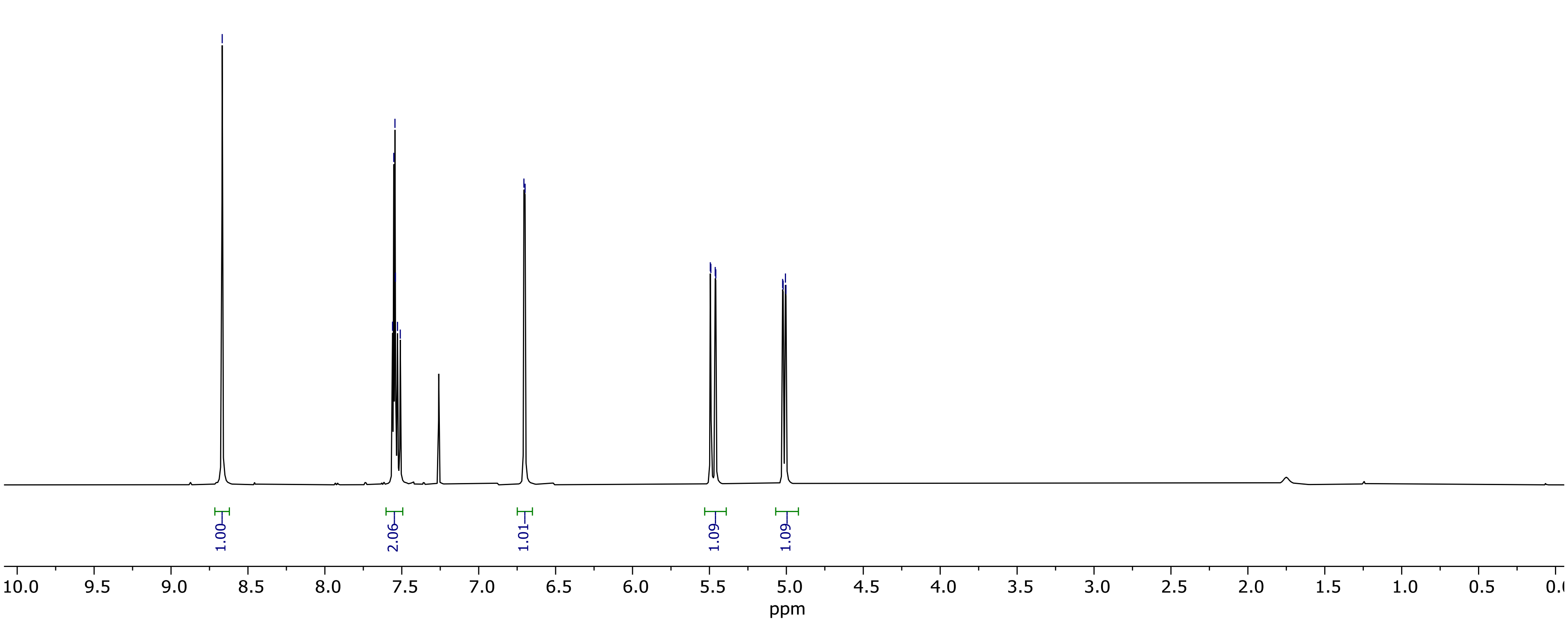

^{13}C NMR of 4-chloro-7-vinyl-7*H*-pyrrolo[2,3-*d*]pyrimidine (20)

CDCl$_3$, 298 K

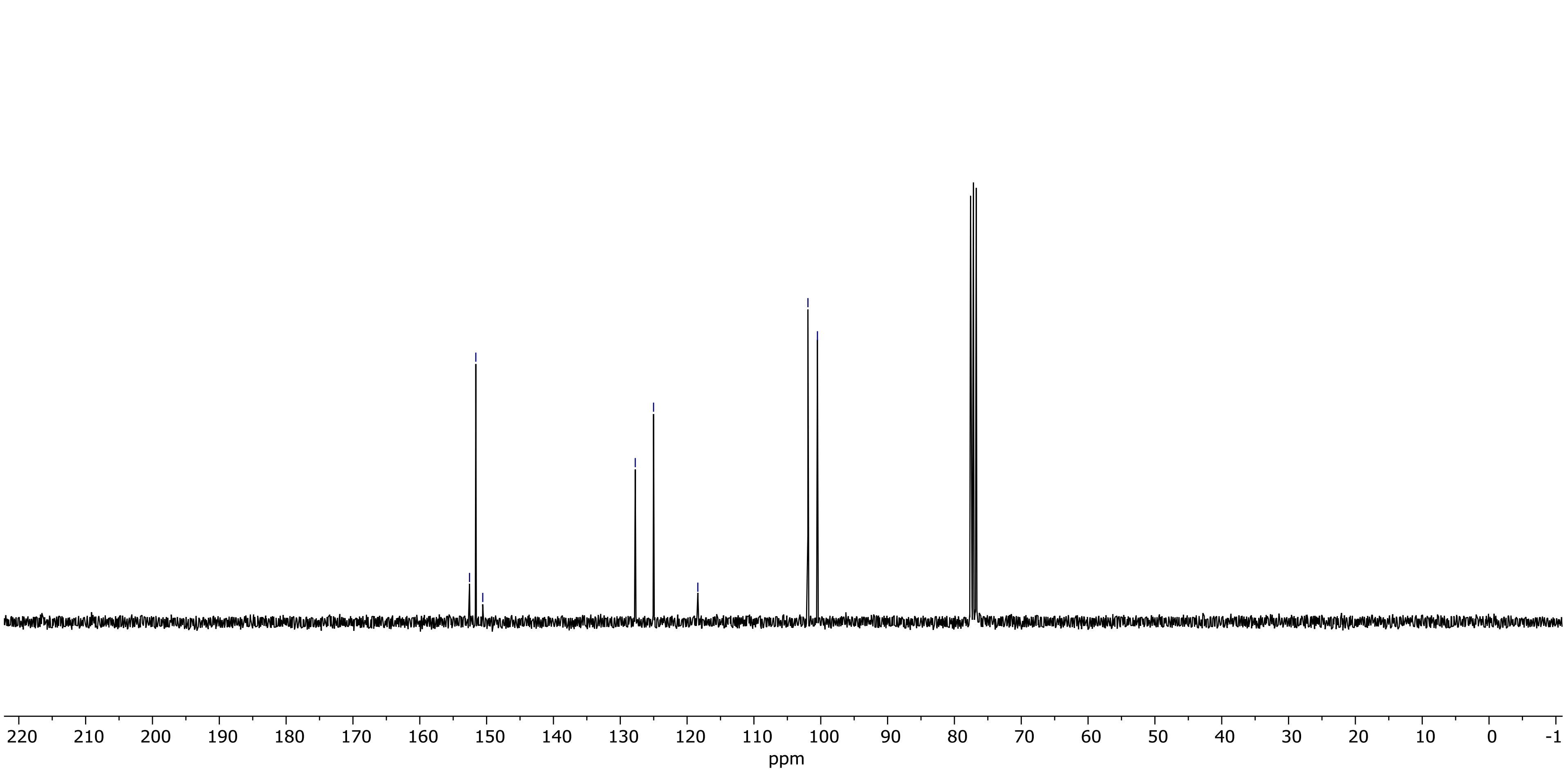

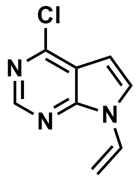

20

^{1}H NMR of *N*-vinyl-theophylline (21)

CDCl$_3$, 298 K

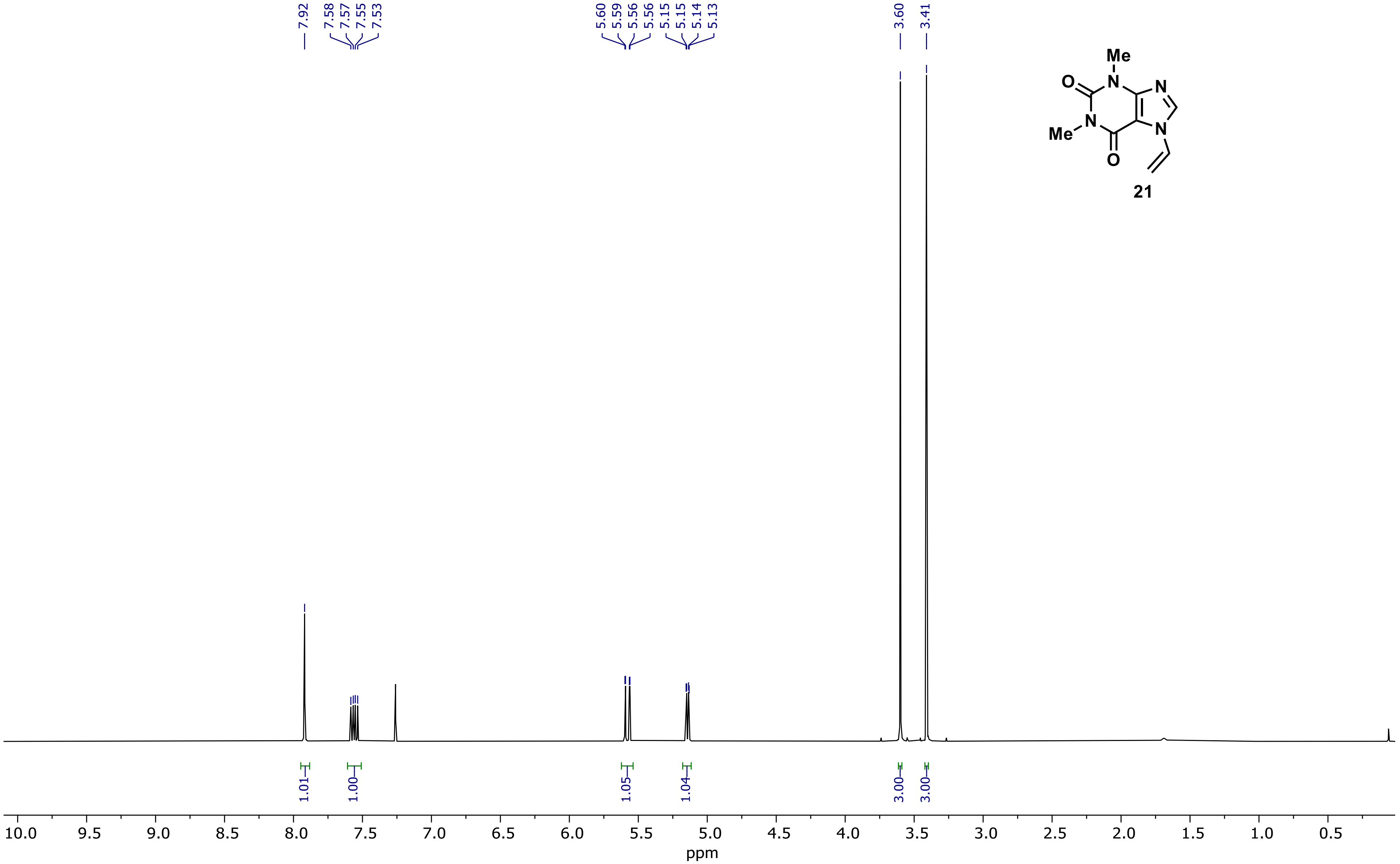

^{13}C NMR of *N*-vinyl-theophylline (21)

$CDCl_3$, 298 K

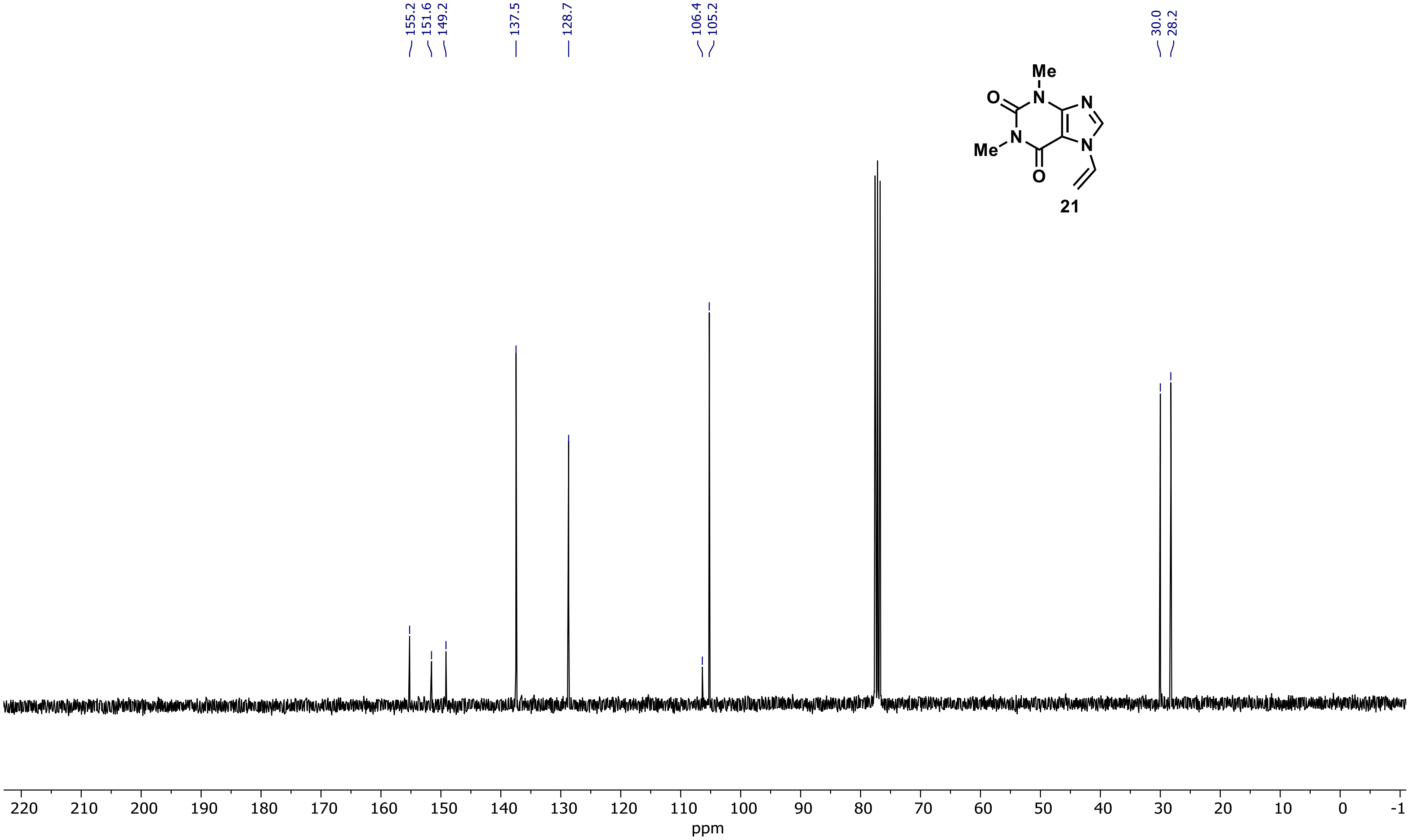

^{1}H NMR of *N*-vinyl-*N'*-acetyl-L-tryptophan ethyl ester (22)

CDCl$_3$, 298 K

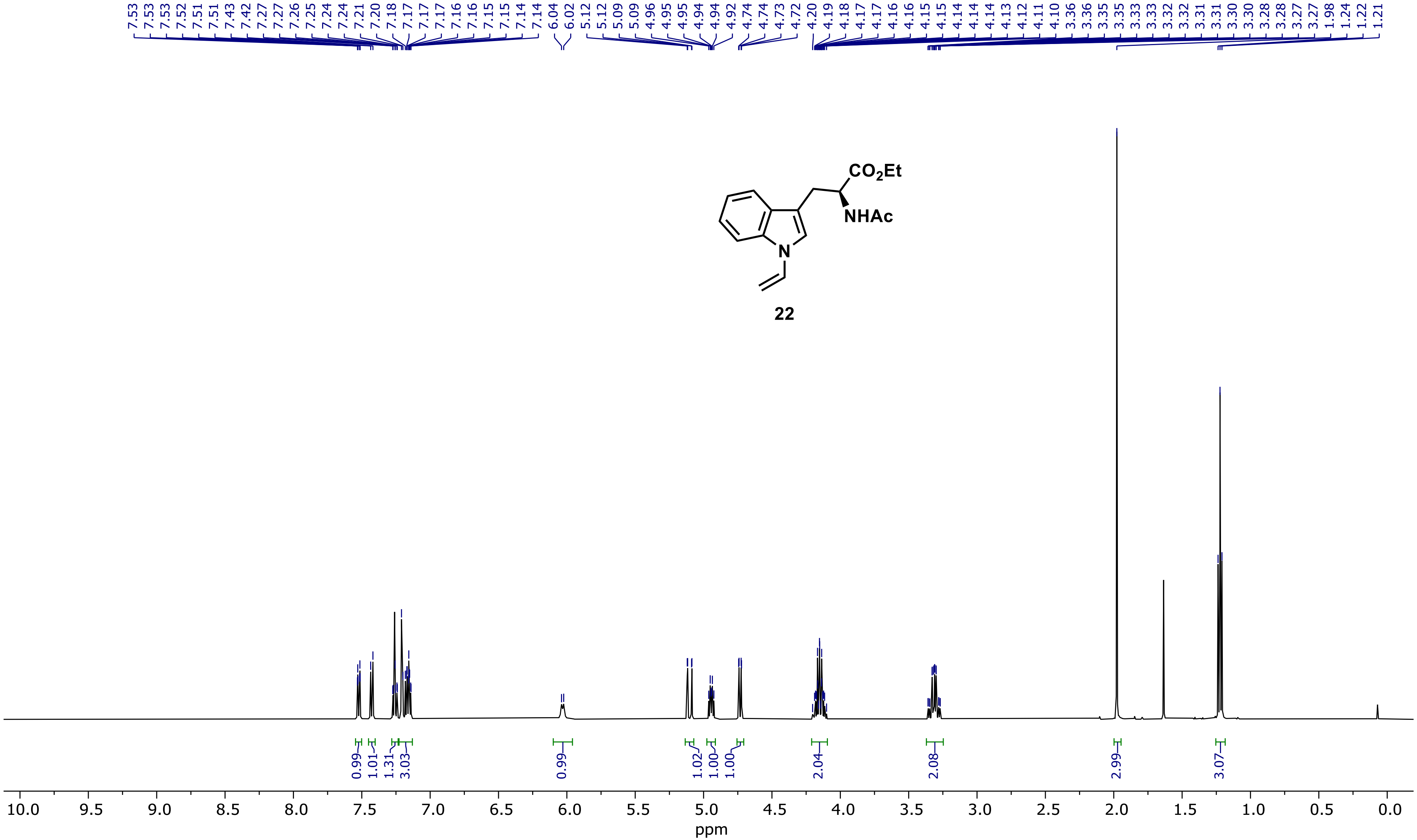

^{13}C NMR of *N*-vinyl-*N'*-acetyl-L-tryptophan ethyl ester (22)

$CDCl_3$, 298 K

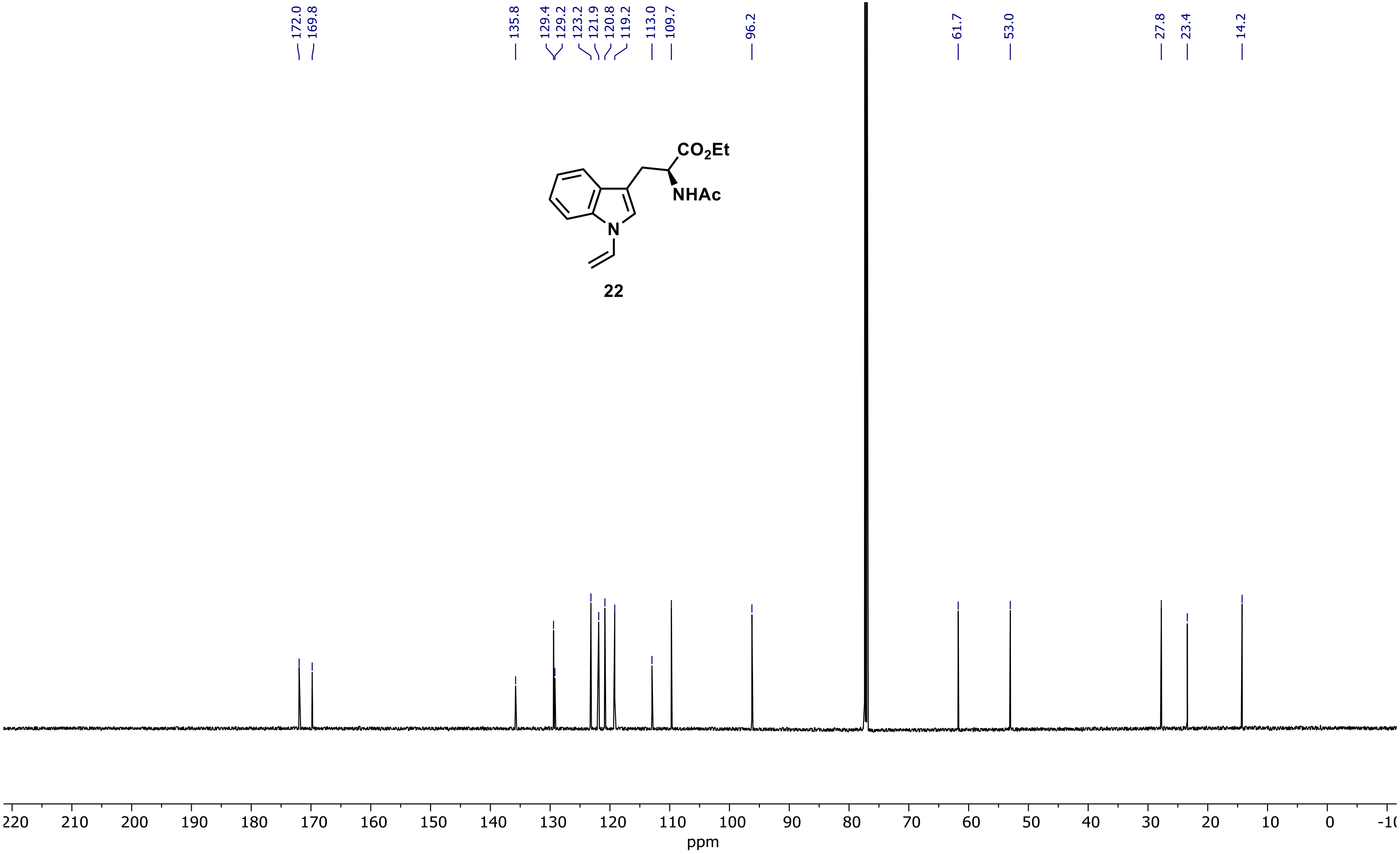

^{1}H NMR of *N*-vinyl-*N'*-Boc-L-histidine methyl ester (23)

$CDCl_3$, 298 K

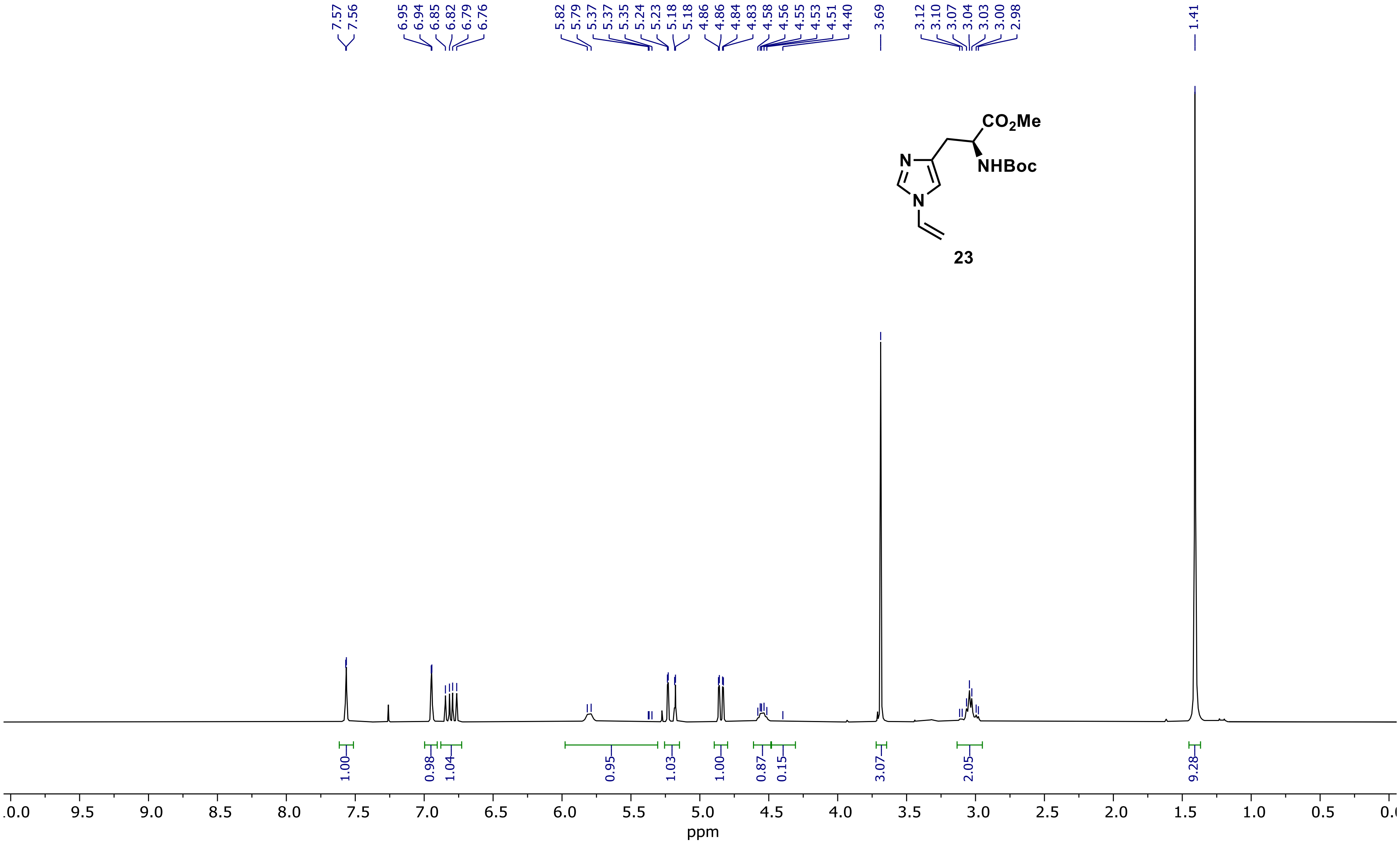

^{13}C NMR of *N*-vinyl-*N'*-Boc-L-histidine methyl ester (23)

$CDCl_3$, 298 K

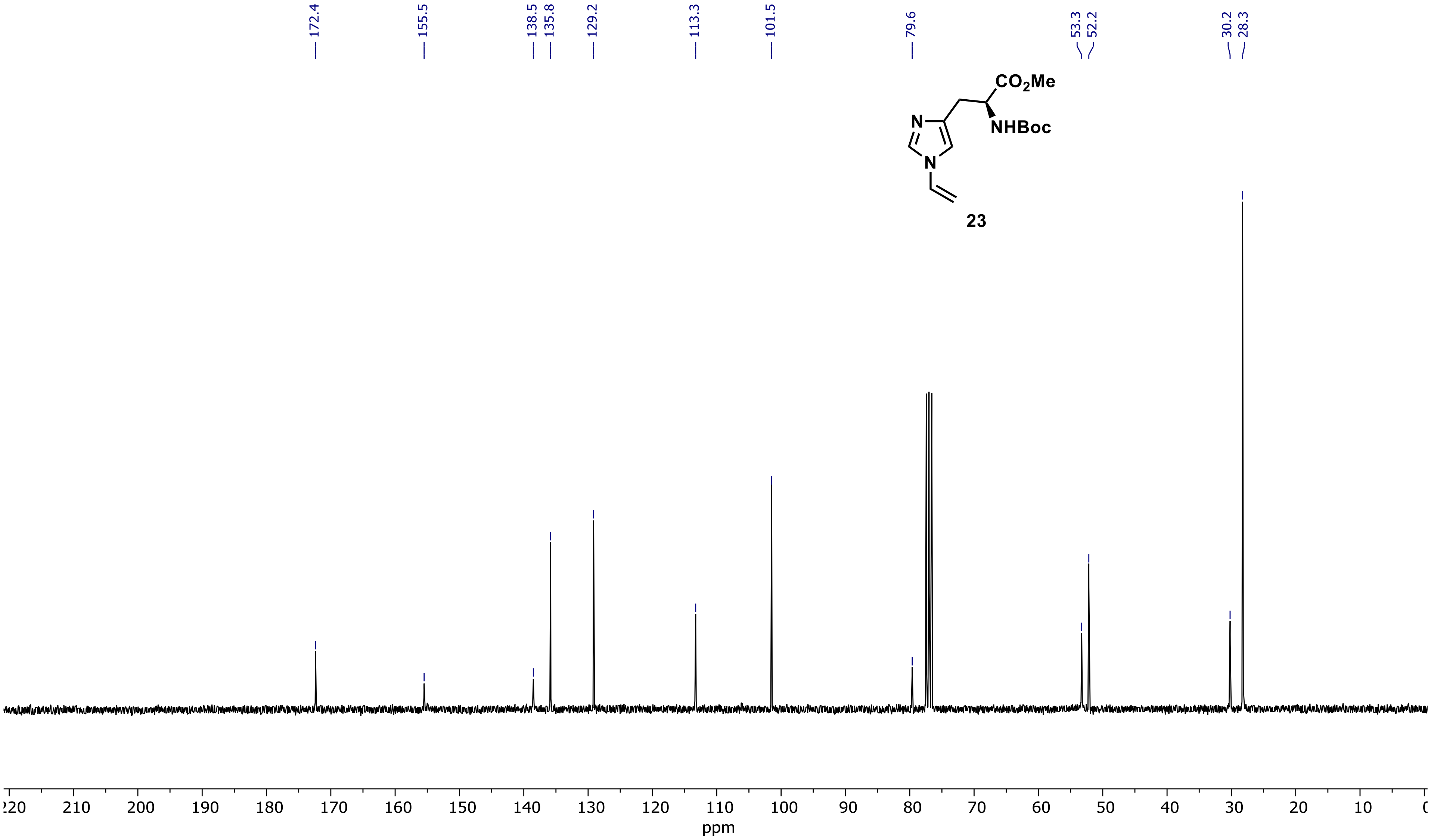

^{1}H NMR of *N*-vinyl-metaxalone (24)

$CDCl_3$, 298 K

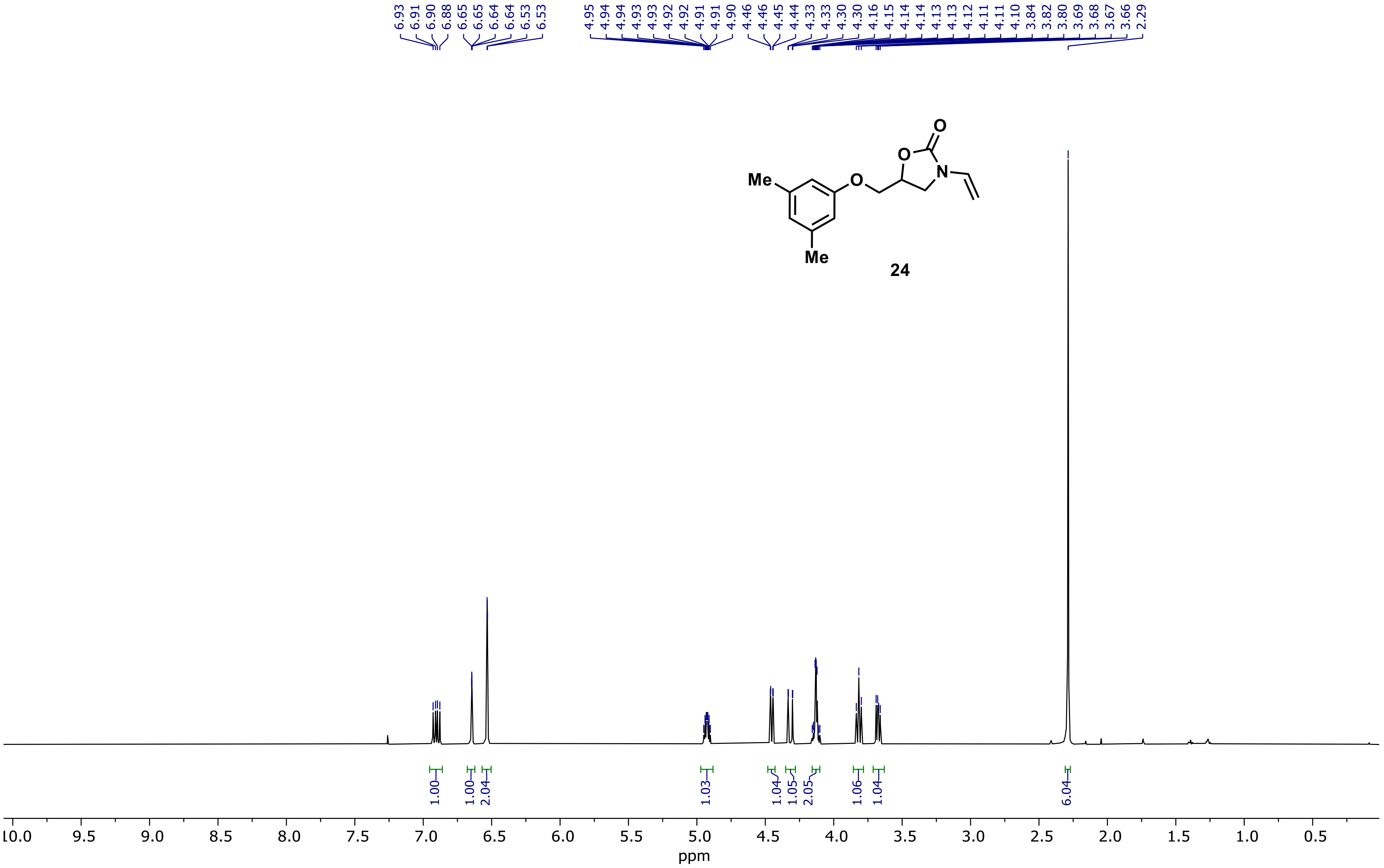

^{13}C NMR of *N*-vinyl-metaxalone (24)

$CDCl_3$, 298 K

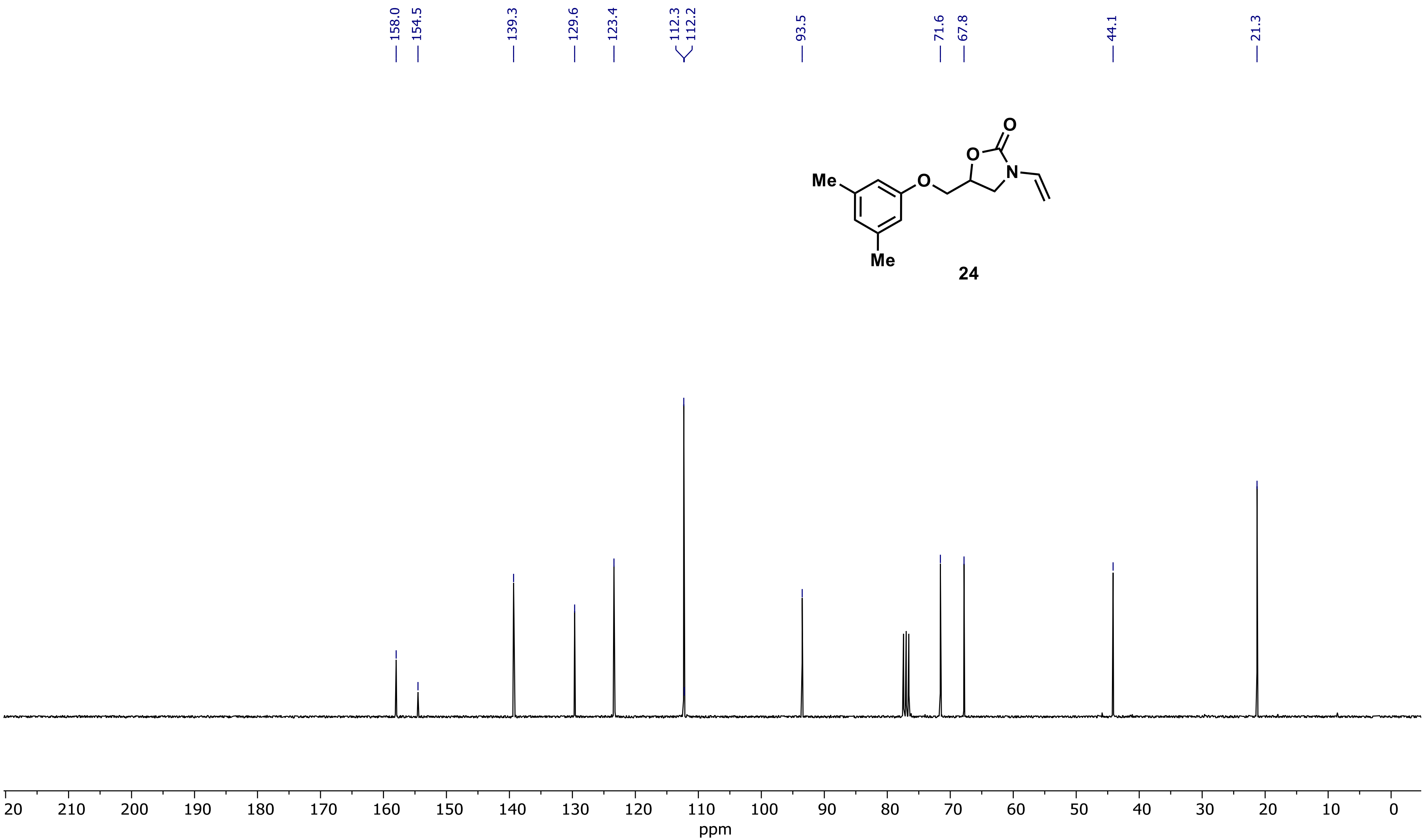

^{1}H NMR of *N*-vinyl-*N'*-Boc-carvedilol (25)

CD_2Cl_2, 298 K

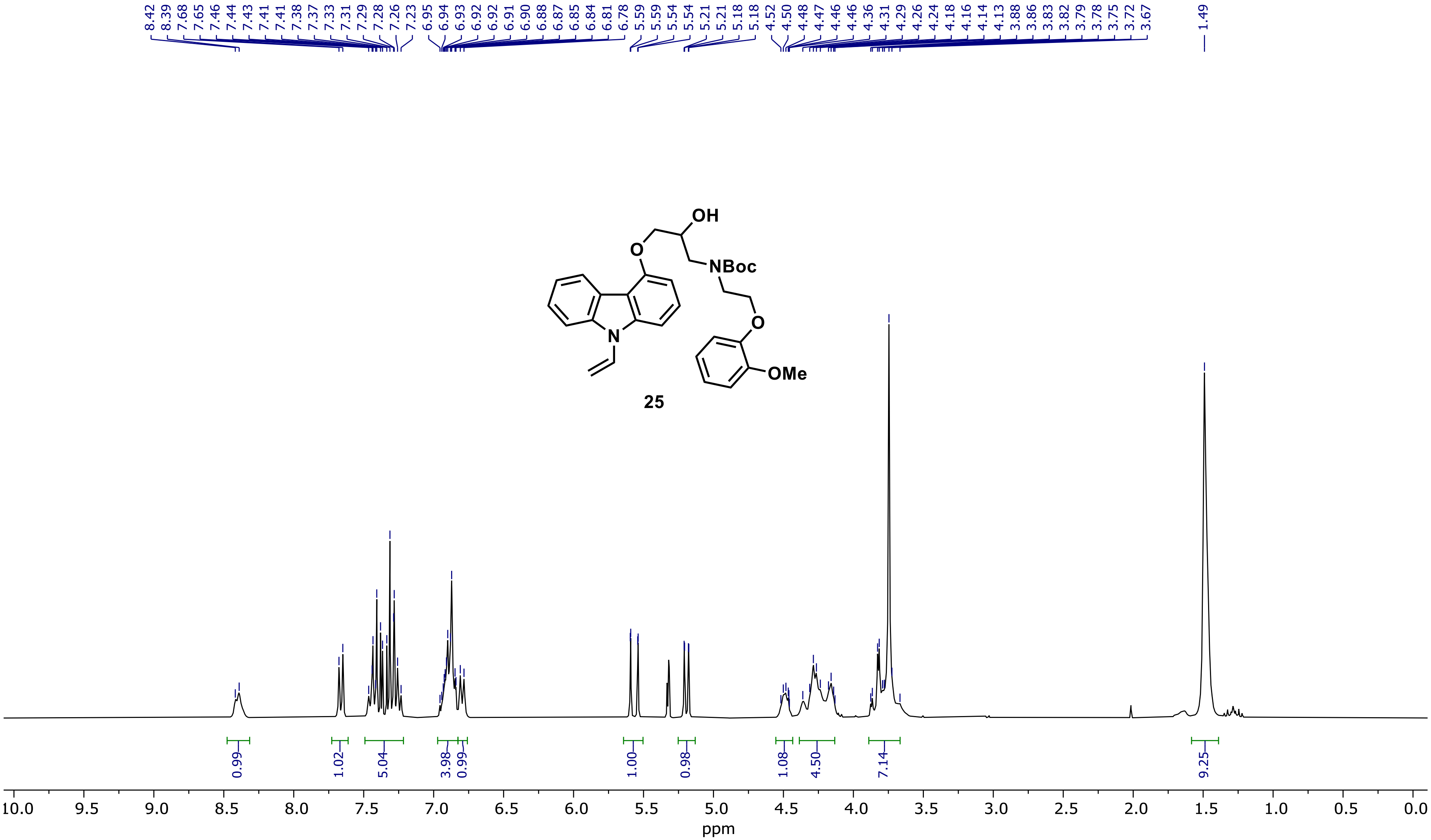

^{13}C NMR of *N*-vinyl-*N'*-Boc-carvedilol (25)

CD_2Cl_2, 298 K

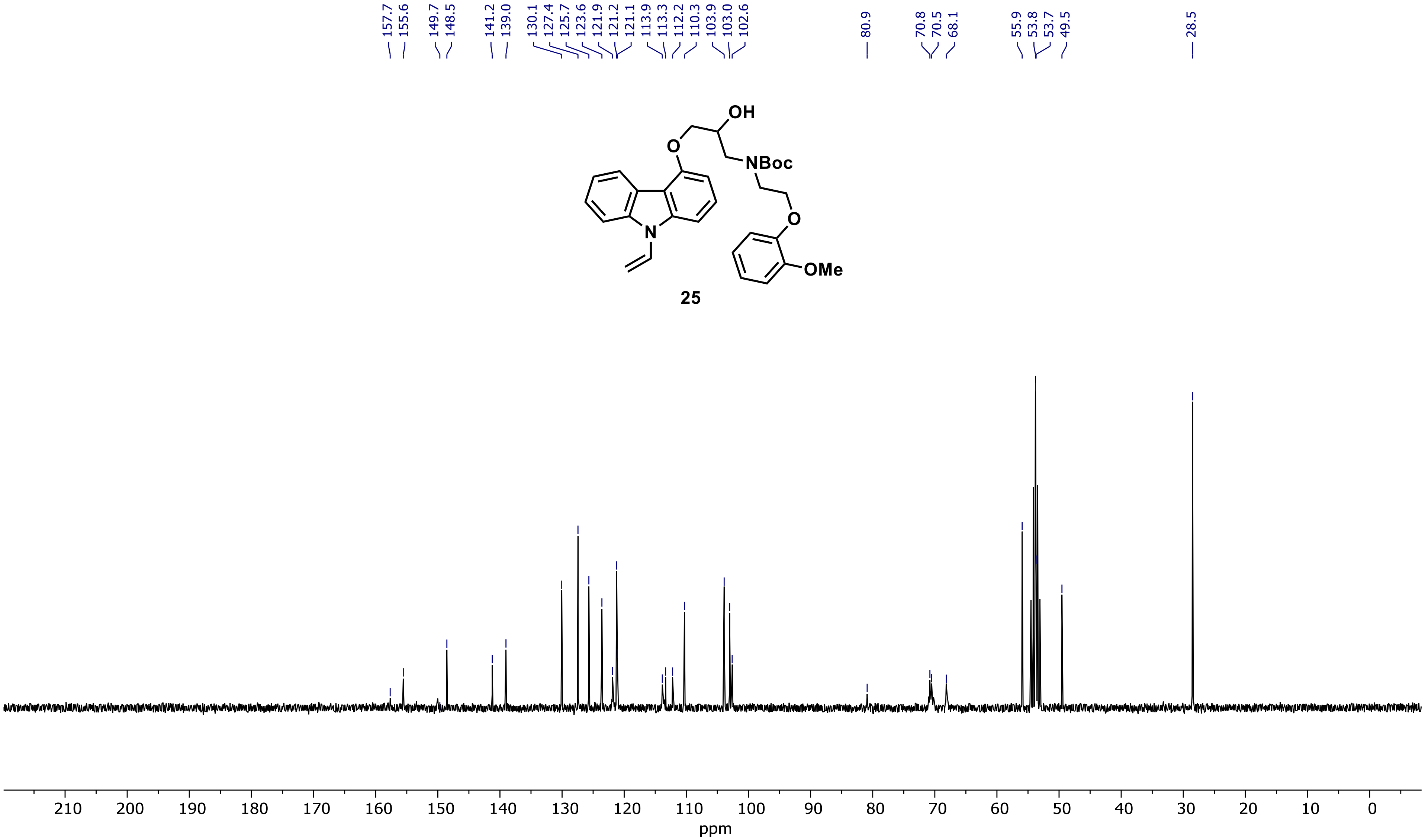

^{1}H NMR of *N*-vinyl-coumarin 7 (26)

$CDCl_3$, 298 K

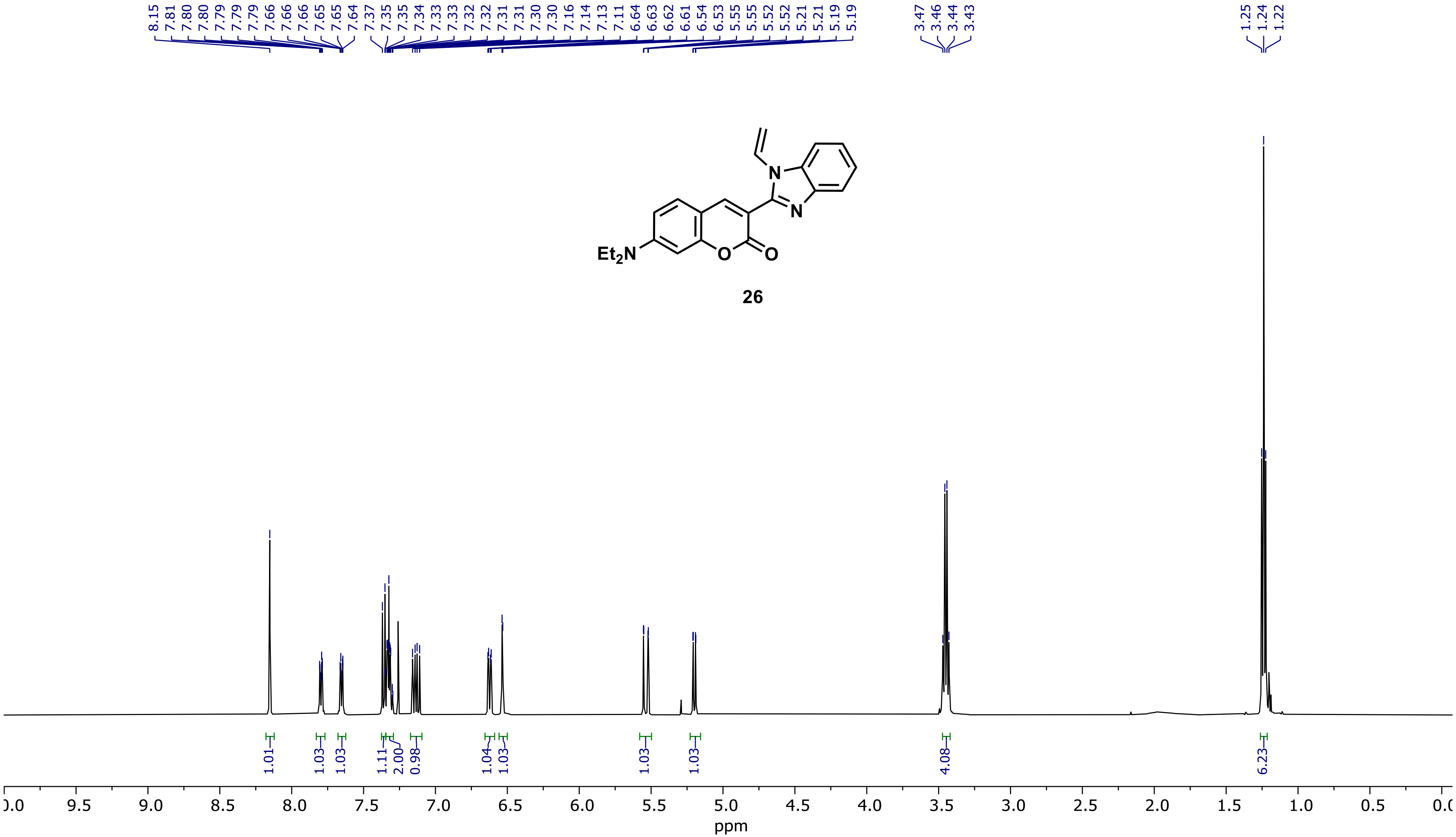

^{13}C NMR of *N*-vinyl-coumarin 7 (26)

CDCl$_3$, 298 K

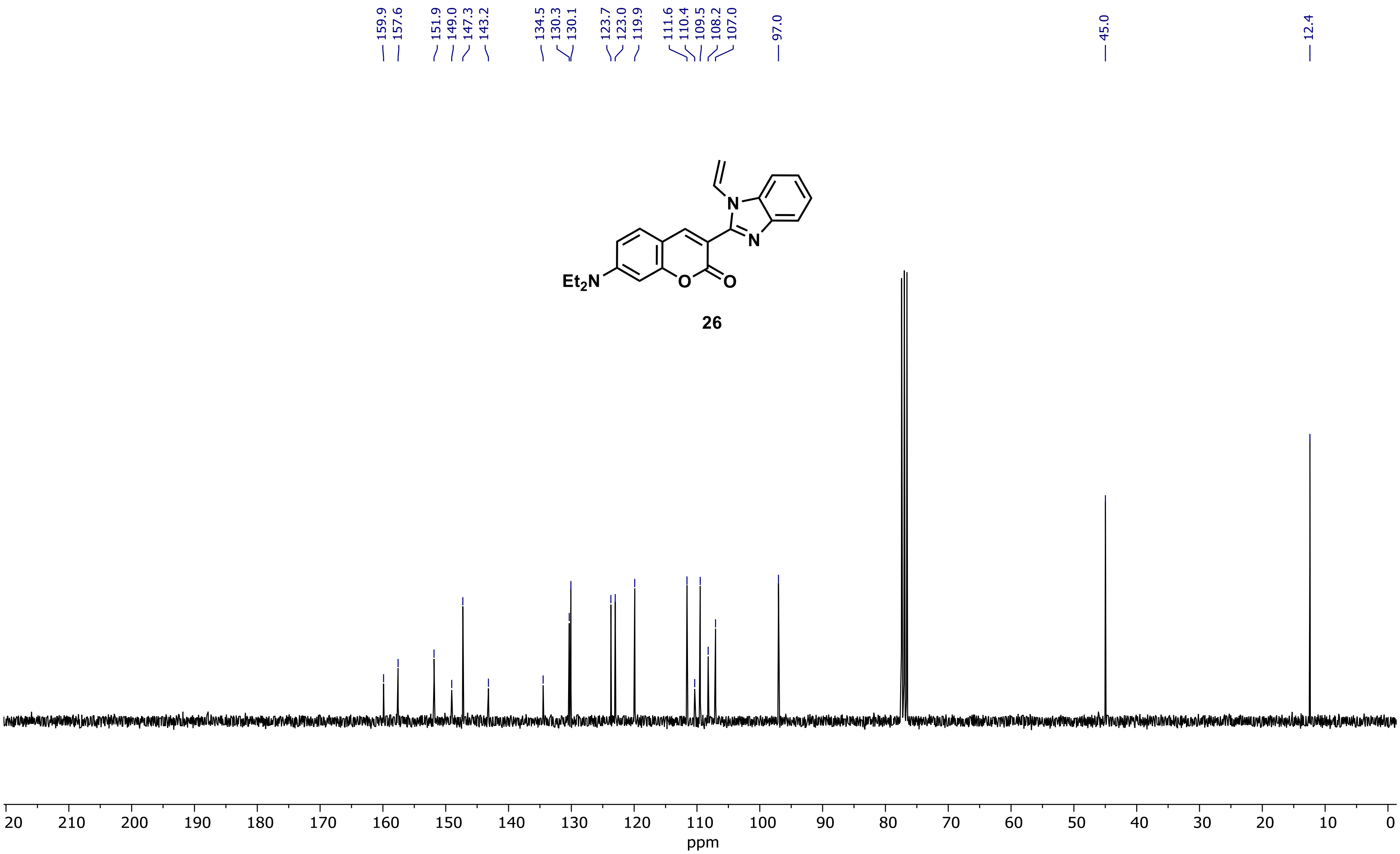

^{1}H NMR of *N*-vinyl-lansoprazole (27)

CD_2Cl_2, 298 K

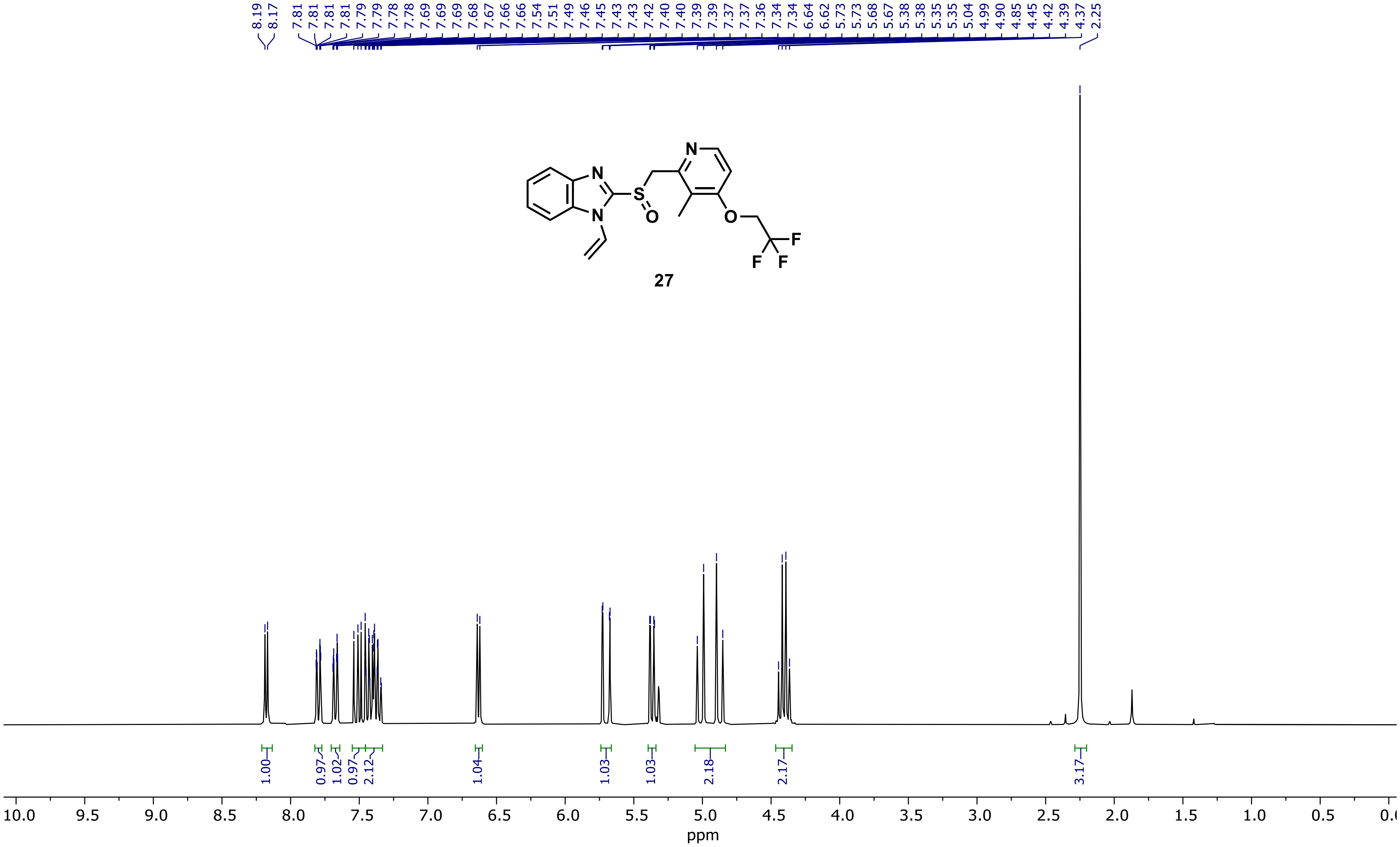

^{13}C NMR of *N*-vinyl-lansoprazole (27)

CD_2Cl_2, 298 K

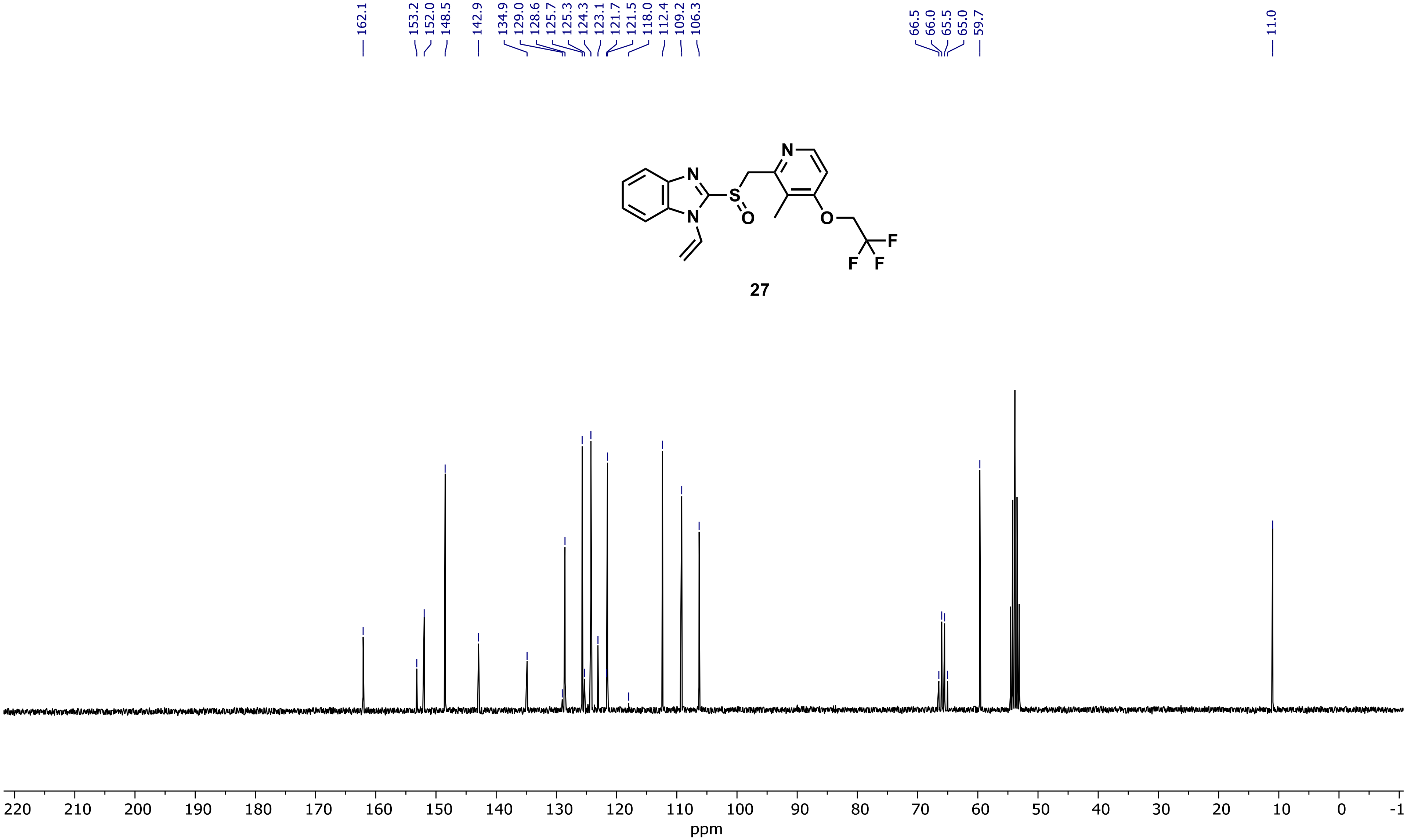

^{19}F NMR of *N*-vinyl-lansoprazole (27)

CD_2Cl_2, 298 K

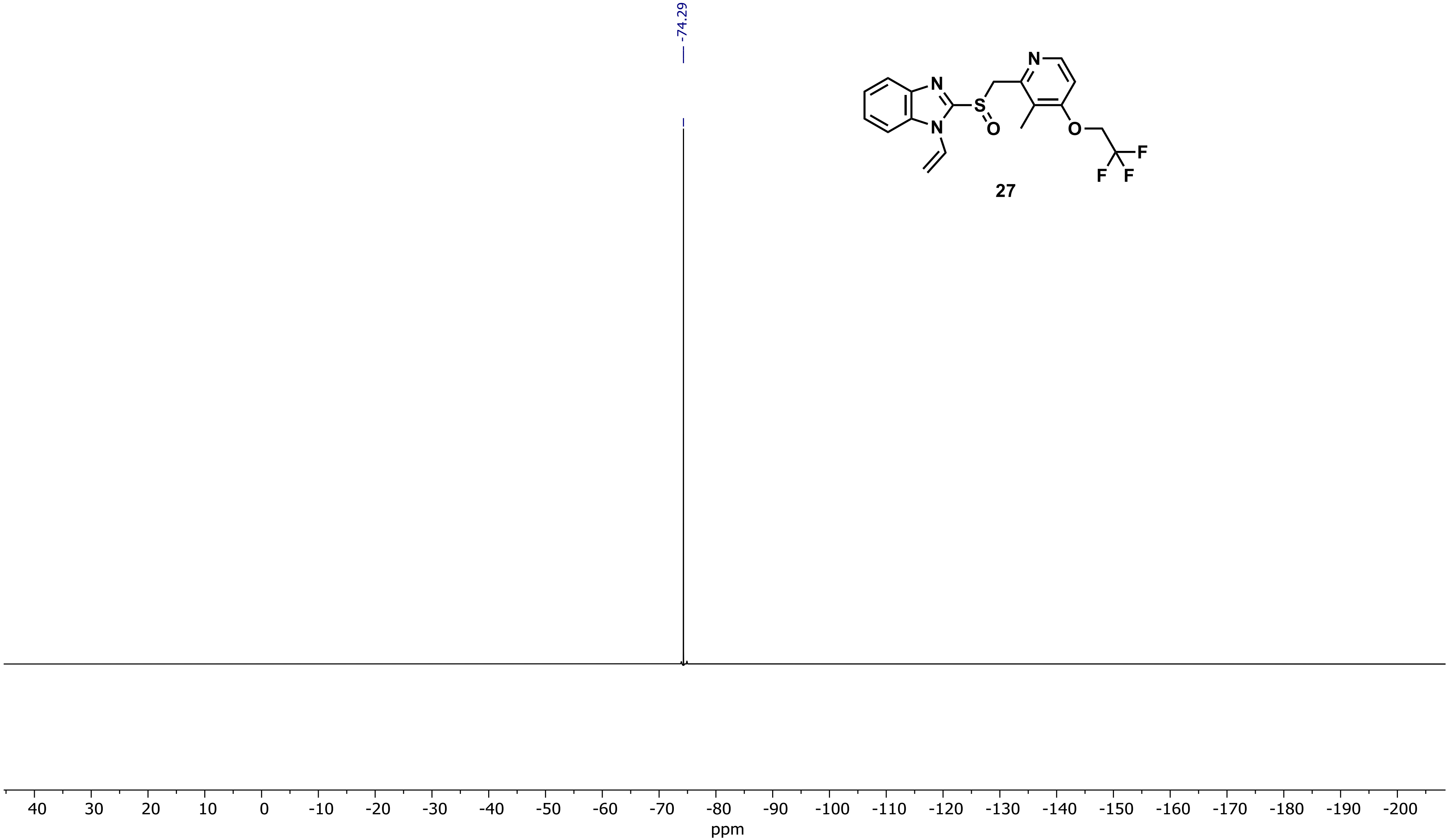

27

^{1}H NMR of 3-methyl-4-vinylbenzonitrile (29)

CDCl$_3$, 298 K

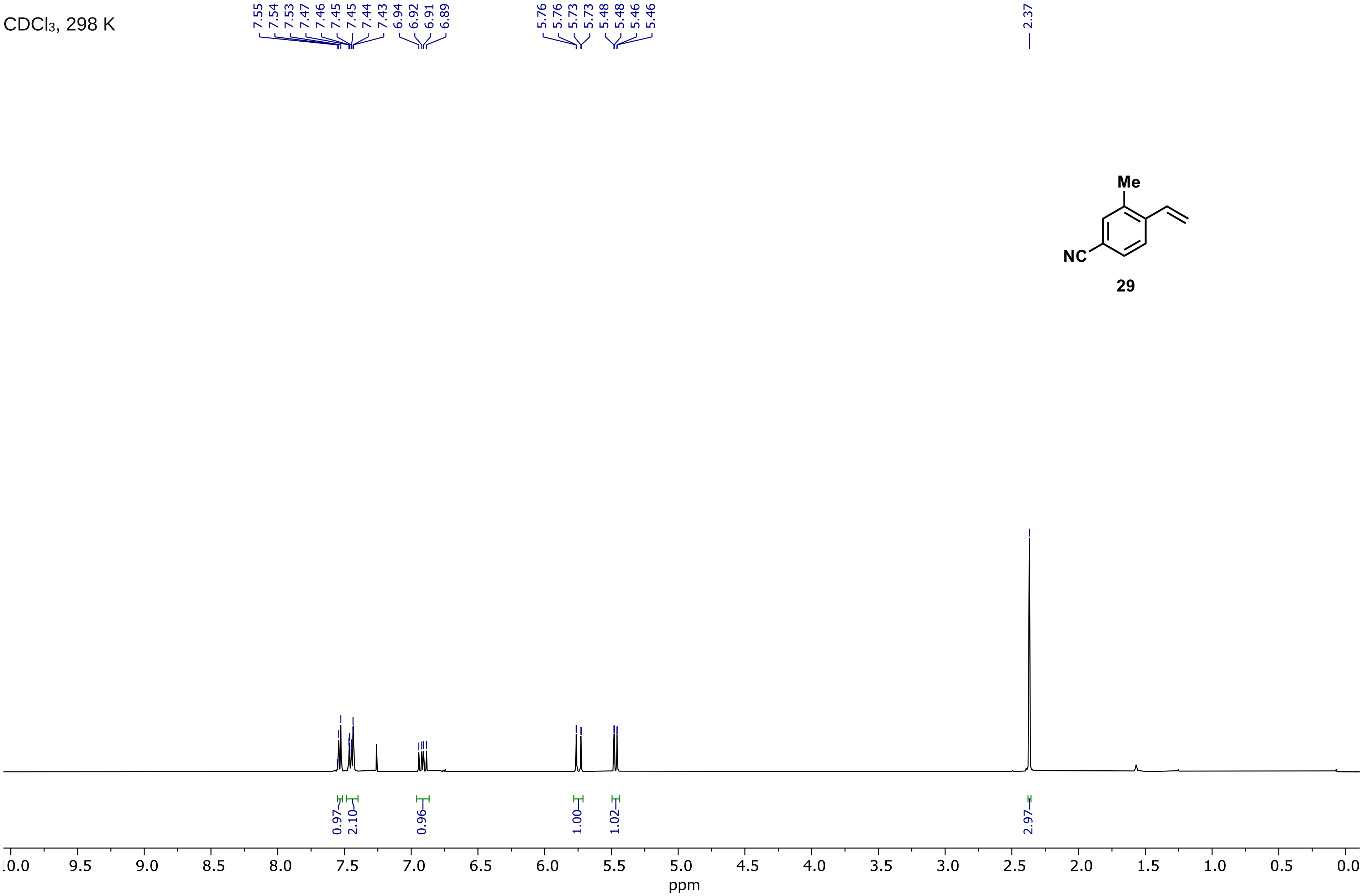

29

^{13}C NMR of 3-methyl-4-vinylbenzonitrile (29)

CDCl$_3$, 298 K

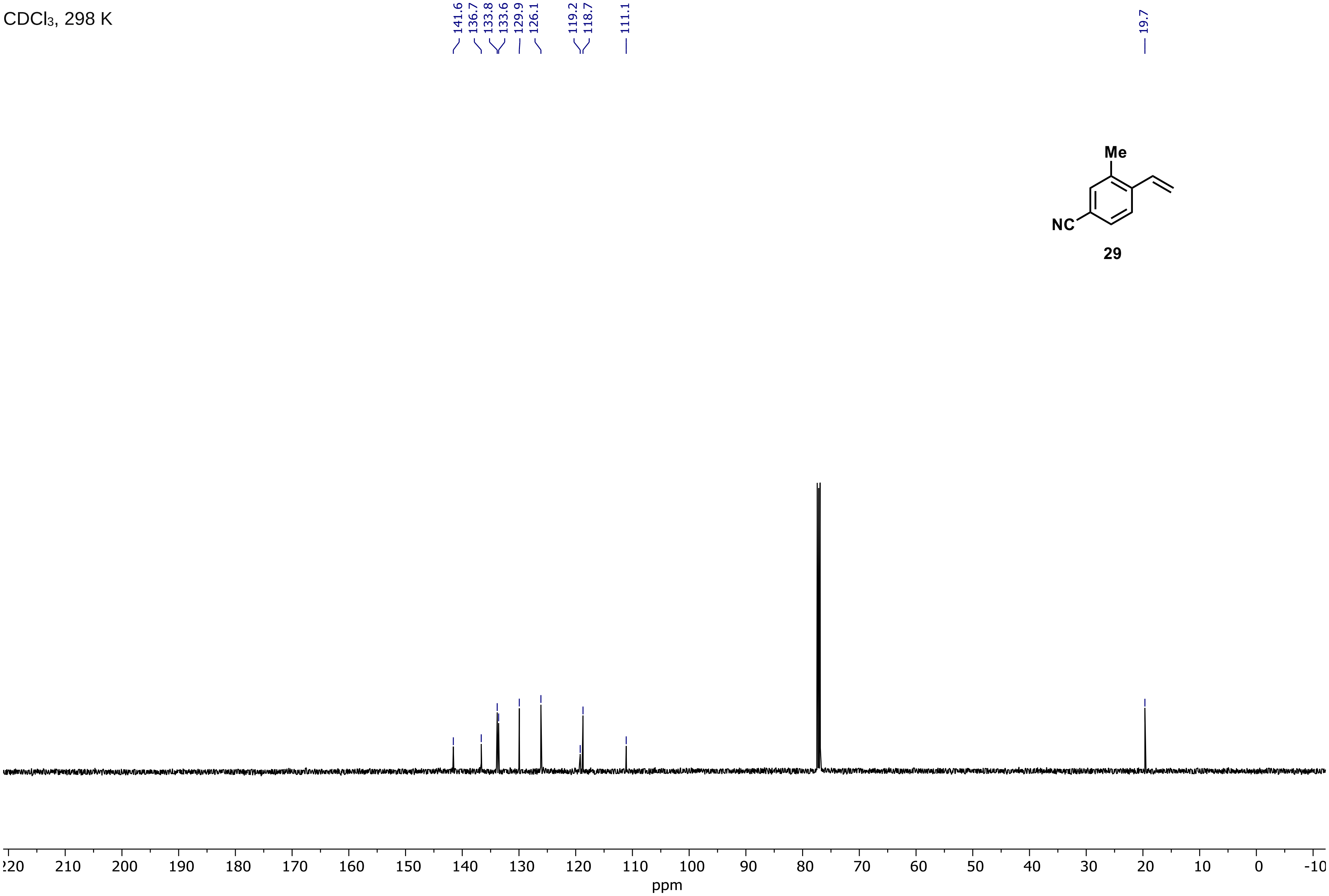

^{1}H NMR of 1-chloro-4-methoxy-2-vinylbenzene (30)

$CDCl_3$, 298 K

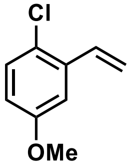

30

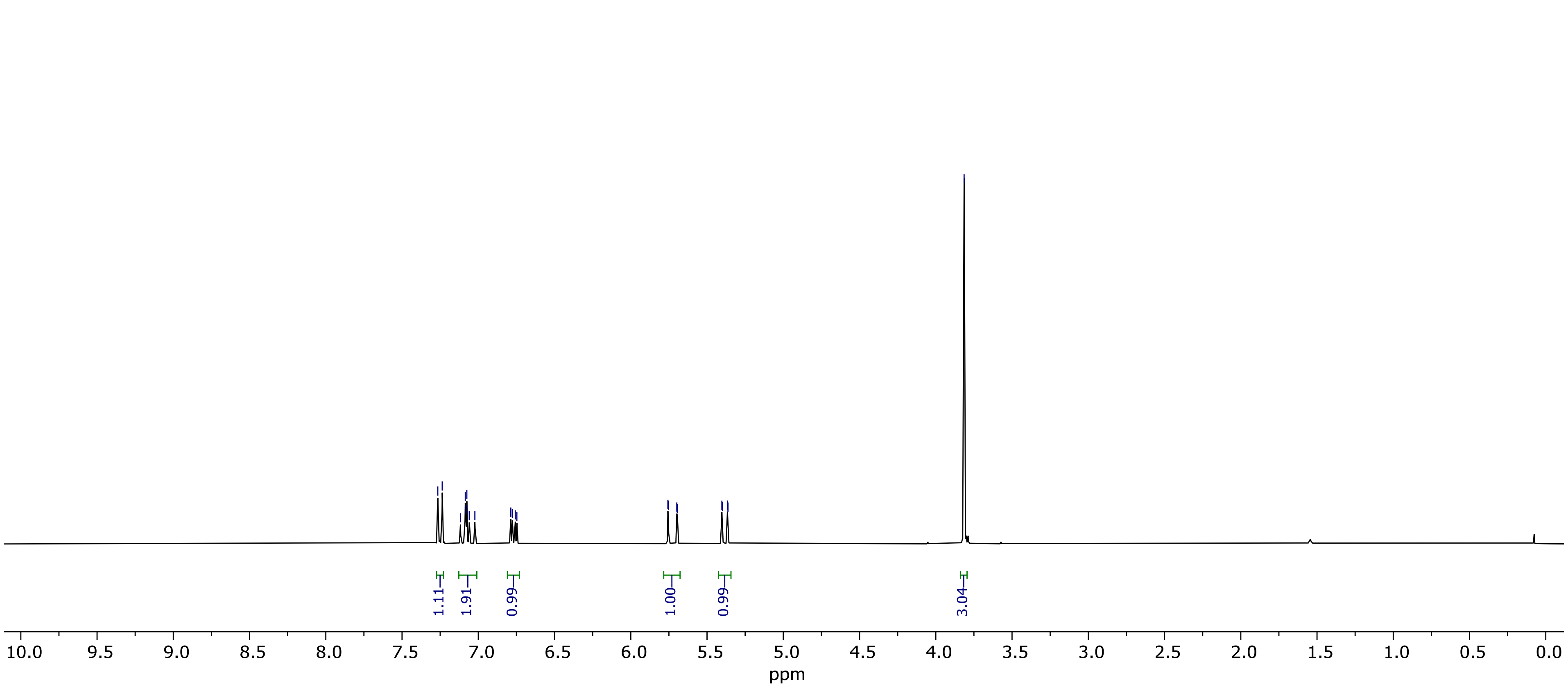

^{13}C NMR of 1-chloro-4-methoxy-2-vinylbenzene (30)

CDCl$_3$, 298 K

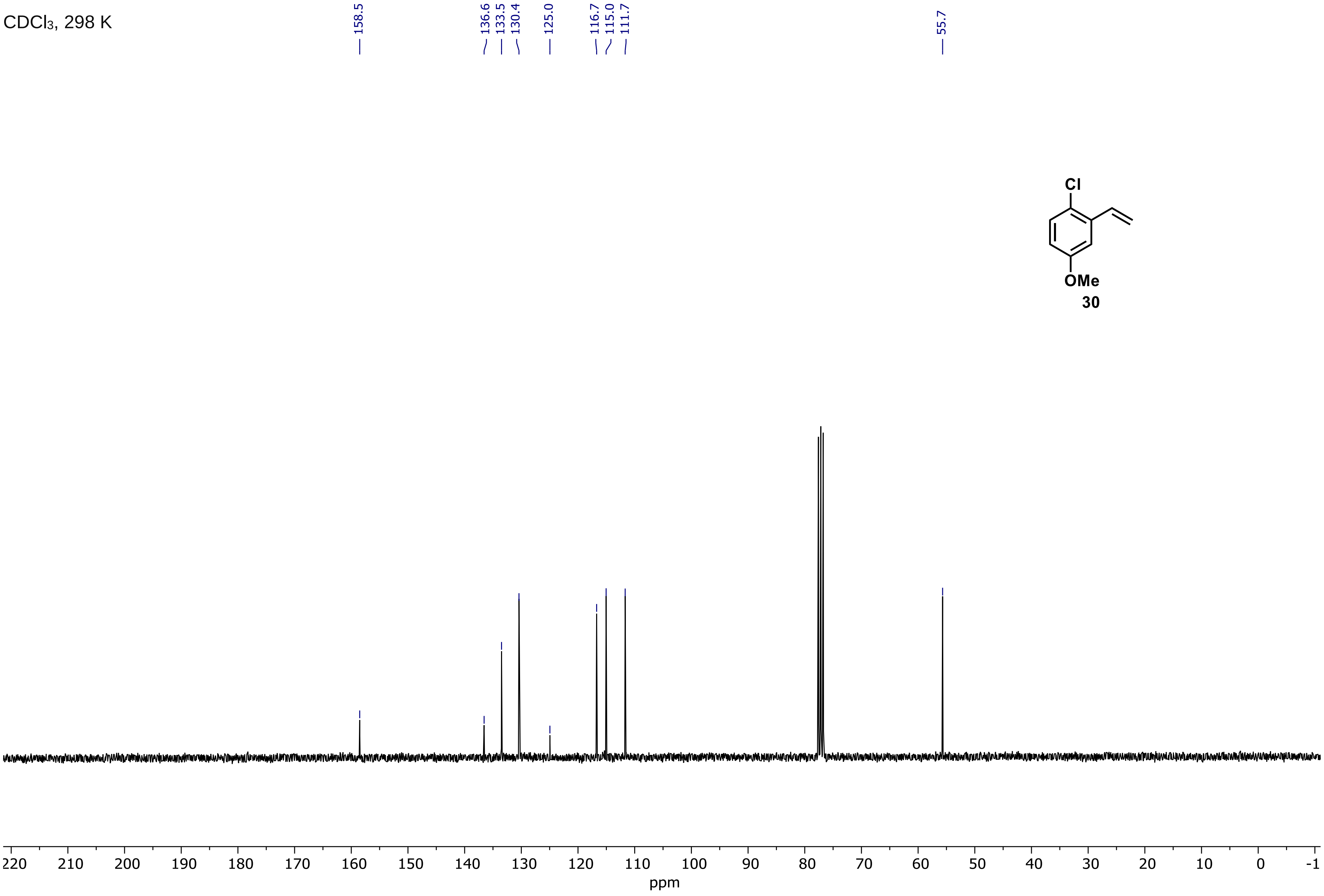

30

^{1}H NMR of 1-chloro-4-vinylbenzene (31)

CDCl$_3$, 298 K

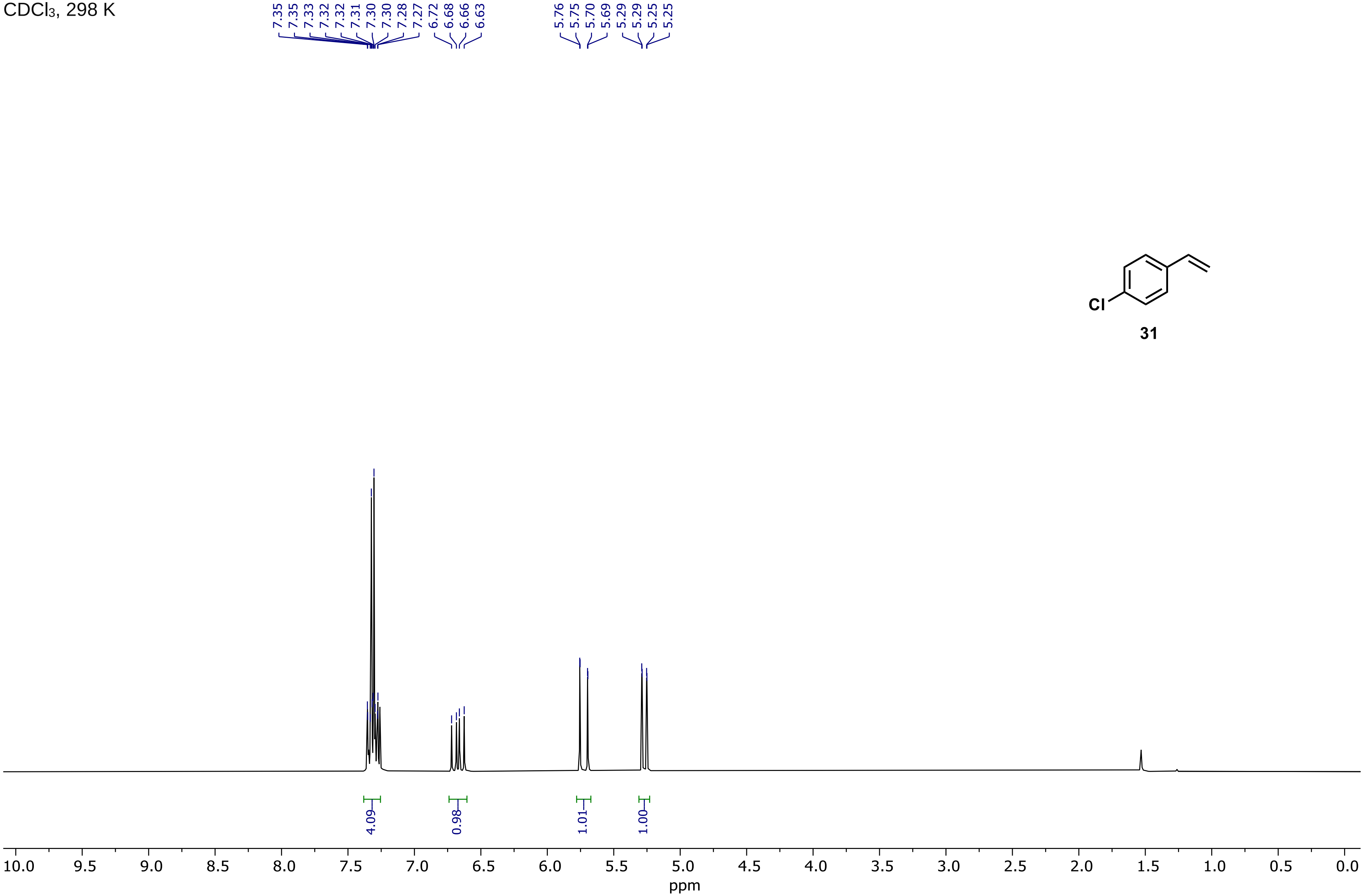

31

^{13}C NMR of 1-chloro-4-vinylbenzene (31)

$CDCl_3$, 298 K

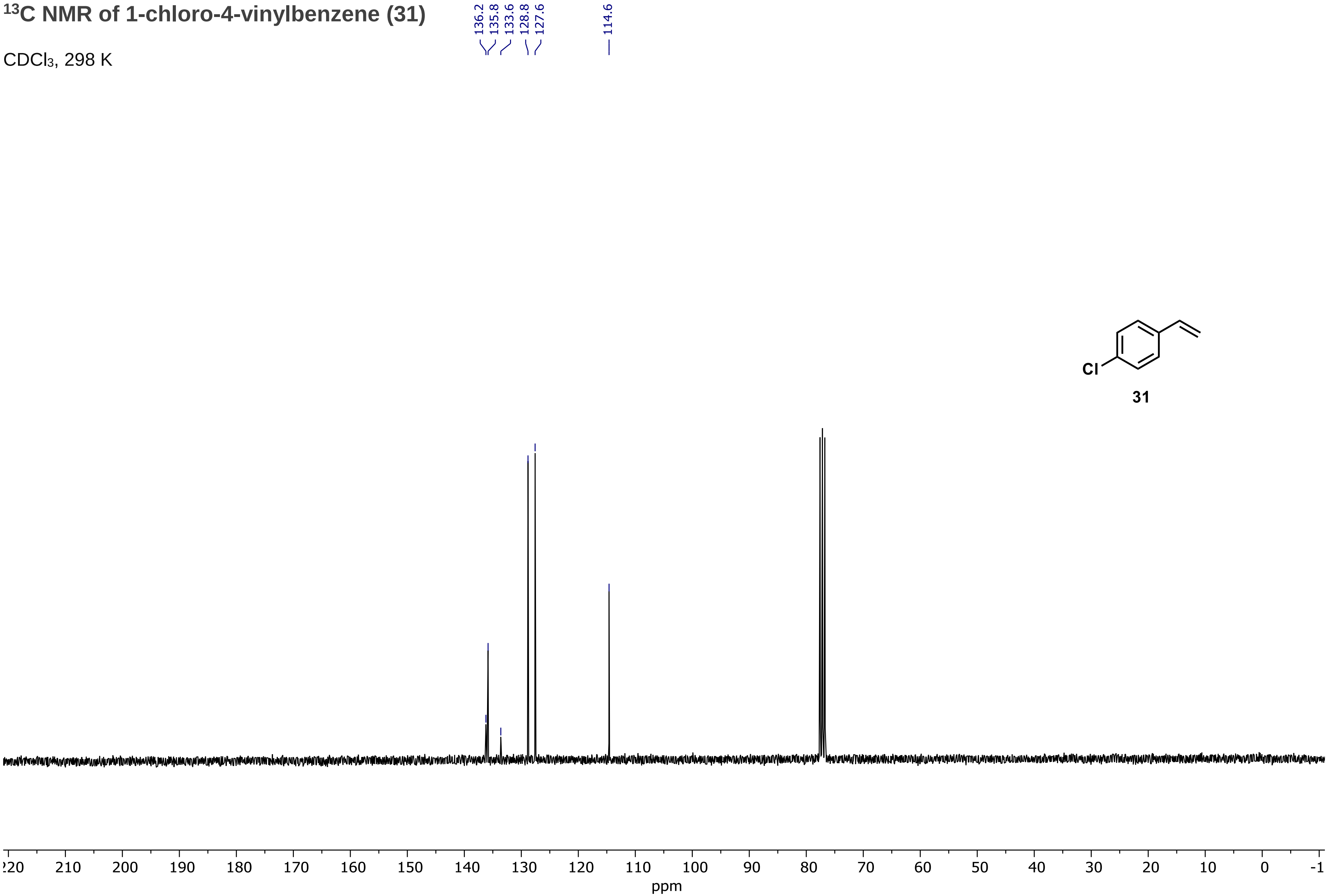

31

^{1}H NMR of 1-chloro-4-(vinyl-d_3)benzene (31-d_3)

CD_2Cl_2, 298 K

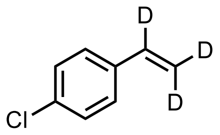

31-d_3

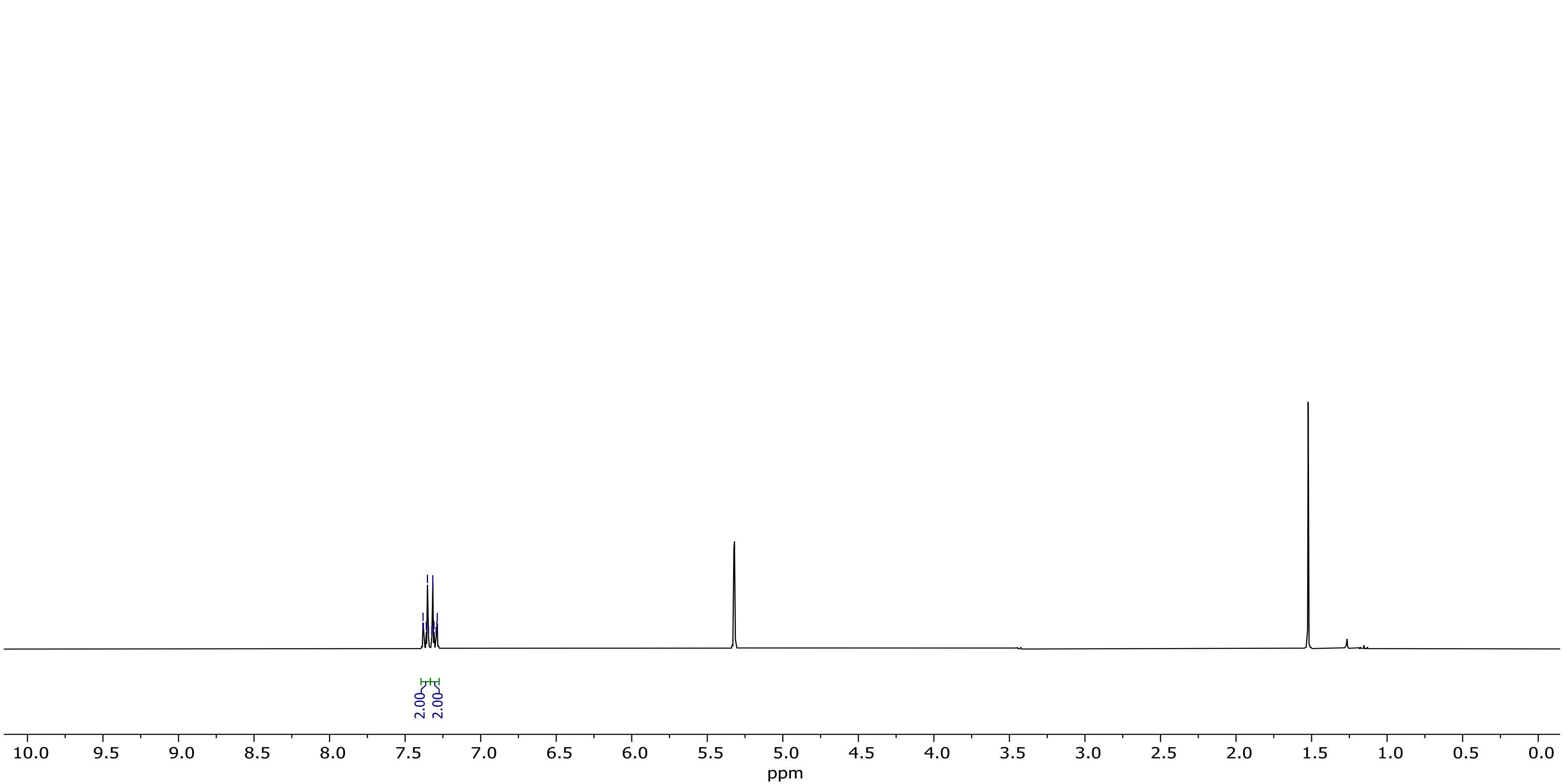

^{2}H NMR of 1-chloro-4-(vinyl-d_3)benzene (31-d_3)

CD_2Cl_2, 298 K

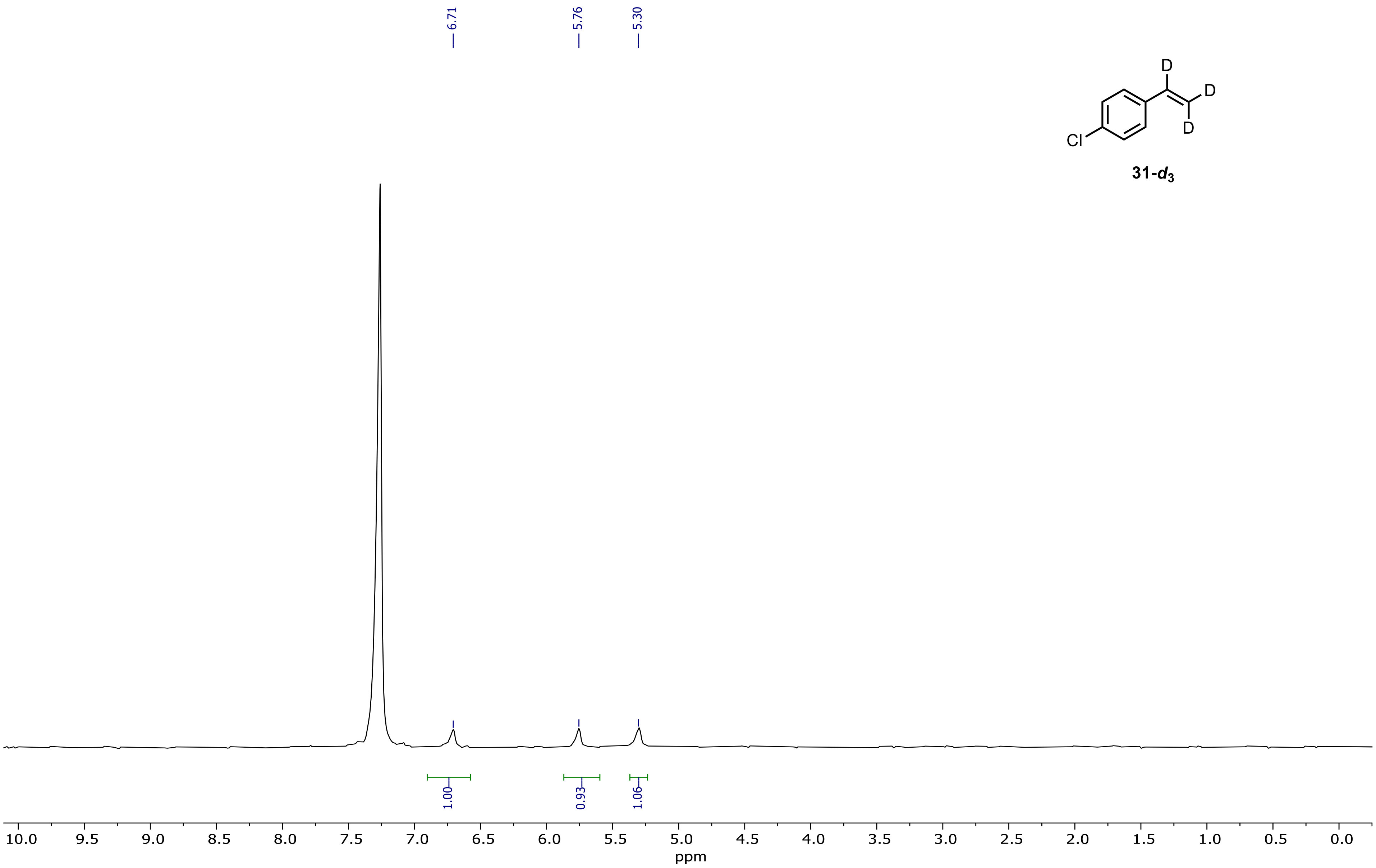

31-d_3

^{13}C NMR of 1-chloro-4-(vinyl-d_3)benzene (31-d_3)

CD_2Cl_2, 243 K

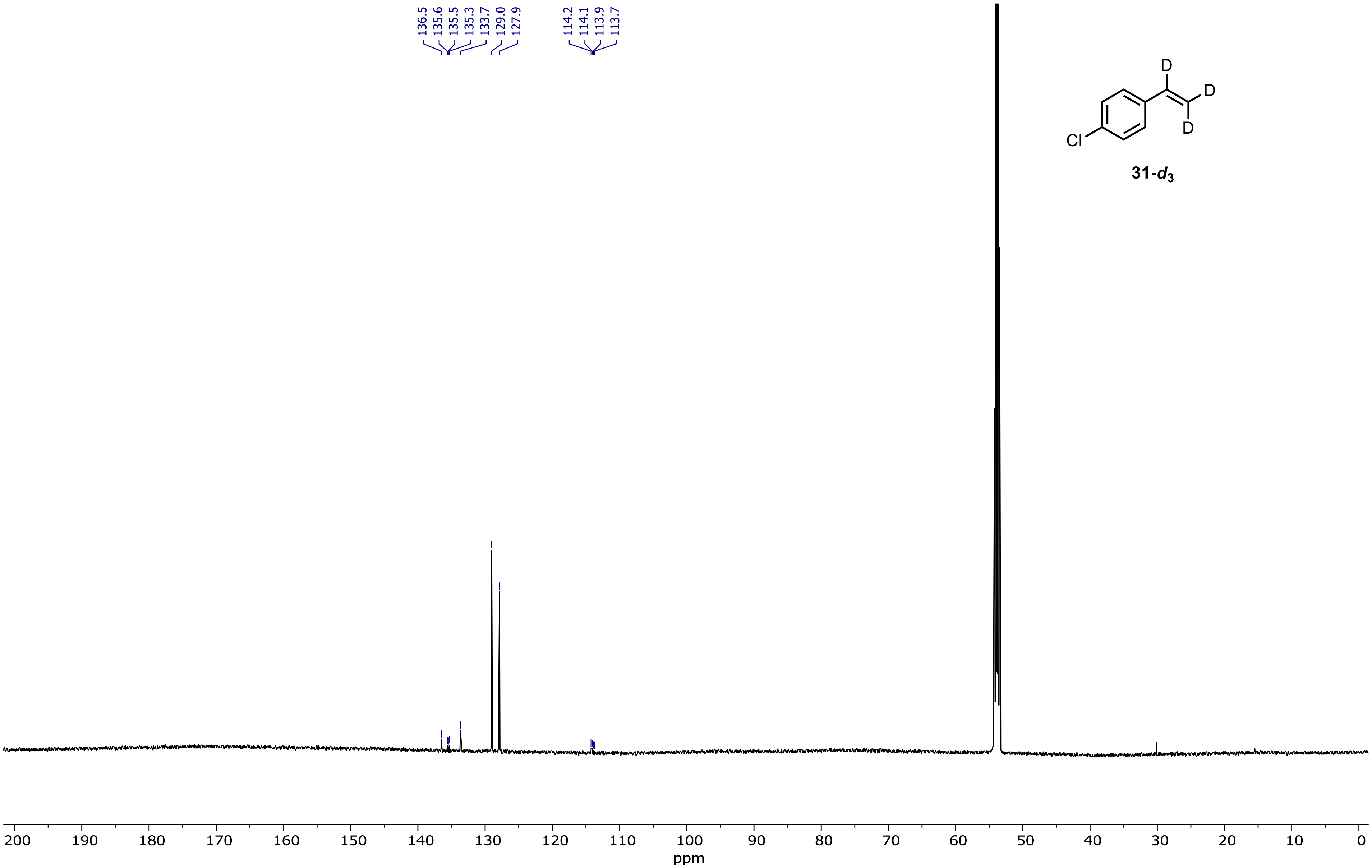

31-d_3

^{1}H NMR of 1-ethoxy-2-vinylbenzene (32)

CDCl₃, 298 K

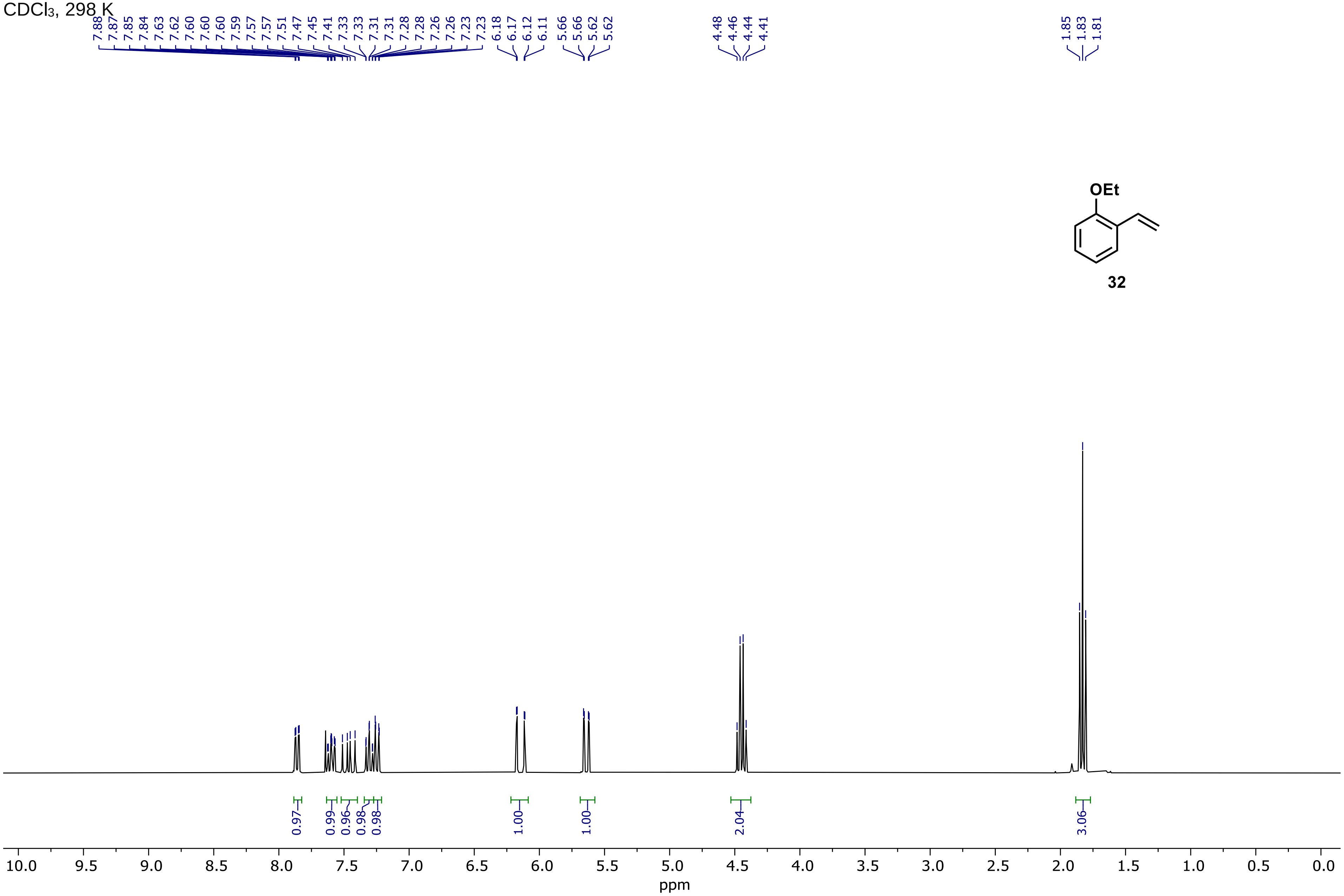

^{13}C NMR of 1-ethoxy-2-vinylbenzene (32)

$CDCl_3$, 298 K

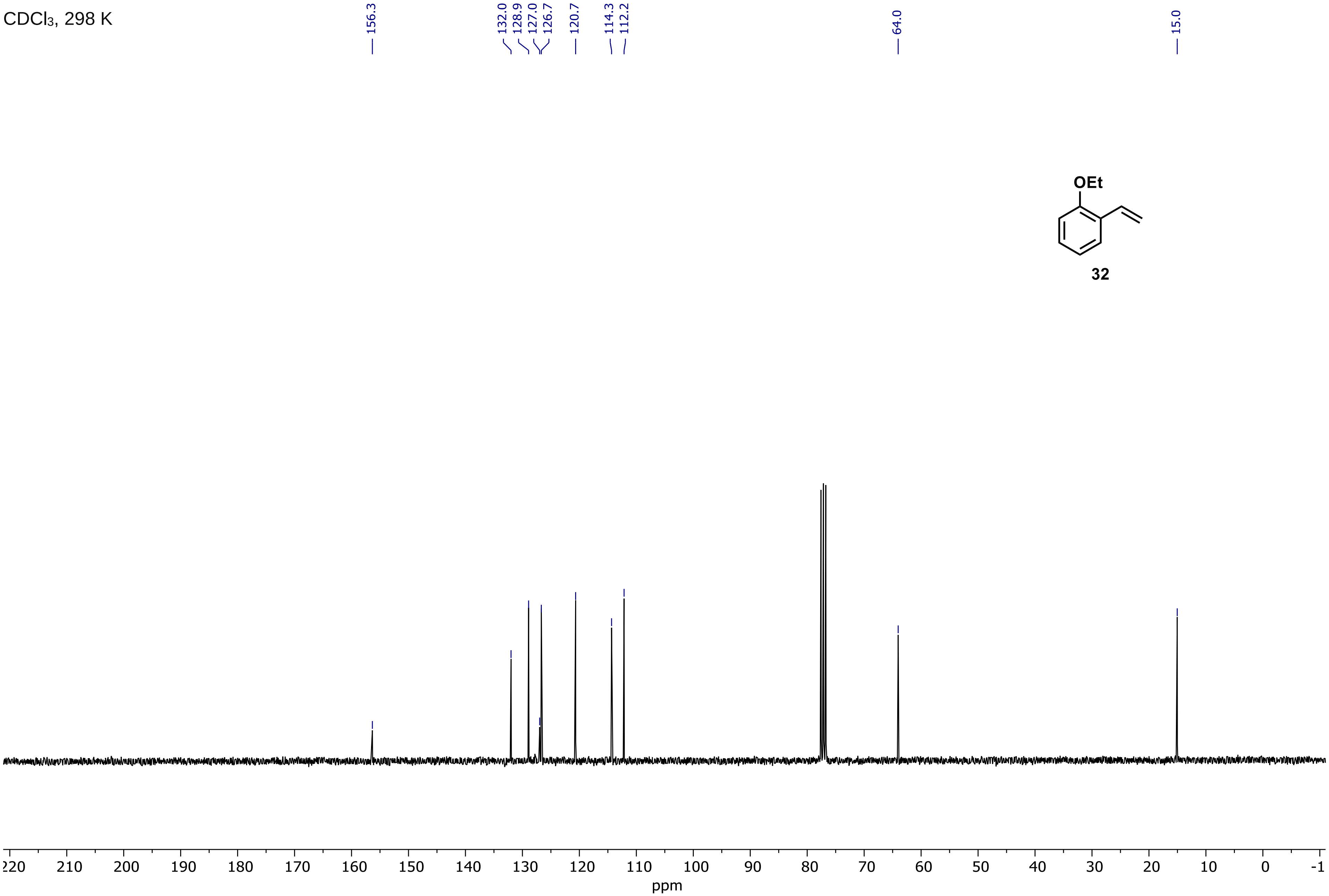

^{1}H NMR of 1-bromo-4-vinylbenzene (33)

$CDCl_3$, 298 K

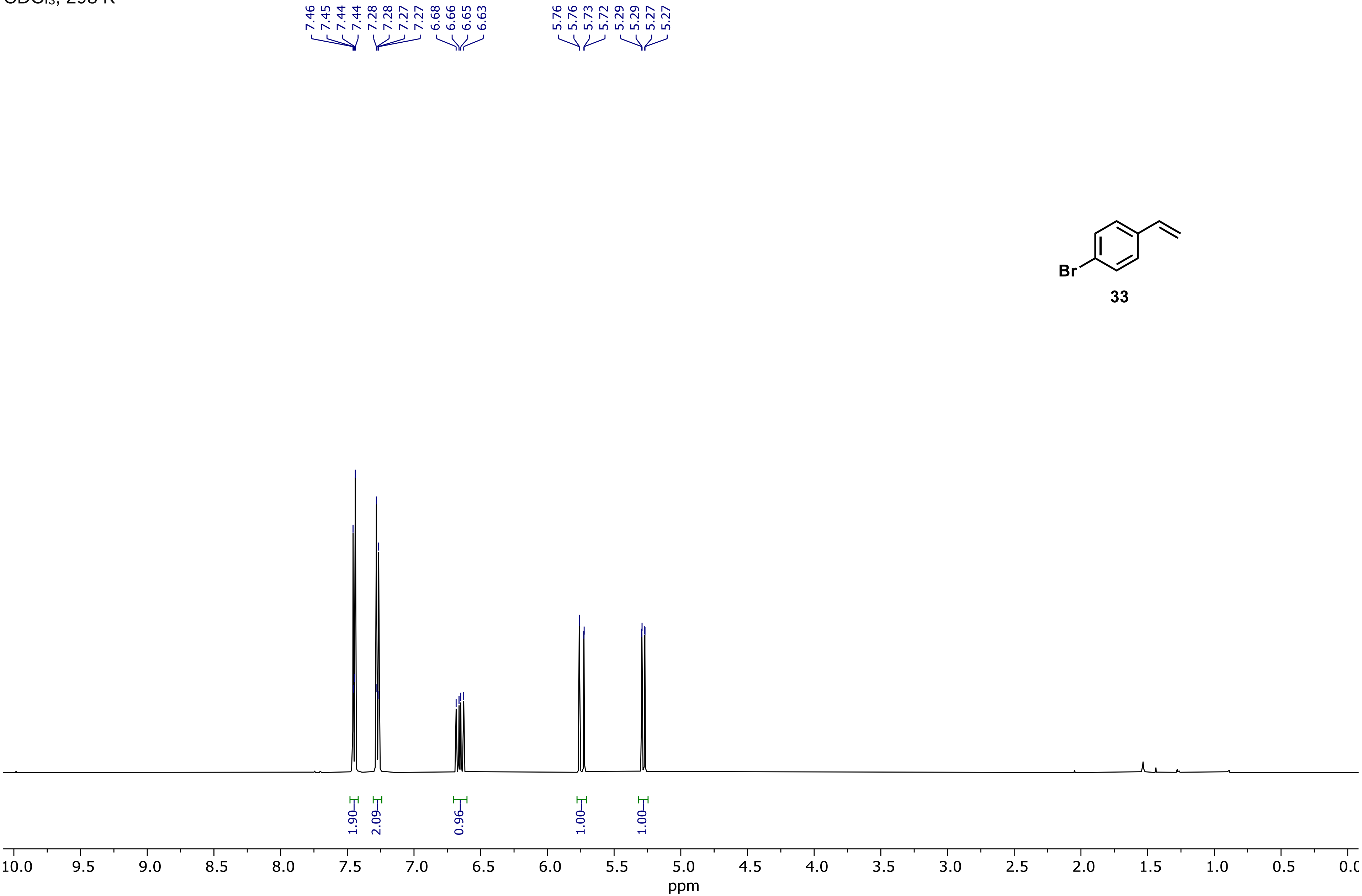

^{13}C NMR of 1-bromo-4-vinylbenzene (33)

$CDCl_3$, 298 K

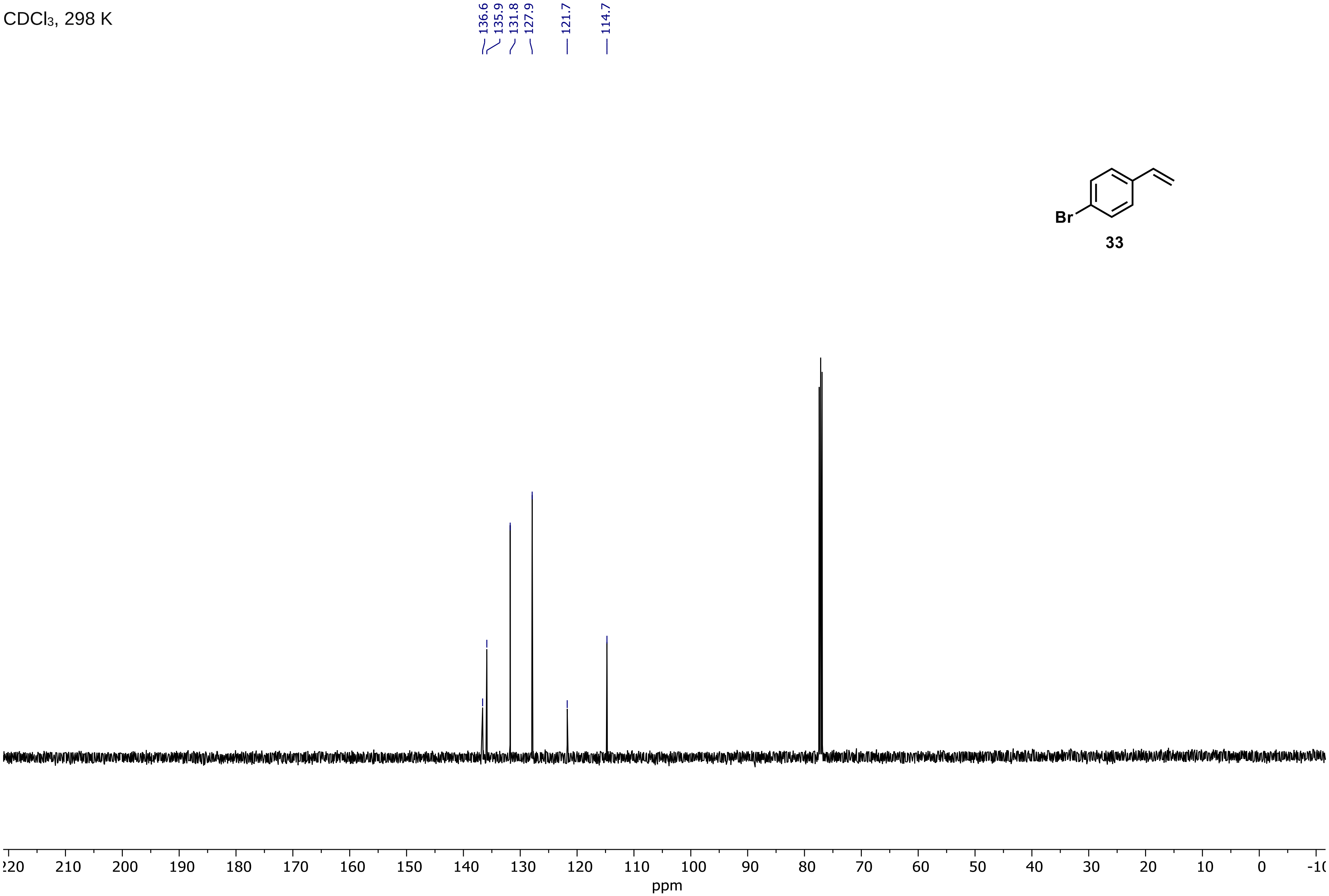

33

¹H NMR of 4-vinylbenzaldehyde (34)

CDCl₃, 298 K

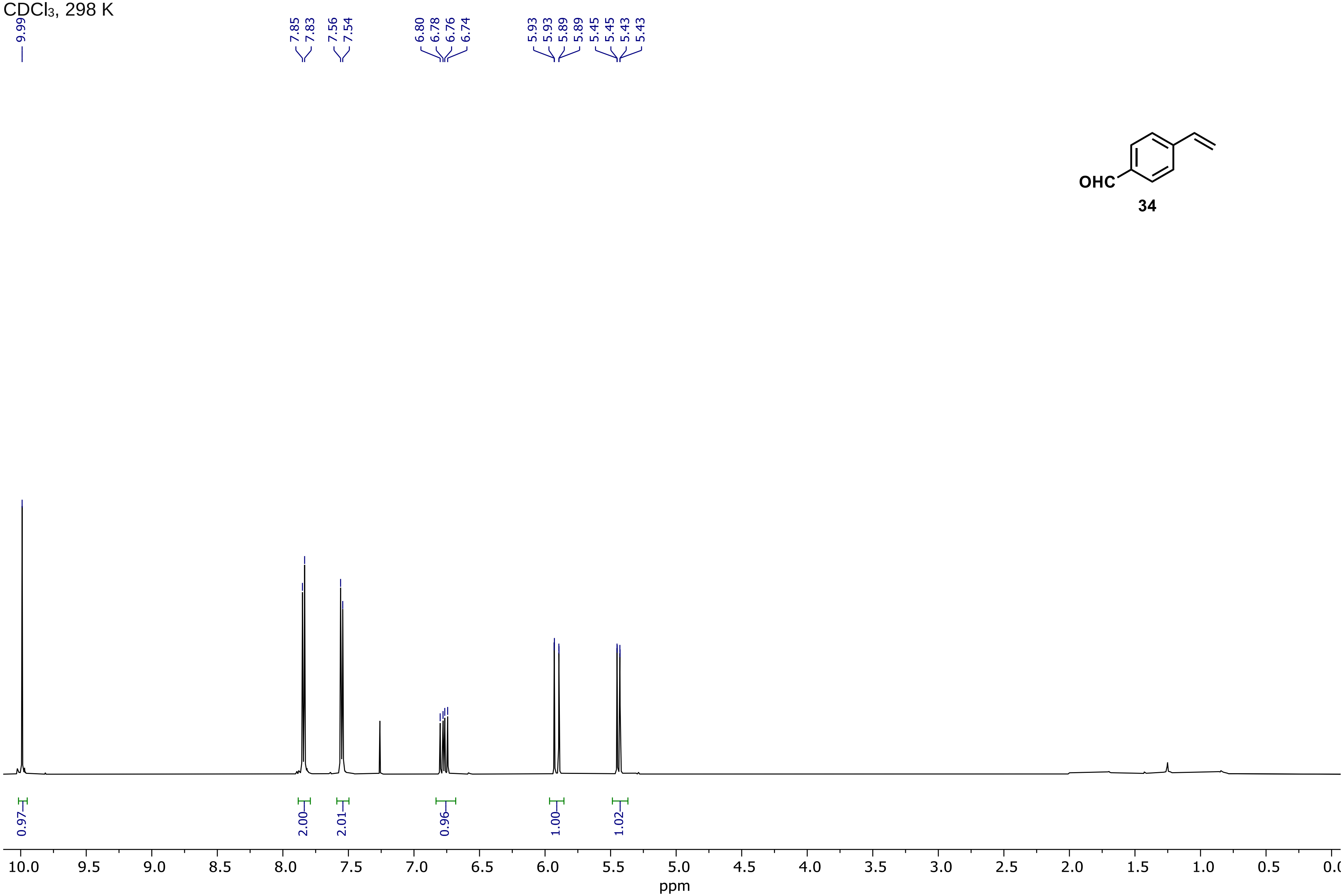

^{13}C NMR of 4-vinylbenzaldehyde (34)

CDCl$_3$, 298 K

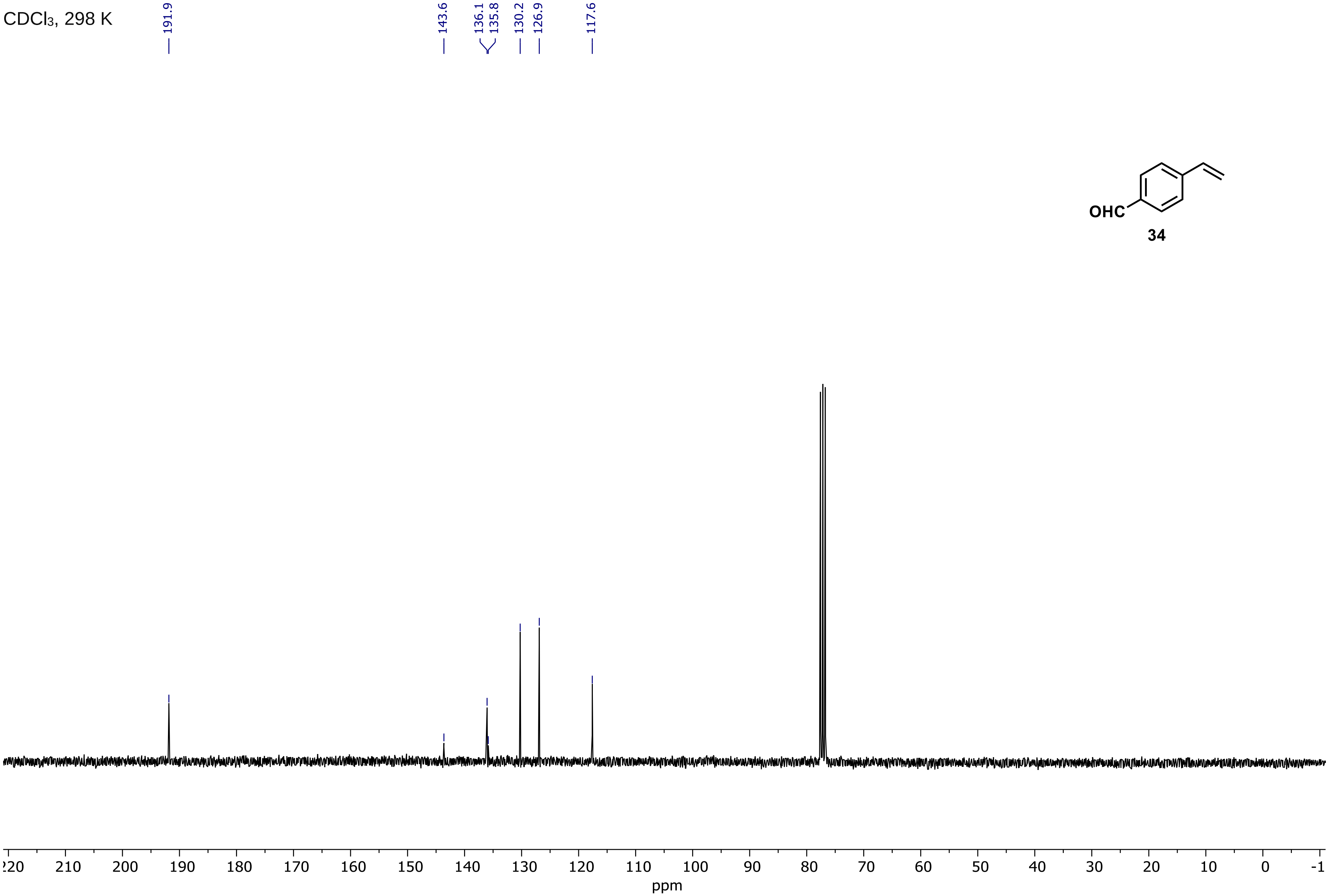

34

^{1}H NMR of 2-vinylbenzo[b]thiophene (35)

CDCl$_3$, 298 K

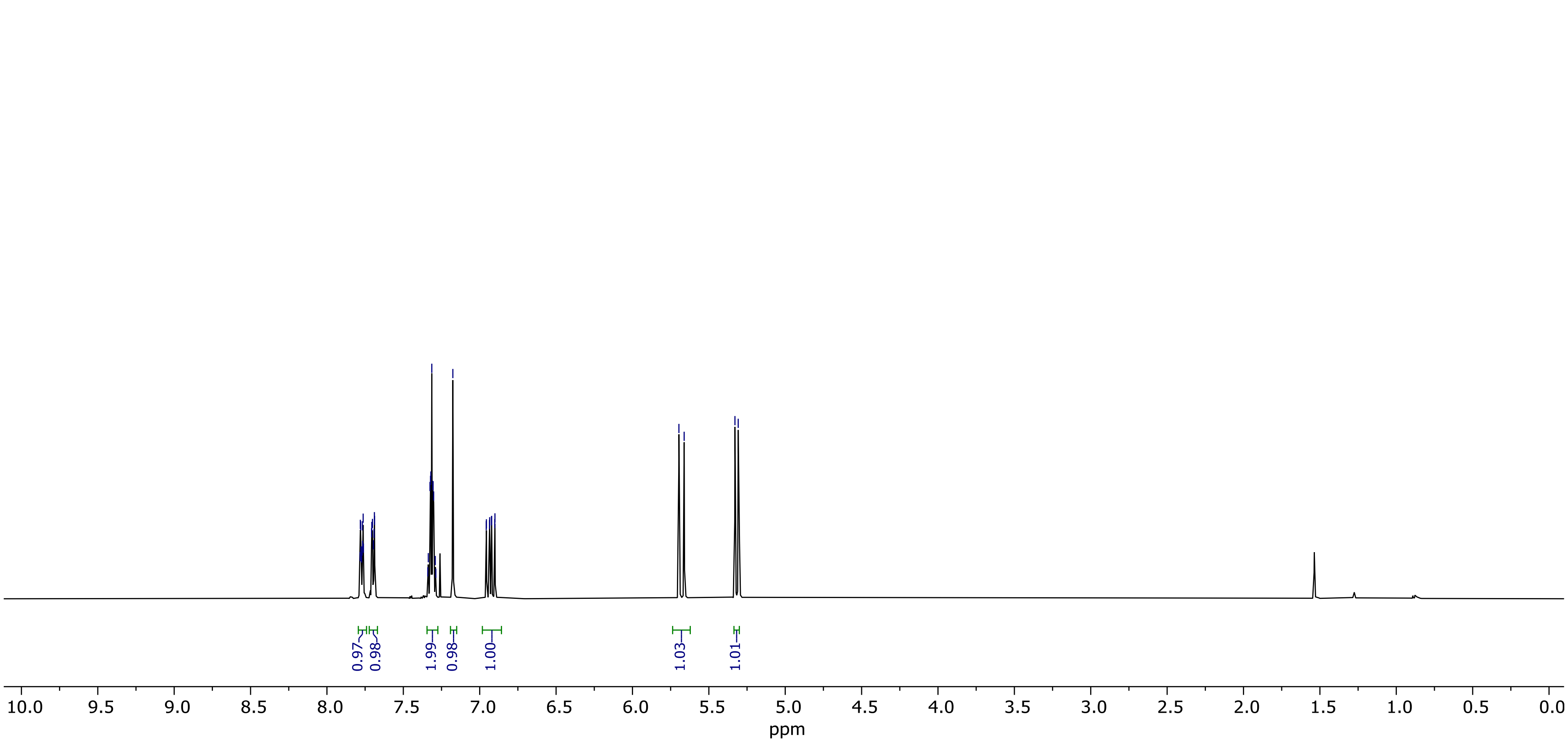

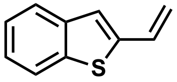

35

^{13}C NMR of 2-vinylbenzo[b]thiophene (35)

CDCl$_3$, 298 K

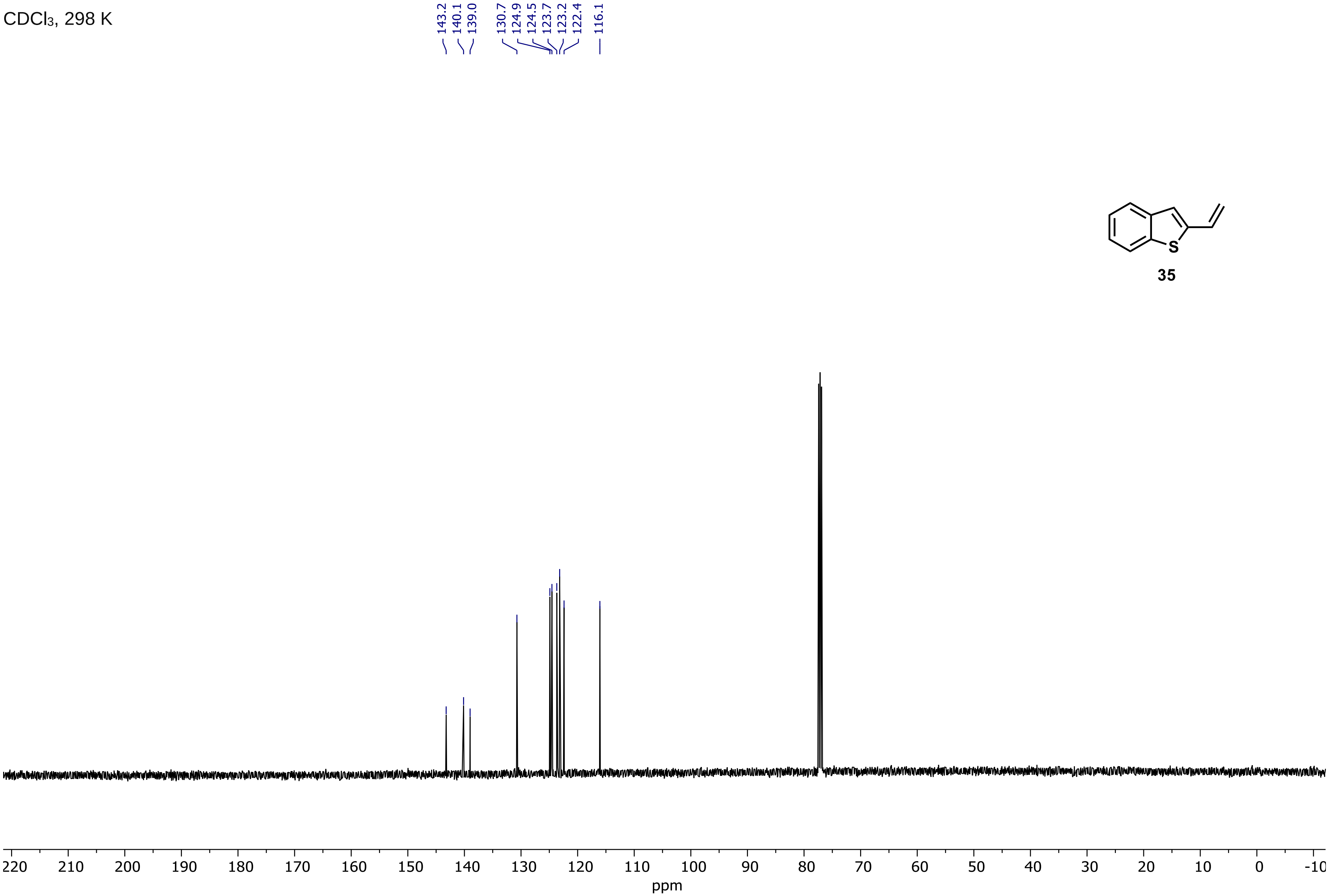

^{1}H NMR of 1-methyl-3-(trifluoromethyl)-5-vinyl-1*H*-pyrazole (36)

$CDCl_3$, 298 K

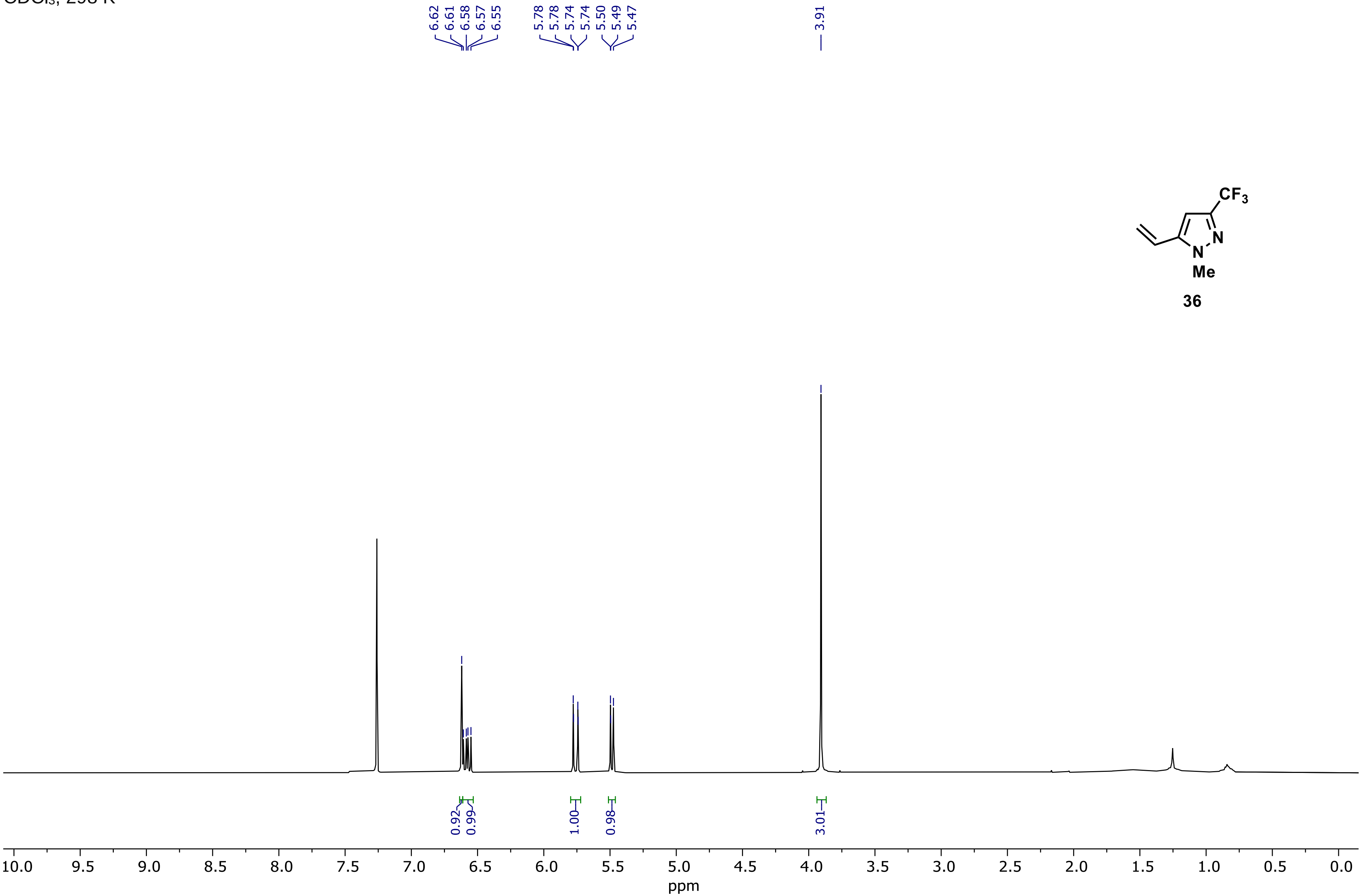

^{13}C NMR of 1-methyl-3-(trifluoromethyl)-5-vinyl-1*H*-pyrazole (36)

CDCl$_3$, 298 K

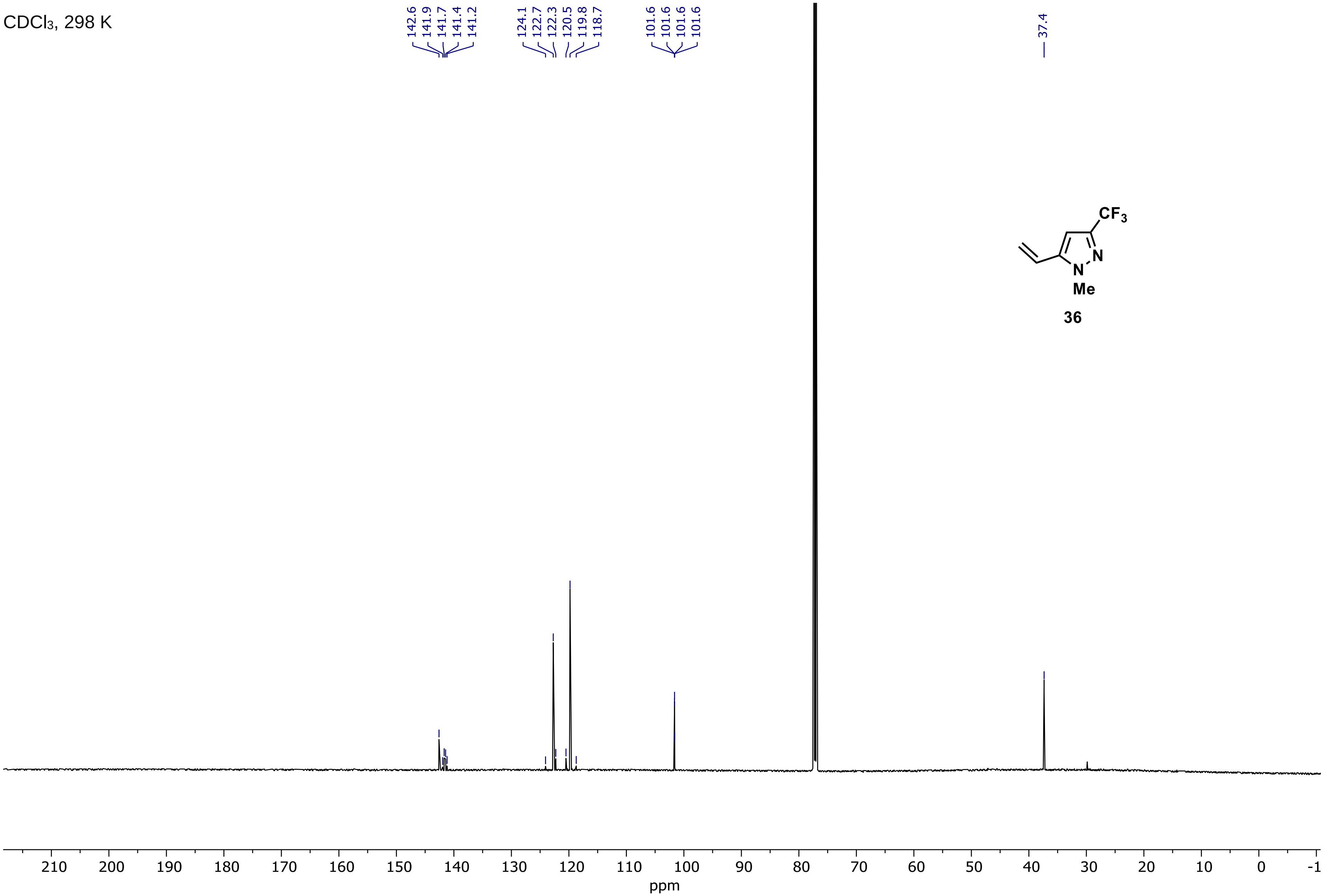

36

^{19}F NMR of 1-methyl-3-(trifluoromethyl)-5-vinyl-1*H*-pyrazole (36)

$CDCl_3$, 298 K

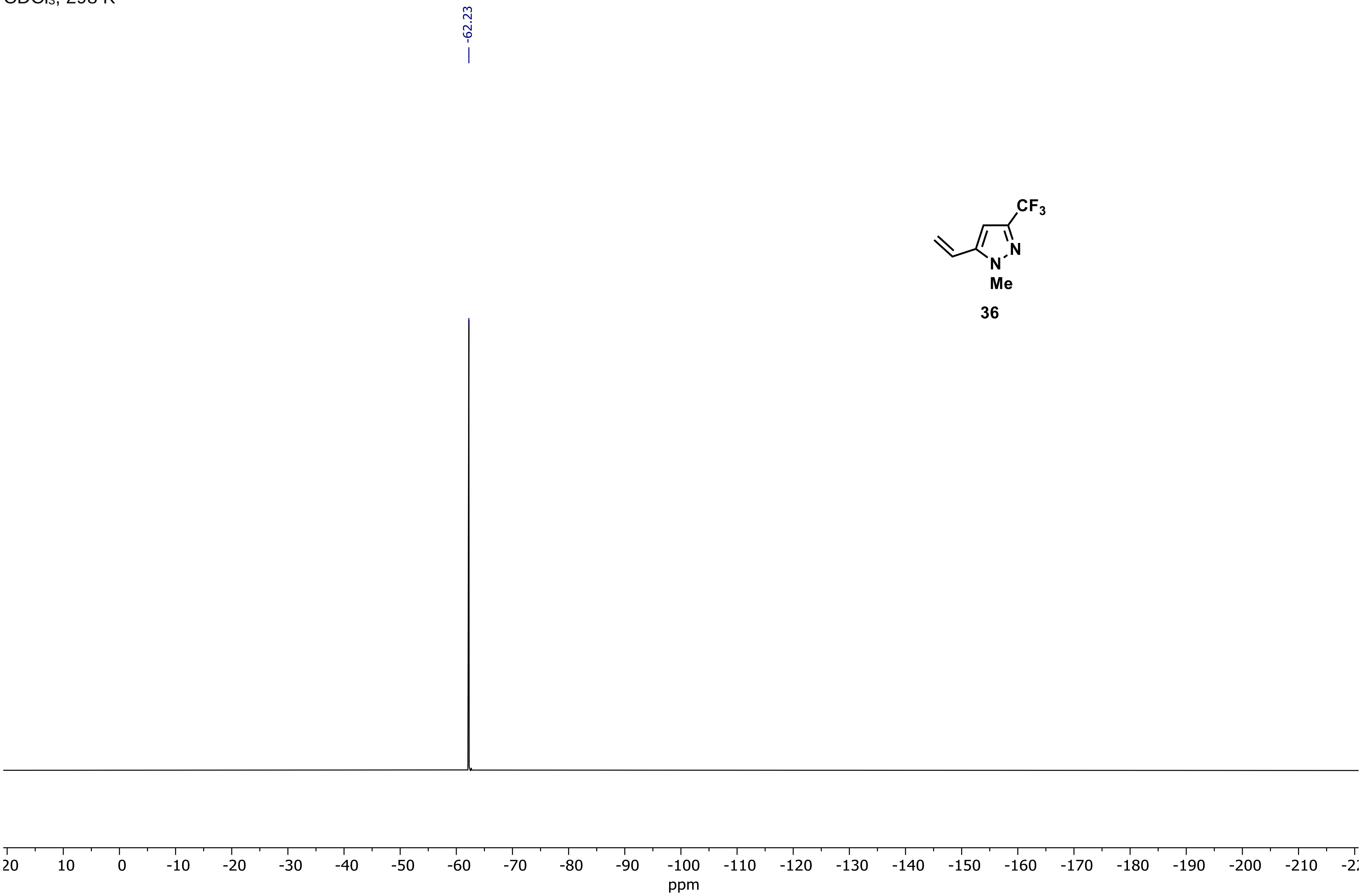

36

^{1}H NMR of 1-chloro-4-(trifluoromethyl)-2-((2-vinylbenzyl)oxy)benzene (37)

CDCl$_3$, 298 K

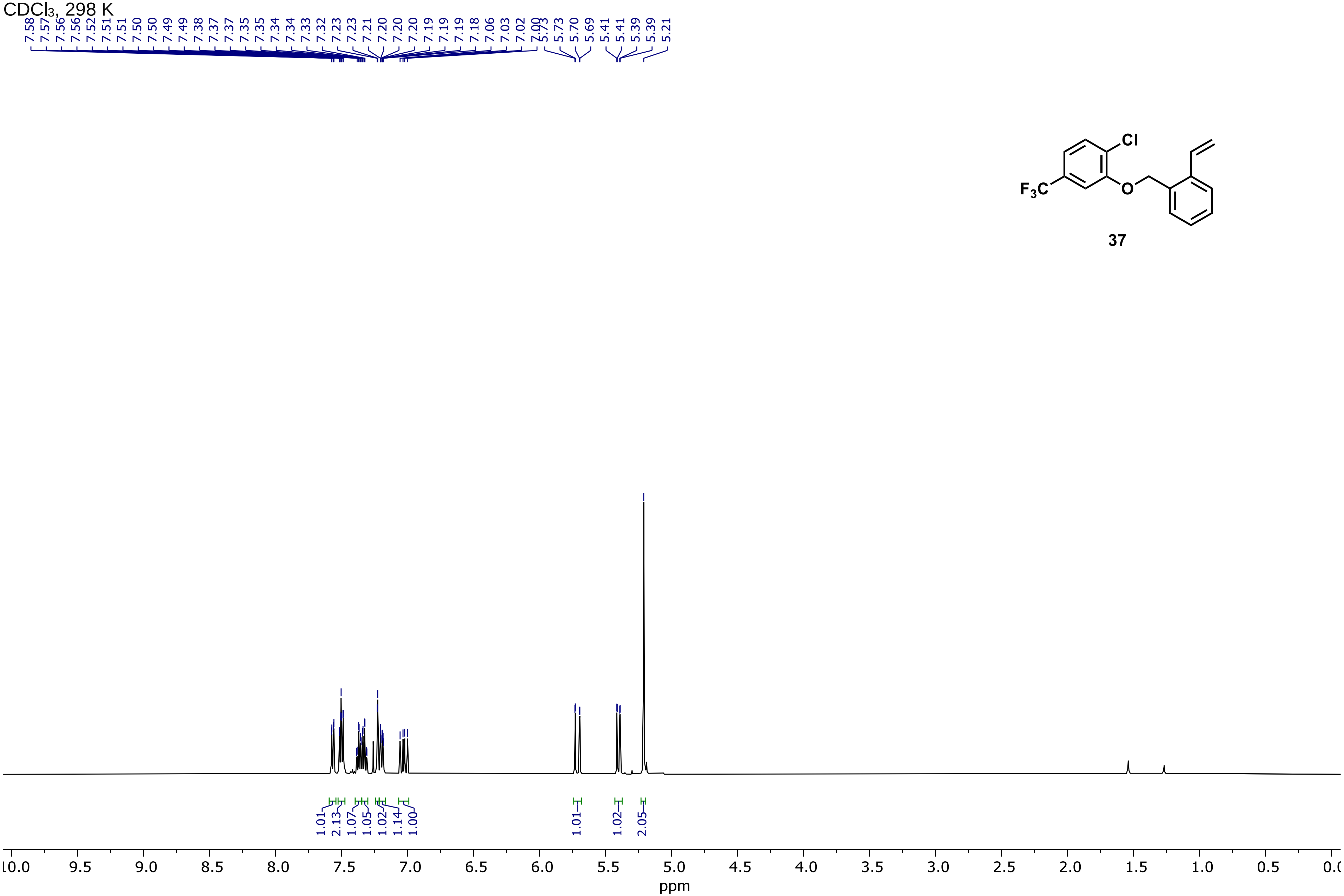

^{13}C NMR of 1-chloro-4-(trifluoromethyl)-2-((2-vinylbenzyl)oxy)benzene (37)

$CDCl_3$, 298 K

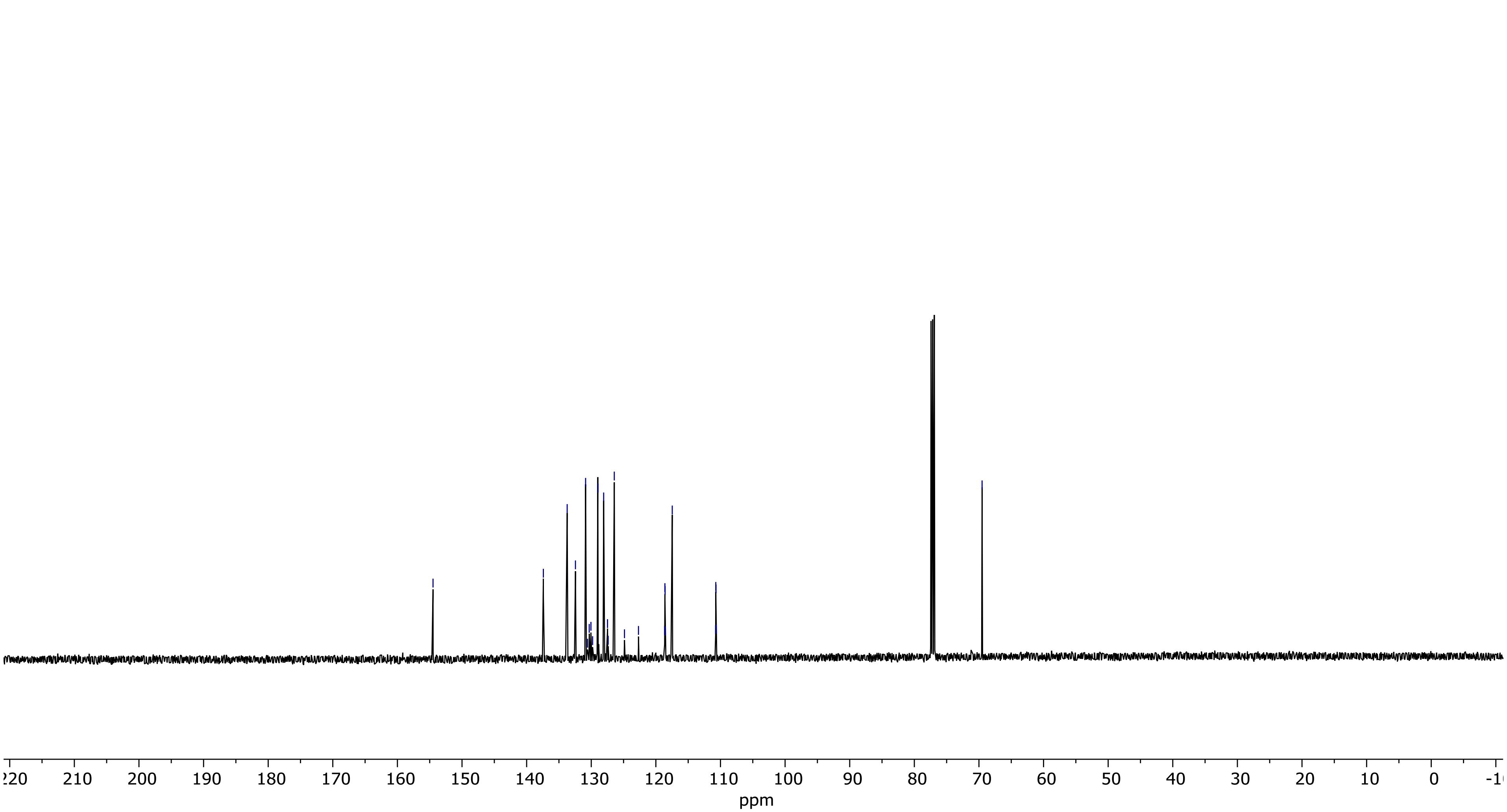

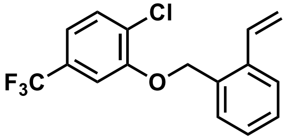

37

^{19}F NMR of 1-chloro-4-(trifluoromethyl)-2-((2-vinylbenzyl)oxy)benzene (37)

$CDCl_3$, 298 K

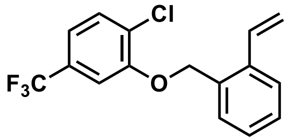

37

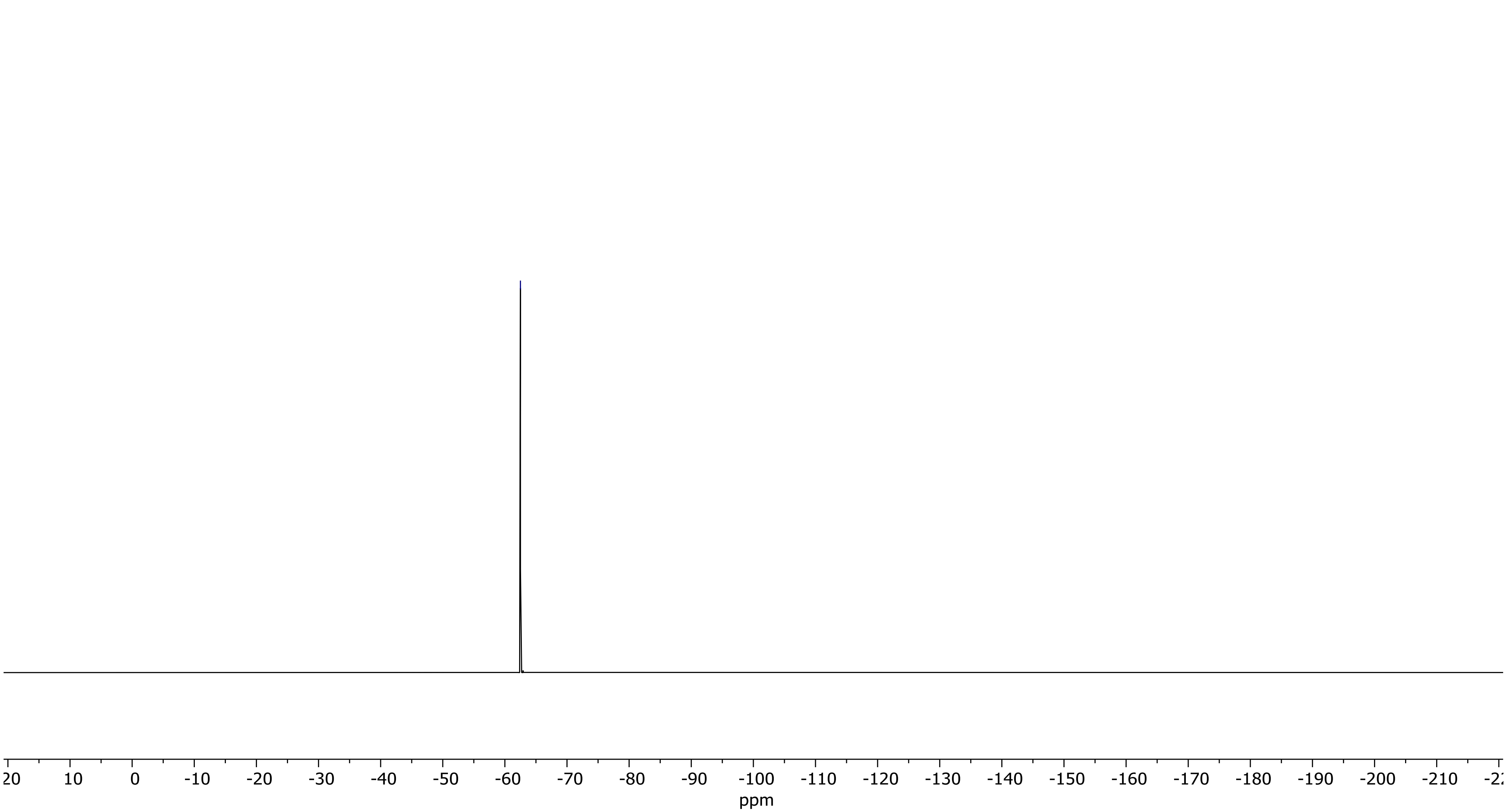

^{1}H NMR of piperidin-1-yl(4-vinylphenyl)methanone (38)

$CDCl_3$, 298 K

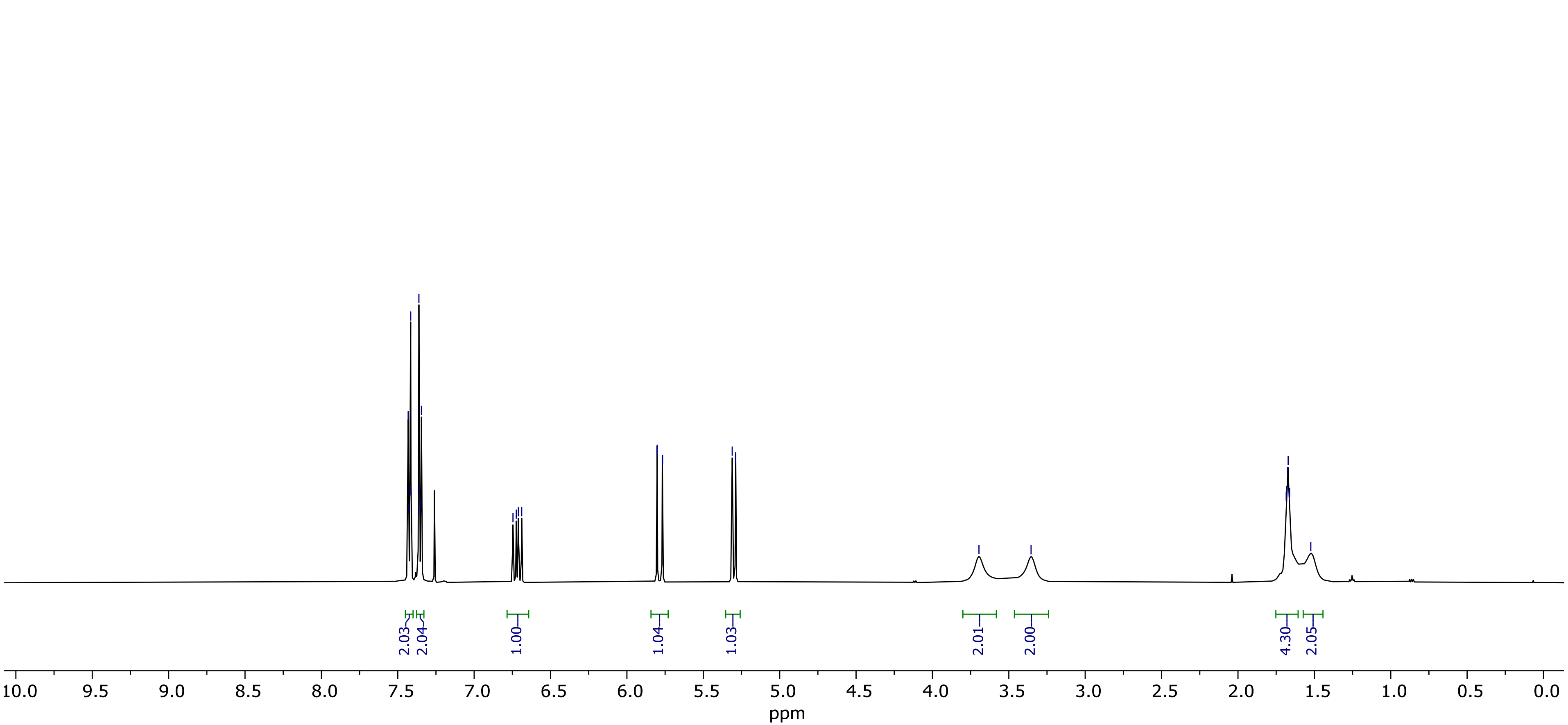

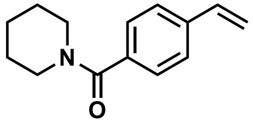

38

^{13}C NMR of piperidin-1-yl(4-vinylphenyl)methanone (38)

CD$_3$CN, 243 K

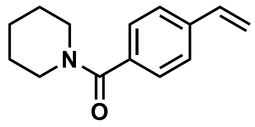

38

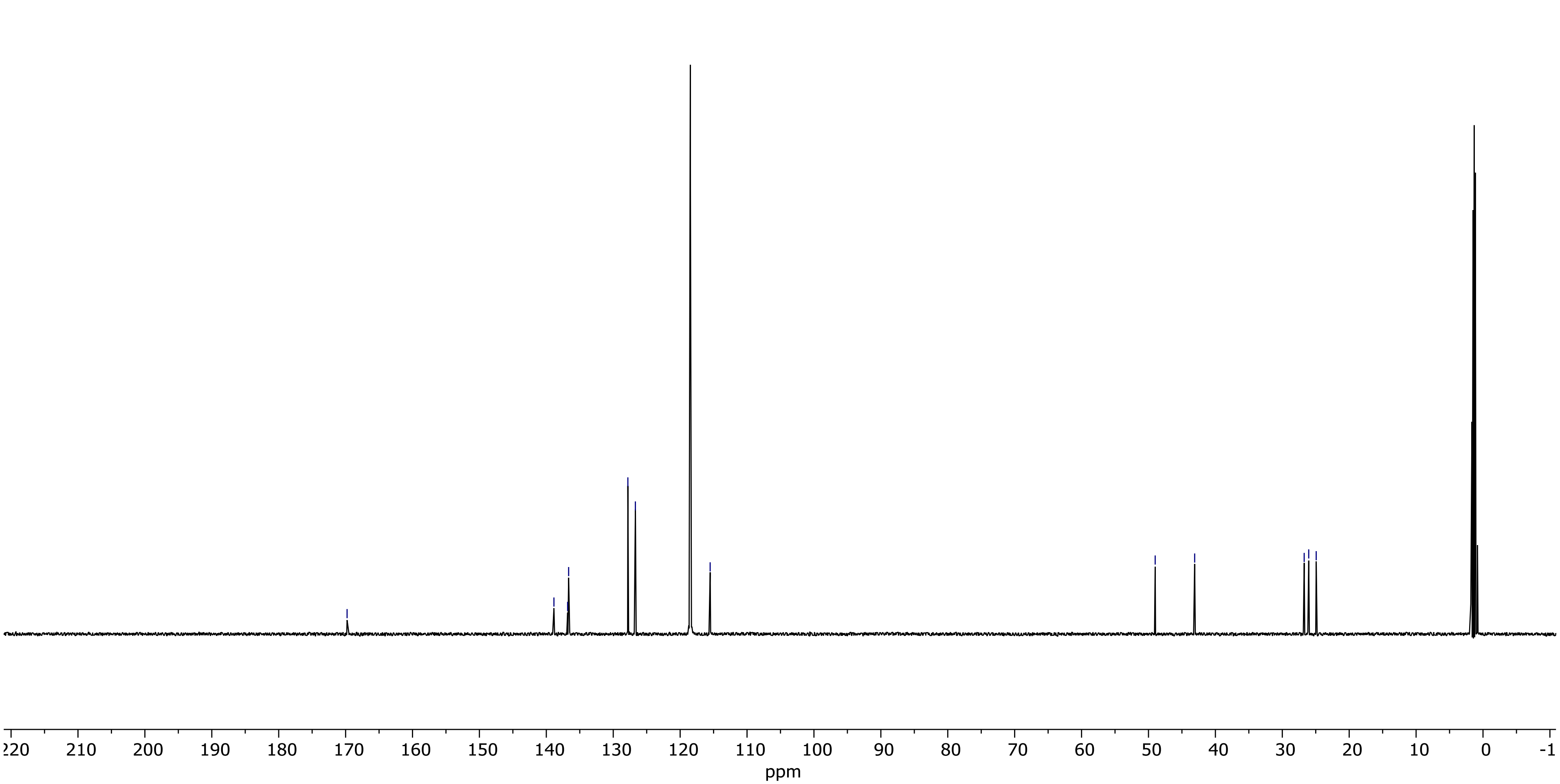

^{1}H NMR of morpholino(3-vinylphenyl)methanone (39)

CDCl3, 298 K

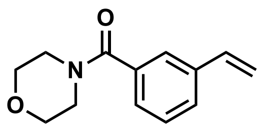

39

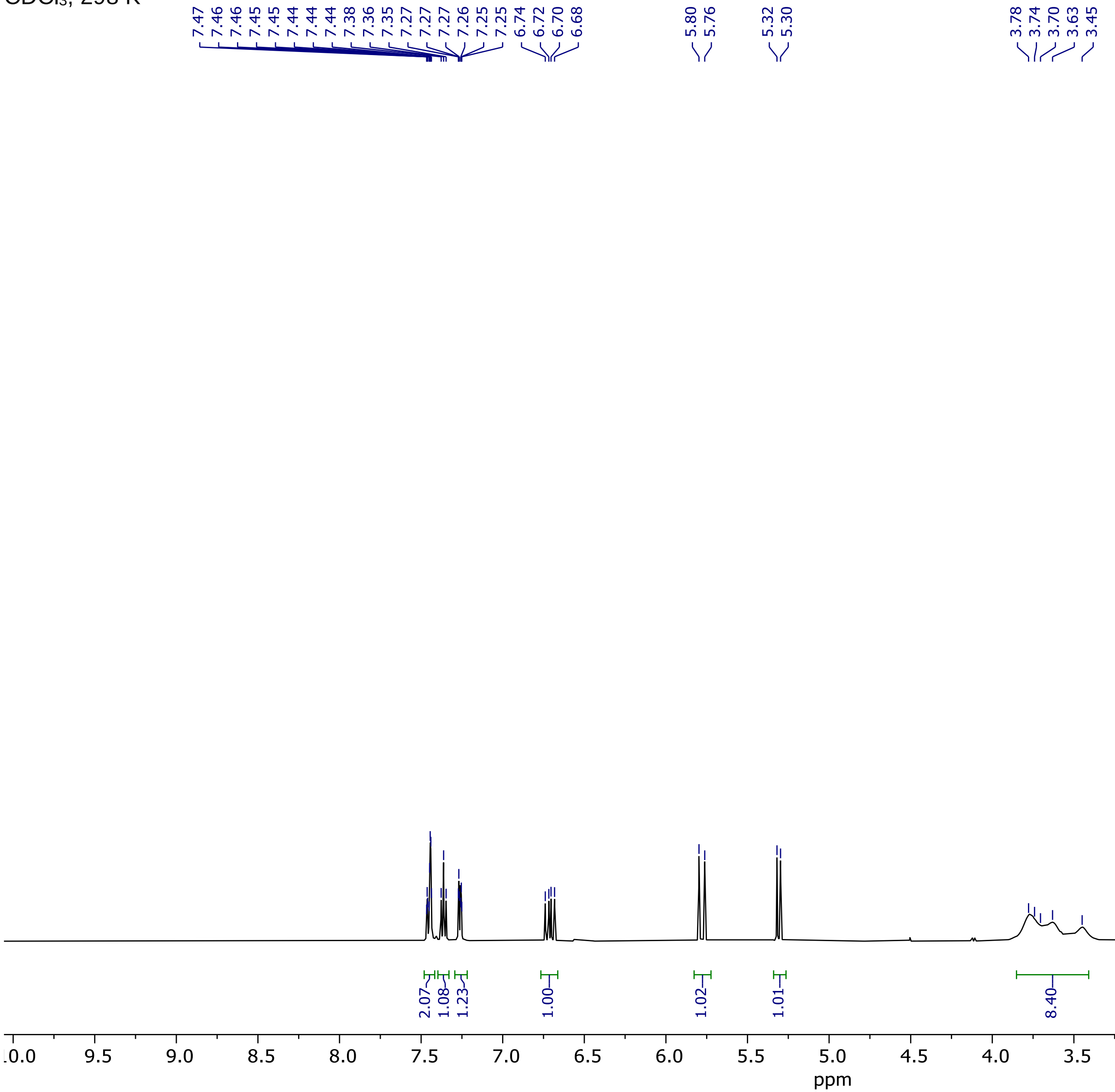

^{13}C NMR of morpholino(3-vinylphenyl)methanone (39)

CDCl$_3$, 298 K

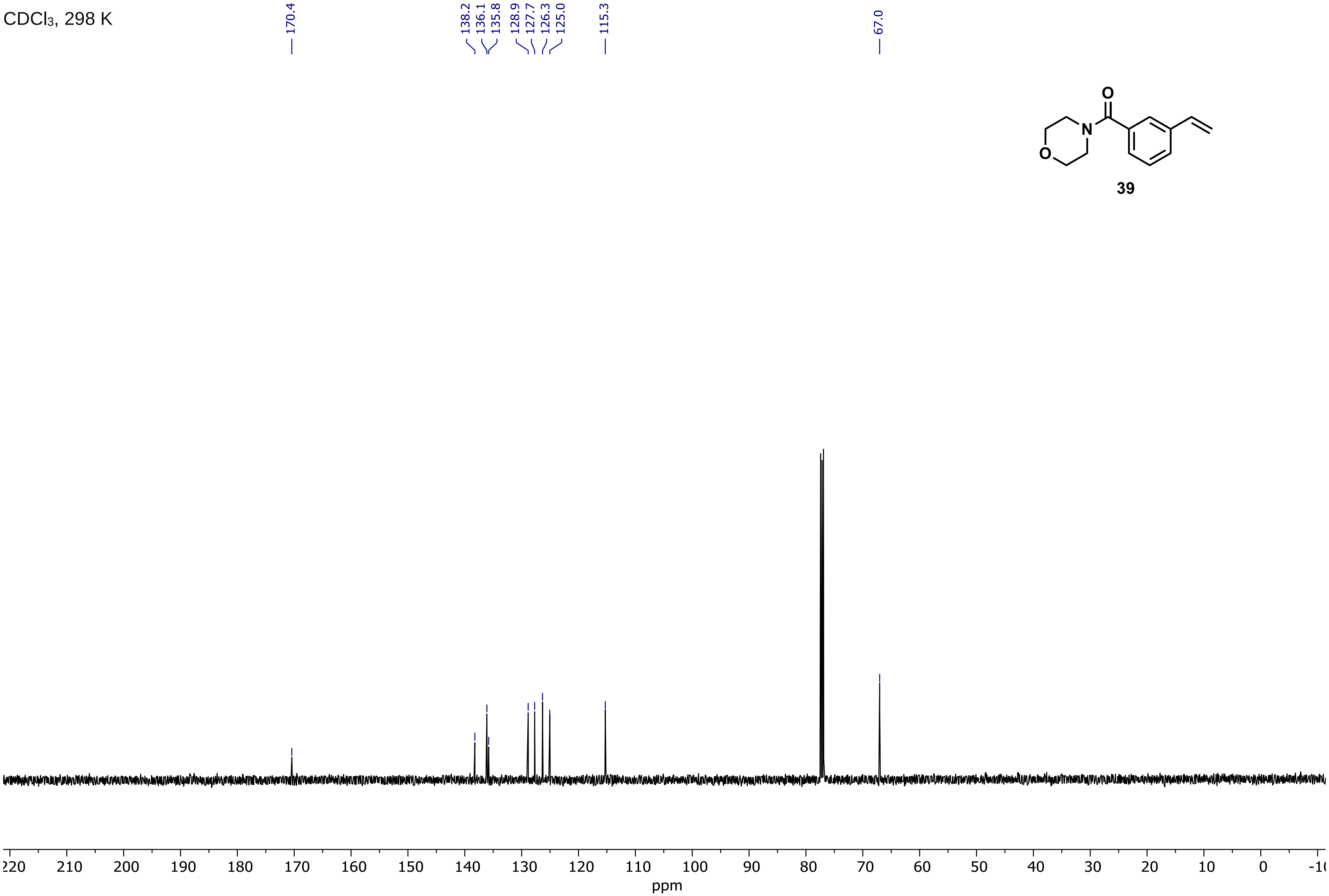

39

^{1}H NMR of (*E*)-4-(buta-1,3-dien-1-yl)-1,1'-biphenyl (40)

CDCl$_3$, 298 K

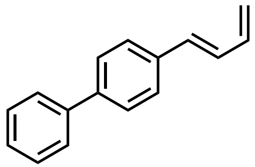

40

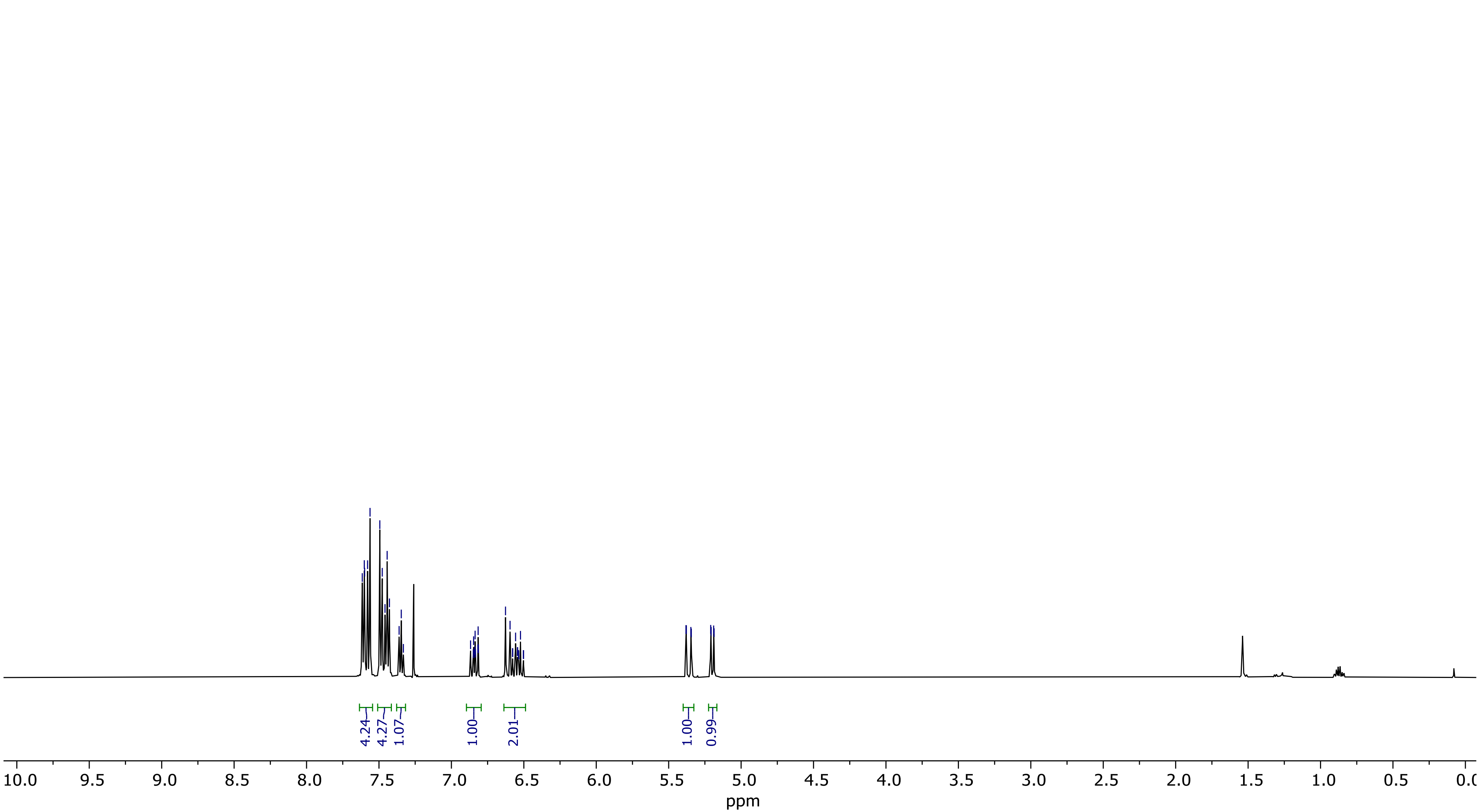

^{13}C NMR of (*E*)-4-(buta-1,3-dien-1-yl)-1,1'-biphenyl (40)

$CDCl_3$, 298 K

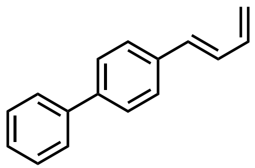

40

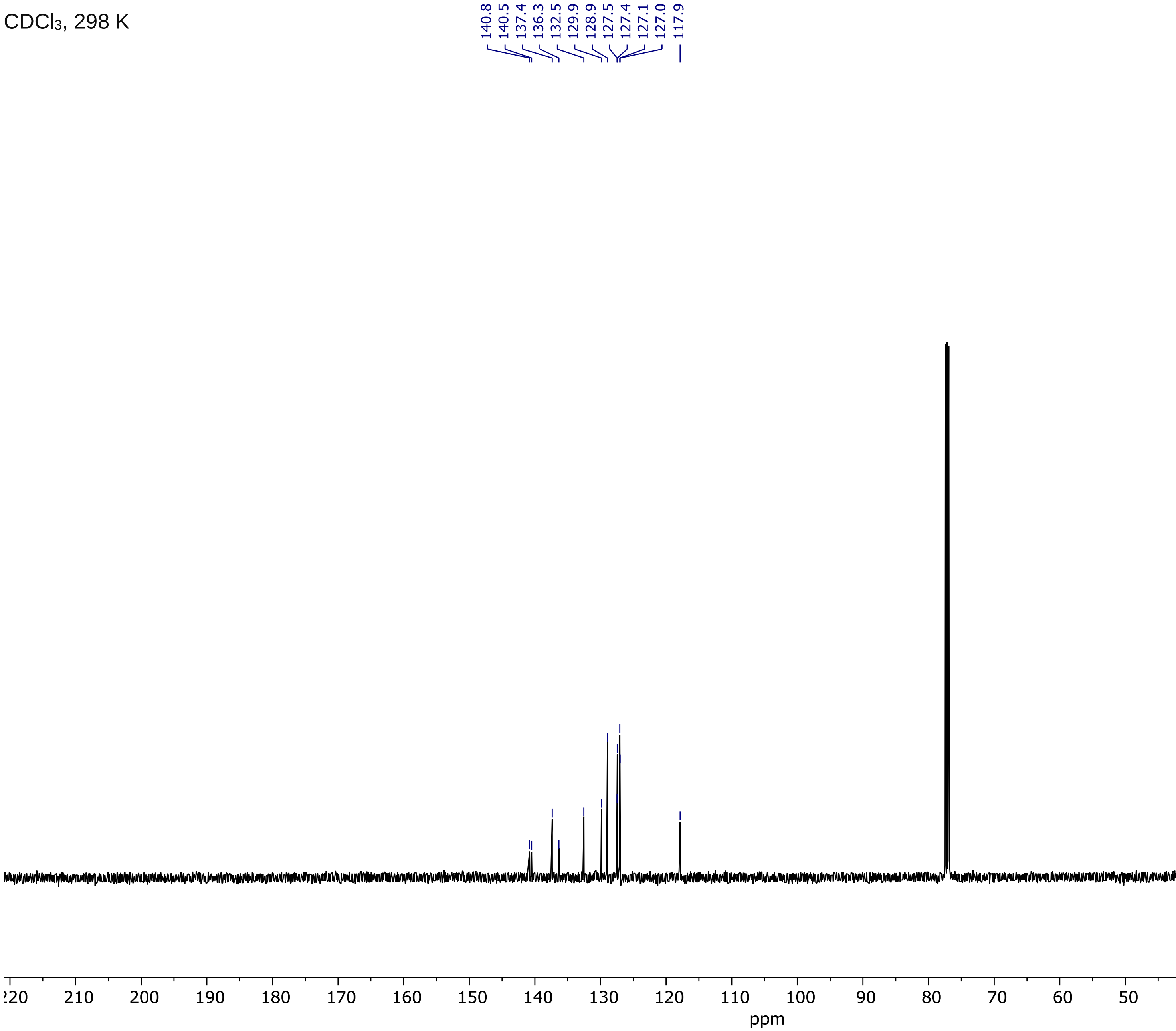

^{1}H NMR of *tert*-butyl (3-vinylphenyl)carbamate (45)

CDCl$_3$, 298 K

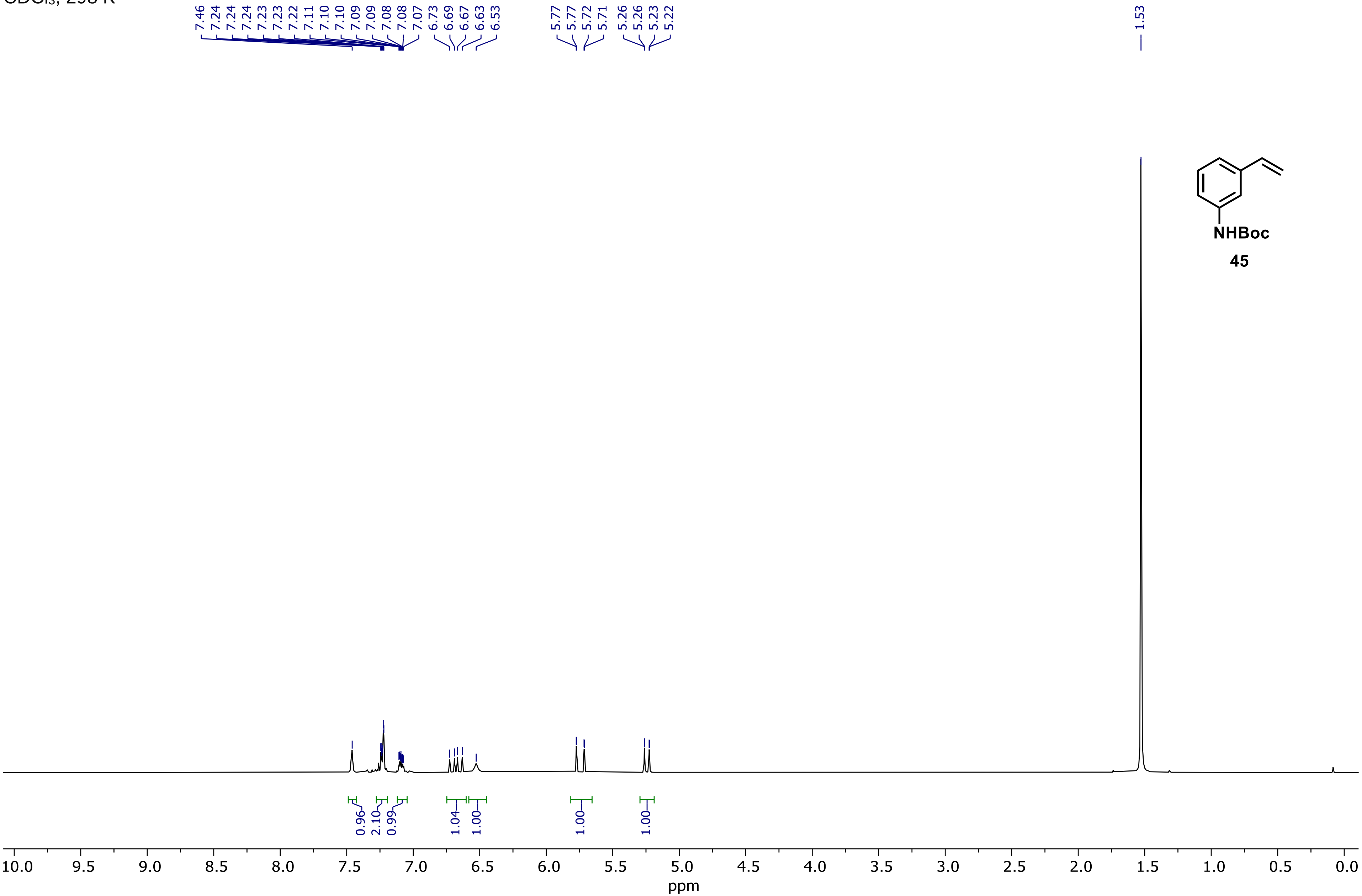

45

^{13}C NMR of *tert*-butyl (3-vinylphenyl)carbamate (45)

CDCl$_3$, 298 K

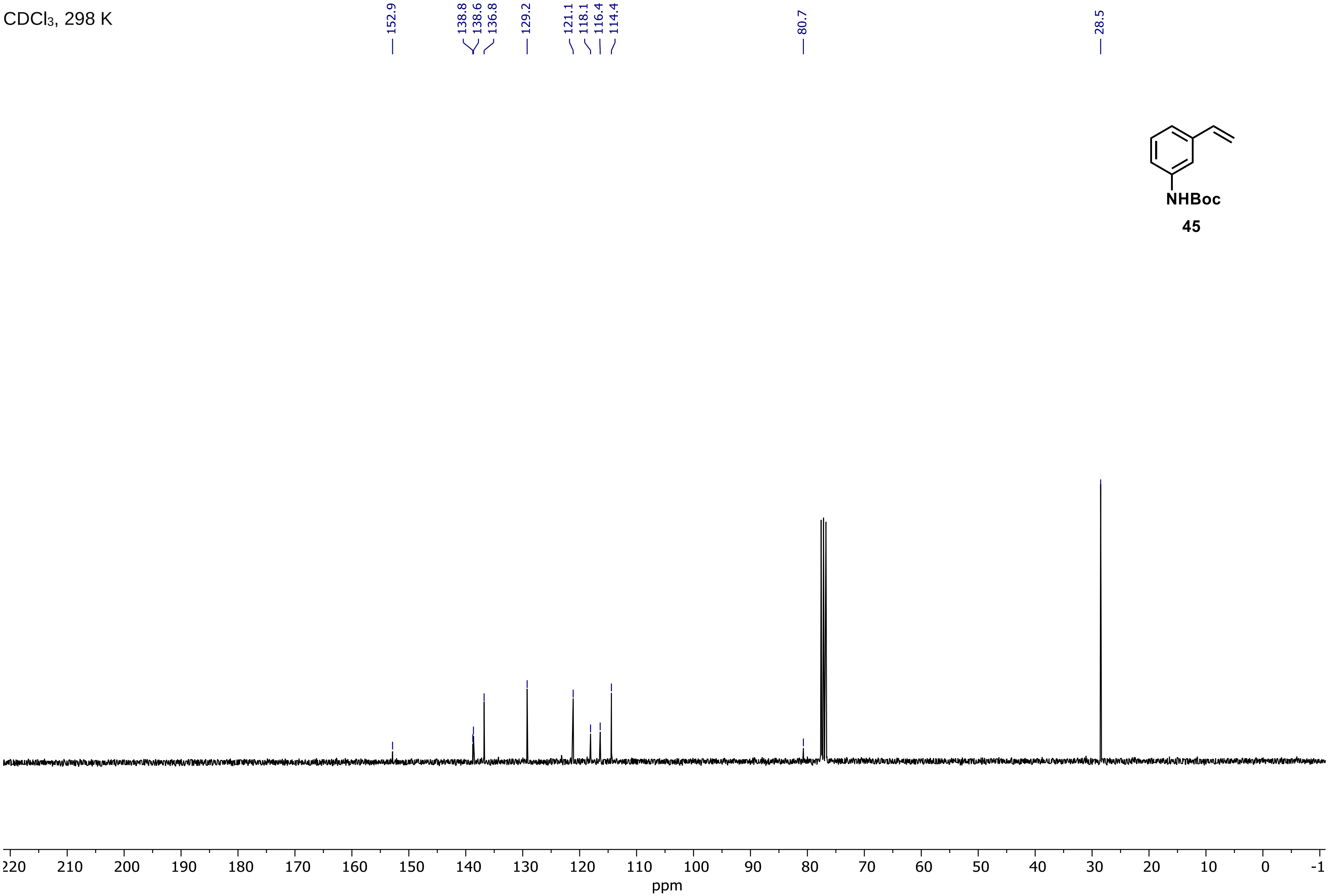

^{1}H NMR of *tert*-butyl [1,1'-biphenyl]-3-ylcarbamate (46)

CDCl₃, 298 K

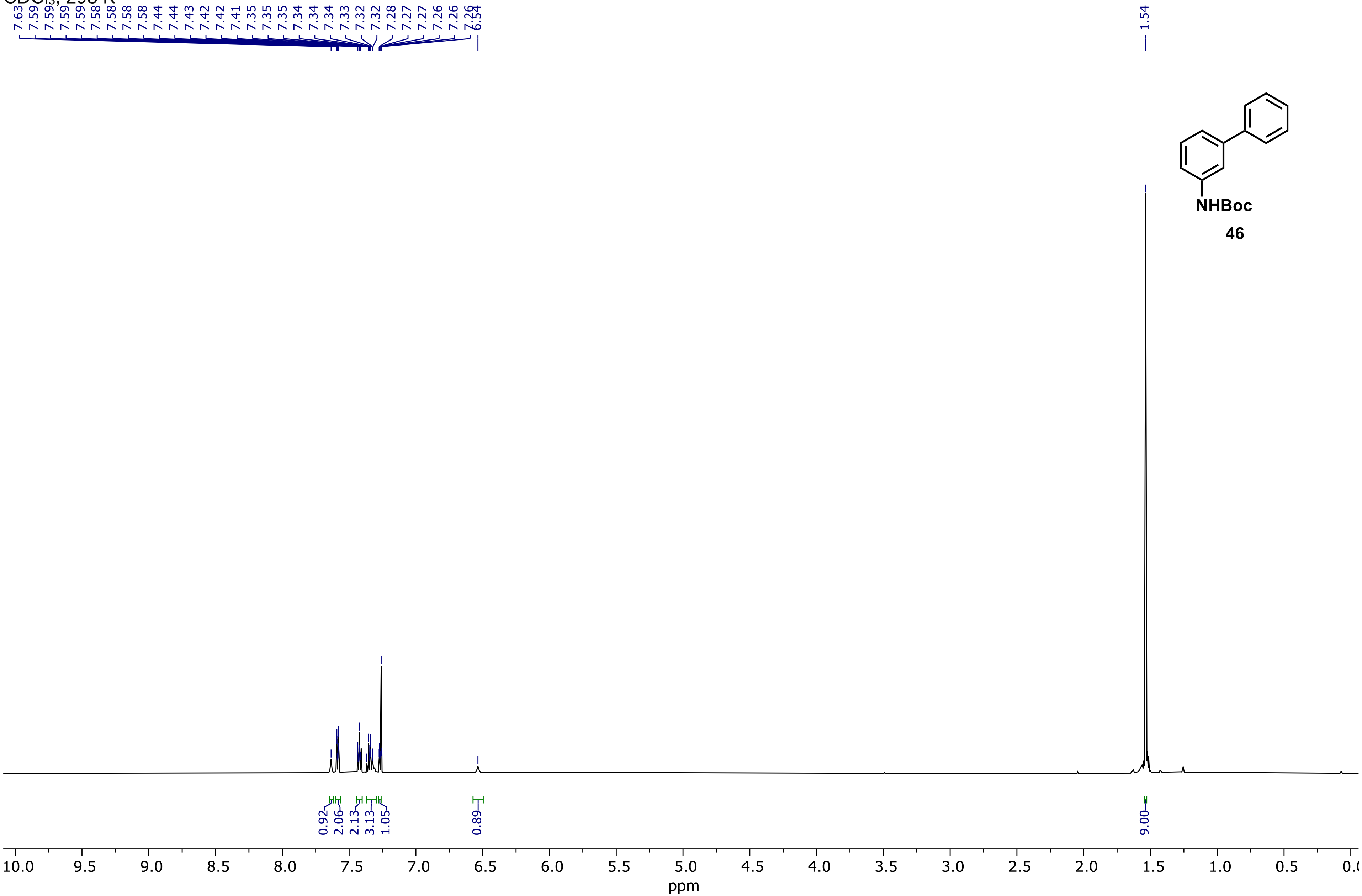

^{13}C NMR of *tert*-butyl [1,1'-biphenyl]-3-ylcarbamate (46)

CDCl$_3$, 298 K

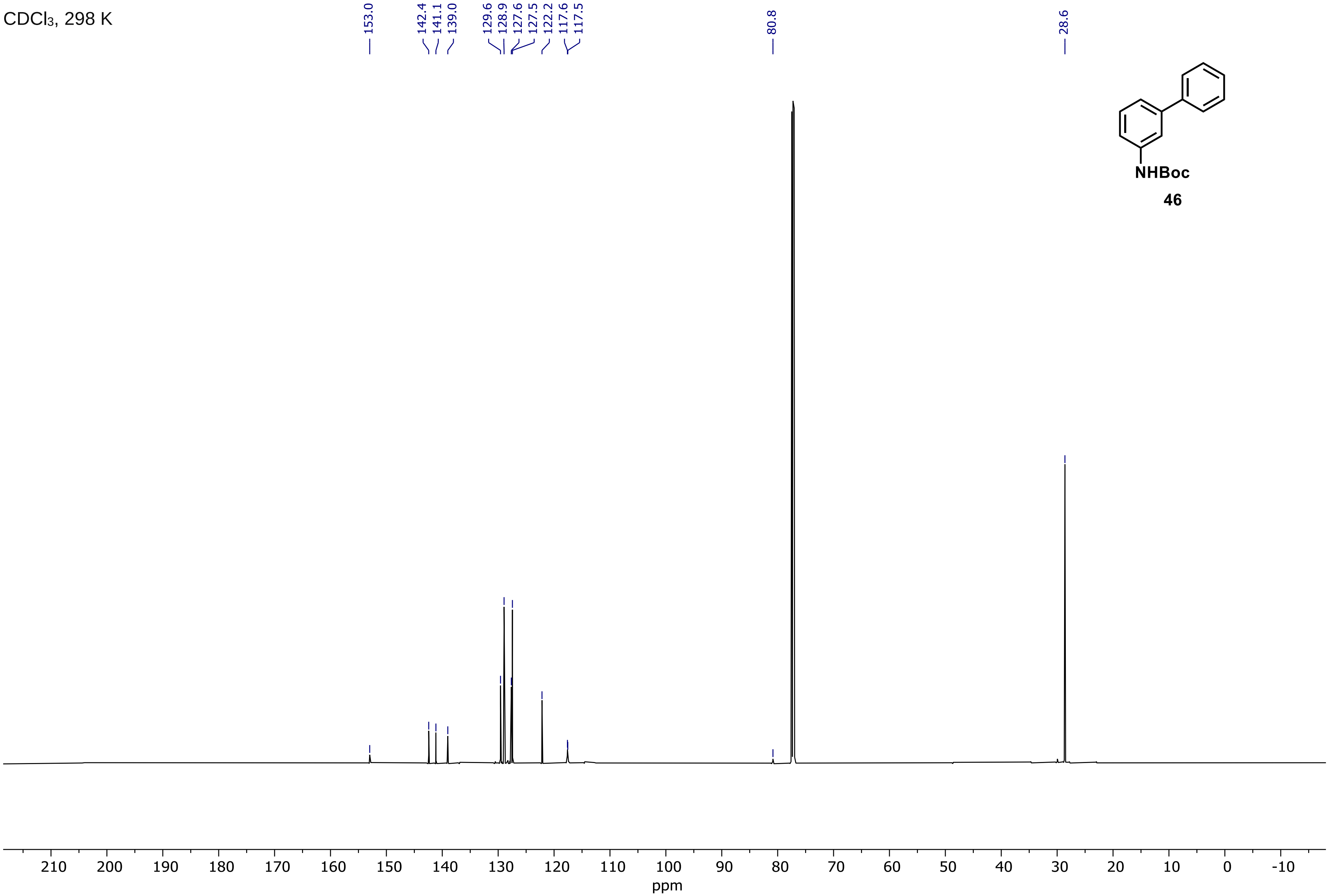

46

References

[1] F. Berger, M. B. Plutschack, J. Riegger, W. Yu, S. Speicher, M. Ho, N. Frank, T. Ritter, *Nature* **2019**, *567*, 223.

[2] M. Yar, E. M. McGarrigle, V. K. Aggarwal, *Angew. Chem. Int. Ed*. **2008**, *47*, 3784.

[3] F. Beltran, I. Fabre, I. Ciofini, L. Miesch, *Org. Lett.* **2017**, *19*, 5042.

[4] A. Younai, B.-S. Zeng, H. Y. Meltzer, K. A. Scheidt, *Angew. Chem. Int. Ed.* **2015**, *54*, 6900.

[5] H. Dai, M. Yang, X. Lu, *Adv. Synth. Catal.* **2008**, *350*, 2

[6] P. Xu, D. Zhao, F. Berger, A. Hamad, J. Rickmeier, R. Petzold, M. Kondratiuk, K. Bohdan, T. Ritter, *Angew. Chem. Int. Ed.* **2020**, *59*, 1956.

[7] V. Pfeifer, M. Certiat, D. Bouzouita, A. Palazzolo, S. Garcia-Argote, E. Marcon, D.-A. Buisson, P. Lesot, L. Maron, B. Chaudret, S. Tricard, I. del Rosal, R. Poteau, S. Feuillastre, G. Pieters, *Chem. Eur. J.* **2020**, *26*, 4988.

[8] H. G. Roth, N. A. Romero, D. A. Nicewicz, *Synlett* **2016**, *27*, 714.

[9] M. Zhou, K. En, Y. Hu, Y. Xu, H. C. Shen, X. Qian, *RSC Adv.* **2017**, *7*, 3741.

[10] S. P. Fritz, J. F. Moya, M. G. Unthank, E. M. McGarrigle, V. K. Aggarwal, *Synthesis* **2012**, *44*, 1584.

[11] M. G. Unthank, N. Hussain, V. K. Aggarwal, *Angew. Chem. Int. Ed.* **2006**, *45*, 7066.

[12] Y. Ye, J. Zhu, Y. Huang, *Org. Lett.* **2021,** *23*, 2386.

[13] P. S. Engl, A. P. Häring, F. Berger, G. Berger, A. Pérez-Bitrián, T. Ritter, *J. Am. Chem. Soc.* **2019**, *141*, 13346.

[14] E. M. Alvarez, M. B. Plutschack, F. Berger, T. Ritter, *Org. Lett.* **2020**, *22*, 4593.

[15] A. Selmani, A. G. Gevondian, F. Schoenebeck, *Org. Lett.* **2020**, *22*, 4802.

[16] B. Lansbergen, P. Granatino, T. Ritter, *J. Am. Chem. Soc.* **2021**, *143*, 7909.

[17] M. Tobisu, Y. Masuya, K. Babaa, N. Chatani, *Chem. Sci.* **2016**, *7*, 2587.

[18] J. Spencer, M. Pfeffer, A. DeCian, J. Fischer, *J.Org.Chem.* **1995**, *60*, 1005.
